# Analysis of pandemic outdoor recreation and green infrastructure in Nordic cities to enhance urban resilience

**DOI:** 10.1038/s42949-022-00068-8

**Published:** 2022-10-03

**Authors:** Nora Fagerholm, Karl Samuelsson, Salla Eilola, Matteo Giusti, Kamyar Hasanzadeh, Anna Kajosaari, Daniel Koch, Silviya Korpilo, Marketta Kyttä, Ann Legeby, Yu Liu, Søren Præstholm, Christopher Raymond, Tiina Rinne, Anton Stahl Olafsson, Stephan Barthel

**Affiliations:** 1 University of Turku, Department of Geography and Geology, 20014 University of Turku, Turku, Finland; 2 Department of Building Engineering, Energy Systems and Sustainability Science, University of Gävle, Gävle, Sweden; 3 Spatial Planning and Transportation Engineering Research Group, Department of Built Environment, School of Engineering, Aalto University, Espoo, Finland; 4 School of Architecture, KTH Royal Institute of Technology, Stockholm, Sweden; 5 Helsinki Institute of Sustainability Science, University of Helsinki, Helsinki, Finland; 6 Ecosystems and Environment Research Programme, Faculty of Biological and Environmental Sciences, University of Helsinki, Helsinki, Finland; 7 Department of Geosciences and Natural Resource Management, University of Copenhagen, Copenhagen, Denmark; 8 Department of Economics and Resource Management, Faculty of Agriculture and Forestry, University of Helsinki, Helsinki, Finland; 9 Department of Landscape Architecture, Planning and Management, Swedish University of Agricultural Sciences, Uppsala, Sweden; 10 Stockholm Resilience Centre, Stockholm University, Stockholm, Sweden

**Keywords:** Geography, Sustainability

## Abstract

Recent empirical research has confirmed the importance of green infrastructure and outdoor recreation to urban people’s well-being during the COVID-19 pandemic. However, only a few studies provide cross-city analyses. We analyse outdoor recreation behaviour across four Nordic cities ranging from metropolitan areas to a middle-sized city. We collected map-based survey data from residents (*n* = 469–4992) in spring 2020 and spatially analyse green infrastructure near mapped outdoor recreation sites and respondents’ places of residence. Our statistical examination reveals how the interplay among access to green infrastructure across cities and at respondents’ residential location, together with respondents’ socio-demographic profiles and lockdown policies or pandemic restrictions, affects outdoor recreation behaviour. The results highlight that for pandemic resilience, the history of Nordic spatial planning is important. To support well-being in exceptional situations as well as in the long term, green infrastructure planning should prioritise nature wedges in and close to cities and support small-scale green infrastructure.

## Introduction

Research across a wide spectrum of disciplines has empirically explored the relationships between nature or ecosystems and human well-being, with the conclusion that contact with nature generally makes people happier and healthier, both physically and mentally (e.g., refs. ^1–3^). Hence, access to green infrastructure has been identified as a necessary component of healthy urban life (e.g., refs. ^4,5^). Green infrastructure refers to a strategically planned network of green (land) and blue (water) spaces that can improve both environmental conditions and citizens’ quality of life^6,7^. Access to urban green infrastructure is typically unequal across urban landscapes, varying with social and economic status of urban areas^8^. This is particularly prominent in several US cities, which show that good access to urban vegetation is strongly correlated with higher education and income and negatively associated with being a person of colour^9^. Also, across eight European countries, evidence shows older age groups and people of lower socio-economic status visit green space less frequently^10^.

Proximity to and availability of green infrastructure are key determinants of access to it and of well-being outcomes. Many studies have reported on the close linkage between use frequencies and distance from home to green space (e.g., refs. ^11–13^). Hence, official planning recommendations highlight a maximum walking distance of 300 metres to the nearest green space^14^. In terms of the specific characteristics of green and blue spaces (e.g., size, shape, facilities, or biodiversity), the findings are more mixed. For example, across European studies, no clear patterns linked to specific user groups and their preferences were identified^15^.

Green infrastructure holds particular importance to people during crises. In Fukushima, Japan, green space restoration was set as a priority to support recovery from the tsunami and the subsequent nuclear disaster in 2011^16^. During the economic recession that the US encountered in 2008, green areas provided stress relief, and urban foraging increased as a means of tackling food insecurity^17^. Also, in the ongoing pandemic caused by the COVID-19 virus^18^, access to urban green infrastructure is a crucially important source of resilience supporting the well-being of urban dwellers^19^. Recent empirical research has intensively explored the role of green infrastructure and outdoor recreation behaviour during the COVID-19 pandemic across the Western world. For example, survey data from the US show that frequency of use and visits to urban and peri-urban natural areas, along with their perceived importance for mental and physical health, increased overall^20^. However, concerns were raised among different populations related to both access and safety due to crowding^21^. Furthermore, a survey from Vermont, US, showed the values ranked as more important during the early months of the pandemic factored into groups of ‘Nurture and Recreation’ and ‘Inspiration and Nourishment’^22^. The former group was more likely to have been ranked important by urban respondents and women. In England, a national survey showed that 40% of adults reported they had spent more time outside during spring 2020^23^, and mobile tracking data confirms green space use increased due to lockdown^24^. However, people were less likely to have visited natural spaces if they lived in an area of high deprivation, had a low level of income or education, or were not working^23^. A survey by Poortinga et al.^25^ in the UK showed that during COVID-19 nearby public green spaces were particularly important for households without a private garden.

In the Northern European context, visitor monitoring in nature areas showed increased use of green areas in cities and nearby nature reserves. For example, in Finland, national parks saw 20% more visitors in 2020 compared to the previous year^26^, and in Denmark, there was an 80–125% increase in visitation of specific peri-urban forest and nature areas^27^. Also, mobile tracking data show urban dwellers more frequently visited natural settings during the pandemic^28^. Map-based survey data from Nordic cities revealed that forest land cover was the best predictor of the well-being benefits of outdoor recreation sites’^29^, that the outdoor recreation sites mapped by intensive users of natural recreational areas and outdoor-oriented users became more dispersed during the pandemic^30^, and that outdoor recreation increased at sites perceived to offer multiple nature-related values and activities^31^.

Only a few studies provide comparative analyses of outdoor recreation during the pandemic. Across 47 countries, Google Mobility Reports show that park visitation increased in most countries in comparison to the pre-pandemic baseline and that park visitation positively correlated with restrictions on social gathering, movement, and the closure of workplaces and indoor recreational spaces^32^. Across nine countries, Pouso et al.^33^ showed that during the COVID-19 lockdown, emotions were more positive among individuals with accessible outdoor spaces and blue-green elements in their views. Ugolini et al.^34^ conducted a survey in five countries with different pandemic-related social restrictions imposed. They report that the most sought-after activities in green spaces were physical exercise and relaxing. Behavioural changes related to proximity were observed, with an increase in people walking to small urban gardens nearby (e.g., in Italy) or tree-lined streets (e.g., in Spain, Israel), and people travelling by car to green areas outside the city (e.g., in Lithuania).

The studies highlighted above confirm the importance of, but also the varying access to, urban green infrastructure during the pandemic. Building on this literature, we find there are important lessons to learn about outdoor recreation behaviour under the pandemic conditions in the Nordic context. Nordic cities perform well in terms of green infrastructure planning^35^ and provision of green-blue infrastructure in dense urban areas^36^. However, in all the large Finnish cities and in some Danish ones, the areal extent of green space has been decreasing slightly or even strongly in the period of 2006–2012, while being mostly stable in Sweden^37^. These trends highlight the fact that green infrastructure preservation and development has to be seriously considered in urban areas even in Nordic cities. It is particularly important for urban resilience in times of crisis. However, there is no clear picture of what differences exist in terms of proximity and availability of urban green infrastructure during the COVID-19 pandemic or what could be learned in terms of future spatial planning, including the delivery of ambitious Urban Greening Plans required under the EU Biodiversity Strategy for 2030^38^.

Our aim is to fill this gap by providing evidence based on empirical data on outdoor recreation behaviour collected during the early phase of the pandemic in spring 2020 across four Nordic cities, ranging from large metropolitan areas (Copenhagen, Denmark; Stockholm, Sweden: Helsinki, Finland) to a middle-sized city (Turku, Finland). We collected online survey data (*n* = 469–4992) asking residents to map (as points) their outdoor recreation sites and residential location. We analyse these data from the perspectives of green infrastructure proximity and availability applying Europe-wide spatial datasets including high-resolution data on the degree of tree cover and impervious surface density, water areas, and street network data. We spatially analysed proximity of outdoor recreation sites from respondents’ residence and green infrastructure availability near the mapped outdoor recreation sites. Then, we examined statistically significant differences across socio-economic profiles of respondents (age, gender, employment, shift to remote working due to COVID-19, and households with children) and compared the cities. With a focus on the observed differences across the cities and residents’ socio-demographic profiles, our specific objectives are:

1. To examine how visitation of outdoor recreation sites during the COVID-19 pandemic is associated with their proximity to respondents’ places of residence (measured as street network distance);
2. To examine how visitation of outdoor recreation sites during the COVID-19 pandemic is associated with green infrastructure availability at the sites (measured as tree cover and imperviousness density, and as distance to water areas); and
3. To examine whether more frequent visitation of outdoor recreation sites during the COVID-19 pandemic is associated with green infrastructure proximity and availability at the sites.

The results are interpreted against the availability of green infrastructure at the respondents’ residential location that was also analysed for differences between the cities and socio-demographic profiles among respondents. Based on our results, we suggest implications for green infrastructure planning and urban resilience in future crisis situations and in the post-pandemic time.

## Results

### Proximity of outdoor recreation sites to residence

**Fig. 1:**
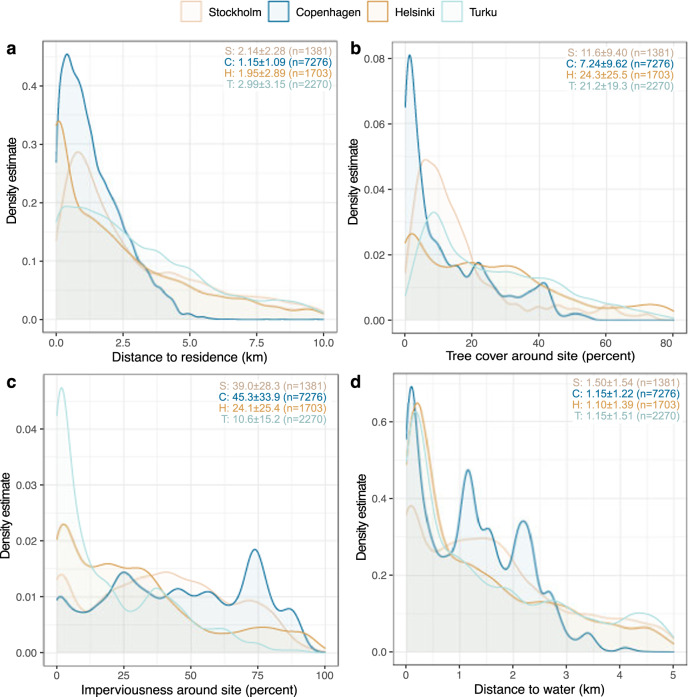
Comparison of outdoor recreation sites across the four case cities. City-wise kernel density estimates (*y*-axes) for a set of recreation site characteristics (*x*-axes): **a** distance to residence, **b** tree cover density, **c** imperviousness density, and **d** distance to water. In the upper right corner, each city’s median ± median absolute deviation (MAD) and number of outdoor recreation sites is shown.

Outdoor recreation sites visited during the COVID-19 are found at the closest proximity to residence in Copenhagen (median ± median absolute deviation (MAD) 1.15 ± 1.09 km), followed by Helsinki (median 1.95 ± 2.89 km) and Stockholm (median 2.14 ± 2.28 km). Turku shows the longest median distance to visited sites (2.99 ± 3.15 km) (Fig. 1a).

**Fig. 2:**
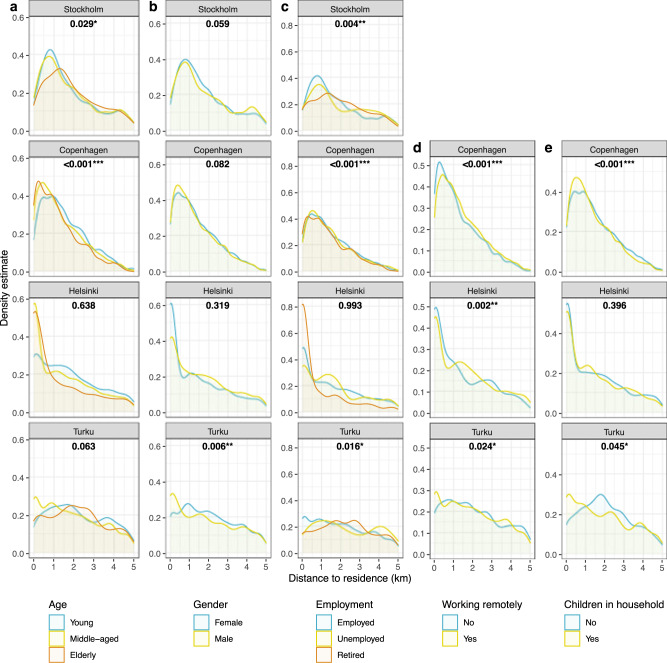
Distance between respondents’ place of residence and outdoor recreation sites. Walking distance in km through the street network is given on *x*-axis and kernel density estimates are given on *y*-axis. Panels show data broken down by city and **a** age, **b** gender, **c** employment, **d** remote working, and **e** children in household. The numbers in each plot correspond to the *p*-value for the Mann–Whitney *U*-test or Kruskal–Wallis test for differences between groups shown in the plot. See also Supplementary Tables 3, 5, 7 and 9 on statistical results as tables.

Comparing age groups within cities, the longest median distance to outdoor recreation sites visited during COVID-19 is observed for the elderly people in Stockholm (median 3.0 km, 0.9–1.0 km higher compared to other groups, *p* = 0.029*) and the young people in Copenhagen (median 1.3 km, 0.2–0.3 km higher, *p* = <0.001***) (Fig. 2a). The difference between genders in distance to outdoor recreation sites is significant only in Turku, where males travelled longer distances compared to females (median 3.8 km, 0.6 km higher, *p* = 0.006**) (Fig. 2b).

Employment shows mixed results. Retired people, compared to other groups, travelled significantly longer distances to sites in Stockholm (median 3.3 km, 1.0–1.2 km higher, *p* = 0.004**) while the data in Copenhagen point slightly in the other direction (median 1.1 km, 0.1 km lower, *p* < 0.001***) (Fig. 2c). In Turku, the three employment groups deviate from each other, and the retired found outdoor recreation sites at the closest proximity (median 3.0 km) and the unemployed at the furthest (median 4.8 km) compared to the employed (median 3.6 km) (*p* = 0.016*) (Fig. 2c). Shifting to remote working due to COVID-19 indicates a statistical significance in the travelled distance to recreation sites in all three cities where data is available (Fig. 2d). Among the Finnish respondents, remote workers visited sites closer to home compared to those who did not experience this change (Helsinki median 2.7 km, 0.7 km lower, *p* = 0.002**; Turku median 3.3 km, 0.4 km lower, *p* = 0.024*). By contrast, in Copenhagen remote workers visited outdoor sites further from home than those who still frequently went to the workplace (median 1.2 km, 0.2 km higher, *p* < 0.001***). For households with children, outdoor recreation sites were found at closer proximity to the residence compared to those without children, observed in Copenhagen (median 1.1 km, 0.2 km lower, *p* < 0.001***) and Turku (median 2.9 km, 0.6 km lower, *p* = 0.045*) (Fig. 2e).

### Green infrastructure availability at outdoor recreation sites

Comparison of the cities shows that at outdoor recreation sites visited during COVID-19, tree cover density is lower and imperviousness density higher in Copenhagen and Stockholm (median±MAD TCD 7.24 ± 9.62/11.6 ± 9.40%; ID 45.3 ± 33.9/39.0 ± 28.3%, respectively) compared to Helsinki and Turku (TCD 24.3 ± 25.5/21.2 ± 19.3%; ID 24.1 ± 25.4/10.6 ± 15.2%, respectively) (Fig. 1b, c). In the Finnish cities, results show similarity in terms of tree cover density, but in Turku imperviousness density is notably lower.

**Fig. 3:**
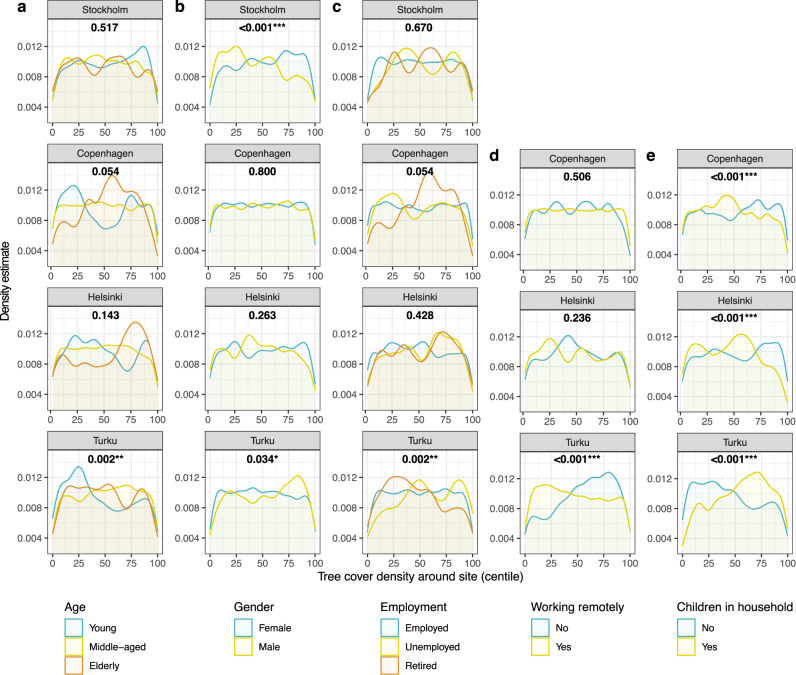
Tree cover density (TCD) within 300m distance around the visited outdoor recreation sites. TCD in centiles of rank order is given on the *x*-axis and kernel density estimates are given on the *y*-axis. Panels show data broken down by city and **a** age, **b** gender, **c** employment, **d** remote working, and **e** children in household. The numbers in each plot correspond to the *p*-value for the Mann–Whitney *U*-test or Kruskal–Wallis test for differences between groups shown in the plot. Rank order centiles are shown to illustrate group differences as estimated by the tests used. See also Supplementary Tables 3, 5, 7 and 9 on statistical results as tables.

**Fig. 4:**
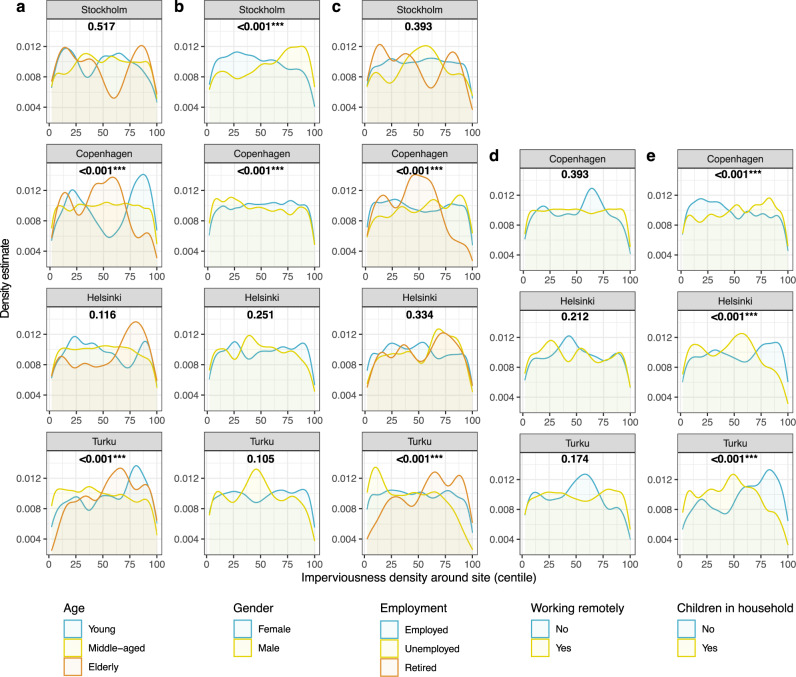
Imperviousness density (ID) within 300m distance around the visited outdoor recreation sites. ID in centiles of rank order is given on the *x*-axis and kernel density estimates are given on the *y*-axis. Panels show data broken down by city and **a** age, **b** gender, **c** employment, **d** remote working, and **e** children in household. The numbers in each plot correspond to the *p*-value for the Mann–Whitney *U*-test or Kruskal–Wallis test for differences between groups shown in the plot. Rank order centiles are shown to illustrate group differences as estimated by the tests used. See also Supplementary Tables 3, 5, 7 and 9 on statistical results as tables.

Age groups display significant differences for both green infrastructure variables in Turku: young people mapped outdoor recreation sites with the lowest tree cover (mean TCD 23.6%, 1.0–2.8% lower, *p* = 0.002**) and the middle-aged differed from other groups by visiting sites with the lowest imperviousness density (mean ID 18.0%, 4.3–4.8% lower, *p* < 0.001***) (Figs. 3a and 4a). In comparison to the middle-aged (ID 45.3%), young people visited sites during COVID-19 with higher imperviousness density (ID 47.9%), and the elderly visited sites with lower imperviousness density (ID 42.2%) (*p* < 0.001***) in Copenhagen (Fig. 4a). Females in Stockholm visited sites with higher tree cover and lower imperviousness density compared to the males (mean TCD 17.5%, 2.2% higher, ID 37.3%, 8.9% lower, *p* < 0.001***) (Figs. 3b and 4b). Compared to females, outdoor recreation by males took place in surroundings with a higher tree cover density in Turku (mean TCD 27.0%, 1.8% higher, *p* = 0.034*) and in lower imperviousness density in Copenhagen (mean ID 43.6%, 2.4% lower, *p* < 0.001***).

In Turku, the unemployed visited outdoor recreation sites with the highest tree cover density (mean TCD 29.1%, 3.5–5.5% higher compared to other groups, *p* = 0.002*) and lowest imperviousness density (mean ID 14.8%, 4.1–8.8% lower, *p* < 0.001***), while retired people did the opposite (Figs. 3c and 4c). Outdoor recreation behaviour of the retired differed from other groups in Copenhagen, and the visited sites display lower imperviousness density (mean ID 41.1%, 3.4–6.5% lower, *p* < 0.001***). For remote working during COVID-19, only in Turku is a significant difference observed where the sites that remote workers visited had lower tree cover density compared to sites visited by other respondents (mean TCD 24.8%, 4.3% lower, *p* < 0.001***) (Fig. 3d).

At the outdoor recreation sites visited during COVID-19, the most prominent group differences are observed when comparing households with children to those without (TCD and ID all *p* < 0.001***). However, the results highlight mixed trends (Figs. 3e and 4e). Respondents with children, compared to those without, visited sites with lower tree cover density in Copenhagen (mean TCD 11.7%, 2.0% lower) and Helsinki (mean TCD 26.5%, 5.2% lower). However, the two cities display divergent results in terms of impervious density. The sites visited by respondents having children, compared to those without, had higher imperviousness in Copenhagen (mean ID 46.6%, 3.2% higher) but lower in Helsinki (mean ID 26.2%, 5.3% lower). Then again, higher tree cover and lower imperviousness density is observed for those sites that respondents with children visited during COVID-19 in Turku (mean TCD27.8%, 4.6% higher, ID 16.6%, 6.6% lower).

Across the four cities, the median distance between the outdoor recreation sites visited during COVID-19, and the closest water element is 1.17 km (Fig. 1d). Outdoor recreation took place closer to water elements in Copenhagen (median ± MAD 1.15 ± 1.22 km), Helsinki (median ± MAD 1.10 ± 1.39 km) and Turku (median ± MAD 1.15 ± 1.51 km) than in Stockholm (median ± MAD 1.50 ± 1.54 km).

Across the cities, age indicates the most common and strong statistically significant (*p* < 0.001***) differences between the groups, except in Turku (Supplementary Fig. 2A). Whereas the sites visited by young people were located at a further distance from water in Helsinki (median 2.1 km, 0.2–0.7 km higher) and Copenhagen (median 1.3 km, 0.2–0.4 km higher), this was instead the case for sites visited by elderly people in Stockholm (median 1.9 km, 1.0–0.9 km higher) (Supplementary Fig. 2A). Differences between genders show sites visited by women were 100 m further from water in Copenhagen (*p* = 0.017*) and 700 m closer to water in Turku (*p* = 0.011*) compared to men (Supplementary Fig. 2B).

Unemployed people differed from those employed and retired by visiting sites at a further distance from water elements in Copenhagen (median 1.3 km, 0.2–0.5 km higher, *p* < 0.001***) (Supplementary Fig. 2C) but closest to them in Stockholm (median 1.0 km, 0.5–0.9 km lower, *p* = 0.009**). Those who shifted to remote working due to COVID-19 visited outdoor sites that were further away from water in Copenhagen (median 1.2 km, 0.2 km higher, *p* < 0.001***) and closer to water in Turku (median 1.1 km, 0.4 km higher, *p* = 0.002**) compared to other respondents (Supplementary Fig. 2D). Households with children visited sites that are further away from water compared to those without children in both Finnish cities (Helsinki median 2.1 km, 0.4 km higher, *p* = 0.017*; Turku median 1.3 km, 0.2 km higher, *p* = 0.017*) (Supplementary Fig. 2E).

### Green infrastructure at sites visited more frequently

**Fig. 5:**
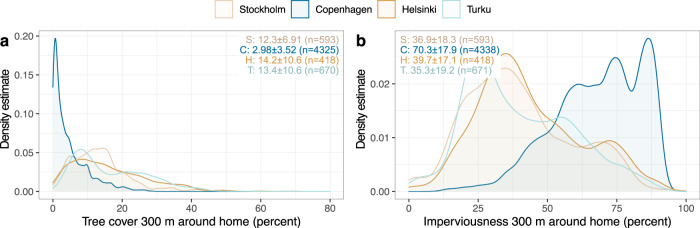
Tree cover density (TCD) and imperviousness density (ID) within 300m around the respondent’s place of residence. *x*-axes show **a** percent tree cover and **b** percent imperviousness surface around the respondent’s place of residence at 300 m distance, while *y*-axis show data point density estimates. The data are grouped by city, with median ± MAD and number of outdoor recreation sites for each city in the upper corners.

The specific segment of data in Copenhagen, Stockholm and Turku that indicates increased frequency of visits to specific outdoor recreation sites during spring 2020 shows that the sites with increased visitation were located at a further distance from residence compared to the whole data of sites particularly in Stockholm (median 2.45 ± 2.41 km, 0.31 km higher) (Supplementary Fig. 3A vs. Fig. 5A). Unemployed people differed from those employed and retired with significantly longer-travelled distances for sites where visits increased in Stockholm (median 5.4 km, 1.8–3.1 km higher, *p* = 0.005**, whole data points to retired visiting sites furthest away) and Turku (median 5.9 km, 3.1 km higher, *p* = 0.004***, the same observed for the whole data) (Supplementary Fig. 3D). Also, a longer-travelled distance to more frequently visited sites is observed for young people compared to other groups in Turku (median 3.7 km, 1.0–1.1 km higher, *p* < 0.001***), whereas the whole data did not indicate significant differences.

**Fig. 6:**
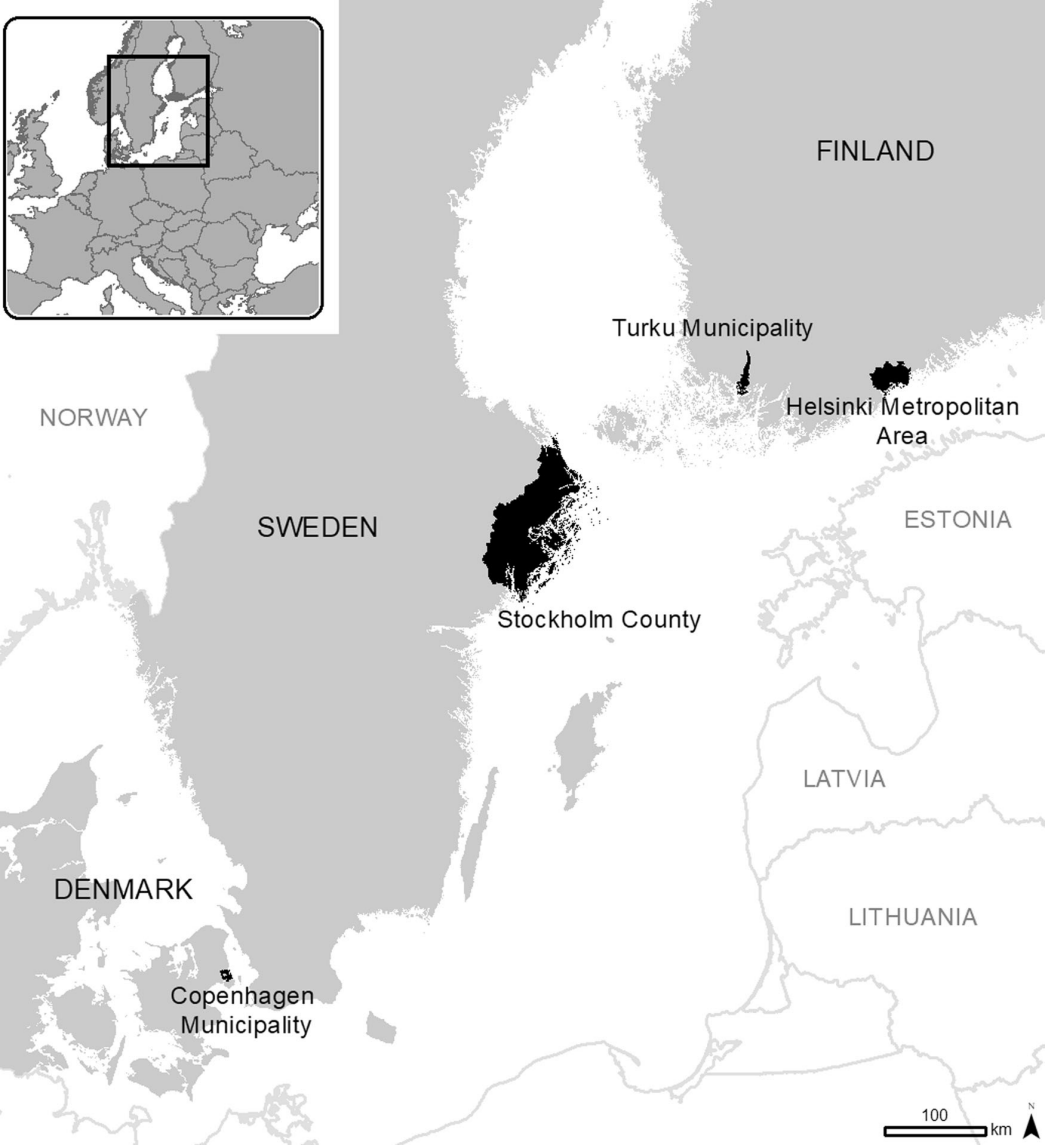
Study areas. The four study areas (black) within the three countries (grey). Inset shows location within Europe.

In terms of green infrastructure availability, people increased recreation where the tree cover density was notably higher and imperviousness density notably lower compared to the whole data (TCD 17.6 ± 14.0% vs. 11.6 ± 9.40%; ID 21.6 ± 26.5% vs. 39.0 ± 28.39%) (Supplementary Figs. 4A and 5A vs. Fig. 6A and 7A). Overall, the statistically significant differences between the cities and the groups of socio-demographic variables (Supplementary Figs. 4 and 5) repeat the general observations made for the whole data (reported in previous section).

### Green infrastructure availability at place of residence

Across the four cities, around the respondents’ place of residence, tree cover density was the lowest (median ± MAD 2.98 ± 3.52% at 300 m) and the imperviousness density the highest (70.3 ± 17.9% at 300 m) in Copenhagen. In comparison, the other cities show notably higher tree cover and lower imperviousness density at residence and are more similar to each other (Stockholm, Helsinki, and Turku: TCD at 300 m 12.3–14.2%; ID at 300 m 35.3–39.7%) (Fig. 5). Overall, few differences are observed between the groups of socio-demographic variables for green infrastructure availability (presented in detail in Supplementary Notes). Young people differed from other groups and lived in the surroundings with the lowest tree cover and the highest imperviousness density. Residence of elderly people was related to the lowest tree cover and highest imperviousness density in Stockholm. Those who shifted to remote working due to COVID-19 lived in surroundings with lower tree cover and higher imperviousness density. Across the four cities, the median distance between place of residence and the closest water element is 1.45 km (Supplementary Fig. 11A), being lower in Copenhagen (median±MAD 1.40 ± 0.90 km) and Turku (1.45 ± 1.35 km) compared to Stockholm (1.78 ± 1.52 km) and Helsinki (1.73 ± 1.51 km).

## Discussion

Unlike earlier work, this cross-city study reveals how the interplay among access to green infrastructure both across cities and at the respondents’ residential location, together with respondents’ socio-demographic profiles and lockdown policies or pandemic restrictions, affects outdoor recreation behaviour. Also, the way people recreated depended on the values residents assigned to specific places. Building on the three spheres of transformation^39^, nature as refuge during a pandemic is conditioned by practical factors, such as the availability of green infrastructure in proximity to one’s domicile and in the wider city structure; political factors, including social distancing regulations; and personal factors, such as values guiding the choice where to recreate. It is therefore critical to assess green infrastructure visitation within specific socio-ecological contexts. Based on our analyses, we identify five main points of results that we discuss below.

1. During the first wave of the COVID-19 pandemic, in the larger cities outdoor recreation took place relatively closer to residence and with less available urban green infrastructure.
  Our results support the evidence that the compact urban structure in the Nordic capitals Copenhagen, Stockholm and Helsinki encourages local activity. Our results show that in the largest cities, Copenhagen and Stockholm, outdoor recreation during COVID-19 in spring 2020 took place in less green and more built-up environments compared to Helsinki and Turku. These results are in line with the share of green infrastructure across the cities^36^. The case-specific analysis in Helsinki shows that respondents were recreating in areas closer to home during the pandemic than before 2018^30^. The smallest city, Turku, offered the greenest outdoor recreation experience during COVID-19. Surprisingly though, residents travelled greater distances to access it compared to the other cities. As Turku residents mostly travelled by foot or bicycle to these sites (>70%, revealed by the attribute data of the sites), the longer-travelled distance could be explained by the relatively well-connected green infrastructure just outside the city centre accessed for example through the National Urban Park^40^.
2. People in Stockholm increased visits to sites offering relatively greener outdoor experiences, influenced by the type of social distancing policies and the availability of green infrastructure to safely recreate.
  The data from Stockholm show that people more often visited sites that offer higher green infrastructure availability (higher TCD and lower ID in more frequently visited sites compared to all mapped sites). Evidence of increased use of green spaces is also confirmed by Legeby et al.^41^. A similar behavioural shift was not observed in Copenhagen nor in Turku (no data for Helsinki). The result may be explained by Stockholm having the most liberal social distancing policies as well as fairly large urban green areas that people could shift to during the pandemic (see Supplementary Methods). Stockholm invested in establishing new nature reserves and the Royal National Urban Park (27 km^2^) during the 1990s and early 2000s and adopted in 2006 comprehensive guidelines for parks and green areas including high accessibility for the residents^42^. The situation was different in the city of Copenhagen where one-way direction of many green paths and a total closure of selected green spaces and playgrounds was enforced in spring 2020. It might be that Copenhagen did not have sufficiently green areas for residents to recreate in while at the same time complying with social distancing regulations. Then again, Turku has overall a greater proportion of green infrastructure among the studied cities, and hence the change was not seen possibly due to this.
3. Low green infrastructure availability at the place of residence seems to encourage people to seek outdoor recreation experiences further away, and vice versa.
  Our results suggest that green infrastructure availability where people live goes hand in hand with how far people travelled for outdoor recreation and indicates the importance of green infrastructure to cope with the crisis. Firstly, young people travelled further than the other age groups for their outdoor recreation during COVID-19 in Copenhagen. They also stand out with increased visits to sites further away in Turku. These results may be explained by the fact that the young people in all cities live in less green and more impervious surroundings, indicating centrally located neighbourhoods. A study from Oslo, Norway, also found that young adults had a large increase in outdoor activity in spring 2020^28^ and evidence from Sweden shows that socialising in nature was particularly emphasised among younger adults^43^. Evidence shows young people were at high risk of suffering from depression and anxiety during COVID-19^33^ and were concerned about their mental well-being, career/studies and economic situation^44^. It may be the case that for young people, nature has offered possibilities for socialisation and coping with the pandemic situation, thus motivating travel for outdoor recreation at longer distances.
  Secondly, interesting observations are made for elderly people. In Stockholm, both elderly and retired people (two groups largely covering the same respondents) travelled furthest away for outdoor recreation in spring 2020 compared to groups of other ages and occupations. In contrast to other cities, Stockholm highlights the most built-up and least green home surroundings for elderly people. Then again, for the retired people in Copenhagen, the trend is the opposite: compared to other respondents, they visited sites closer to home that also have less impervious surface and longer distance to water, and lived in greener and less impervious surroundings closest to water. Explanations for the clear differences may be linked to different housing policies in the Nordic countries. Housing affordability is an issue in all countries, but Sweden stands out with a particularly challenging situation; prices have increased during the past 20 years, and there is a housing shortage for older people with low pensions and modest savings^45^. This may limit the possibilities of older people for choosing to live close to nature or greenery and, hence, motivate for seeking opportunities for pleasant outdoor recreation at longer distances.
  Thirdly, respondents with children targeted their outdoor recreation closer to home in Copenhagen and Turku, and further from water in the Finnish cities. In Turku, families were prone to go outdoors near their residence, even on a daily basis^31^. Respondents having children were also categorised as intensive users of green areas revealed in the site-specific analysis in Helsinki^30^. Across the three cities with available data (Stockholm excluded), respondents with children live in surroundings with high urban green space availability (high TCD and low ID), likely indicating neighbourhoods with less housing density.
4. As an exception, those working remotely due to COVID-19 did not generally seek outdoor recreation experiences in green or blue spaces although living in surroundings with relatively lower availability of green infrastructure.
  The shift to remote working during the COVID-19 spring 2020 happened among those who live in less green and more built-up surroundings compared to those who did not experience this change. Again, this indicates likely more centrally located surroundings. When looking at the outdoor recreation behaviour of those shifting to remote working in terms of visited sites, few significant differences were observed across the cities. Hence, it seems that remote working due to COVID-19 did not encourage people to seek nature recreation experiences in green or blue spaces further away from home, despite the change in daily working routines and possible extra time acquired due to reduced commuting. In addition, in the Finnish cities our results show that the distance travelled from residence to visited sites is shorter among those shifting to remote working compared to those who did not. For more regular breaks during the work days, people working remotely likely use the nearby urban green infrastructure such as private gardens, pocket parks or public spaces such as waterfronts as sites of personal refuge^34,46^ and to mitigate the negative psychological impacts caused by loss of routine^47^, confirmed also in Turku^31^. On the other hand, in Copenhagen the remote workers went slightly further away for outdoor recreation, which could be due to the extensive and advanced biking infrastructure and long-term strategic planning towards creating a *liveable cycling city* that allowed easy, low-cost and safe mobility even during the pandemic^48^.
5. Specific socio-demographic groups sought outdoor recreation experiences during COVID-19 at a notable distance from their residence (young in Copenhagen; elderly and unemployed in Stockholm; young and unemployed in Turku).
  In addition to the young, elderly and retired people in specific cities travelling the longest distances for outdoor recreation, as discussed above, similar observations were also made for unemployed respondents. In Stockholm and Turku, the unemployed increased visitation at sites located further away compared to the other groups. In addition, among the unemployed in Turku, outdoor recreation behaviour differed from other groups’ behaviour by targeting sites offering greener and less built-up experiences, located at a significantly longer distance from residence. The site-specific study from Stockholm showed that the further a visited site was from the respondent’s residence, the more likely it was to have a positive well-being influence^29^. Indeed, when most activities were restricted due to COVID-19, spending time exploring seldom-visited or even new outdoor settings, such as the forests and national parks near urban areas^26^, could be a coping strategy to escape the stress caused by COVID-19^22^. This opportunity was presumably more available for those not occupied by everyday work. Interestingly, our result contrasts with a national survey from England, showing those not working (including unemployed, long-term sick/disabled, looking after children/house/other caring responsibilities, and retired) were less likely to visit natural spaces^23^.

Our results indicate some implications for future green infrastructure planning. We see a clear signal that the heritage of Nordic spatial planning, where availability of green-blue infrastructure has traditionally been an important part of urban development^35,49^, supports urban resilience, not just in normal times but also during the current pandemic^19^. Nordic capitals encouraged more local outdoor recreation behaviour compared to the provincial city of Turku, which can be attributed to the fact that Nordic capitals have a compact urban structure that can promote sustainable lifestyles also in the wider sense^50,51^. In the light of our results, particularly Stockholm seemed to support pandemic resilience. However, the question remains how this will change in the future, as populations are expected to increase, most heavily in Stockholm by 60% and in Copenhagen by 35% between 2015 and 2050^52^, leading to fewer square metres of green infrastructure per person and for the promotion of ecological functions. This has led to a stressing of the quality, accessibility and connectivity over quantity of green infrastructure and, for example in Stockholm, raised concerns over ‘parkification’ of green infrastructure^53^. In Helsinki, where the main strategic target in urban planning is densification^54^, the loss of green spaces due to densification has already created tensions between various groups of inhabitants and urban planners. To ease these tensions, the city promotes ambitious public participation processes that have been realised, for example, through a large-scale participatory mapping survey^55^. Carefully considering the spatial pattern of the expected population growth versus local supply of green space is important. As an example, the past development in Copenhagen reveals simultaneous population growth and increasing greenness (measured as normalised difference vegetation index) but also several hot spots with population growth and stable or declining greenness^56^. Warming climate is the main driver of observed greening (idib.) but urban planning policies that seek to preserve and increase nature in densifying cities^57^ are the important enablers of resilience.

A transformative change is currently happening due to COVID-19 with a new normal in which the remote working culture will partly remain for some job sectors^58^. Reduced pollution levels due to decreased commuting may increase people’s well-being^59^, but the change puts pressure on the development of built-up urban areas, where the workers in these sectors specifically reside, as indicated by our results. The need for living environments with easy access to resilient outdoor spaces offering green-blue experiences increases, particularly for those without private green spaces at their residence^25^. For families with children, our results highlight the need for places designed for outdoor activities and natural places close to residential areas, such as hybrids of playgrounds and small patches of forests or ‘pocket forests’^60^. Also, access for children to green court yards or green school yards for informal after-school activities might be highly relevant to consider as a green resource as shown in Sweden and Denmark^61^. Hence, in light of these groups, urban development should confront the risk of losing small-scale green infrastructure that can offer important sites for personal refuge, not only during times of crisis but also in daily life in general^34^. Interesting evidence of the importance of a rather new type of public urban green infrastructure comes from Copenhagen where lush and green cemeteries having recreational zones, strategically promoted as recreational spaces^62^, were increasingly used local public green spaces in the dense urban structure during the pandemic^63^. Similar observations were made in Stockholm^41^.

Small-scale green infrastructure is also beneficial from the perspective of affordable housing, as large development projects easily cause rising property values and subsequent replacement of residents with low incomes^8,64^. The pandemic has increased the demand for detached and single-family houses^65^ that indicates urban sprawl and further challenges the availability of affordable housing in the Nordic cities^45^. This should be addressed by promoting small-scale green infrastructure in dense urban areas that are ‘just green enough’ and explicitly protect social as well as ecological sustainability^66,67^. The argumentation can be extended to the crucial sustainability aspect of having enough density to support local grocery stores, shops, health care and other services that Legeby et al.^41^ showed, in addition to urban green infrastructure, to be crucial for pandemic resilience. Supporting everyday activities and needs locally supports also active and sustainable mobility in general.

Furthermore, it can be expected that the green wedges penetrating the cities and the large nature areas both in and near cities have specific importance for some socio-demographic groups of people with less structured time in everyday life (the young, elderly, unemployed). It is crucial to protect these areas from urban encroachment, focus on outdoor recreational infrastructure development^68^, and support easy access by environmentally friendly means of public transport and biking infrastructure development. Negative effects of public transportation use during a pandemic could be diminished by ‘responsible transport’ measures^69^. These measures aim to make individual citizens aware of the effects their mobility and travel behaviour have on themselves, other people, and the environment and empower them to act accordingly.

In conclusion, in the current green infrastructure development, both the green wedges penetrating the suburbs and the nature areas close to core cities need to be preserved. However, attention should also be placed on preservation and establishment of small-scale green infrastructure. These two aspects should be priority aims of local city authorities developing the Urban Greening Plans per the EU Biodiversity Strategy^38^ and should be acknowledged in spatial planning. At the same time, these targets should be balanced with adequate access to local services dependent on a certain population density. This type of urban planning would support resilience and well-being specifically during health crises such as the current pandemic and in general in the post-COVID-19 future.

Finally, we raise some methodological considerations for future research. Integrating data collected through different surveys, though with a similar participatory mapping approach and digital survey platform, required some compromises. The focus of this article is on individuals and the patterns their outdoor recreation behaviour forms in relation to specific places, but a comparative analysis of the descriptive data related to mapped sites (e.g., reporting the frequency of visits, actual time spent on a site, or the actual use of or values related to these sites) was restricted by the heterogeneity of questions posed in each survey. It should be acknowledged that the applied spatial approach cannot uncover the perceived quality of the green infrastructure, which can have crucial effects on its use, for example, if considered unsafe^70^. With the current data, we cannot infer how outdoor recreation behaviour changed from the pre-pandemic to the actual pandemic situation, which would be interesting to analyse in order to create in-depth understanding of coping with a crisis^30^. Also, it would have been interesting to analyse respondents’ economic status (not available across our datasets) or outdoor recreation behaviour of vulnerable and minority groups disproportionately impacted by COVID-19^71^ in order to contribute to discussions through the environmental justice lens^72^. The Turku survey was available in six languages and widely promoted, but only a few responses in the less-spoken languages were captured.

Our data show overrepresentation of women and deviations in age and employment groups across cities. Women seem to respond more often to studies on green areas^73,74^. Also, the pandemic has likely increased burdens on women^75^, who may have had a greater need for stress relief and have potentially turned to nature^22^. These issues support the higher response rates of women. In terms of geographical representation, data were collected in five districts in the city centre of Copenhagen. This can affect some of the observed differences between the cities (e.g., that the respondents in Copenhagen live in less green and more built-up surroundings than in other cities). Respondent recruitment strategies influence data quality and the underlying respondent effort. Following Brown^76^, we expect that the data quality is highest for the random sampling (Helsinki), followed by convenience samples (Stockholm, Turku) and panels (Copenhagen).

## Methods

### Study areas

This study is performed across four cities in three countries: Copenhagen (five districts), Helsinki Metropolitan Area (hereafter, Helsinki), Stockholm County (hereafter, Stockholm) and Turku (Fig. 6). The cities differ in the number of inhabitants, with Turku being the smallest (192,962) and Stockholm the largest (2,377,081) (Supplementary Table 1). Population densities in all cities are lower and the share of green infrastructure higher compared to the average in the European Union (EU)^36,37^. However, the share of green urban areas in the core city is lower in Copenhagen compared to the other cities (22.2% vs. 53.0–75.4%, Supplementary Table 1). Please refer to Supplementary Methods for the detailed description of green infrastructure across cities, its roots in spatial planning in the Nordic context, and for the restrictions introduced due to the COVID-19 pandemic in spring 2020.

### Survey data

In this article, we bring together separately administered survey datasets collected during May–June 2020 in the four cities. Each case has been reported separately: Copenhagen by Præstholm et al.^77^, Stockholm by Samuelsson et al.^29^, Helsinki by Korpilo et al.^30^, and Turku by Fagerholm et al.^31^. We collected all data applying a participatory mapping approach using online map-based surveys targeted to residents older than 15 (Table 1). Participatory mapping (public participation GIS, PPGIS) offers the possibility to study the behaviour of individuals and perceptions behind their behaviour in a place-based way^78^. As a spatial approach, it gives an experience-based perspective on outdoor recreation behaviour compared to, for example, large-scale mobility patterns observed through passive sensing technologies such as Google tracking or mobile phone data^79,80^. PPGIS has been successfully applied in order to understand the everyday experiences of urban dwellers and their perceptions of green infrastructure (e.g., refs. ^73,81^).

**Table 1:** Characteristics of map-based surveys applied during COVID-19 spring 2020.

City	Copenhagen	Stockholm	Helsinki	Turku
Data collection time	15.5–15.7 2020 (9 weeks)	27.4–15.6.2020 (7 weeks)	7.5.–31.5.2020 (4 weeks)	11.5.–21.6.2020 (6 weeks)
Sampling approach	Five local citizen panels relating to local district councils (volunteered recruitment)	Convenience sampling	A simple random sample of 10,000 inhabitants	Convenience sampling
Targeted respondents	Inhabitants above 19 in the five local districts	People 15 or older with reading competence in Swedish	Working-age adults (18–65 years) living permanently in the study area (Helsinki metropolitan area consisting of the municipalities of Helsinki, Espoo, Vantaa, and Kauniainen)	Turku inhabitants above 15 years old
Survey respondents total/% of population	4992/1.8% (of local district population) Avg. response rate across panels = 27%	593/0.02%	469/0.04%	730/0.4%

All surveys were operated on the Maptionnaire platform. In the Copenhagen, Helsinki and Turku surveys, we asked respondents to locate their outdoor recreation sites during the spring of 2020. The point marker instructions in the surveys included in Copenhagen and Turku ‘Here I spent time outdoors’ and in Helsinki ‘Place for leisure time activity’ (see Supplementary Methods). In the Stockholm survey, we asked the respondent to mark as a point either ‘a site I visit less or have avoided’, ‘a site I continue to visit with a similar frequency’ or ‘a site I have visited more’ in recent weeks compared to the time before COVID-19. From the Stockholm data, we used the outdoor recreation sites visited with similar frequency or more. In all surveys, after mapping an outdoor recreation site, subsequent questions addressed further details of the sites (not treated in this article, except the indication whether the site was visited more frequently due to COVID-19 in Copenhagen and Turku). In all surveys, we asked respondents to map the location of their residence (as point marker) and to respond to various socio-demographic and other respondent-related questions (see Supplementary Methods for individual survey contents).

In Copenhagen, we distributed the survey through five local citizen panels relating to the local district councils. In Stockholm, respondents were targeted through convenience sampling by sharing the survey in press releases from the involved universities and the urban planning department of the city of Stockholm. Hence, the majority of respondents came from the city of Stockholm. In Turku, convenience sampling was similarly used by sharing the survey in press releases, social media channels of the city of Turku, several local social and print media channels, and e-mail lists of local associations. In Helsinki, a simple random sample of 10,000 working-age (18–65 years) adults living permanently in the study area were recruited to a PPGIS survey in fall 2018. A mail invitation to answer a follow-up survey in spring 2020 was sent to the 1512 respondents of the previous survey.

In all surveys, participation was voluntary. Participants could withdraw in the middle of the survey if they preferred to. The universities of Copenhagen, Gävle, Aalto and Turku human research ethics committees did not require a full ethics application to be submitted because the studies were deemed low risk; that is, in all surveys participants were above 15 years old and prior written informed consent was obtained in the survey platform.

The surveys reached between 469 and 4992 respondents, which corresponds to 0.02–1.8% of each city’s respective population (Table 1). Comparison to population (Table 2) shows that young respondents aged up to 29 years were underrepresented in all study sites but Stockholm. Older adult and elderly respondents were overrepresented in Helsinki and Copenhagen and underrepresented in Turku and Stockholm. Women were overrepresented across all sites. Group employed/student were overrepresented in the data collected in Turku and Stockholm.

**Table 2:** Socio-demographic characteristics in sampled data with comparison to the population in the study area.

	Copenhagen		Stockholm		Helsinki		Turku	
	Sample (%)	Study population (%)^a^	Sample (%)	Study population (%)^b^	Sample (%)	Study population (%)^c^	Sample (%)	Study population (%)^d^
Age group^e^	*n* = 4299		*n* = 593		*n* = 420		*n* = 717	
Young (15–29)	11.5	32.9	29.0	18.3	13.1	22.5	19.9	28.3
Middle-aged (30–64)	73.5	55.4	62.2	39.0	76.2	73.1	70.0	48.0
Older adults/elderly (65+)	14.9	11.8	8.8	16.0	10.7	4.3	10.0	23.8
Gender	*n* = 4302		*n* = 593		*n* = 417		*n* = 713	
Male	39.2	49.0	33.6	50.1	43.4	49.6	27.1	47.8
Female	60.8	51.0	66.4	49.9	56.6	50.4	72.9	52.2
Employment	*n* = 2686		*n* = 593		*n* = 345		*n* = 568	
Employed/student	80.4	83.1	88.2	80.3	82.3	81.9	80.6	60.7
Unemployed	5.5	4.2	3.0	4.9	5.7	6.9	5.5	6.6
Retired	14.1	12.7	8.8	12.3	8.5	4.4	13.9	28.4

^a^Residents age/gender group 18+ years from Statistic Copenhagen (2020), Educational level/Occupation situation 16+ years from Statistic Denmark (2018).

^b^Residents aged 15+ years. Occupation age groups 16+ years. Source: Statistics Sweden (2021).

^c^Residents aged 18 to 65 years. Educational level age groups 20 to 64 years, occupation age groups 18 to 64 years. Source: Statistics Finland, 2019 (occupation, educational level) and 2020 (gender, age group).

^d^Residents aged 15+ years. Occupation age groups 18+ years. Source: Statistics Finland, 2018 (educational level) and 2019 (occupation, gender, age group).

^e^In the Stockholm survey, people aged 15–34 were classified as young and those aged 35–64 as middle-aged. In the Helsinki survey, age was calculated at the time of the follow-up survey and compared to the sample population aged 20 to 67 years.

Sources: Statistics Finland, Statistic Copenhagen, Statistic Denmark, Statistics Sweden.

### Analysis

To analyse the proximity of urban green infrastructure, we developed a customised tool to calculate the distance between each outdoor recreation site (point) and 1) the respondent’s residence (point) and 2) the boundary of the closest water body (polygon). The analysis uses HERE street network data from ArcGIS Online^82^ and applies a snapping distance of 1000 m in order to reach the nearest available network segment for all mapped sites. We extracted the land-use class ‘water’, from Urban Atlas 2018, offered by the Copernicus Land Monitoring Service (https://land.copernicus.eu/) by the European Environment Agency, and used it to describe the coverage of water bodies, including the sea, rivers, and lakes (minimum mapping width 10 m).

To analyse the availability of green infrastructure at the outdoor recreation sites, we downloaded from the Copernicus Land Monitoring Service 2018 spatial data of the high-resolution layers of the tree cover density (TCD, derived from Sentinel-2A + B time series) and imperviousness density (ID). The datasets, covering all of Europe, describe the degree of tree cover and sealed (hard) surface, respectively, ranging from 0–100% for each 10 m cell. Hence, the data give an indication of the amount of green space based on trees and indirectly by indicating the share of unbuilt surface. The mean tree cover and imperviousness density were calculated within a 300 m buffer around each mapped outdoor recreation site. A radius of 300 m was applied to represent the local scale of a site.

In addition, to interpret the outdoor recreation during COVID-19 in the context of green infrastructure available at respondent’s residential location, we calculated the mean tree cover and imperviousness density within a buffer of 300 m around each respondents’ place of residence. In urban areas, living less than 300 m from an area suitable for recreation is commonly considered as a threshold distance for their good accessibility^14,36^. However, it has been suggested that the larger community or neighbourhood scale also matters for experiencing benefits from nature^83^. Therefore, we performed a sensitivity analysis of the green infrastructure availability at the place of residence by repeating the analysis with a 1000 m buffer (see Appendix 4). Furthermore, we calculated distance between residence and closest water body in an identical way as described above. We performed the geospatial analyses in ArcGISPro and QGIS.

Once the geospatial analyses were performed, we proceeded with statistical analysis and visualisation of all outdoor recreation sites (research objectives 1 and 2) and sites with more frequent visitation (research objective 3; Copenhagen, Stockholm and Turku only). In addition, the residential locations were analysed separately.

Firstly, we calculated median and median absolute deviation (MAD) values of tree cover and impervious density around the mapped sites and place of residence, and for the proximity analysis results. Median and MAD statistics were used because they are robust to outliers, which were most notably present in terms of tree cover density and distance from home.

Next, we analysed differences between groups for the variables of gender (male; female), age (young: 15–29; middle-aged: 30–64; elderly: 65+), employment (employed, incl. student; unemployed; retired), shift to remote working due to COVID-19 (yes; no), and households with children (yes; no). Data on remote working and households with children were not available for Stockholm. Statistically significant differences between the groups of each socio-demographic variable were tested with a nonparametric Mann–Whitney *U*-test and an independent samples Kruskal–Wallis test, as the tree cover density, imperviousness density, and distance variables did not show a normal distribution between the groups. The statistical analyses were done in SPSS and R.

We visualised the group differences between cities and socio-demographic groups with kernel density plots, which are used for estimating probability density functions. A helpful way to think about density plots is as smoothed histograms. The smoothing facilitates group comparison as compared to regular histograms. The estimated density function depends on the kernel used. The kernel has a range (or ‘smoothing window’) over which observed data around any given value influences the estimated probability of that value. It also has a function for weighting observations within the smoothing window. We used a Gaussian (‘bell curve’) kernel function, meaning that the influence of observations on the estimated probability of any value decays with distance from it up until the limits of the smoothing window. We used the ggplot2 package^84^ in R to produce the density plots, and hence followed Silverman’s rule of thumb^85^ as implemented in ggplot2 for choosing kernel width. After an iteration of visual inspection of the plots, we adjusted the kernel width to 75% of the original value using the adjust function.

## Supplementary information

Supplementary Information

## Data Availability

Copenhagen: The data (without residential locations) are available at Zenodo.org at: 10.5281/zenodo.5782954. Stockholm: The data are not published openly due to lacking permission from the respondents. The data is available for reviewers upon request. Helsinki: The data (without residential locations) are available at Zenodo.org at: 10.5281/zenodo.5789047. Turku: The data (without residential locations) are available at the University of Turku Geospatial Data Service at: https://geonode.utu.fi/layers/geonode:Places. Data on tree cover density and imperviousness density are publicly available data with free accesses at the Copernicus Land Monitoring Service (https://land.copernicus.eu/) provided by the European Environment Agency.

## Code availability

The code used to produce the results of distance analysis is available from the authors upon request.
