# Supplementary material for: Analysis of pandemic outdoor recreation and green infrastructure in Nordic cities to enhance urban resilience
Source: NPJ Urban Sustain. 2022 Oct 3;2(1):25. doi: 10.1038/s42949-022-00068-8 (PMC9529166; doi:10.1038/s42949-022-00068-8)
Supplement: Supplementary file 1 — Supplementary Information [file 42949_2022_68_MOESM1_ESM.pdf]

Supplementary information

Fagerholm et al. Analysis of pandemic outdoor recreation and green infrastructure in Nordic cities to enhance urban resilience

Supplementary Methods

Green infrastructure and spatial planning

The four cities entail diverse green-blue infrastructure (Supplementary Table 1). All the study areas have a few larger maintained urban parks, numerous pocket parks and sports areas as well as communal and private gardens. In Helsinki and Turku, there are also small and medium-sized semi-natural forests remaining among the residential areas surrounding the city centre. In all the cities there are also larger green-blue areas and nature reserves bordering the urban core: in Copenhagen, a wetland system of Utterslev Mose, Amager Nature Park and Amager Beach Park; in Stockholm, 10 nature reserves as well as several protected shorelines; in Helsinki, National Parks of Nuuksio and Sipoonkorpi; and in Turku, altogether 18 conservation areas, including Ruissalo island. Uniquely, both Copenhagen and Stockholm have firmly protected green wedges that make their way to the very centre of the city and connect the urban green infrastructure to the larger ecosystems surrounding the city, crossing several municipalities[1]. Access to both public and private forests and other natural spaces is in general allowed through the 'everyman' right of access in Sweden and Finland, while access in Denmark is allowed only on roads and trails in private forests[2]. Furthermore, all the study areas are located on the Baltic Sea coast, with varying public access to the seafront as well as recreation and residential areas on islands. In addition, the blue infrastructure includes, for example, small lakes, canals in Copenhagen, and a river that runs through the city centre of Turku.

What is seen today across the four cities has roots in the history of spatial planning of Nordic cities that has traditionally stressed the integration of blue–green infrastructures in urban environments, reflecting the values of the Nordic welfare state including equality, closeness to nature, collectivism and community building[3,4]. In the urbanization processes during the decades after World War II (1940s–50s) and during the following decades of economic growth (1960s–70s), preservation of nature, particularly forests surrounding cities, has been important for recreational purposes[5]. In Denmark, forests in particular have been legally protected for two centuries, which has led to afforestation even in the fringes of large cities[6]. Nowadays the role of urban green infrastructure is acknowledged as important for biodiversity conservation across the EU and has become prominent in spatial planning over the last few decades[7]. The European Commission has, for example, launched urban green infrastructure as a strategic focus in Europe with ambitious targets for cities[8].

Supplementary Table 1. Study area characteristics.

City (country)	Full study area name	Number of municipalities	Population[1]	Case area size (km2)	Pop density (pop/km2)	Share of green areas inside core city (% of total area)[2]	Description of green infrastructure
Copenhagen (DK)	Municipality of Copenhagen: five city districts<br><br>(Brønshøj-Husum, Bispebjerg, Nørrebro, Amager-Vest and Amager-Øst)	1	296,554 in the five districts (633,449 in the municipality of Copenhagen, 2,052,348 in the Metropolitan Region)	48.9	6064	22.2	In the Metropolitan area of Copenhagen, the Copenhagen Finger Plan of green wedges maintains ecological corridors alongside the development of urban structures in the city including the Copenhagen Municipality. The five districts where the PPGIS survey was carried out are together forming a North-South transect of Copenhagen Municipality, bordering some of the city's biggest green areas and nature reserves, including a wetland system to the north (Utterslev Mose), Amager Nature Park to the southwest and Amager Beach Park to the southeast. Further, the blue-green infrastructure is characterized by a few bigger urban parks, pocket parks, cemeteries open for recreational use, green courtyards, areas for sports and private gardens, as well as a series of lakes, canals and a seafront.

Stockholm (SE)	Stockholm County	26	2,377,081	7193.9	330	56.4	The green infrastructure of Stockholm is characterized by firmly protected green wedges that make their way to the very centre of the city. These nature reserves are connected to the larger ecosystems surrounding the city and cross several municipalities. Apart from acting as wildlife corridors, they are also important recreational areas for Stockholmers. Altogether one-third of Stockholm's green spaces and water areas are protected, consisting of ten nature reserves, as well as many beautiful protected shorelines. Forestlands cover 20 % of Stockholm's area.
Helsinki (FI)	Helsinki Metropolitan Area	4	1,187,138	814.5	1458	53.0	In the Helsinki region, the degree of urbanization varies greatly, and thus areas differ by the type and neighbourhood availability of green infrastructure. There are smaller, maintained green spaces in the city centre and small to medium-sized maintained green spaces throughout the urbanized parts of the region. Small and medium-sized forests and other natural environments are well accessible throughout the region. Large nature areas and national parks (Nuuksio, Sipoonkorpi) are accessible in the borders of the study area. The seaside in the study area is extensive and mostly open for public access.

Turku (FI)	Turku municipality	1	192,962	249.4	774	75.4	In the city centre of Turku, there are several small urban parks as well as a recreational area along the Aura River that runs through the centre. Similarly, as in many cities in Finland, residential areas further away from the centre are surrounded by semi-natural urban forests and green areas that have been spared from urban infill. Hence, there are several larger forests within 4–6 km radius from the centre in different neighbourhoods. Furthermore, the city has 18 conservation areas (13.9 km² Natura areas, 5.7 % of land area) that include, for example, protected wetlands, old oak forests and marshland. The city, located on the southwestern coast of Finland, also has several islands within its proximity, some of them accessible by public transport.

[1]Sources: Statistics Finland, Statistic Copenhagen, Statistic Denmark, Statistics Sweden.
[2]Source: The European Data Journalism Network, 2019. https://www.europeandatajournalism.eu/Tools-for-journalists/Useful-data/Green-infrastructure-in-Europe

Restrictions introduced due to COVID-19 in spring 2020

National governments have met the spread of COVID-19 with a variety of regulations, both in terms of guidelines and stricter policies. In spring 2020, these regulations included new investments in healthcare facilities, contact tracing and welfare provision to increase the healthcare capacity, but mostly aimed to rapidly mitigate the spread of the epidemic. Hence, these measures included restrictions on travelling, public gatherings, and social events to reduce the number of contacts between people. Cross-national comparison using the COVID-19 Stringency Index (a composite measure of response indicators such as school closures, workplace closures, cancellation of public events, restrictions on public gatherings, closures of public transport, stay-at-home requirements, public information campaigns, restrictions on internal movements, and international travel controls) shows that in the first half of 2020[9], restrictions across Denmark, Sweden, and Finland were similar (Supplementary Fig. 1A). Also, stay-at-home requirements were only recommended (Supplementary Fig. 1A). It was mostly citizens' responsibility to reduce social contact and respect social distancing. During spring 2020, Sweden's response to the pandemic largely took the form of guidelines revolving around individual responsibility[10], and was frequently described as 'soft-touch' by international news media. The Stringency Index shows that Sweden was later than Denmark or Finland to introduce restrictions but does not show any clear difference when the restrictions were at the highest level in April–May 2020 (Supplementary Fig. 1A).

In April–May 2020, in Denmark and Finland schools and universities were closed and shifted to distance education, social distancing and remote work was recommended, restaurants were closed or services limited, indoor cultural and sports facilities were closed, and public gatherings of more than 10 people were banned[11,12]. Kindergartens were closed in Denmark, whereas in Finland parents of preschool children were recommended to keep their children at home. In Denmark specific regulations were set for outdoor activities, including travelling in one direction on green paths and closing selected green spaces and playgrounds. In Finland, movement in and out of the entire Helsinki capital region was limited to the most essential travel from late March until mid-April. In Sweden, secondary schools were closed, social distancing and remote working was recommended, spacing in restaurants and bars was regulated, and public gatherings were limited to 50 people[13]. Gradually, starting in late May, these restrictions were relaxed in all the three countries.

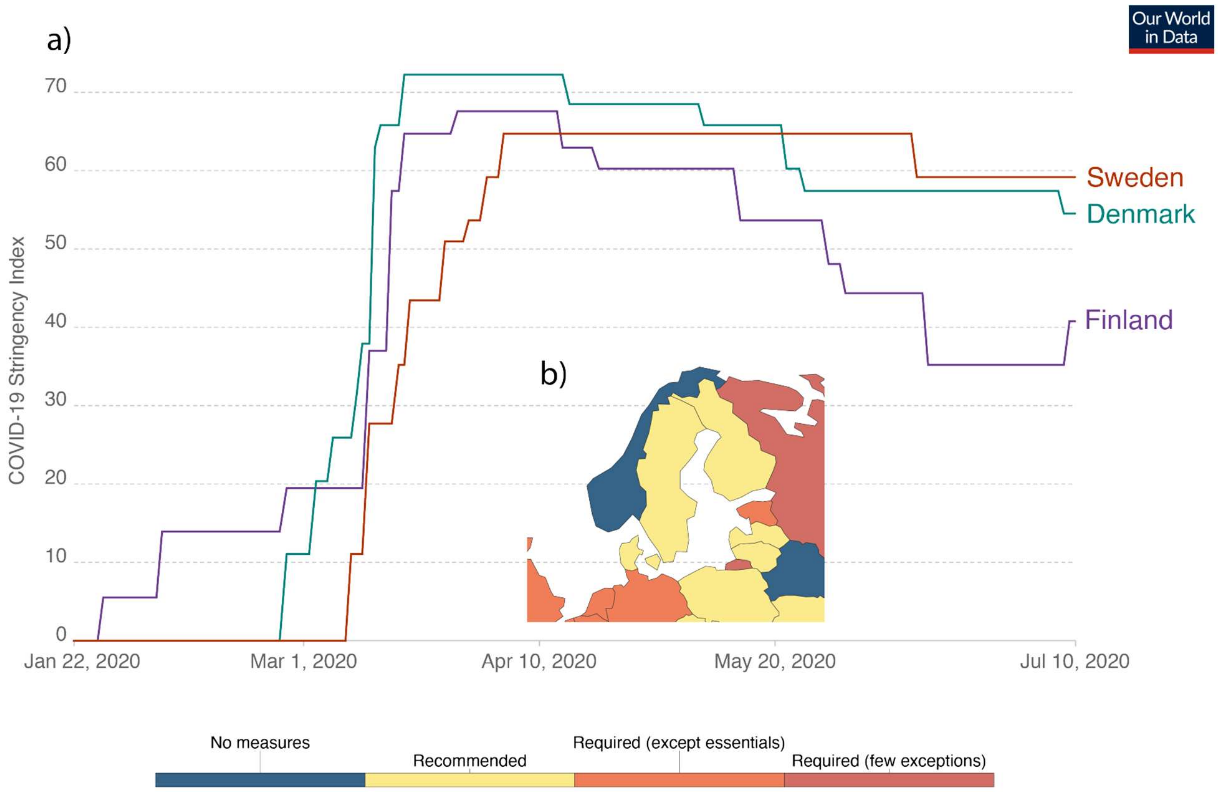

Supplementary Figure 1. Restrictions introduced to reduce the spread of COVID-19. (a) COVID-19 Stringency Index: This is a composite measure based on nine response indicators including school closures, workplace closures, and travel bans, rescaled to a value from 0 to 100 (100 = strictest). If policies vary at the subnational level, the index is shown as the response of the level of the strictest sub-region. (b) Stay-at-home requirements during the COVID-19 pandemic as for May 1, 2020. Data from Hale, T. et al. A global panel database of pandemic policies (Oxford COVID-19 Government Response Tracker). Nat. Hum. Behav. 5, 529–538 (2021) and Oxford COVID-19 Government Response Tracker, Blavatnik School of Government, University of Oxford (2021) via Our World in Data (Ritchie, H., Mathieu, E., Rodés-Guirao, L., Appel, C., Giattino, C., Ortiz-Ospina, E., Hasell, J., Macdonald, B., Beltekian, D., & Roser, M. Coronavirus Pandemic (COVID-19). https://ourworldindata.org/coronavirus (2020)).

Survey contents

Contents of four surveys as screen shots:

Urban Environment and Health -survey, follow-up during the COVID-19 pandemic (Helsinki)

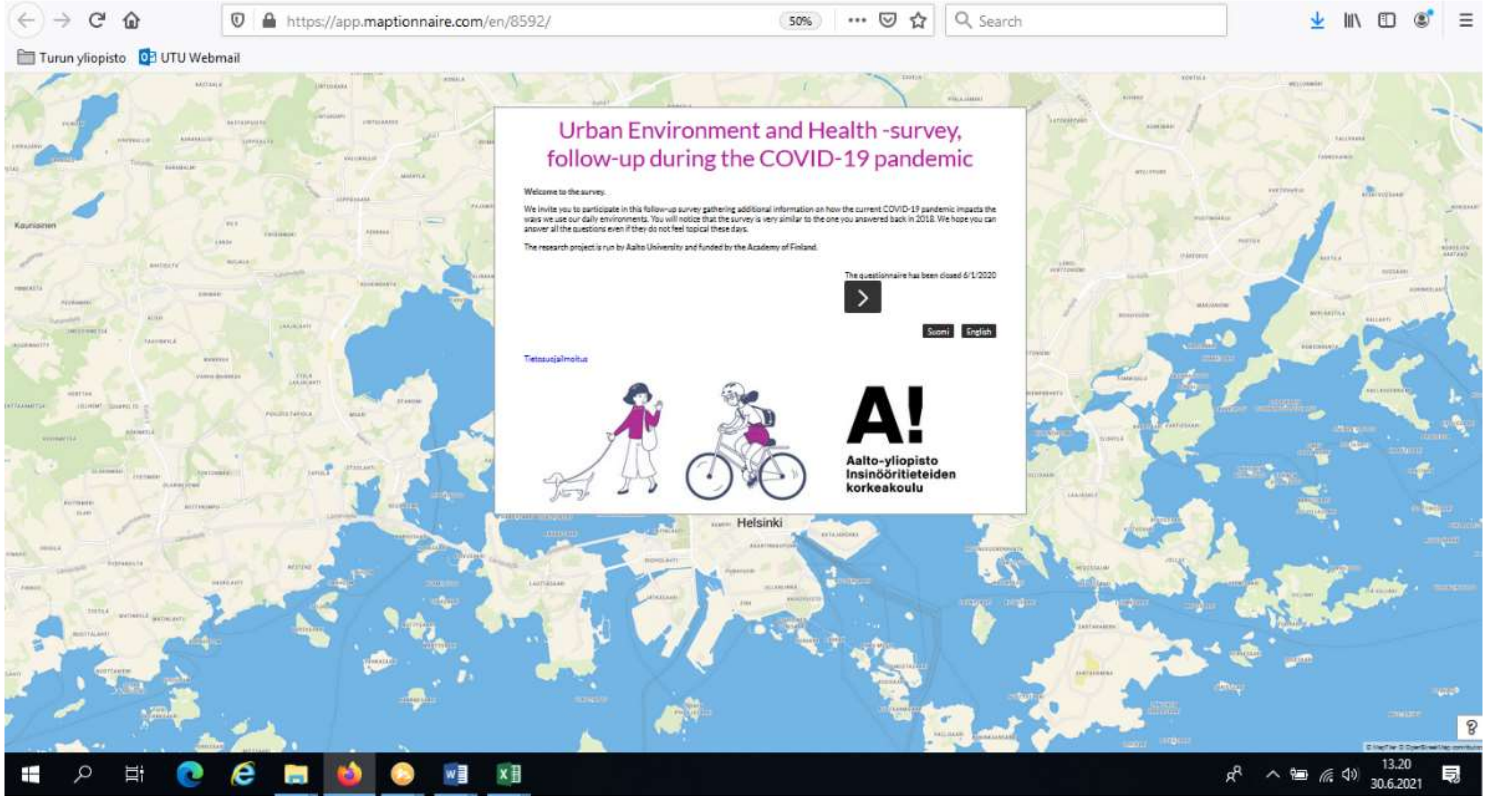

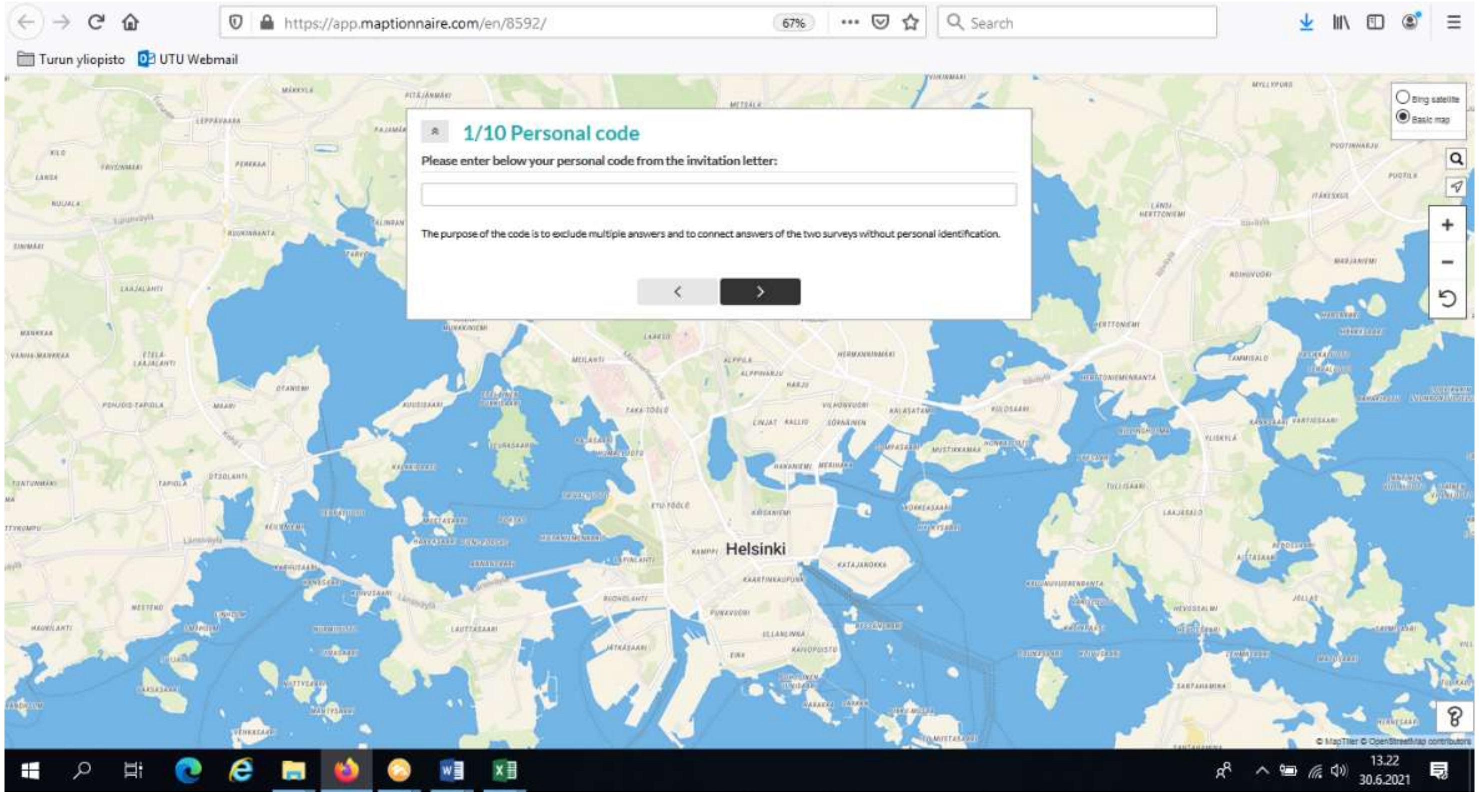
https://app.maptionnaire.com/en/8592/
67%
Search
Turun yliopisto
UTU Webmail
1/10 Personal code
Please enter below your personal code from the invitation letter:
The purpose of the code is to exclude multiple answers and to connect answers of the two surveys without personal identification.
Helsinki
13.22
30.6.2021

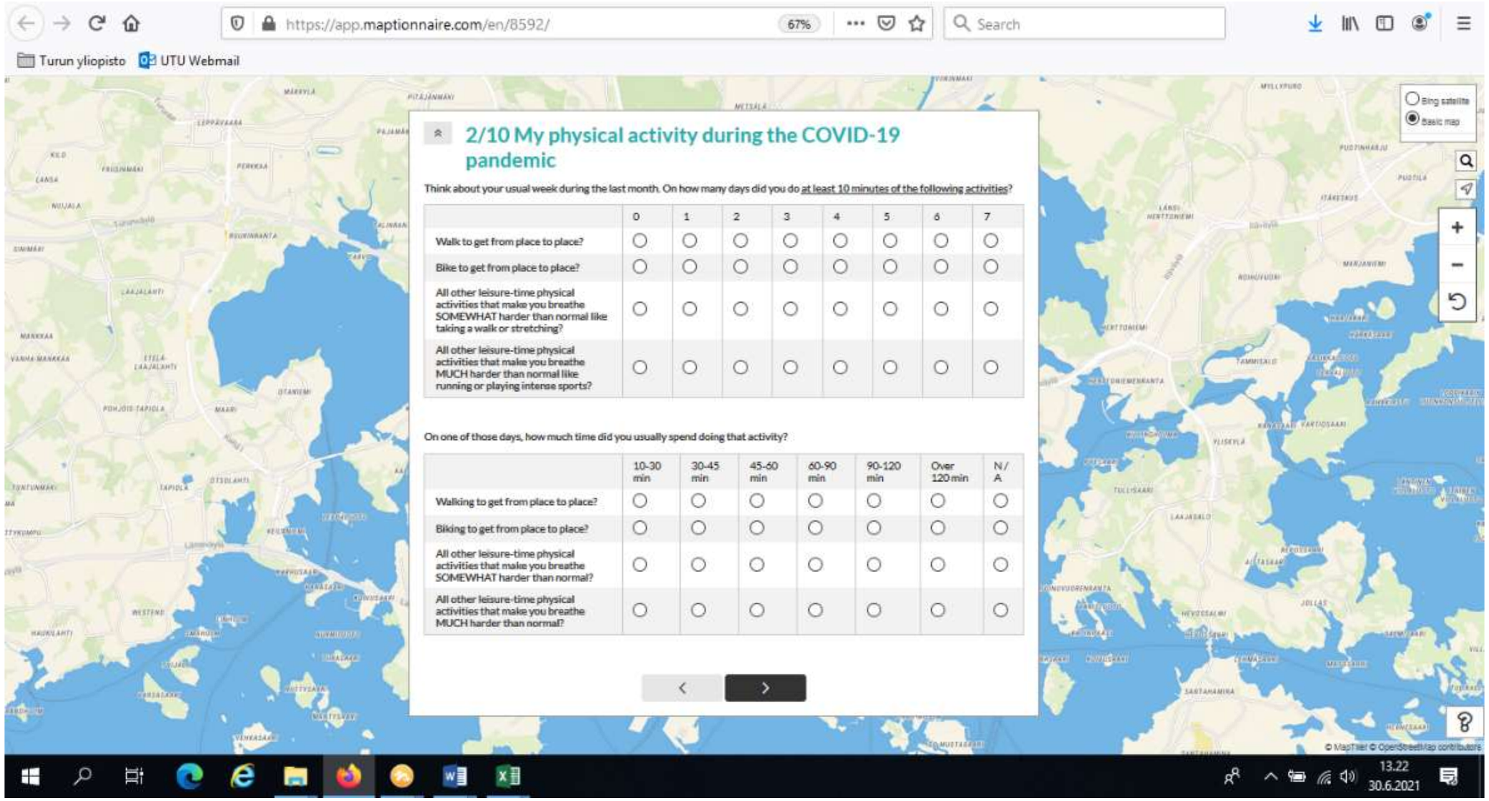
https://app.maptionnaire.com/en/8592/
Turun yliopisto
UTU Webmail
2/10 My physical activity during the COVID-19 pandemic
Think about your usual week during the last month. On how many days did you do at least 10 minutes of the following activities?
0 1 2 3 4 5 6 7
Walk to get from place to place?
Bike to get from place to place?
All other leisure-time physical activities that make you breathe SOMEWHAT harder than normal like taking a walk or stretching?
All other leisure-time physical activities that make you breathe MUCH harder than normal like running or playing intense sports?
On one of those days, how much time did you usually spend doing that activity?
10-30 min
30-45 min
45-60 min
60-90 min
90-120 min
Over 120 min
N/A
Walking to get from place to place?
Biking to get from place to place?
All other leisure-time physical activities that make you breathe SOMEWHAT harder than normal?
All other leisure-time physical activities that make you breathe MUCH harder than normal?
13.22
30.6.2021

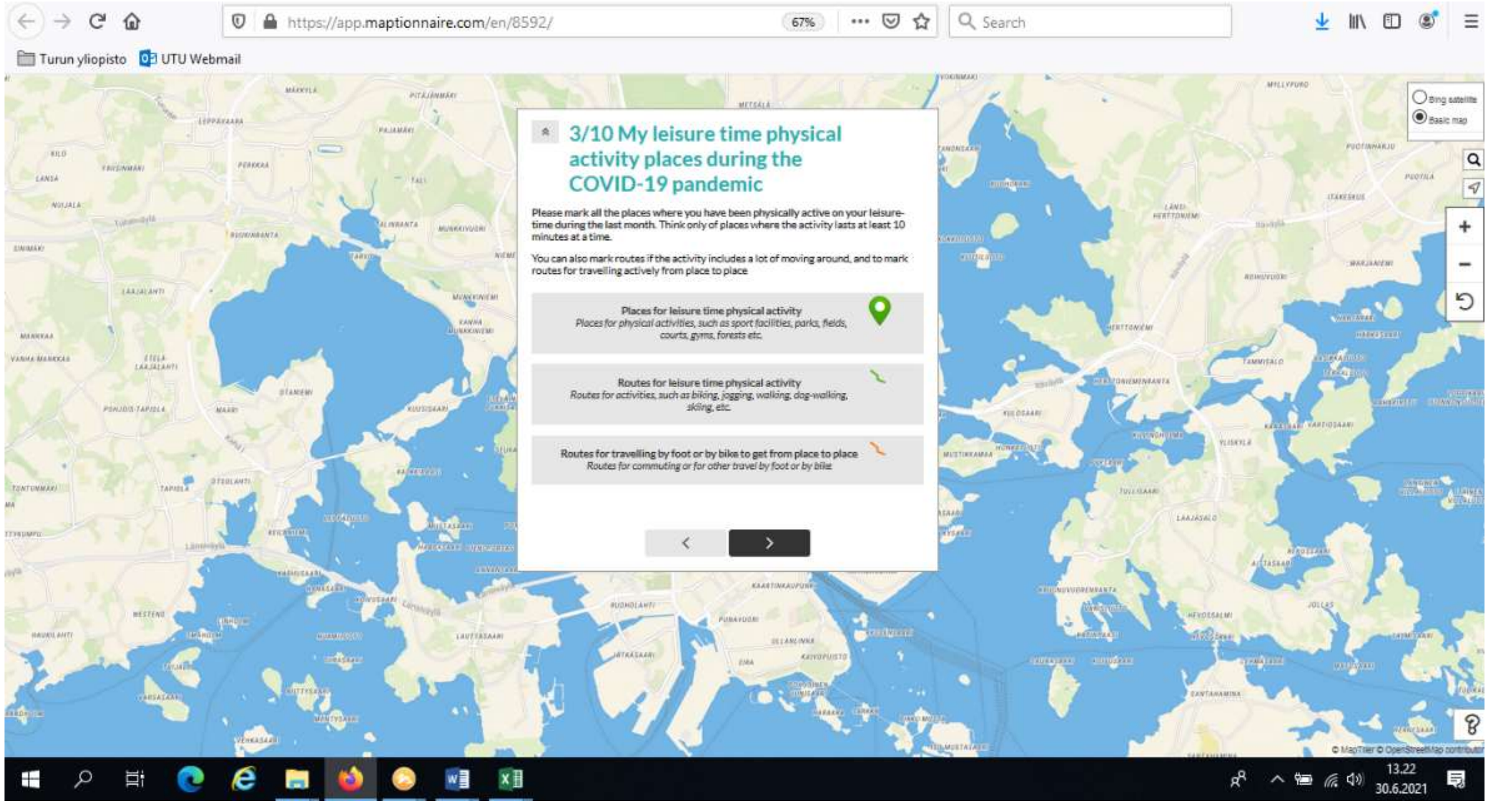
https://app.maptionnaire.com/en/8592/
67%
Search
Turun yliopisto
UTU Webmail
Bing satellite
Basic map
3/10 My leisure time physical activity places during the COVID-19 pandemic
Please mark all the places where you have been physically active on your leisure-time during the last month. Think only of places where the activity lasts at least 10 minutes at a time.
You can also mark routes if the activity includes a lot of moving around, and to mark routes for travelling actively from place to place
Places for leisure time physical activity
Places for physical activities, such as sport facilities, parks, fields, courts, gyms, forests etc.
Routes for leisure time physical activity
Routes for activities, such as biking, jogging, walking, dog-walking, skiing, etc.
Routes for travelling by foot or by bike to get from place to place
Routes for commuting or for other travel by foot or by bike
13.22
30.6.2021

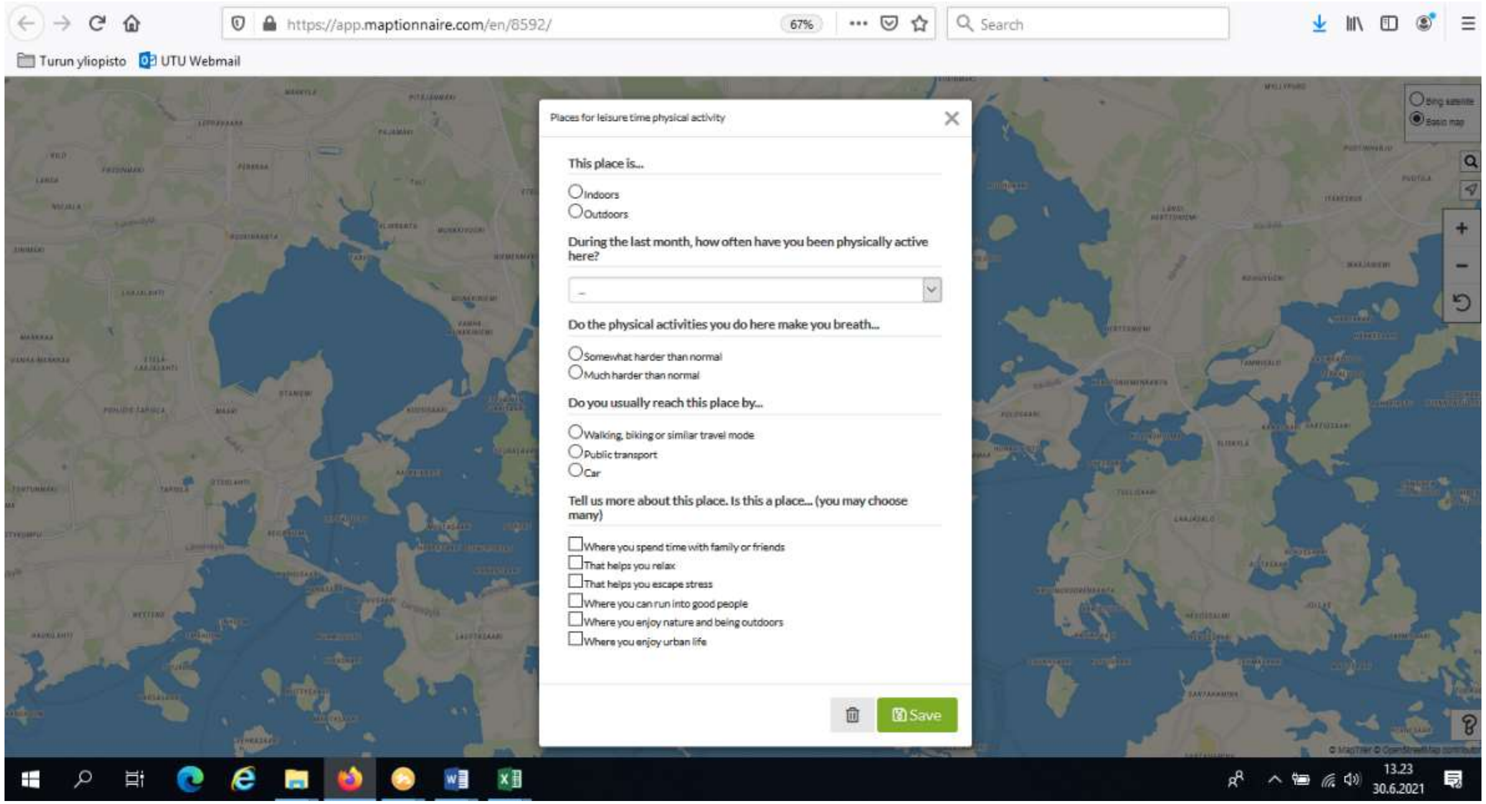
https://app.maptionnaire.com/en/8592/
67%
Search
Turun yliopisto
UTU Webmail
Places for leisure time physical activity
This place is...
Indoors
Outdoors
During the last month, how often have you been physically active here?
Do the physical activities you do here make you breath...
Somewhat harder than normal
Much harder than normal
Do you usually reach this place by...
Walking, biking or similar travel mode
Public transport
Car
Tell us more about this place. Is this a place... (you may choose many)
Where you spend time with family or friends
That helps you relax
That helps you escape stress
Where you can run into good people
Where you enjoy nature and being outdoors
Where you enjoy urban life
Save
13.23
30.6.2021

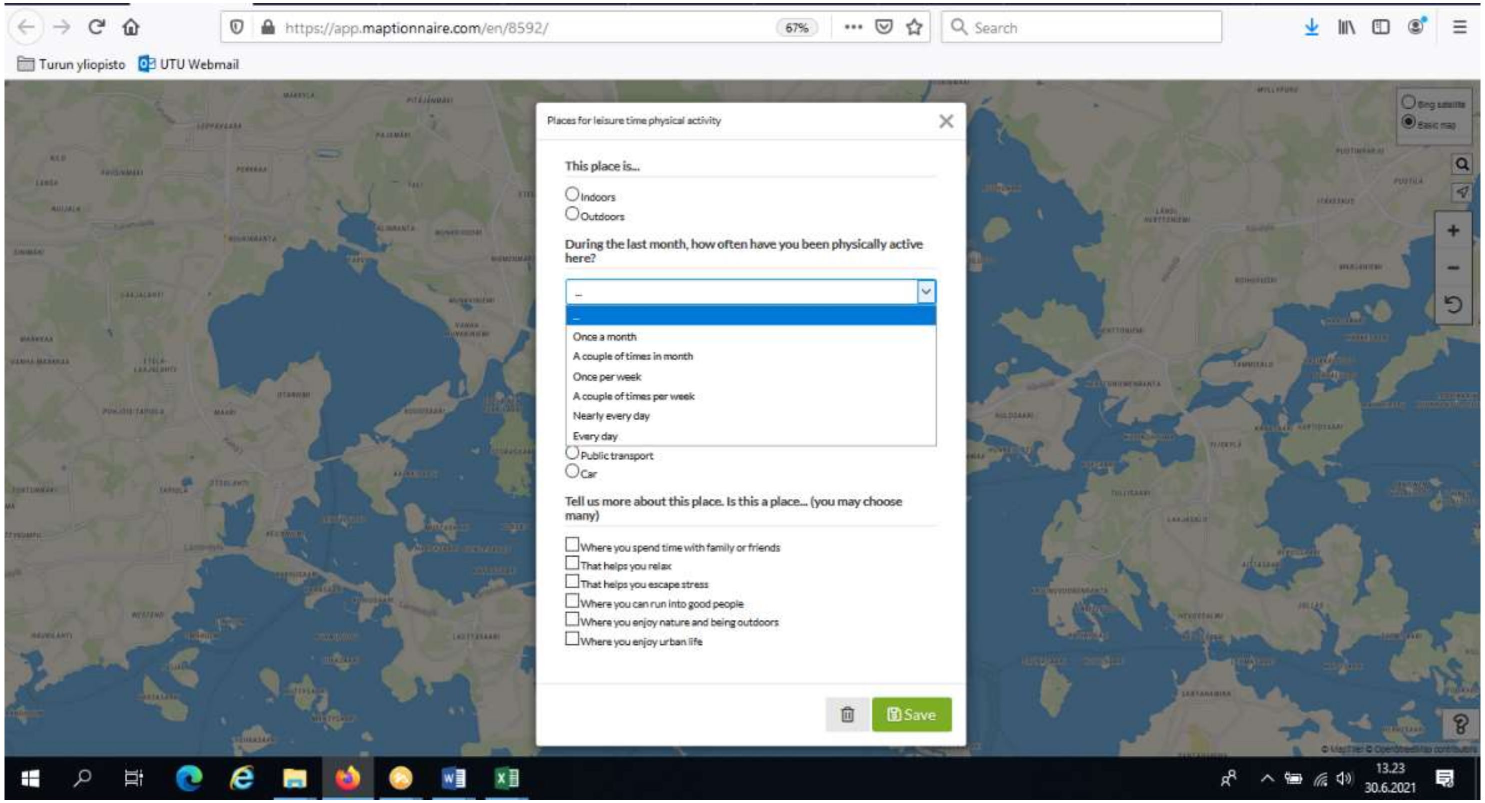
https://app.maptionnaire.com/en/8592/
67%
Search
Turun yliopisto
UTU Webmail
Places for leisure time physical activity
This place is...
Indoors
Outdoors
During the last month, how often have you been physically active here?
Once a month
A couple of times in month
Once per week
A couple of times per week
Nearly every day
Every day
Public transport
Car
Tell us more about this place. Is this a place... (you may choose many)
Where you spend time with family or friends
That helps you relax
That helps you escape stress
Where you can run into good people
Where you enjoy nature and being outdoors
Where you enjoy urban life
Save
13.23
30.6.2021

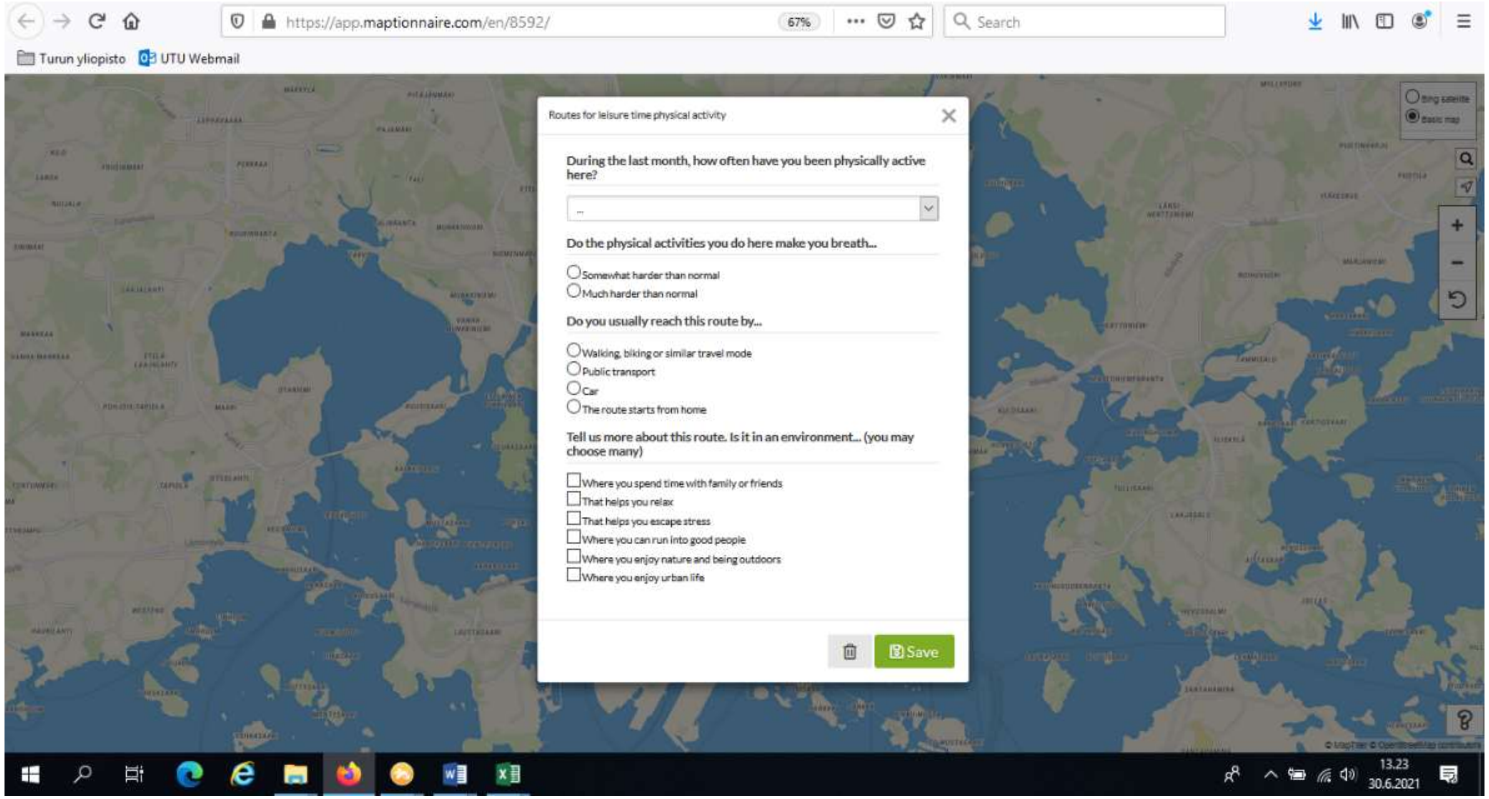
https://app.maptionnaire.com/en/8592/
67%
Search
Turun yliopisto
UTU Webmail
Routes for leisure time physical activity
During the last month, how often have you been physically active here?
Do the physical activities you do here make you breath...
Somewhat harder than normal
Much harder than normal
Do you usually reach this route by...
Walking, biking or similar travel mode
Public transport
Car
The route starts from home
Tell us more about this route. Is it in an environment... (you may choose many)
Where you spend time with family or friends
That helps you relax
That helps you escape stress
Where you can run into good people
Where you enjoy nature and being outdoors
Where you enjoy urban life
Save
13.23
30.6.2021

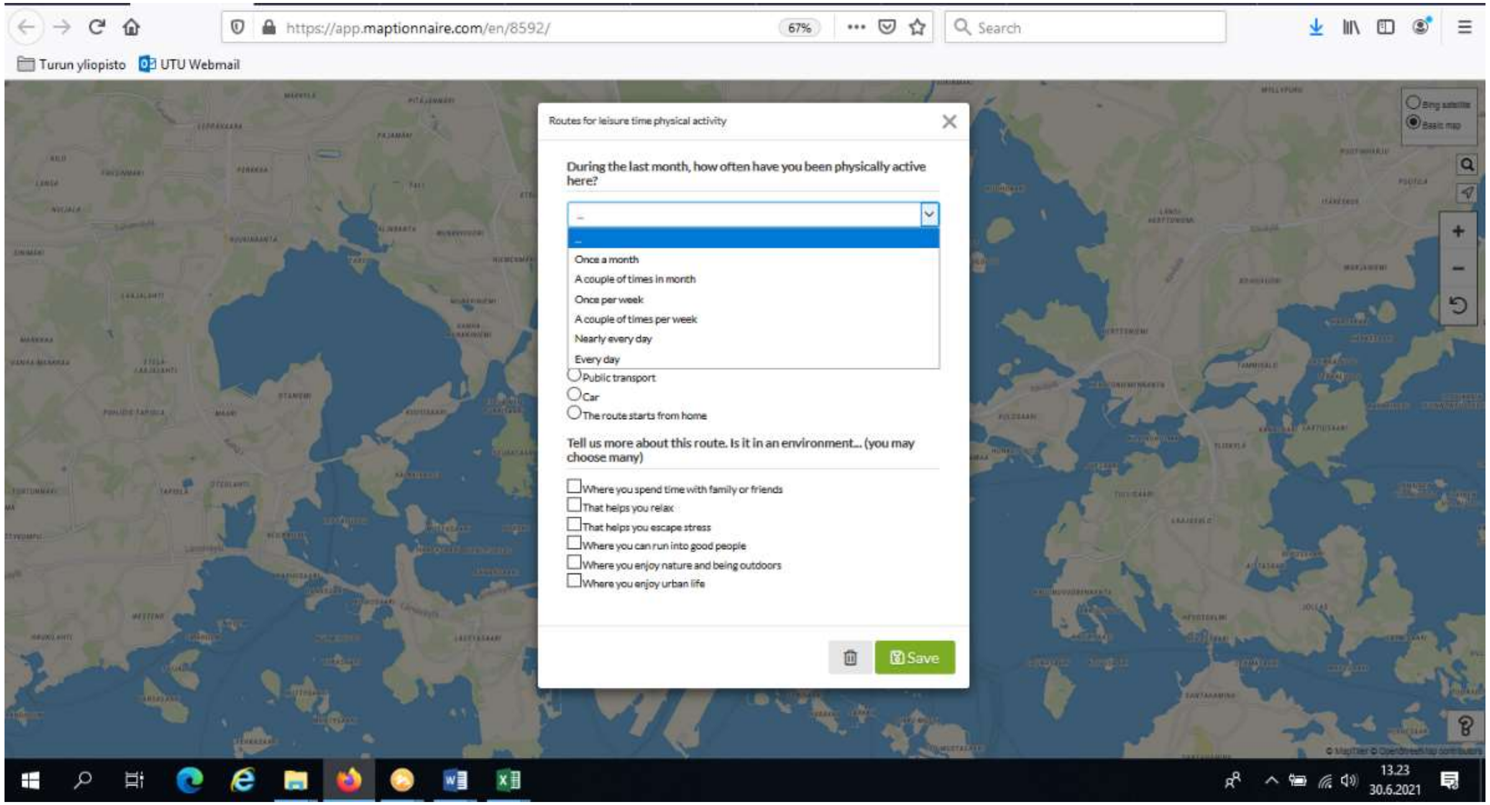

https://app.maptionnaire.com/en/8592/
Search
Turun yliopisto
UTU Webmail
Routes for leisure time physical activity
During the last month, how often have you been physically active here?
Once a month
A couple of times in month
Once per week
A couple of times per week
Nearly every day
Every day
Public transport
Car
The route starts from home
Tell us more about this route. Is it in an environment... (you may choose many)
Where you spend time with family or friends
That helps you relax
That helps you escape stress
Where you can run into good people
Where you enjoy nature and being outdoors
Where you enjoy urban life
Save
13.23
30.6.2021

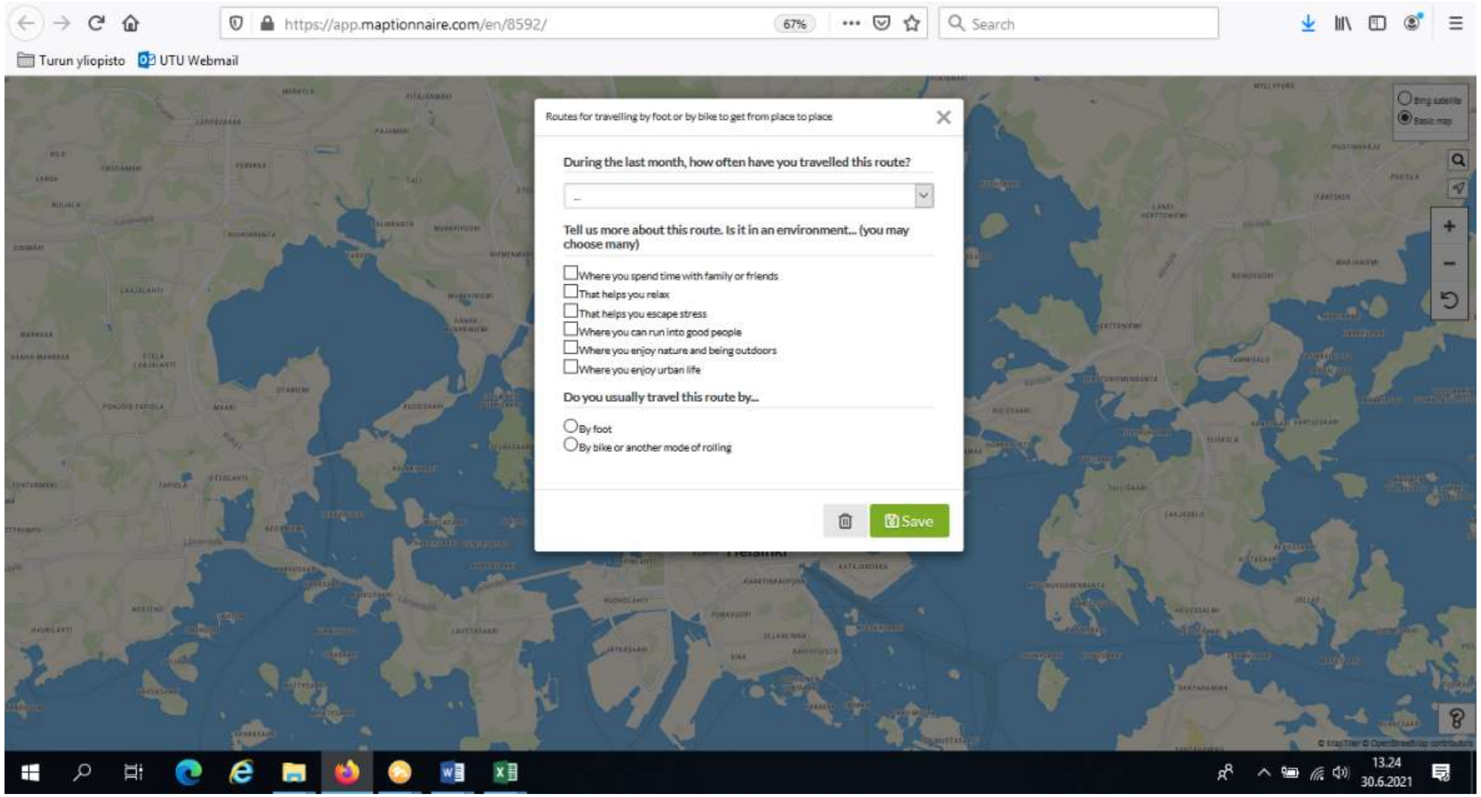
https://app.maptionnaire.com/en/8592/
67%
Search
Turun yliopisto
UTU Webmail
Routes for travelling by foot or by bike to get from place to place
During the last month, how often have you travelled this route?
Tell us more about this route. Is it in an environment... (you may choose many)
Where you spend time with family or friends
That helps you relax
That helps you escape stress
Where you can run into good people
Where you enjoy nature and being outdoors
Where you enjoy urban life
Do you usually travel this route by...
By foot
By bike or another mode of rolling
Save
13.24
30.6.2021

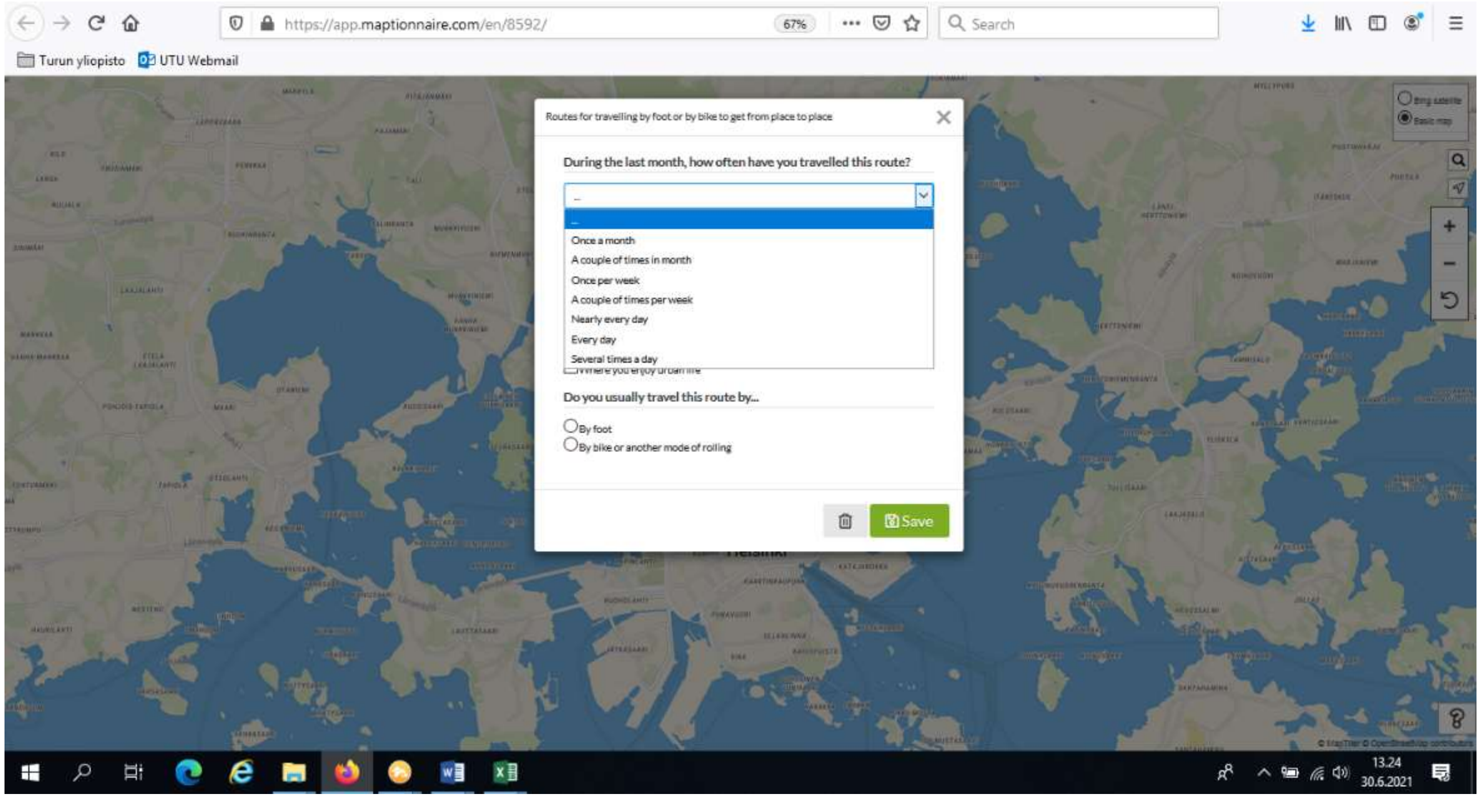
https://app.maptionnaire.com/en/8592/
Search
Turun yliopisto
UTU Webmail
Routes for travelling by foot or by bike to get from place to place
During the last month, how often have you travelled this route?
Once a month
A couple of times in month
Once per week
A couple of times per week
Nearly every day
Every day
Several times a day
Do you usually travel this route by...
By foot
By bike or another mode of rolling
Save
13.24
30.6.2021

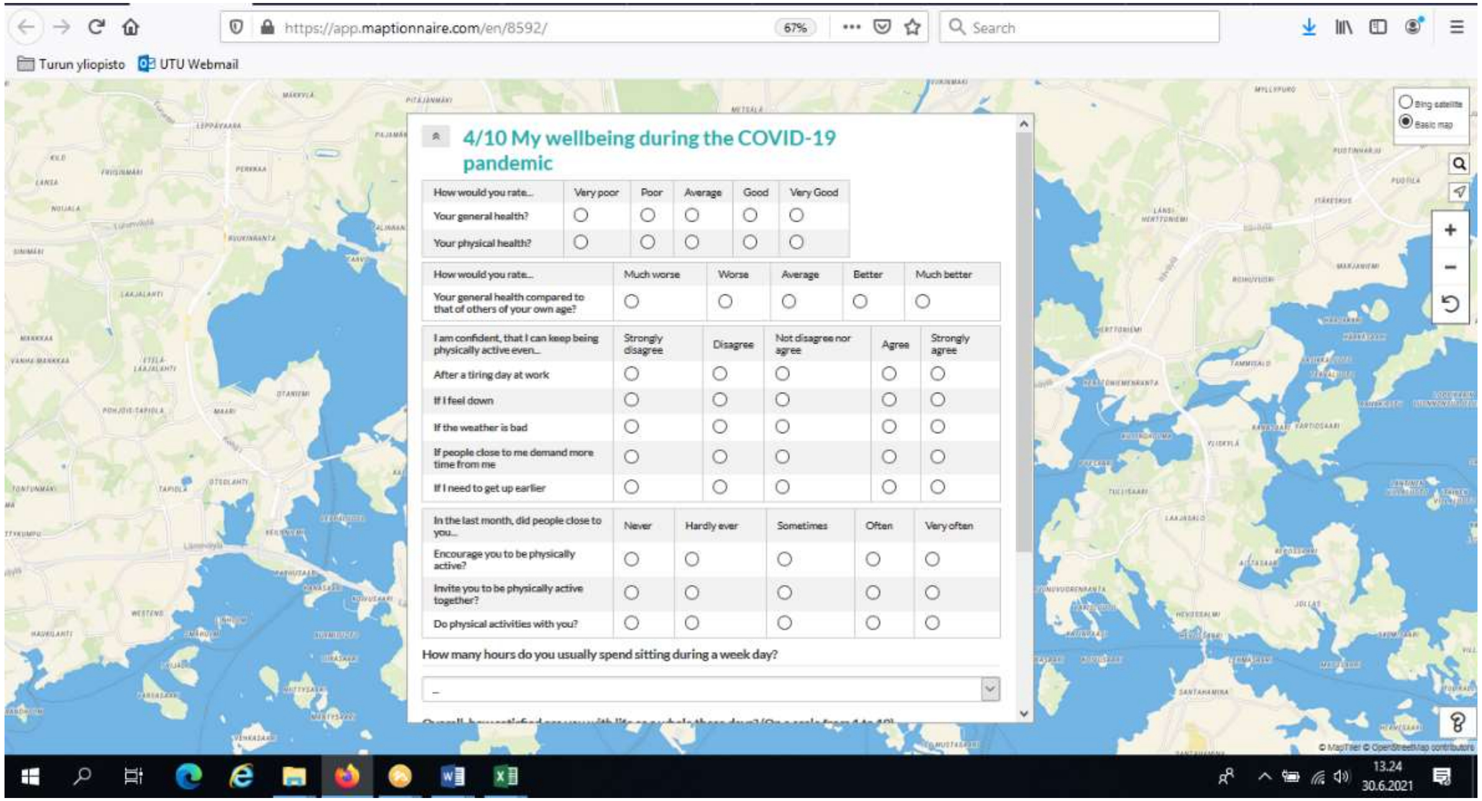

https://app.maptionnaire.com/en/8592/

4/10 My wellbeing during the COVID-19 pandemic

How would you rate...	Very poor	Poor	Average	Good	Very Good
Your general health?	○	○	○	○	○
Your physical health?	○	○	○	○	○

How would you rate...	Much worse	Worse	Average	Better	Much better
Your general health compared to that of others of your own age?	○	○	○	○	○

I am confident, that I can keep being physically active even...	Strongly disagree	Disagree	Not disagree nor agree	Agree	Strongly agree
After a tiring day at work	○	○	○	○	○
If I feel down	○	○	○	○	○
If the weather is bad	○	○	○	○	○
If people close to me demand more time from me	○	○	○	○	○
If I need to get up earlier	○	○	○	○	○

In the last month, did people close to you...	Never	Hardly ever	Sometimes	Often	Very often
Encourage you to be physically active?	○	○	○	○	○
Invite you to be physically active together?	○	○	○	○	○
Do physical activities with you?	○	○	○	○	○

How many hours do you usually spend sitting during a week day?

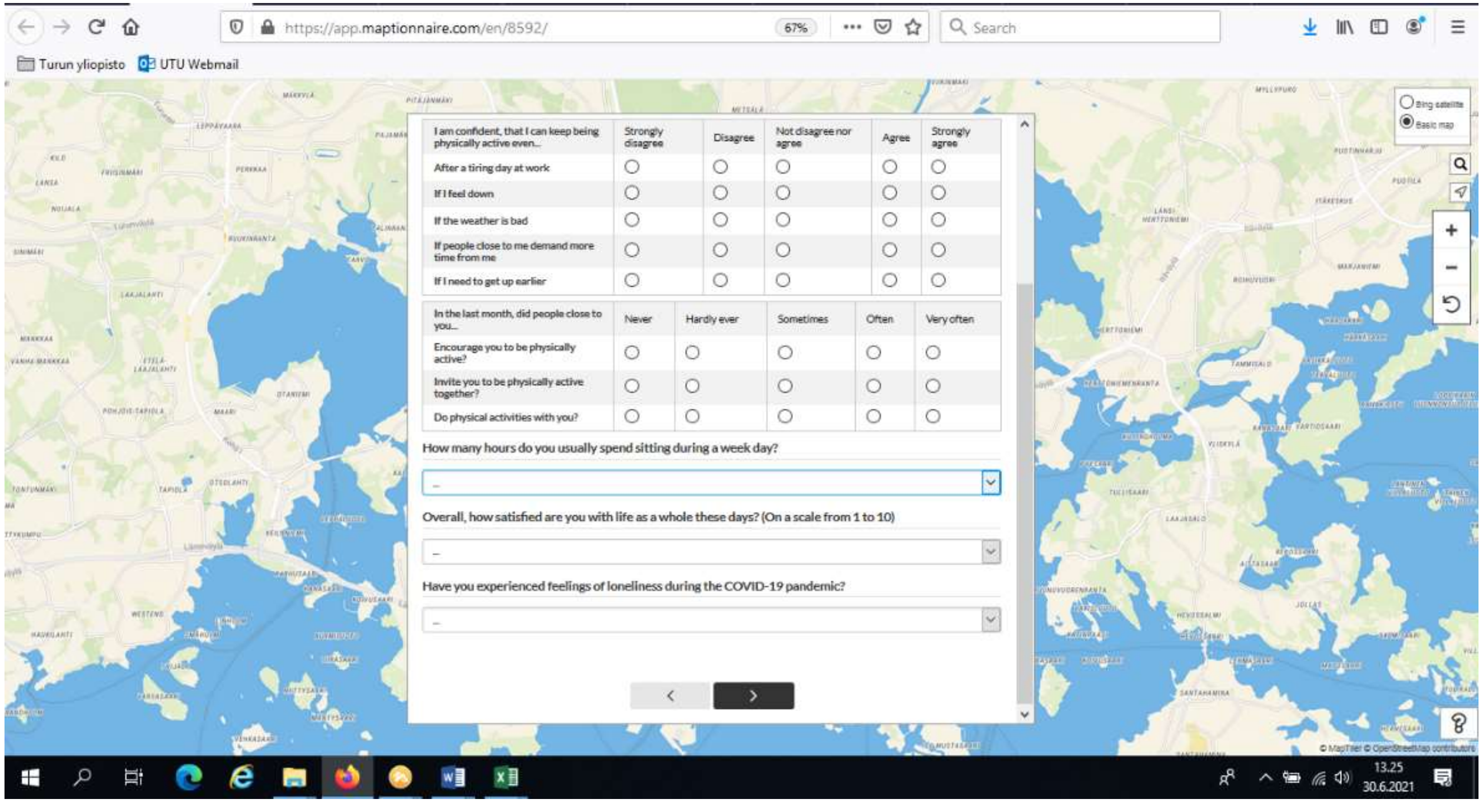
https://app.maptionnaire.com/en/8592/
67%
Search
Turun yliopisto
UTU Webmail
I am confident, that I can keep being physically active even...
Strongly disagree
Disagree
Not disagree nor agree
Agree
Strongly agree
After a tiring day at work
If I feel down
If the weather is bad
If people close to me demand more time from me
If I need to get up earlier
In the last month, did people close to you...
Never
Hardly ever
Sometimes
Often
Very often
Encourage you to be physically active?
Invite you to be physically active together?
Do physical activities with you?
How many hours do you usually spend sitting during a week day?
Overall, how satisfied are you with life as a whole these days? (On a scale from 1 to 10)
Have you experienced feelings of loneliness during the COVID-19 pandemic?
Bing satellite
Basic map
13.25
30.6.2021

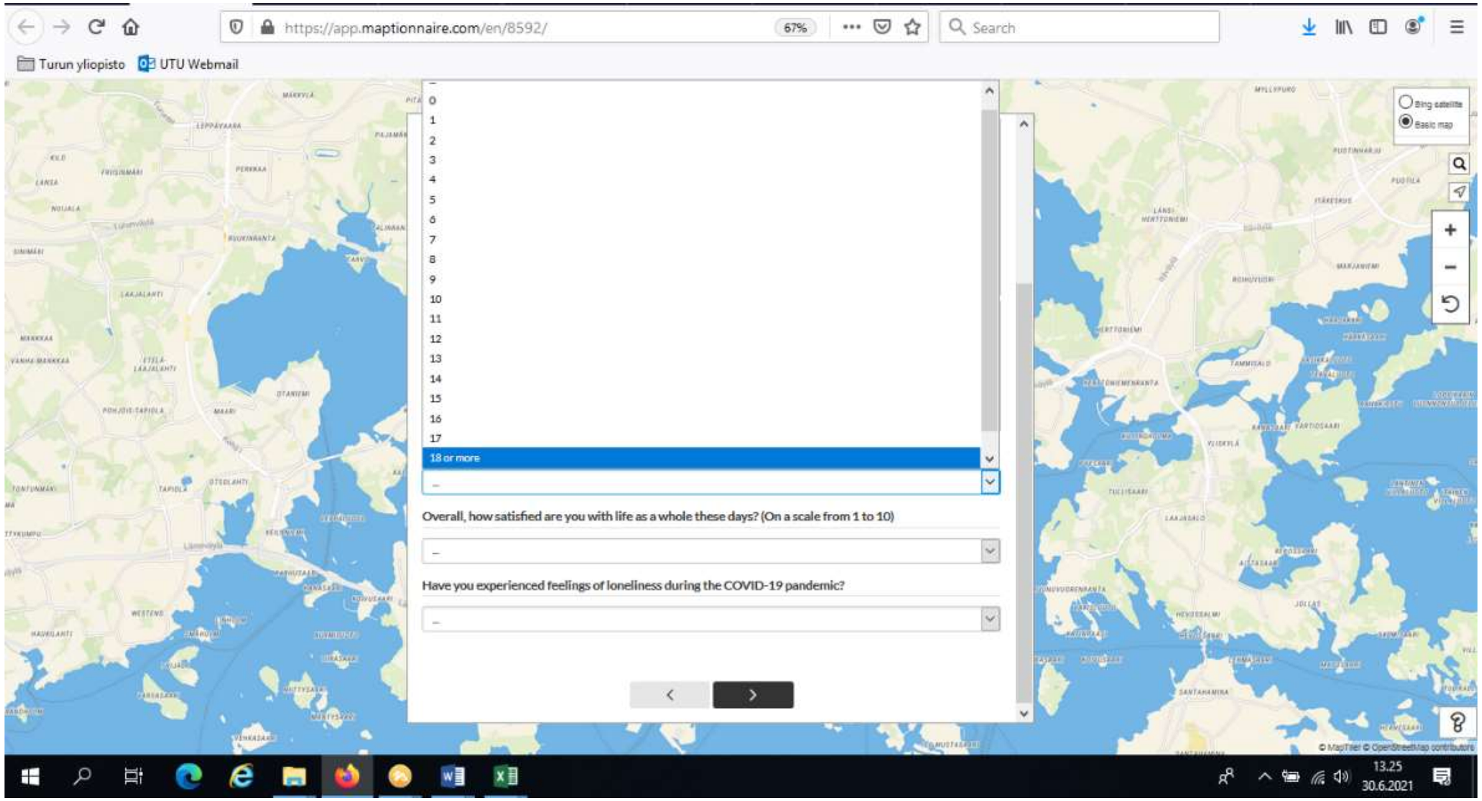
https://app.maptionnaire.com/en/8592/
67%
Search
Turun yliopisto
UTU Webmail
0
1
2
3
4
5
6
7
8
9
10
11
12
13
14
15
16
17
18 or more
Overall, how satisfied are you with life as a whole these days? (On a scale from 1 to 10)
Have you experienced feelings of loneliness during the COVID-19 pandemic?
Bing satellite
Basic map
13.25
30.6.2021

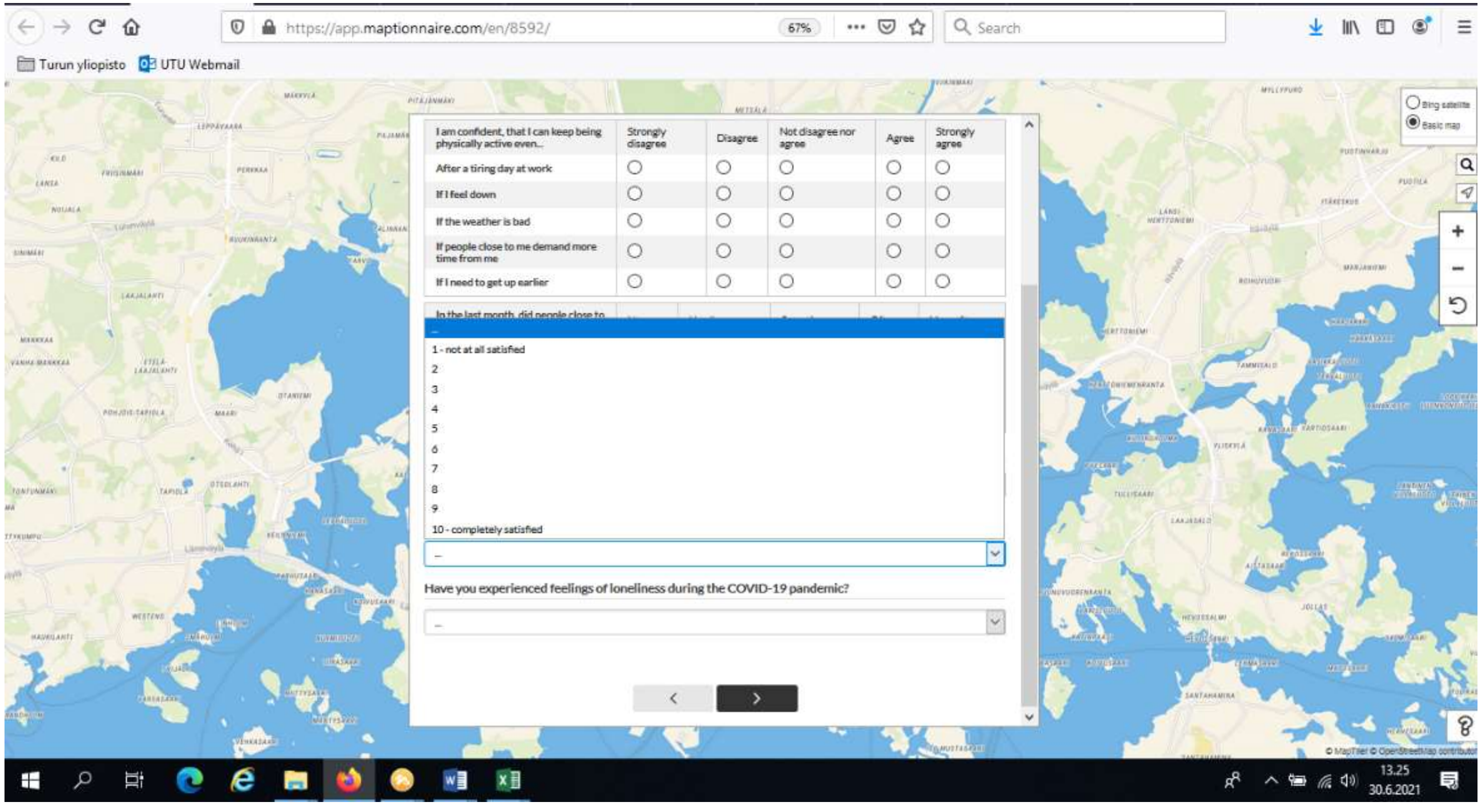
https://app.maptionnaire.com/en/8592/
Turun yliopisto
UTU Webmail
I am confident, that I can keep being physically active even...
Strongly disagree
Disagree
Not disagree nor agree
Agree
Strongly agree
After a tiring day at work
If I feel down
If the weather is bad
If people close to me demand more time from me
If I need to get up earlier
1 - not at all satisfied
2
3
4
5
6
7
8
9
10 - completely satisfied
Have you experienced feelings of loneliness during the COVID-19 pandemic?
Bing satellite
Basic map
13.25
30.6.2021

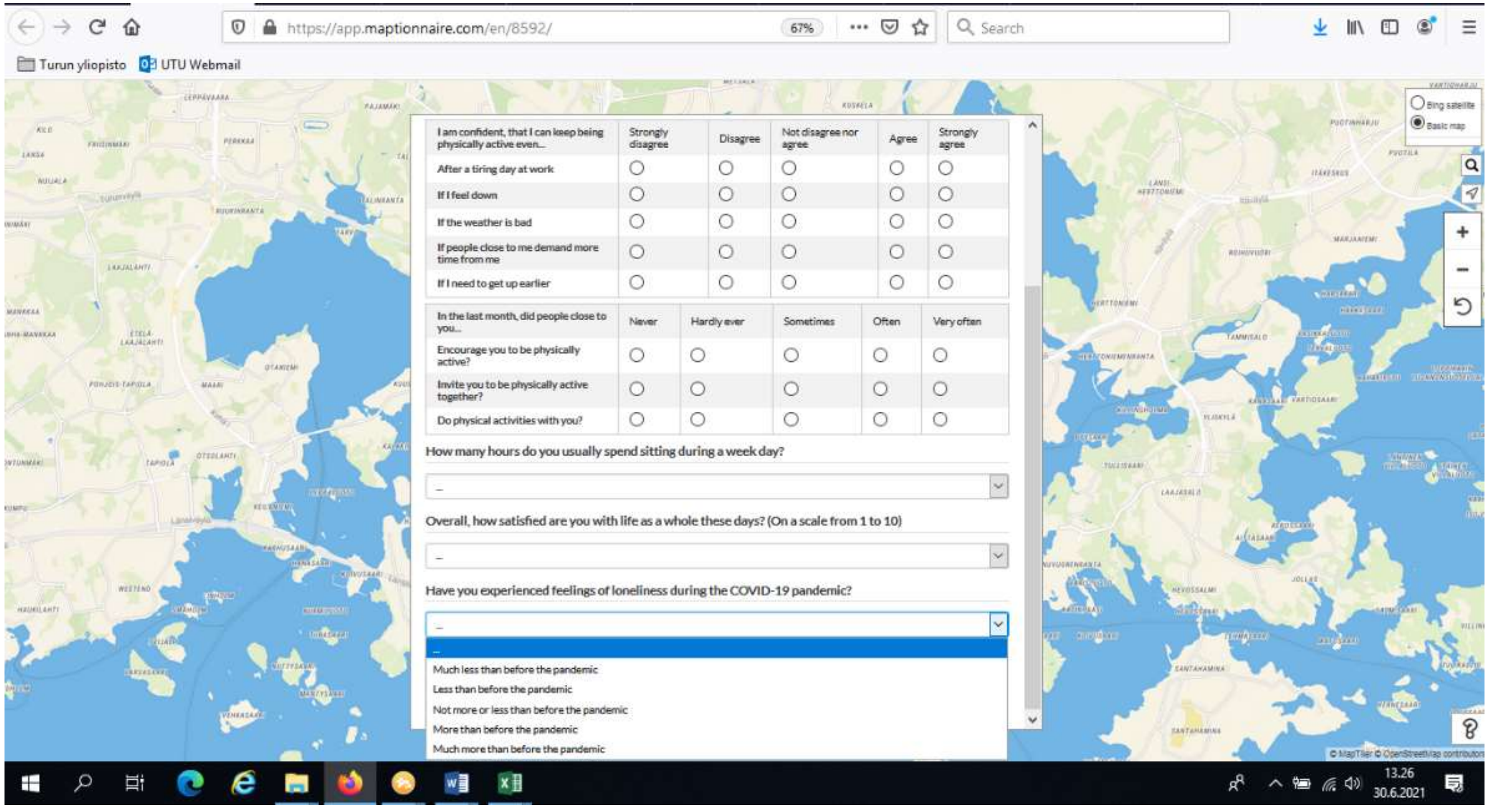
https://app.maptionnaire.com/en/8592/
Turun yliopisto
UTU Webmail
I am confident, that I can keep being physically active even...
Strongly disagree
Disagree
Not disagree nor agree
Agree
Strongly agree
After a tiring day at work
If I feel down
If the weather is bad
If people close to me demand more time from me
If I need to get up earlier
In the last month, did people close to you...
Never
Hardly ever
Sometimes
Often
Very often
Encourage you to be physically active?
Invite you to be physically active together?
Do physical activities with you?
How many hours do you usually spend sitting during a week day?
Overall, how satisfied are you with life as a whole these days? (On a scale from 1 to 10)
Have you experienced feelings of loneliness during the COVID-19 pandemic?
Much less than before the pandemic
Less than before the pandemic
Not more or less than before the pandemic
More than before the pandemic
Much more than before the pandemic
13.26
30.6.2021

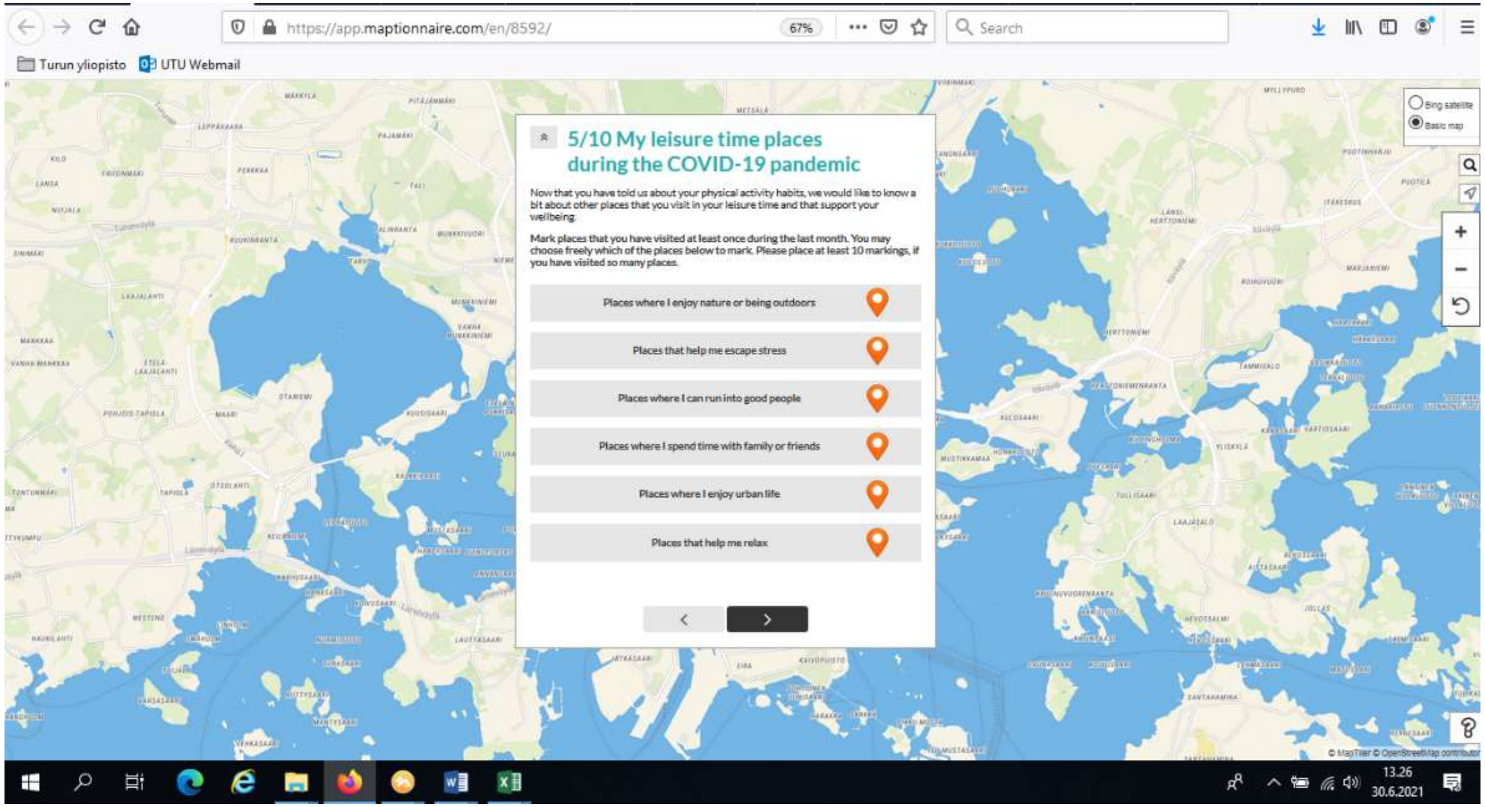
https://app.maptionnaire.com/en/8592/
67%
Search
Turun yliopisto
UTU Webmail
5/10 My leisure time places during the COVID-19 pandemic
Now that you have told us about your physical activity habits, we would like to know a bit about other places that you visit in your leisure time and that support your wellbeing.
Mark places that you have visited at least once during the last month. You may choose freely which of the places below to mark. Please place at least 10 markings, if you have visited so many places.
Places where I enjoy nature or being outdoors
Places that help me escape stress
Places where I can run into good people
Places where I spend time with family or friends
Places where I enjoy urban life
Places that help me relax
Bing satellite
Basic map
13.26
30.6.2021

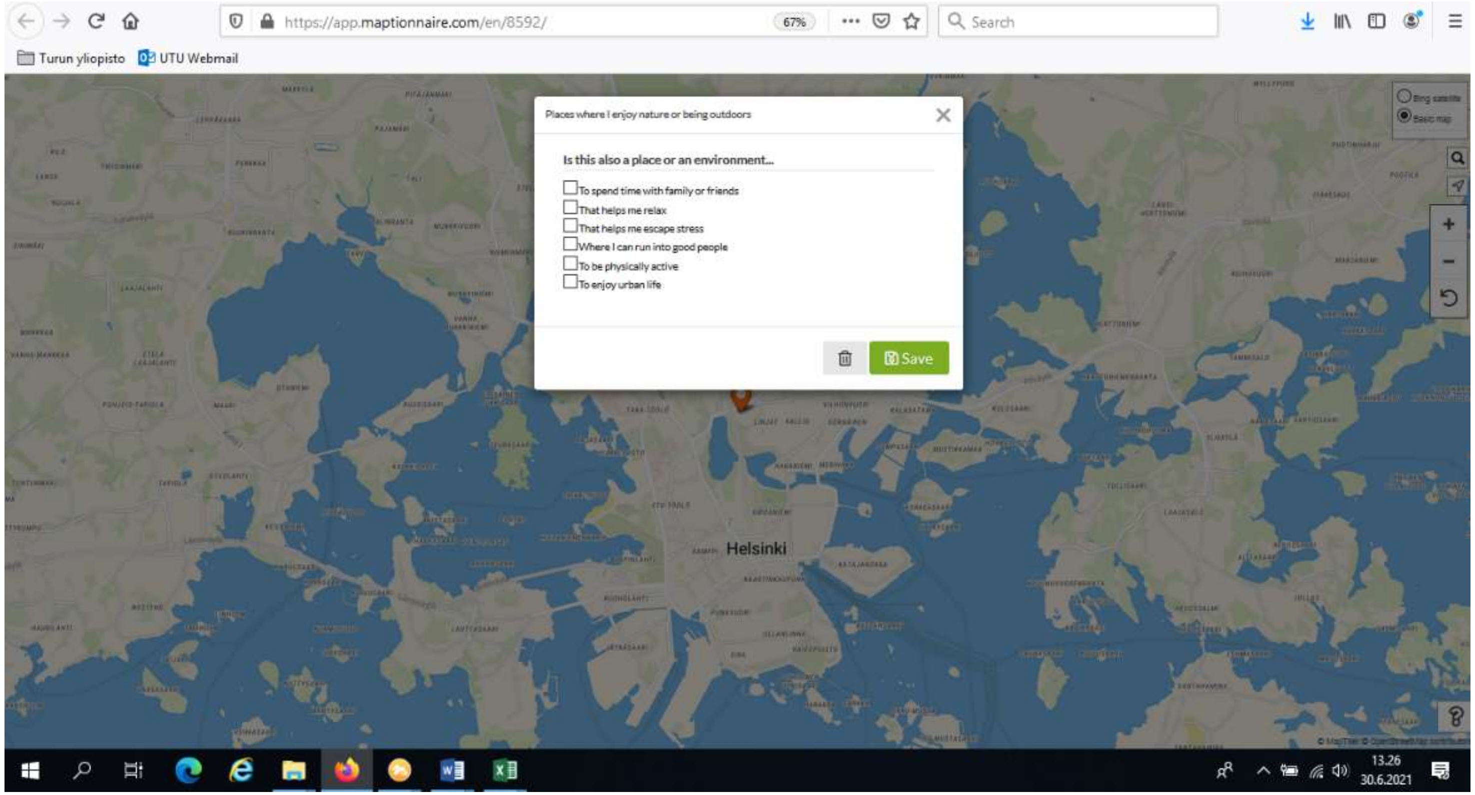
https://app.maptionnaire.com/en/8592/
67%
Search
Turun yliopisto
UTU Webmail
Places where I enjoy nature or being outdoors
Is this also a place or an environment...
To spend time with family or friends
That helps me relax
That helps me escape stress
Where I can run into good people
To be physically active
To enjoy urban life
Save
Helsinki
13.26
30.6.2021

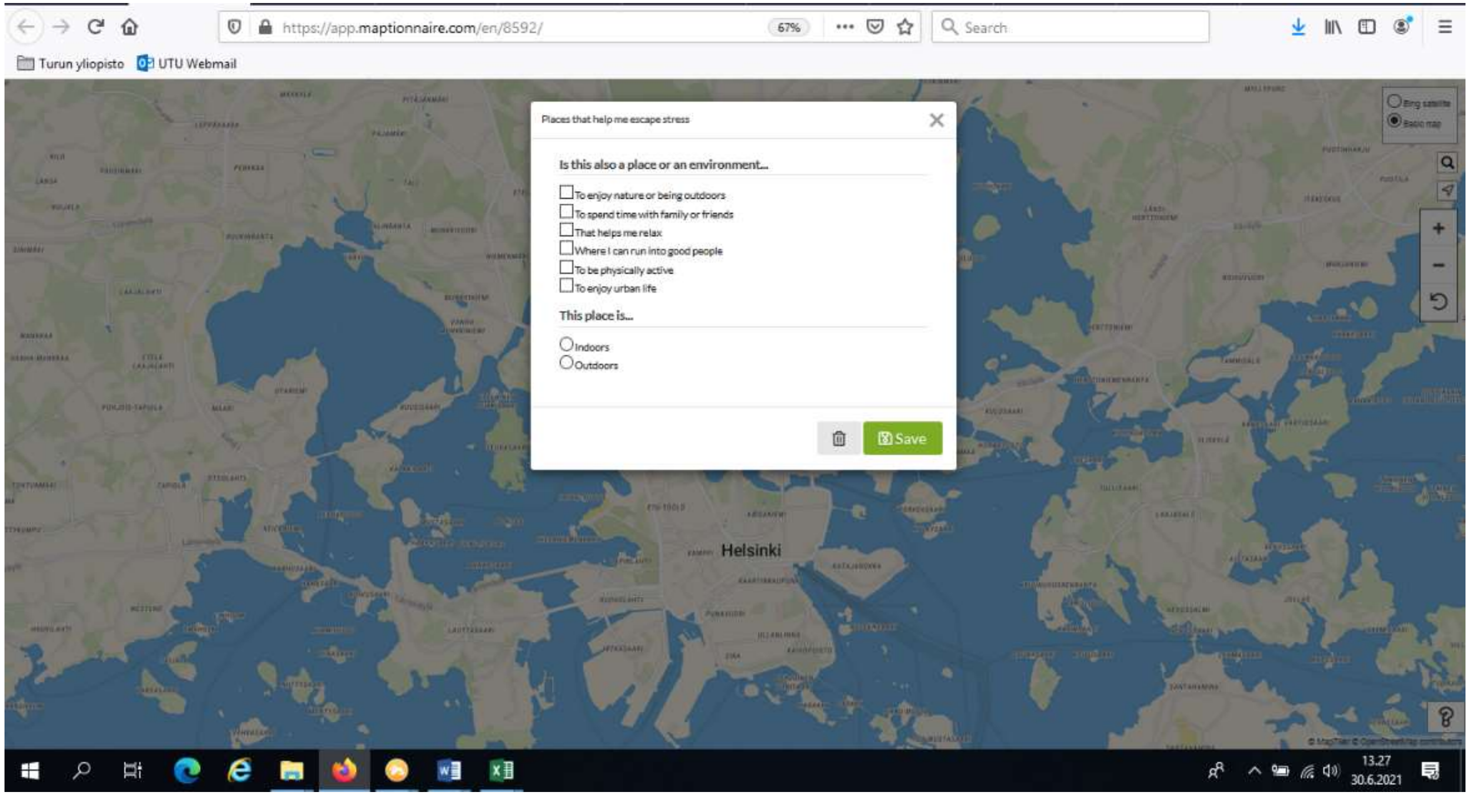

https://app.maptionnaire.com/en/8592/
Turun yliopisto
UTU Webmail
Places that help me escape stress
Is this also a place or an environment...
To enjoy nature or being outdoors
To spend time with family or friends
That helps me relax
Where I can run into good people
To be physically active
To enjoy urban life
This place is...
Indoors
Outdoors
Save
Helsinki
13.27
30.6.2021

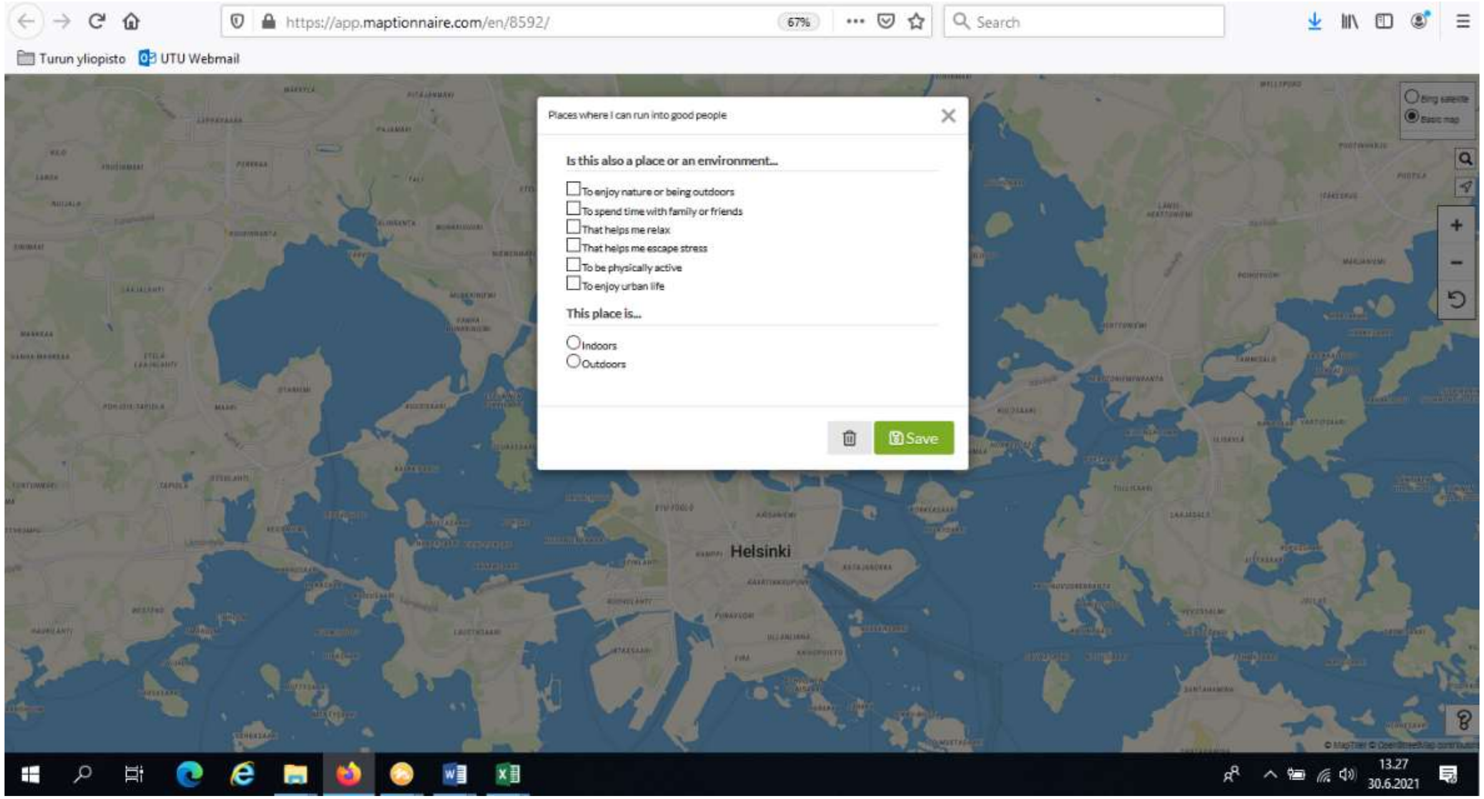

https://app.maptionnaire.com/en/8592/
Turun yliopisto
UTU Webmail
Places where I can run into good people
Is this also a place or an environment...
To enjoy nature or being outdoors
To spend time with family or friends
That helps me relax
That helps me escape stress
To be physically active
To enjoy urban life
This place is...
Indoors
Outdoors
Save
Helsinki
13.27
30.6.2021

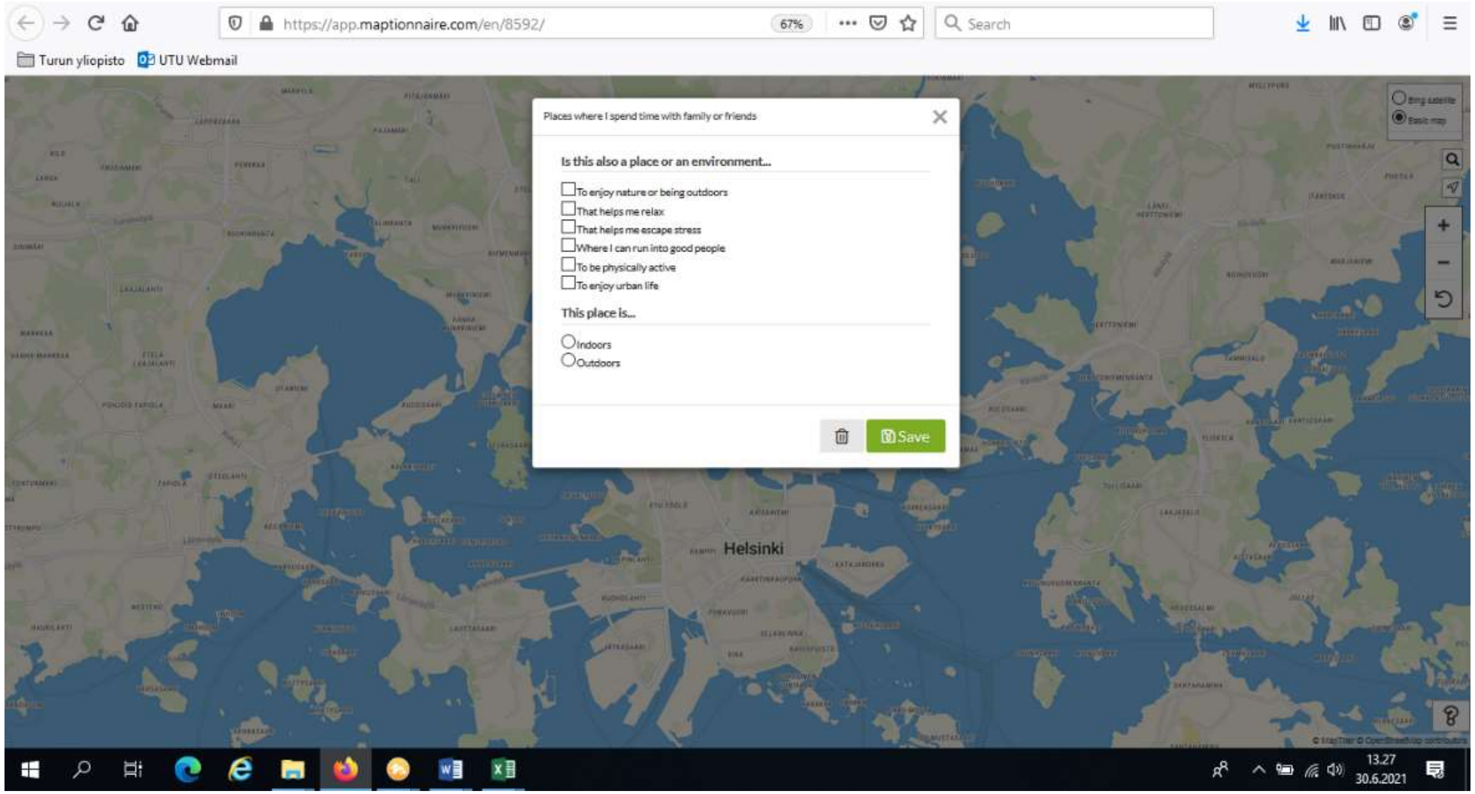
https://app.maptionnaire.com/en/8592/
Turun yliopisto
UTU Webmail
Places where I spend time with family or friends
Is this also a place or an environment...
To enjoy nature or being outdoors
That helps me relax
That helps me escape stress
Where I can run into good people
To be physically active
To enjoy urban life
This place is...
Indoors
Outdoors
Save
Helsinki
13.27
30.6.2021

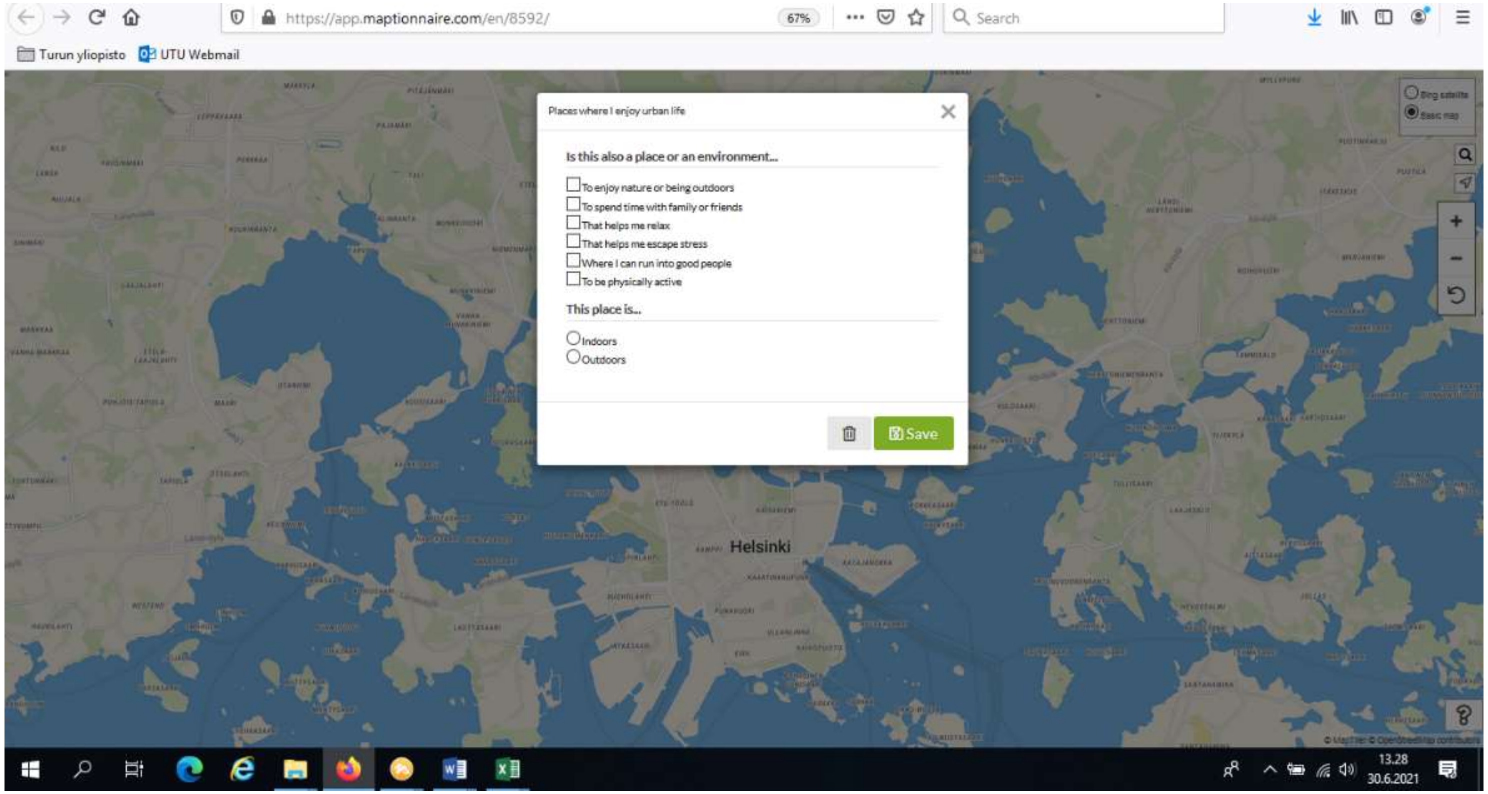
https://app.maptionnaire.com/en/8592/
Turun yliopisto
UTU Webmail
Places where I enjoy urban life
Is this also a place or an environment...
To enjoy nature or being outdoors
To spend time with family or friends
That helps me relax
That helps me escape stress
Where I can run into good people
To be physically active
This place is...
Indoors
Outdoors
Save
Helsinki
13.28
30.6.2021

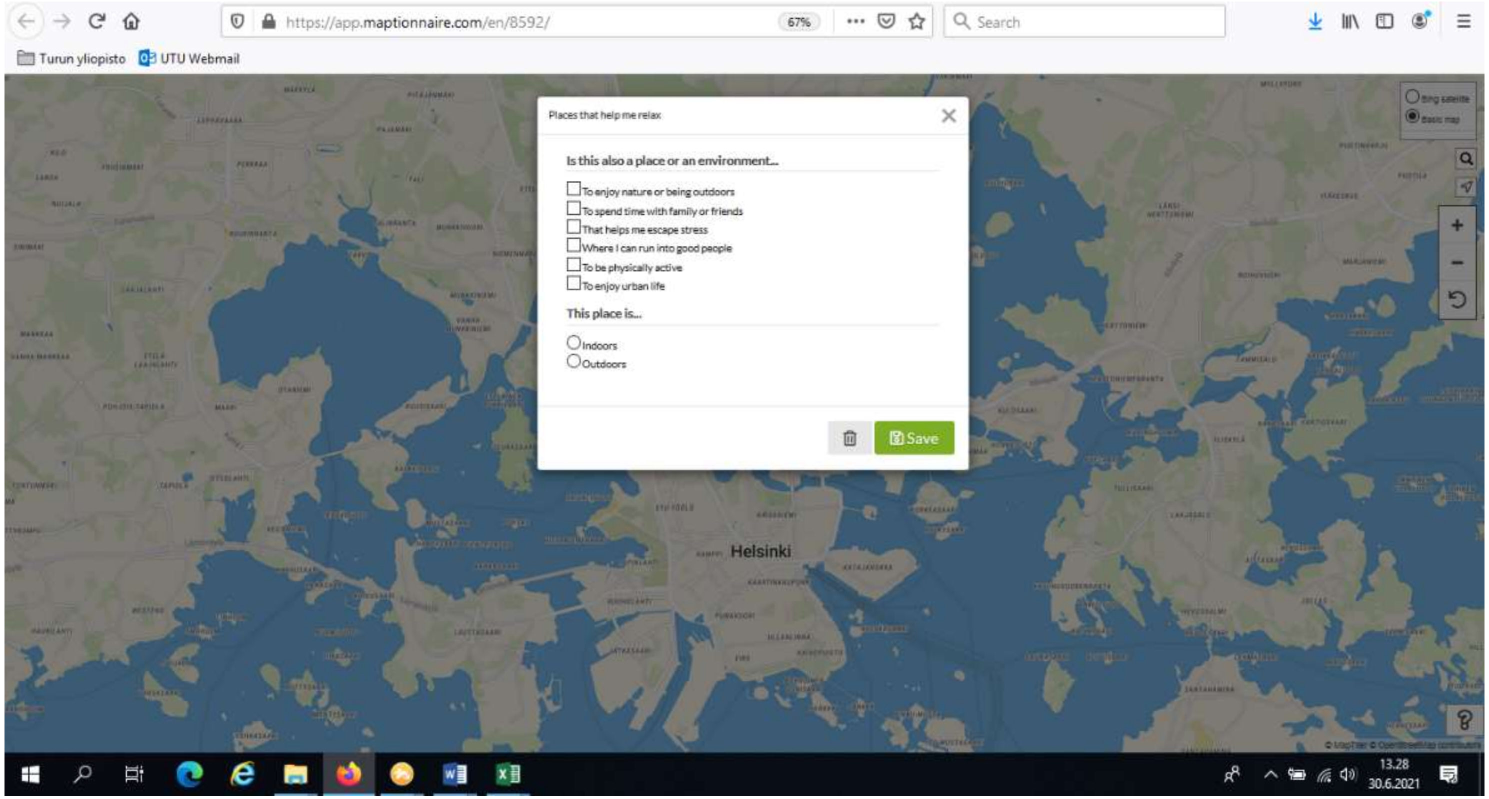
https://app.maptionnaire.com/en/8592/
67%
Search
Turun yliopisto
UTU Webmail
Places that help me relax
Is this also a place or an environment...
To enjoy nature or being outdoors
To spend time with family or friends
That helps me escape stress
Where I can run into good people
To be physically active
To enjoy urban life
This place is...
Indoors
Outdoors
Save
Helsinki
13.28
30.6.2021

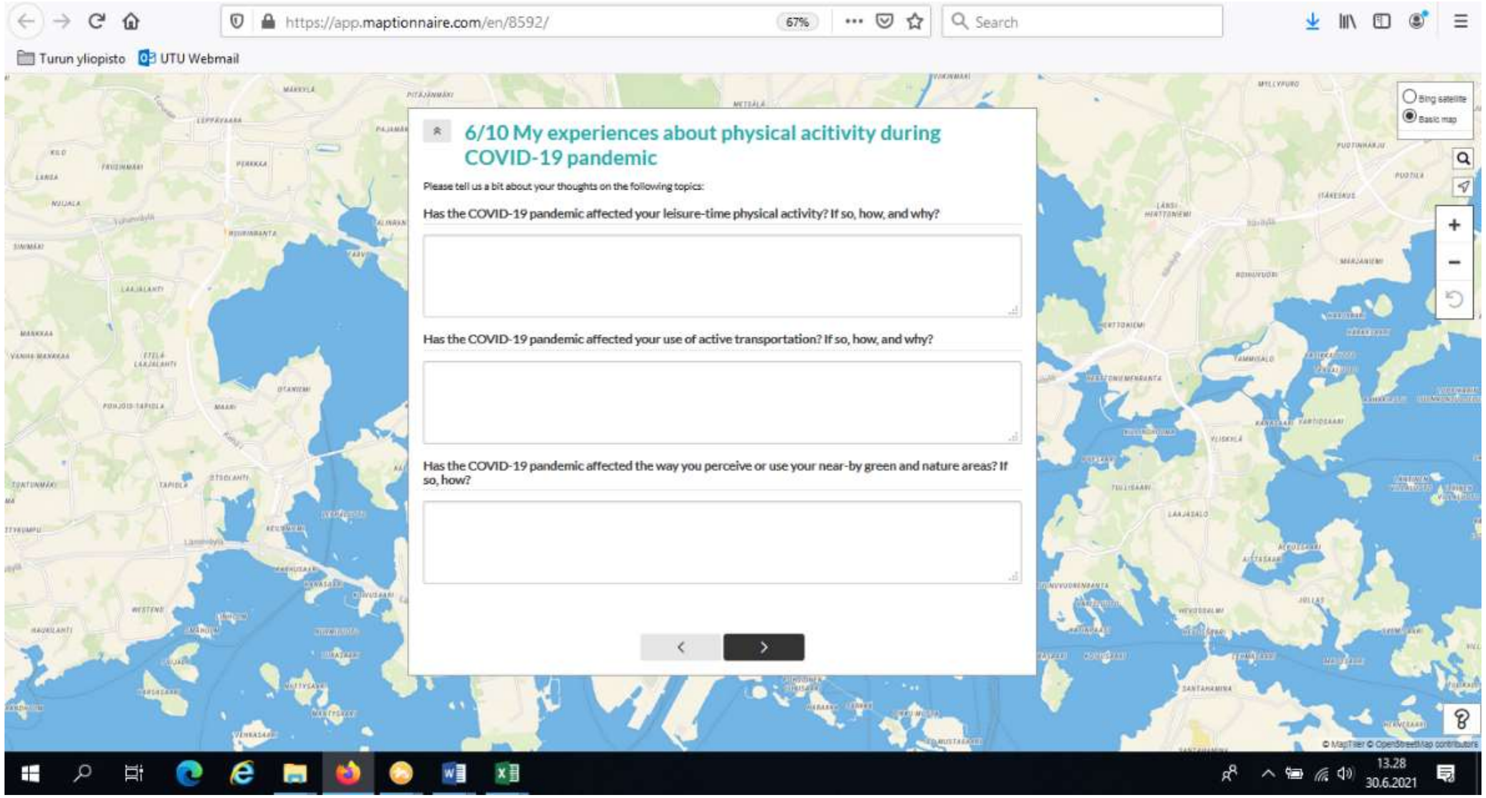

https://app.maptionnaire.com/en/8592/
Turun yliopisto
UTU Webmail
6/10 My experiences about physical acitivity during COVID-19 pandemic
Please tell us a bit about your thoughts on the following topics:
Has the COVID-19 pandemic affected your leisure-time physical activity? If so, how, and why?
Has the COVID-19 pandemic affected your use of active transportation? If so, how, and why?
Has the COVID-19 pandemic affected the way you perceive or use your near-by green and nature areas? If so, how?
Bing satellite
Basic map
13.28
30.6.2021

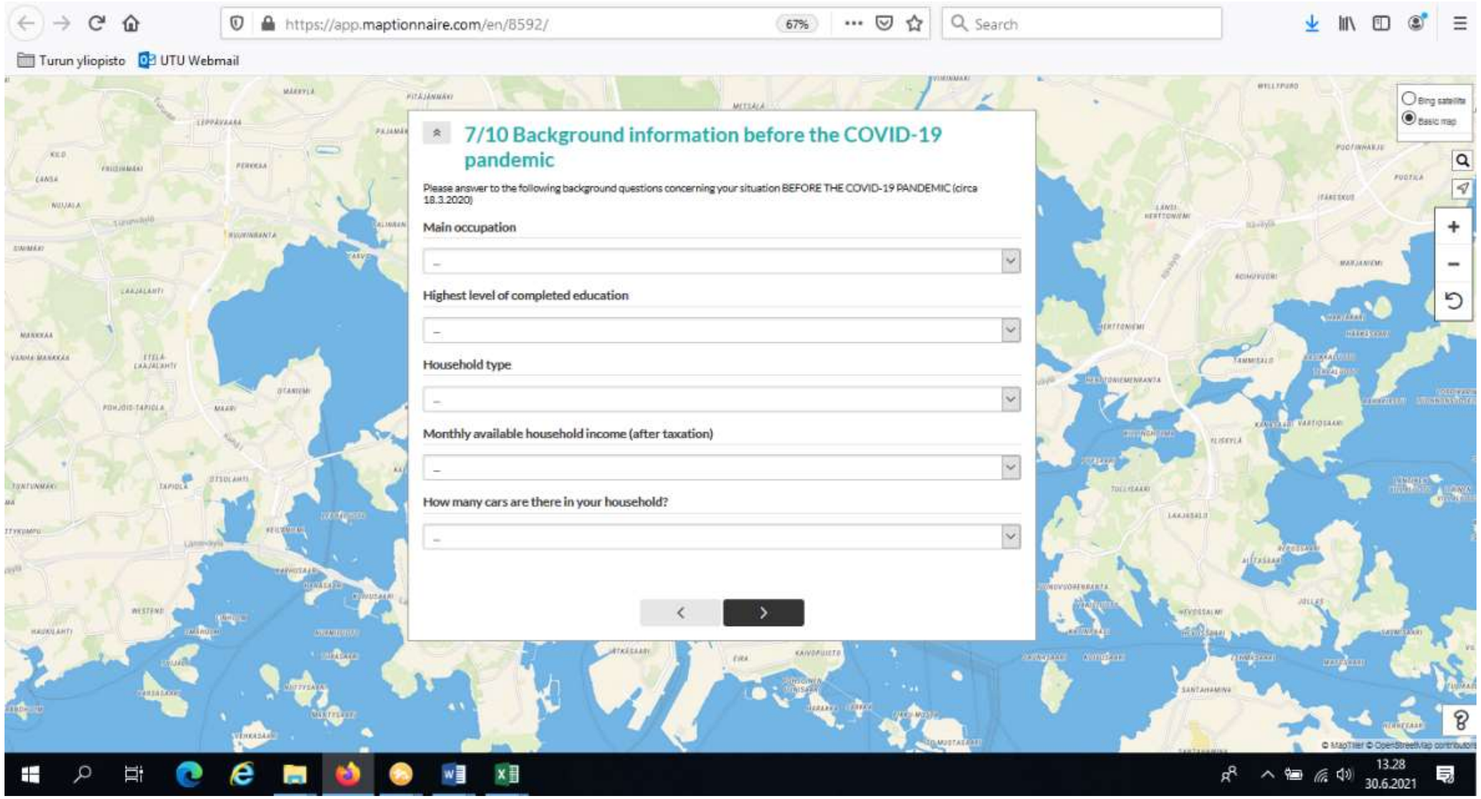
https://app.maptionnaire.com/en/8592/
Turun yliopisto
UTU Webmail
7/10 Background information before the COVID-19 pandemic
Please answer to the following background questions concerning your situation BEFORE THE COVID-19 PANDEMIC (circa 18.3.2020)
Main occupation
Highest level of completed education
Household type
Monthly available household income (after taxation)
How many cars are there in your household?
Bing satellite
Basic map
13.28
30.6.2021

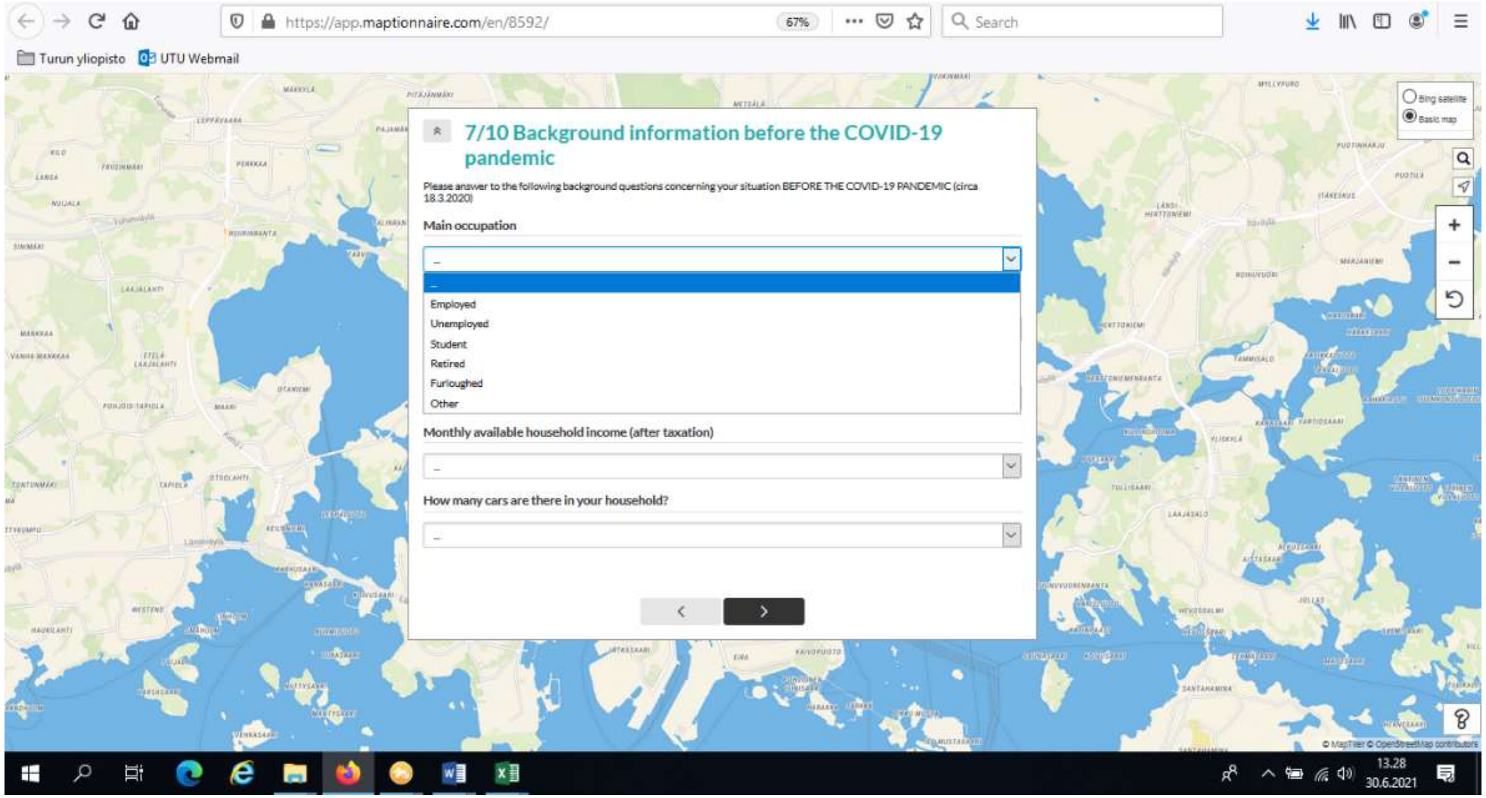
https://app.maptionnaire.com/en/8592/
Turun yliopisto
UTU Webmail
7/10 Background information before the COVID-19 pandemic
Please answer to the following background questions concerning your situation BEFORE THE COVID-19 PANDEMIC (circa 18.3.2020)
Main occupation
Employed
Unemployed
Student
Retired
Furloughed
Other
Monthly available household income (after taxation)
How many cars are there in your household?
13.28
30.6.2021

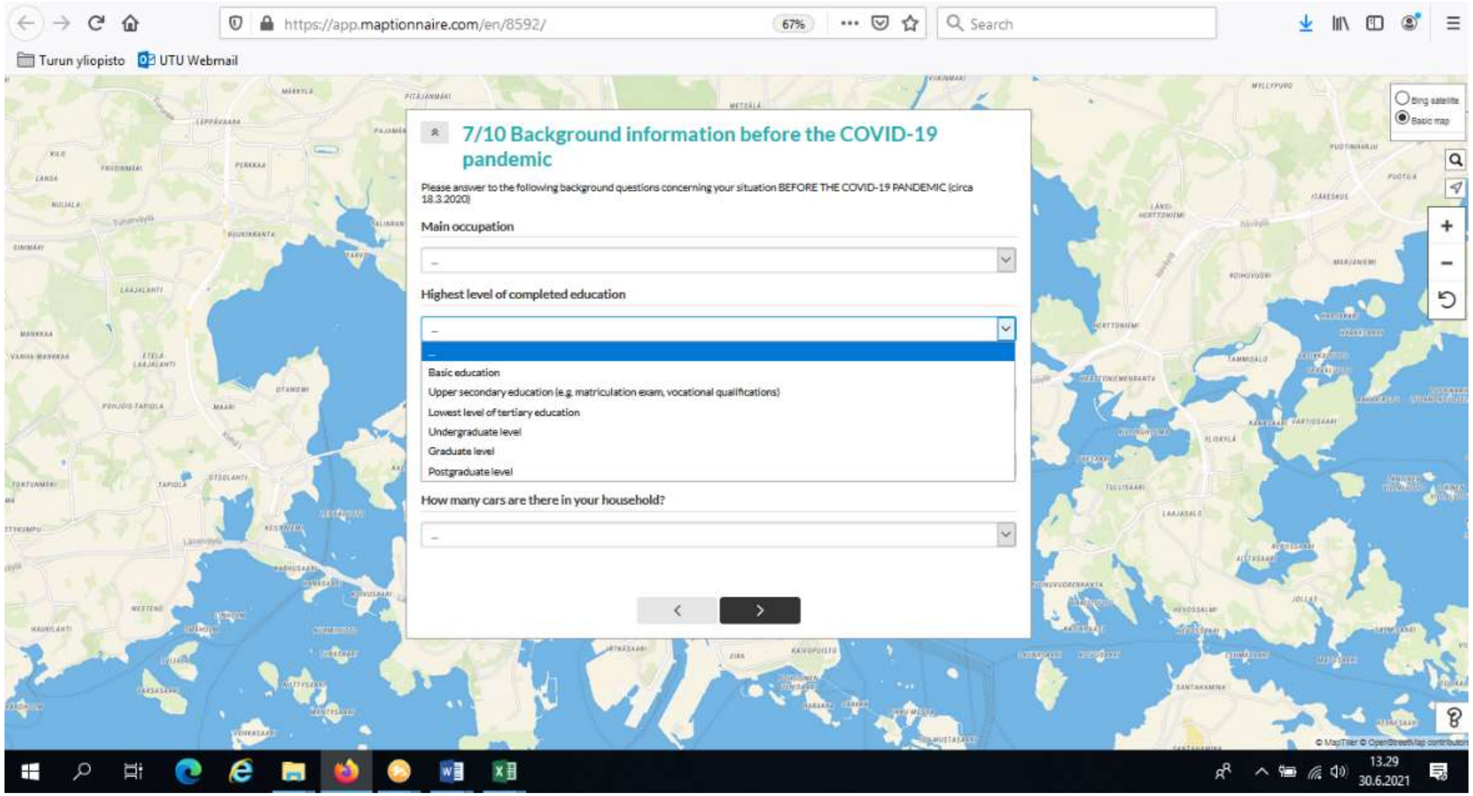
https://app.maptionnaire.com/en/8592/
67%
Search
Turun yliopisto
UTU Webmail
7/10 Background information before the COVID-19 pandemic
Please answer to the following background questions concerning your situation BEFORE THE COVID-19 PANDEMIC (circa 18.3.2020)
Main occupation
Highest level of completed education
Basic education
Upper secondary education (e.g. matriculation exam, vocational qualifications)
Lowest level of tertiary education
Undergraduate level
Graduate level
Postgraduate level
How many cars are there in your household?
Bing satellite
Basic map
13.29
30.6.2021

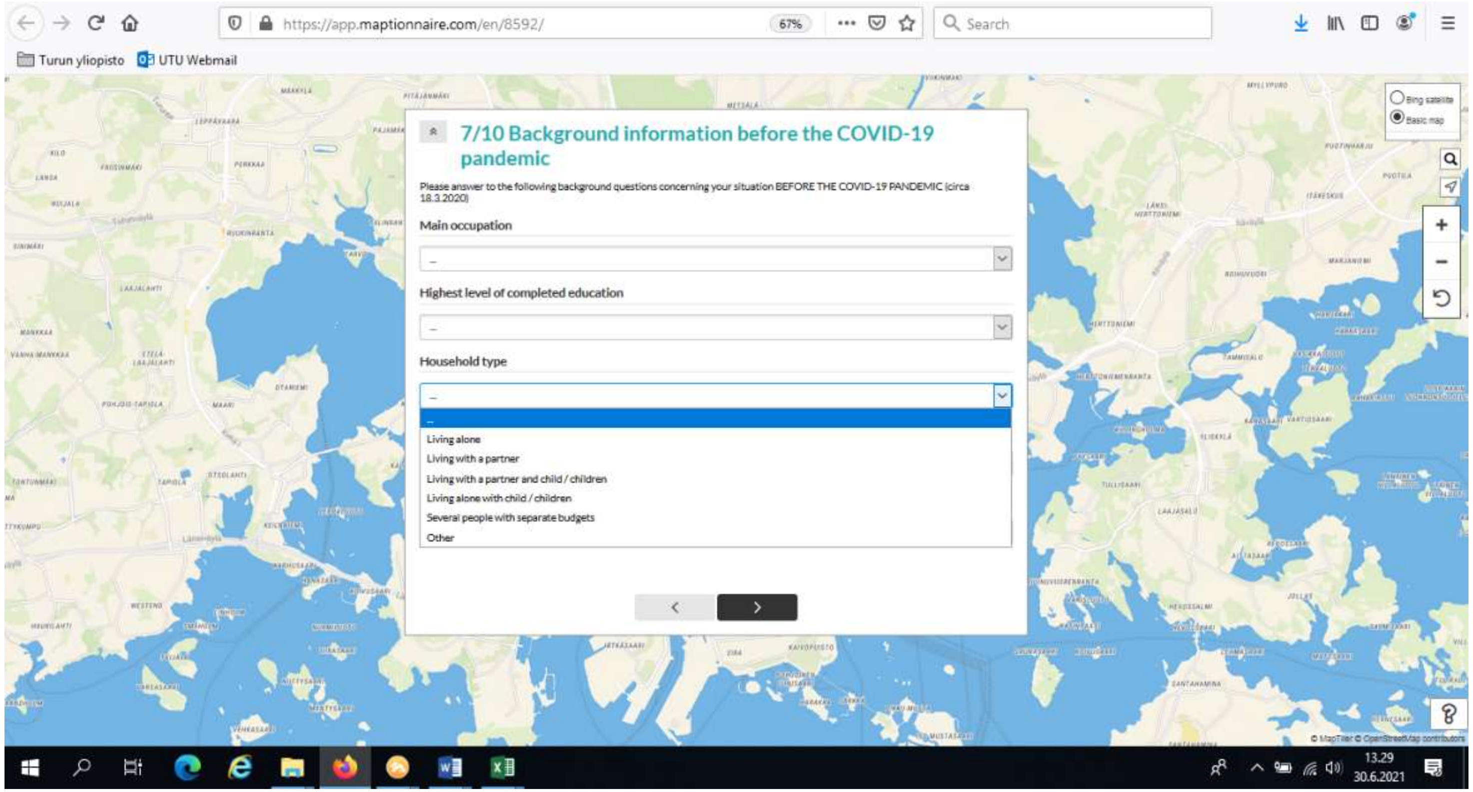
https://app.maptionnaire.com/en/8592/
Turun yliopisto
UTU Webmail
7/10 Background information before the COVID-19 pandemic
Please answer to the following background questions concerning your situation BEFORE THE COVID-19 PANDEMIC (circa 18.3.2020)
Main occupation
Highest level of completed education
Household type
Living alone
Living with a partner
Living with a partner and child / children
Living alone with child / children
Several people with separate budgets
Other
Bing satellite
Basic map
13.29
30.6.2021

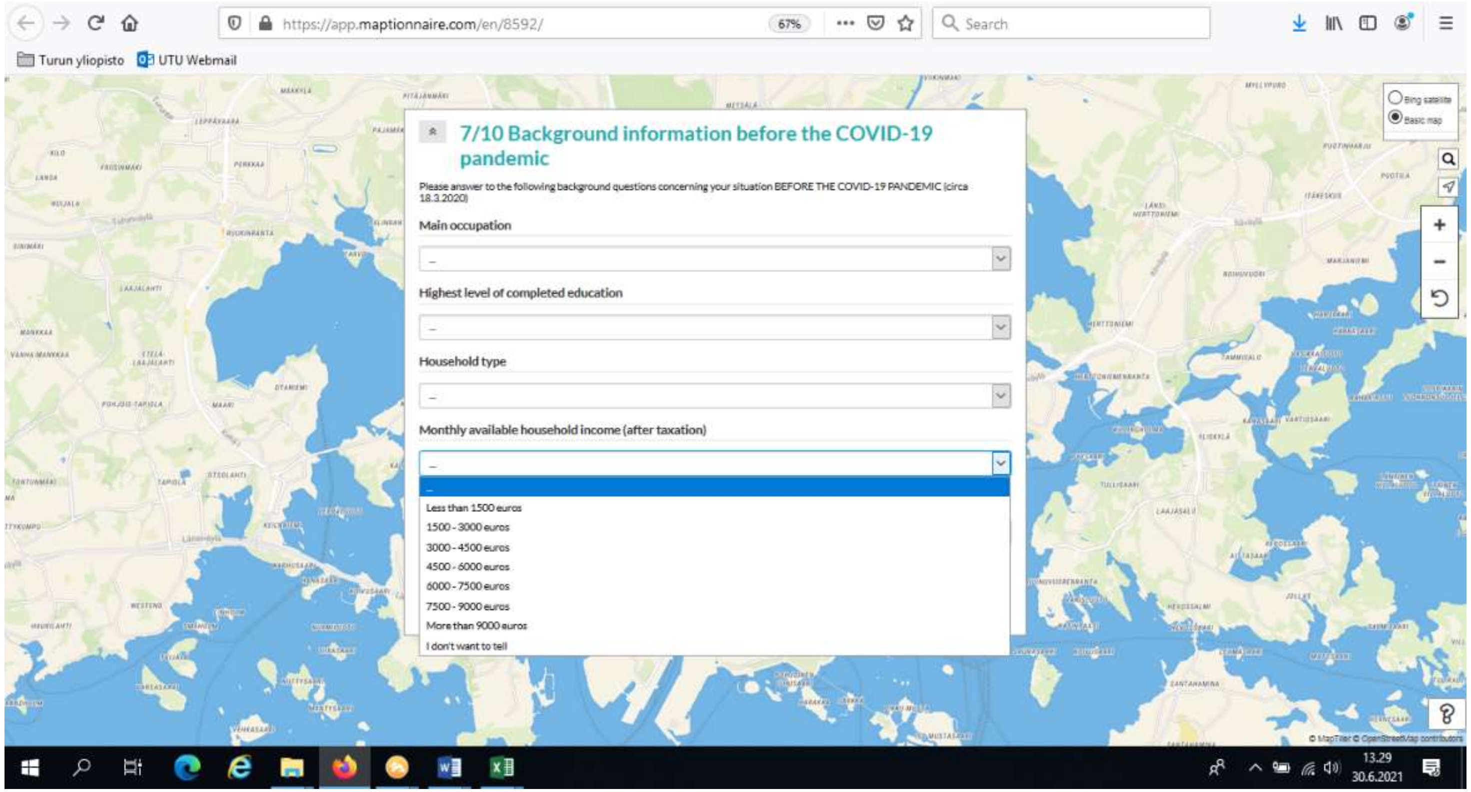
https://app.maptionnaire.com/en/8592/
Turun yliopisto
UTU Webmail
7/10 Background information before the COVID-19 pandemic
Please answer to the following background questions concerning your situation BEFORE THE COVID-19 PANDEMIC (circa 18.3.2020)
Main occupation
Highest level of completed education
Household type
Monthly available household income (after taxation)
Less than 1500 euros
1500 - 3000 euros
3000 - 4500 euros
4500 - 6000 euros
6000 - 7500 euros
7500 - 9000 euros
More than 9000 euros
I don't want to tell
13.29
30.6.2021

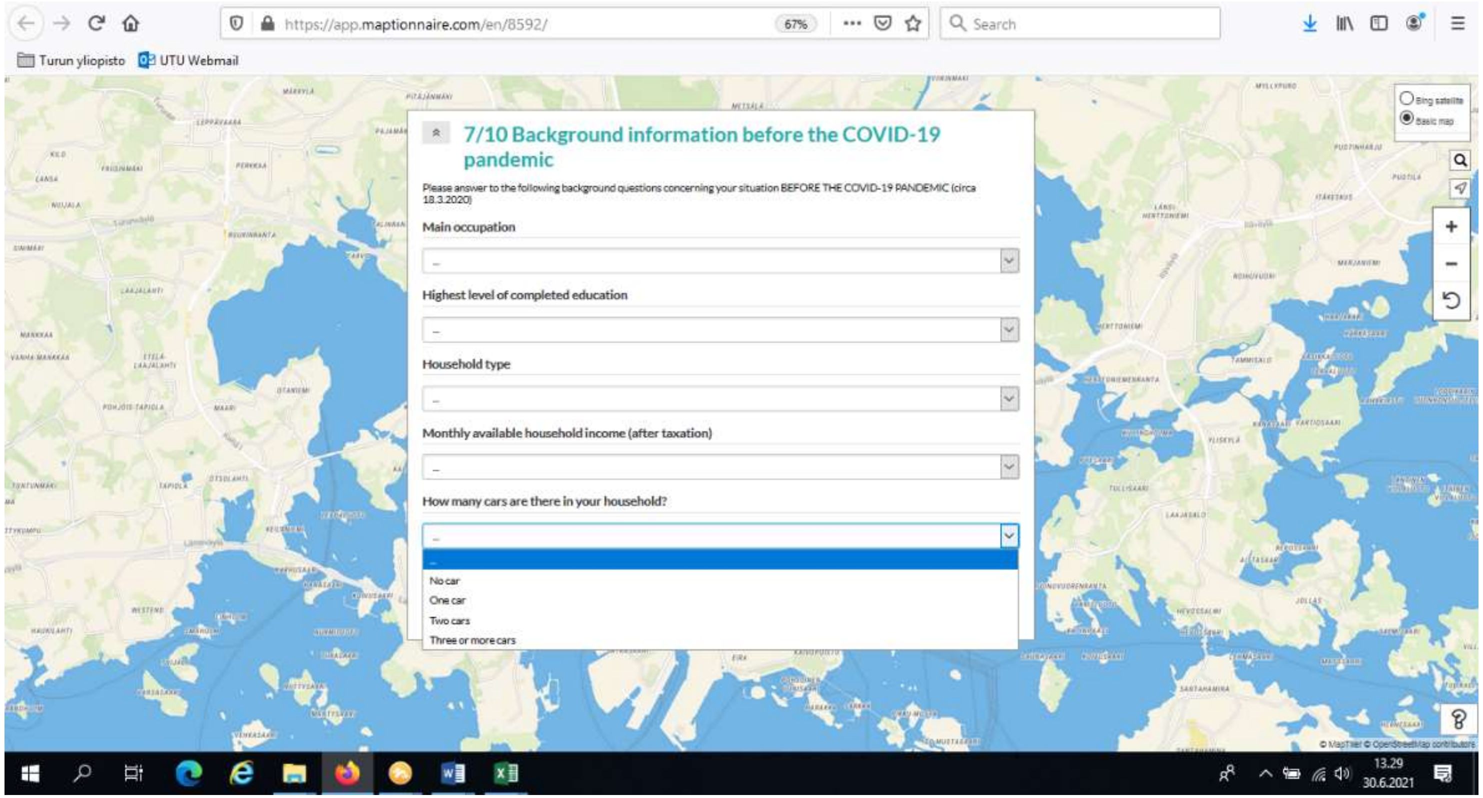
https://app.maptionnaire.com/en/8592/
7/10 Background information before the COVID-19 pandemic
Please answer to the following background questions concerning your situation BEFORE THE COVID-19 PANDEMIC (circa 18.3.2020)
Main occupation
Highest level of completed education
Household type
Monthly available household income (after taxation)
How many cars are there in your household?
No car
One car
Two cars
Three or more cars

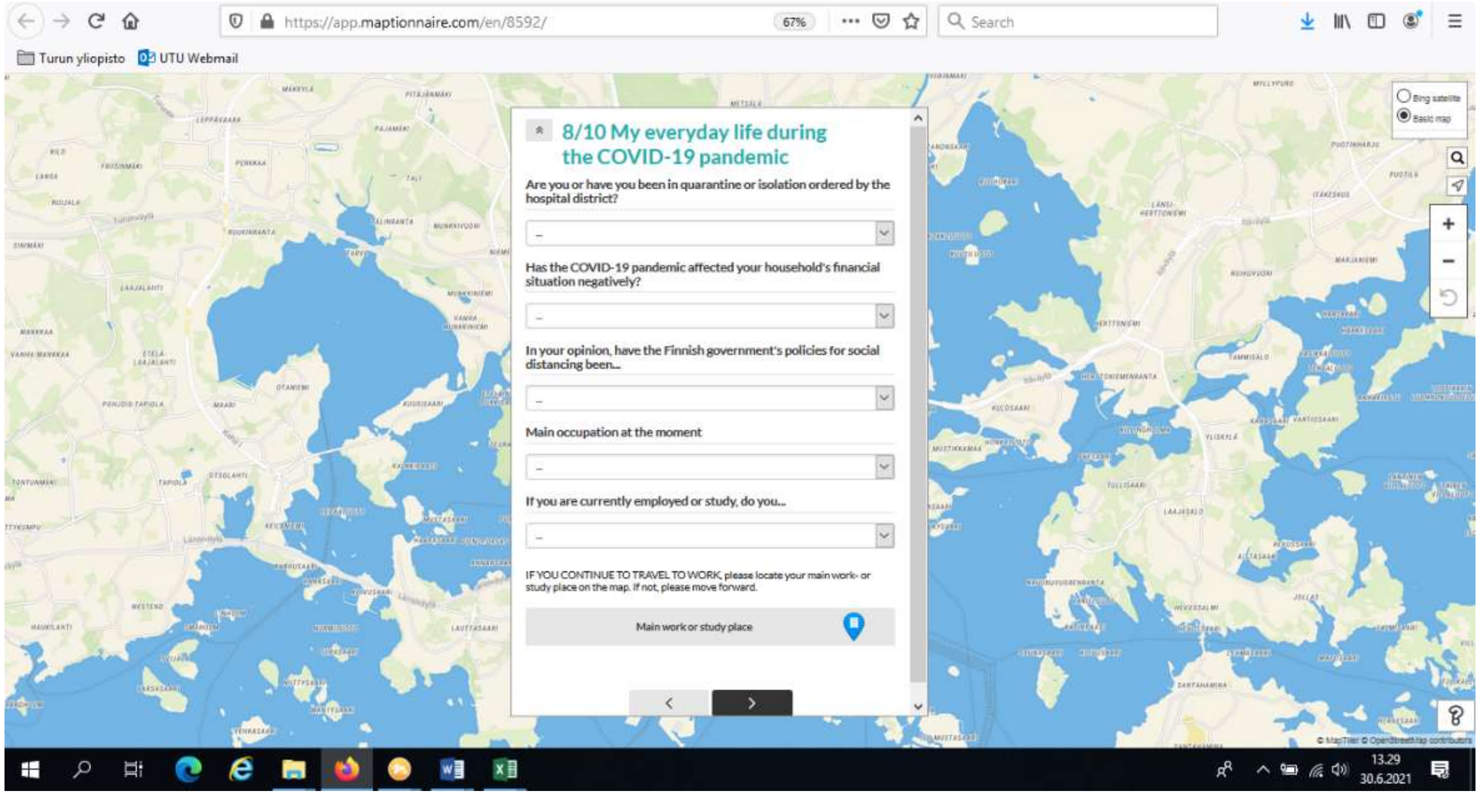
https://app.maptionnaire.com/en/8592/
Turun yliopisto
UTU Webmail
8/10 My everyday life during the COVID-19 pandemic
Are you or have you been in quarantine or isolation ordered by the hospital district?
Has the COVID-19 pandemic affected your household's financial situation negatively?
In your opinion, have the Finnish government's policies for social distancing been...
Main occupation at the moment
If you are currently employed or study, do you...
IF YOU CONTINUE TO TRAVEL TO WORK, please locate your main work- or study place on the map. If not, please move forward.
Main work or study place
Bing satellite
Basic map
13.29
30.6.2021

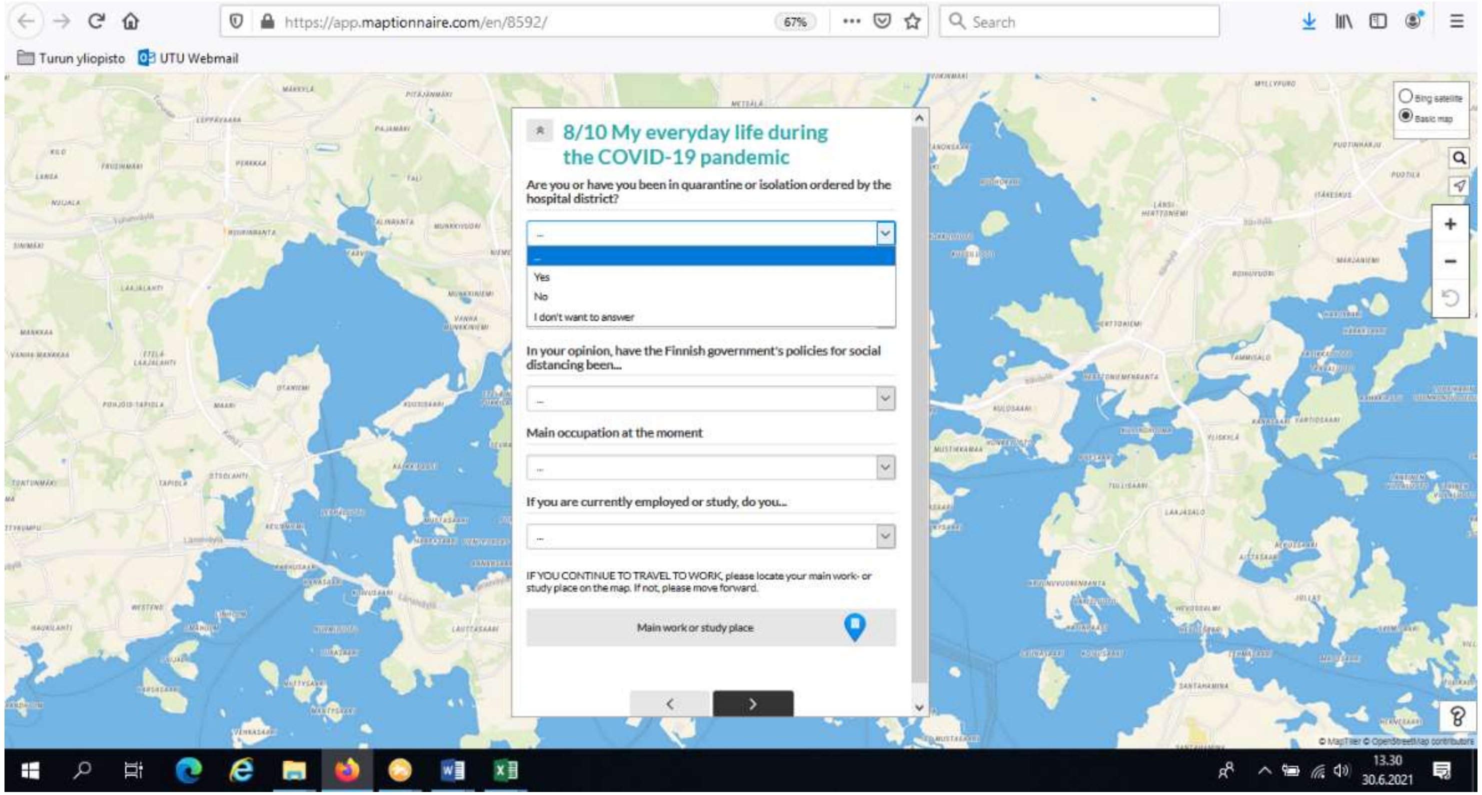

https://app.maptionnaire.com/en/8592/
Turun yliopisto
UTU Webmail
8/10 My everyday life during the COVID-19 pandemic
Are you or have you been in quarantine or isolation ordered by the hospital district?
Yes
No
I don't want to answer
In your opinion, have the Finnish government's policies for social distancing been...
Main occupation at the moment
If you are currently employed or study, do you...
IF YOU CONTINUE TO TRAVEL TO WORK, please locate your main work- or study place on the map. If not, please move forward.
Main work or study place
Bing satellite
Basic map
13.30
30.6.2021

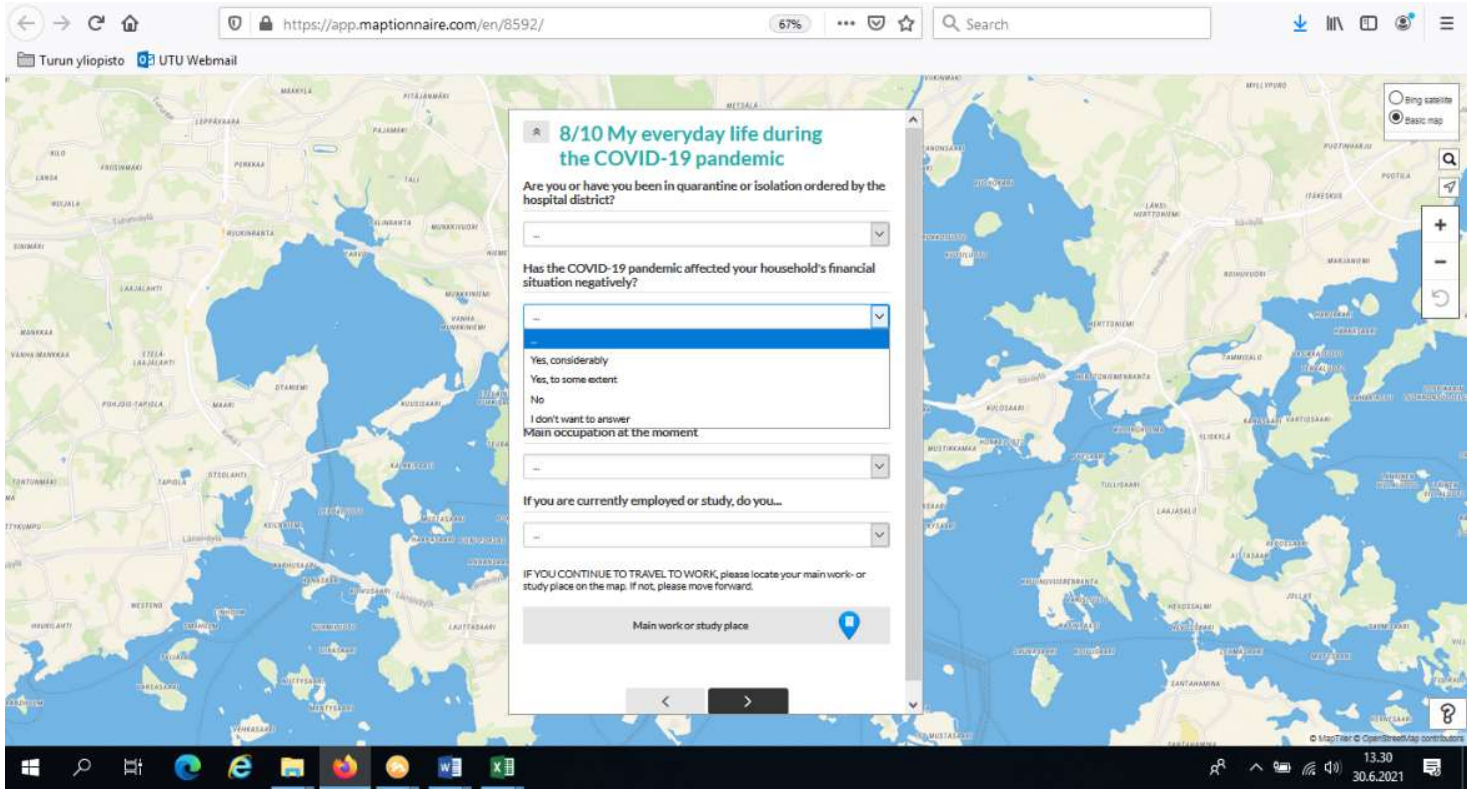
https://app.maptionnaire.com/en/8592/
8/10 My everyday life during the COVID-19 pandemic
Are you or have you been in quarantine or isolation ordered by the hospital district?
Has the COVID-19 pandemic affected your household's financial situation negatively?
Yes, considerably
Yes, to some extent
No
I don't want to answer
Main occupation at the moment
If you are currently employed or study, do you...
IF YOU CONTINUE TO TRAVEL TO WORK, please locate your main work- or study place on the map. If not, please move forward.
Main work or study place
Turun yliopisto
UTU Webmail
Search
13.30
30.6.2021

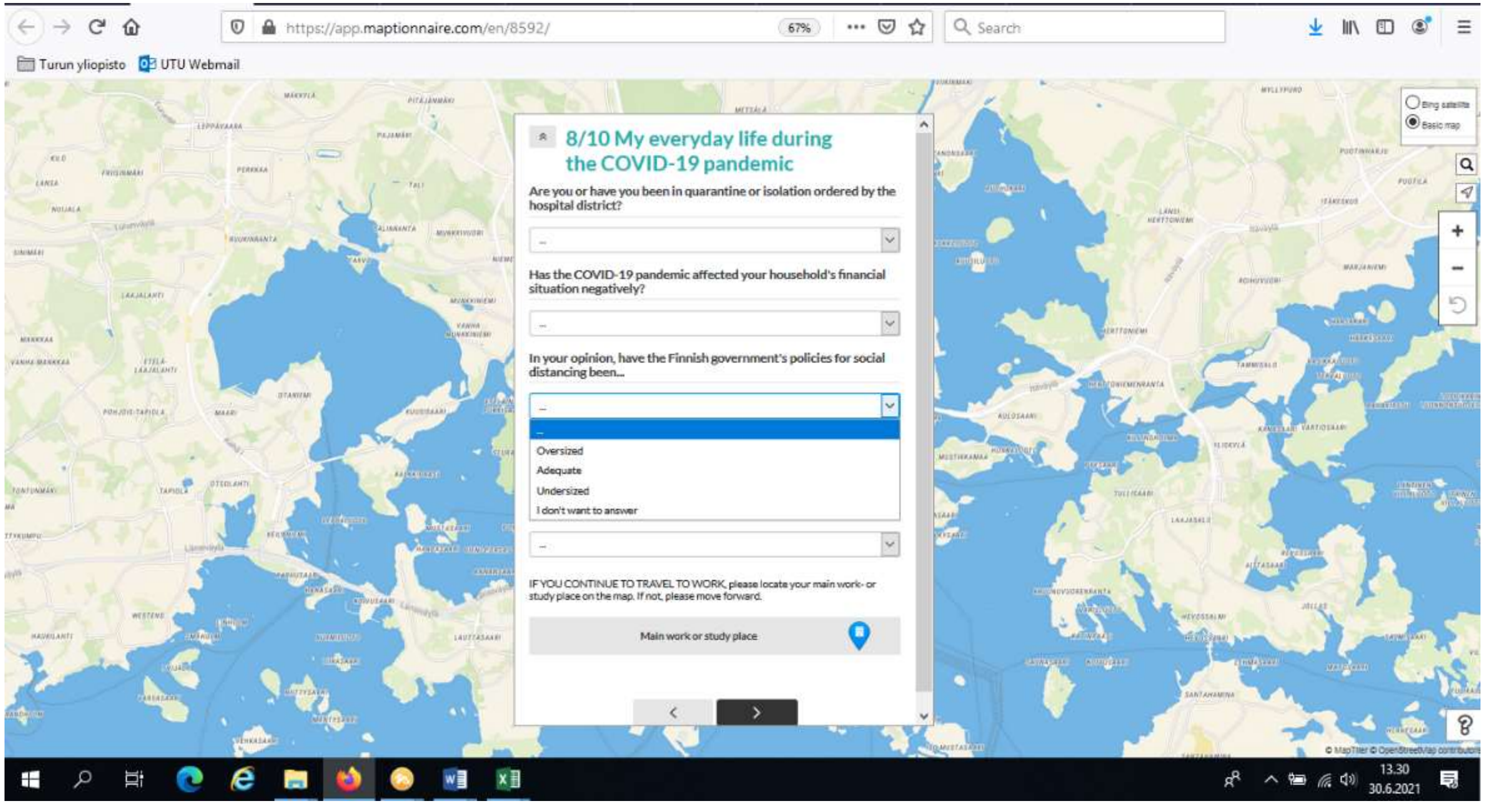
https://app.maptionnaire.com/en/8592/
Turun yliopisto
UTU Webmail
8/10 My everyday life during the COVID-19 pandemic
Are you or have you been in quarantine or isolation ordered by the hospital district?
Has the COVID-19 pandemic affected your household's financial situation negatively?
In your opinion, have the Finnish government's policies for social distancing been...
Oversized
Adequate
Undersized
I don't want to answer
IF YOU CONTINUE TO TRAVEL TO WORK, please locate your main work- or study place on the map. If not, please move forward.
Main work or study place
Bing satellite
Basic map
13.30
30.6.2021

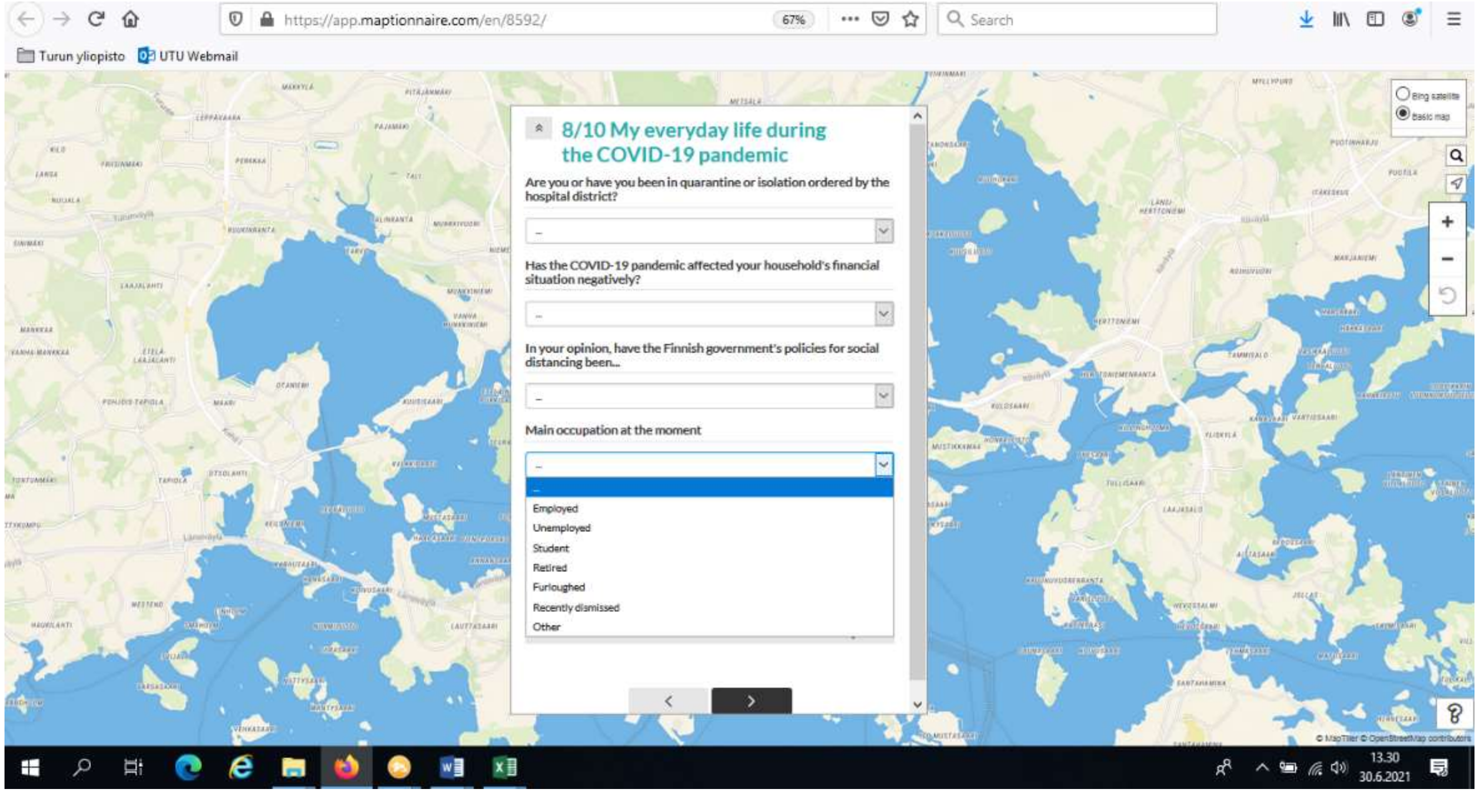
https://app.maptionnaire.com/en/8592/
67%
Search
Turun yliopisto
UTU Webmail
8/10 My everyday life during the COVID-19 pandemic
Are you or have you been in quarantine or isolation ordered by the hospital district?
Has the COVID-19 pandemic affected your household's financial situation negatively?
In your opinion, have the Finnish government's policies for social distancing been...
Main occupation at the moment
Employed
Unemployed
Student
Retired
Furloughed
Recently dismissed
Other
13.30
30.6.2021

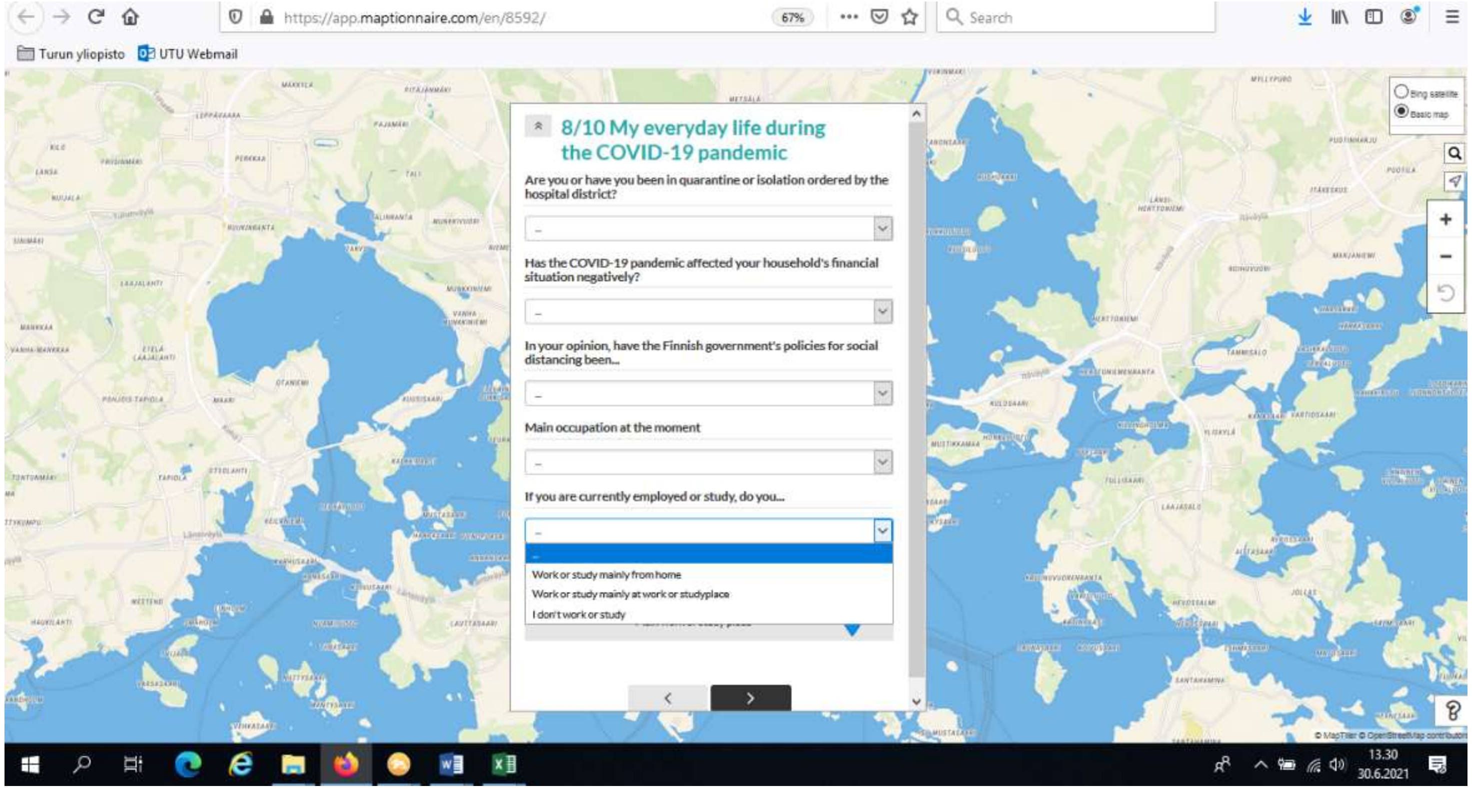
https://app.maptionnaire.com/en/8592/
Search
Turun yliopisto
UTU Webmail
Bing satellite
Basic map
8/10 My everyday life during the COVID-19 pandemic
Are you or have you been in quarantine or isolation ordered by the hospital district?
Has the COVID-19 pandemic affected your household's financial situation negatively?
In your opinion, have the Finnish government's policies for social distancing been...
Main occupation at the moment
If you are currently employed or study, do you...
Work or study mainly from home
Work or study mainly at work or studyplace
I don't work or study
13.30
30.6.2021

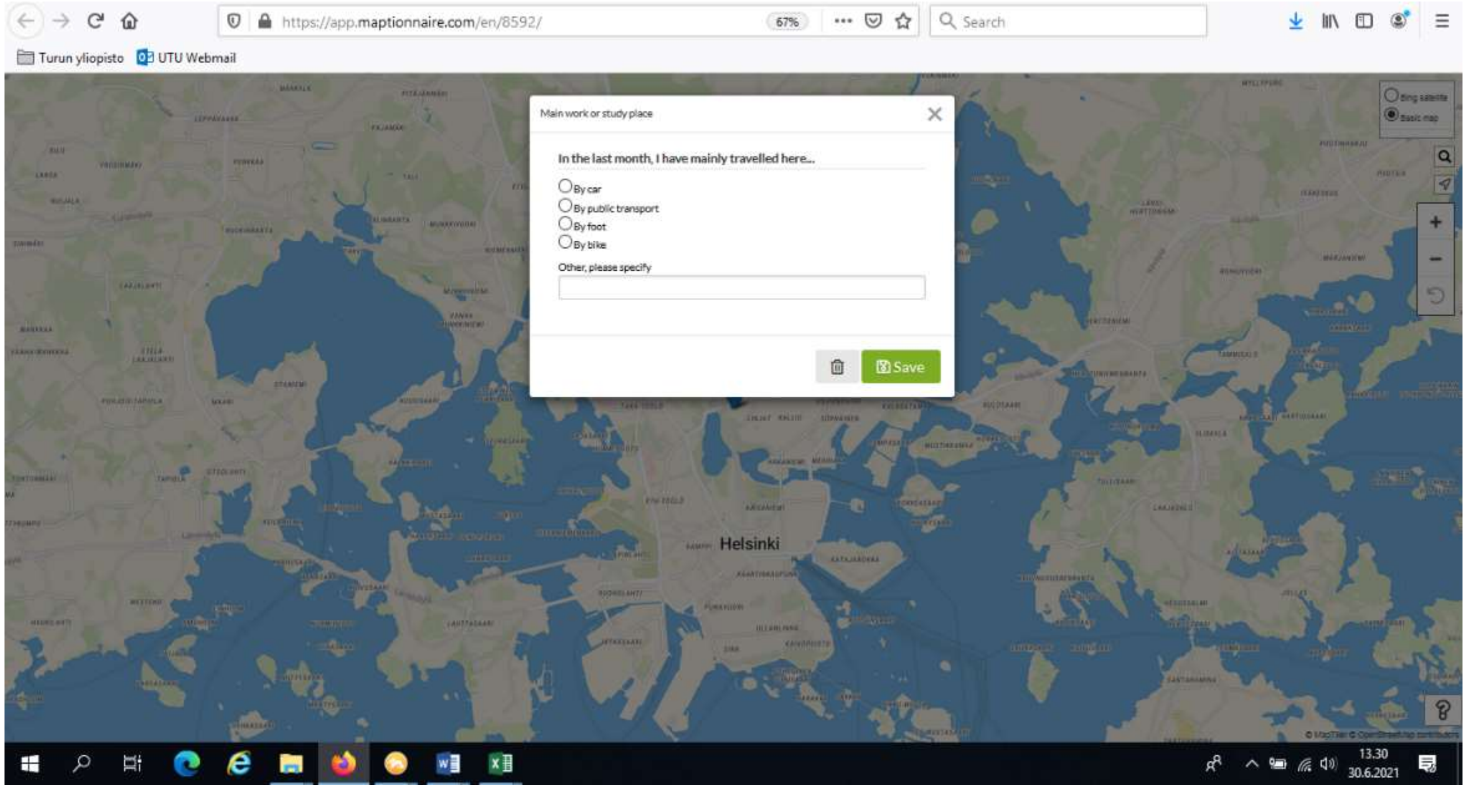

https://app.maptionnaire.com/en/8592/
Search
Turun yliopisto
UTU Webmail
Main work or study place
In the last month, I have mainly travelled here...
By car
By public transport
By foot
By bike
Other, please specify
Save
Helsinki
13.30
30.6.2021

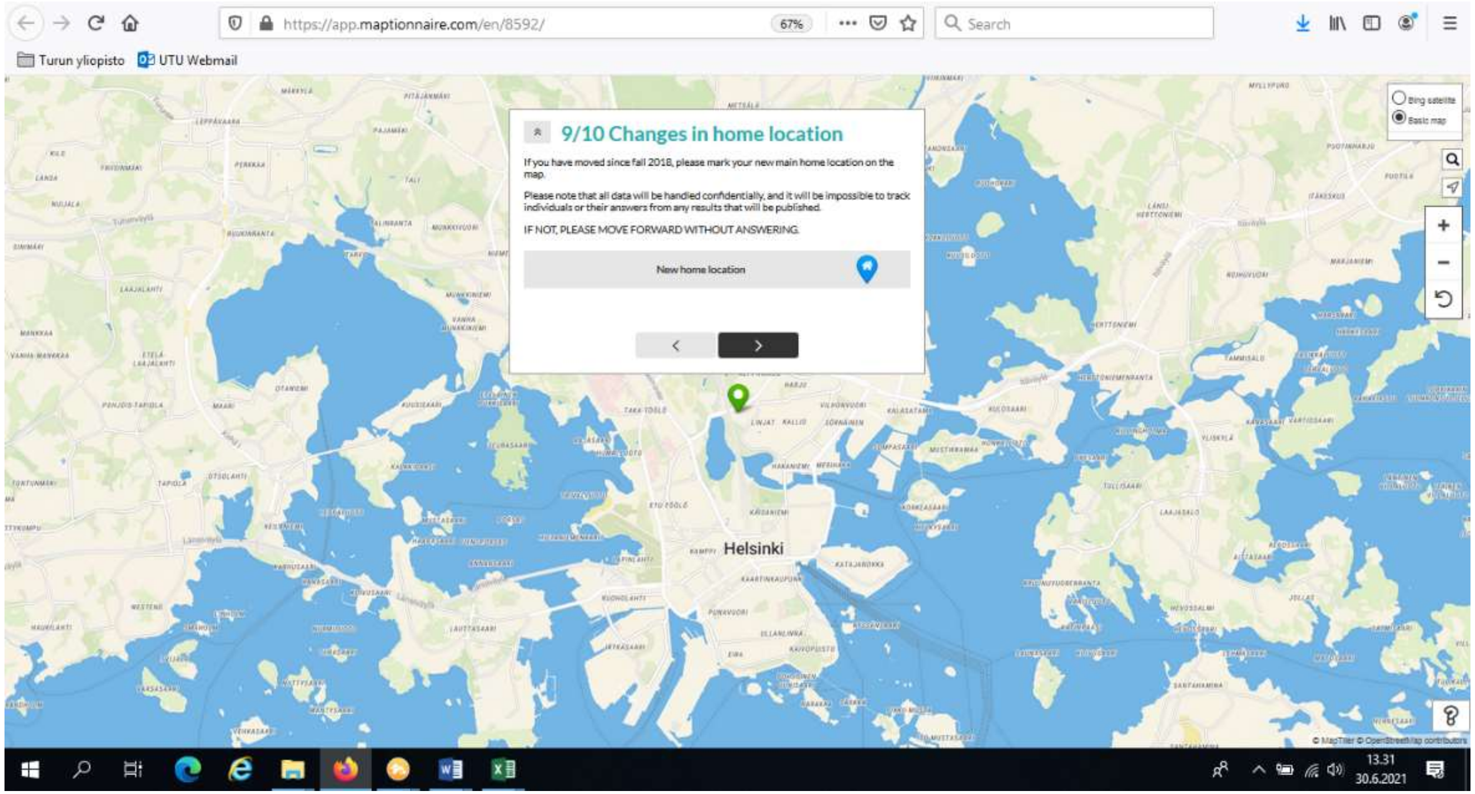
https://app.maptionnaire.com/en/8592/
Turun yliopisto
UTU Webmail
9/10 Changes in home location
If you have moved since fall 2018, please mark your new main home location on the map.
Please note that all data will be handled confidentially, and it will be impossible to track individuals or their answers from any results that will be published.
IF NOT, PLEASE MOVE FORWARD WITHOUT ANSWERING.
New home location
Helsinki
13.31
30.6.2021

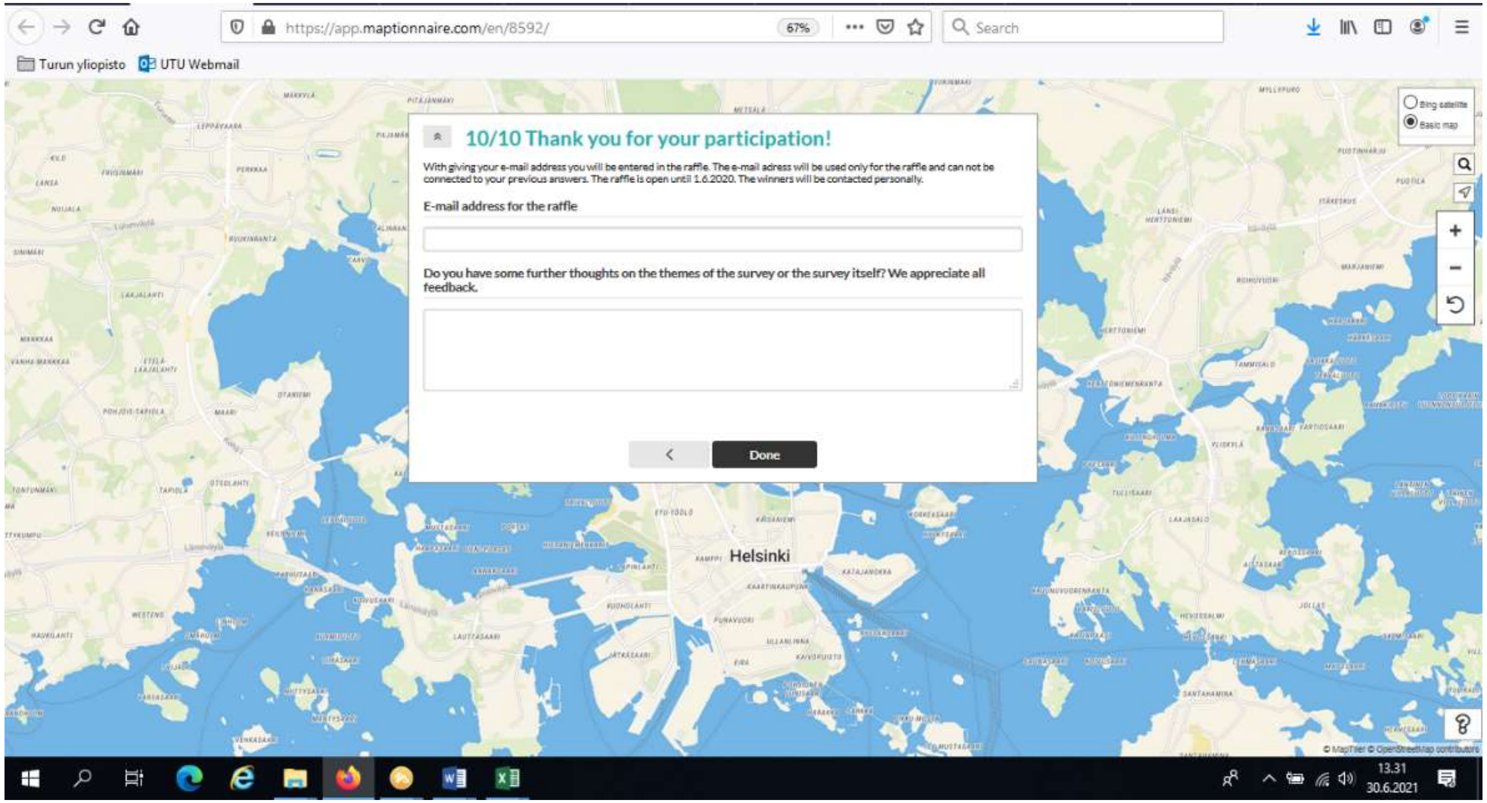

Outdoor activities in Turku

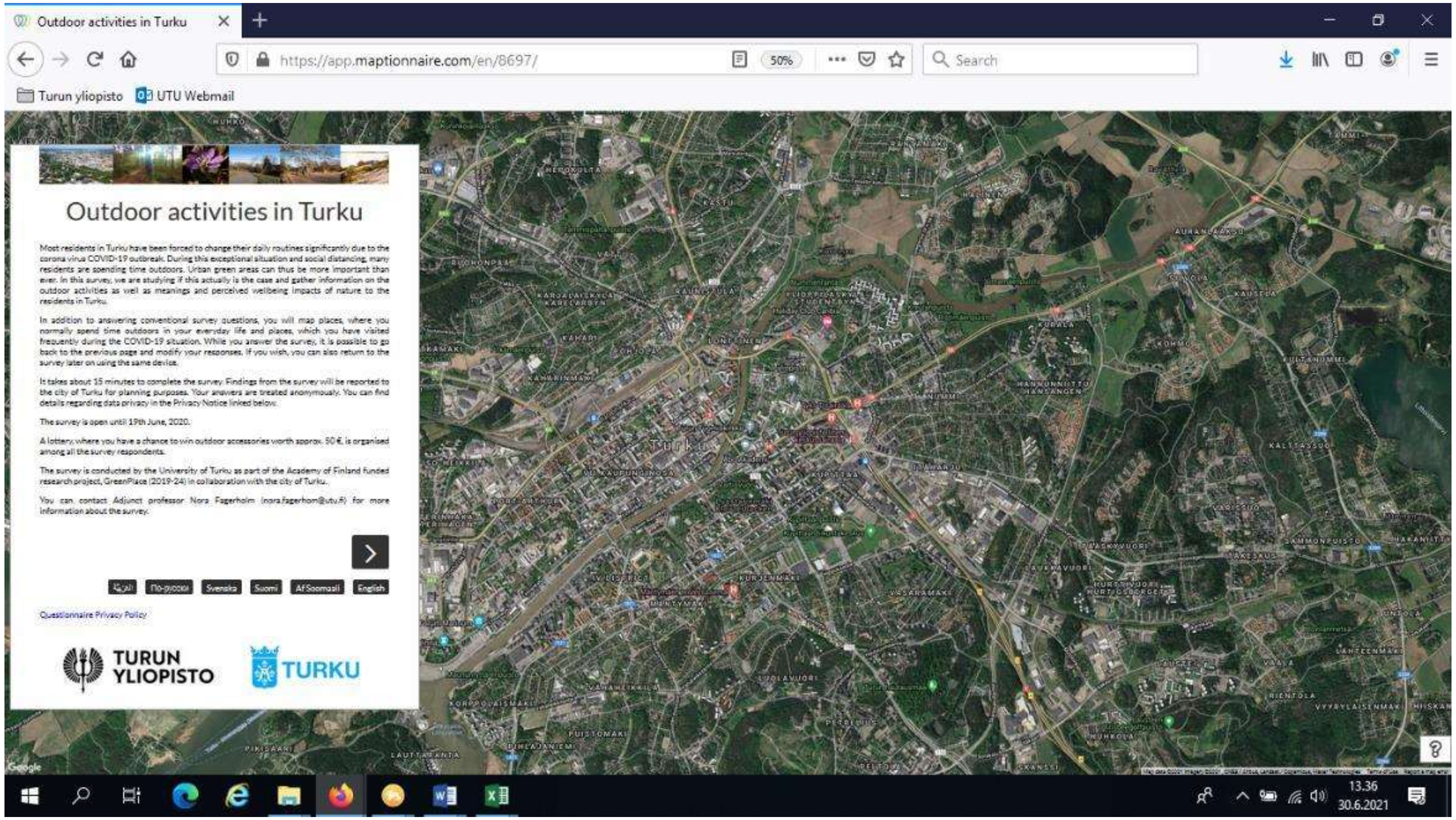
Outdoor activities in Turku
https://app.maptionnaire.com/en/8697/
Turun yliopisto
UTU Webmail
Outdoor activities in Turku
Most residents in Turku have been forced to change their daily routines significantly due to the corona virus COVID-19 outbreak. During this exceptional situation and social distancing, many residents are spending time outdoors. Urban green areas can thus be more important than ever. In this survey, we are studying if this actually is the case and gather information on the outdoor activities as well as meanings and perceived wellbeing impacts of nature to the residents in Turku.
In addition to answering conventional survey questions, you will map places, where you normally spend time outdoors in your everyday life and places, which you have visited frequently during the COVID-19 situation. While you answer the survey, it is possible to go back to the previous page and modify your responses. If you wish, you can also return to the survey later on using the same device.
It takes about 15 minutes to complete the survey. Findings from the survey will be reported to the city of Turku for planning purposes. Your answers are treated anonymously. You can find details regarding data privacy in the Privacy Notice linked below.
The survey is open until 19th June, 2020.
A lottery, where you have a chance to win outdoor accessories worth approx. 50 €, is organised among all the survey respondents.
The survey is conducted by the University of Turku as part of the Academy of Finland funded research project, GreenPlace (2019-24) in collaboration with the city of Turku.
You can contact Adjunct professor Nora Fagerholm (nora.fagerhom@utu.fi) for more information about the survey.
العربية
По-русски
Svenska
Suomi
Af Soomaali
English
Questionnaire Privacy Policy
TURUN YLIOPISTO
TURKU
13.36
30.6.2021

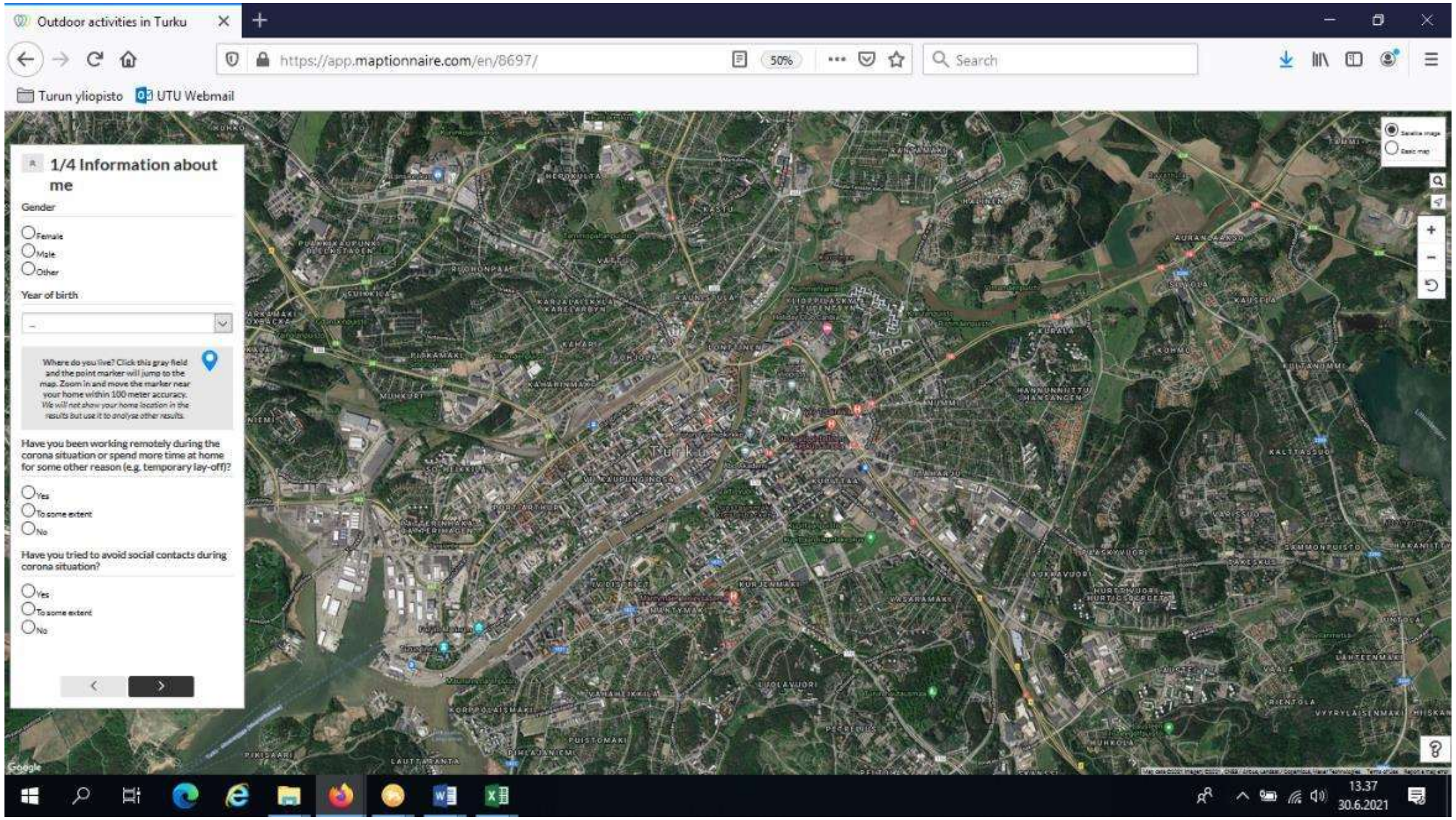

Outdoor activities in Turku
https://app.maptionnaire.com/en/8697/
50%
Search
Turun yliopisto
UTU Webmail
1/4 Information about me
Gender
Female
Male
Other
Year of birth
Where do you live? Click this gray field and the point marker will jump to the map. Zoom in and move the marker near your home within 100 meter accuracy. We will not show your home location in the results but use it to analyse other results.
Have you been working remotely during the corona situation or spend more time at home for some other reason (e.g. temporary lay-off)?
Yes
To some extent
No
Have you tried to avoid social contacts during corona situation?
Yes
To some extent
No
Turku
Google
13.37
30.6.2021

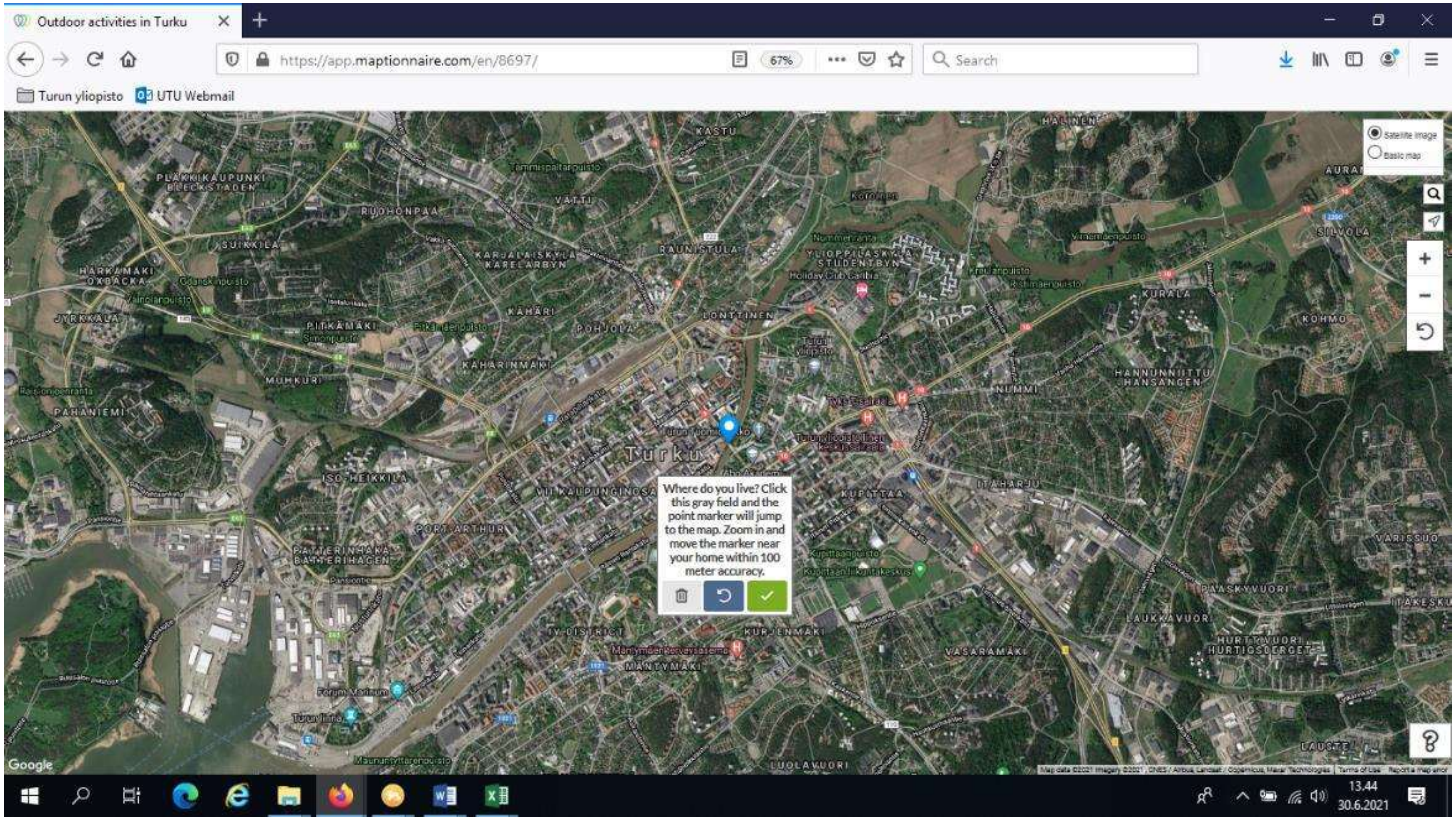
Outdoor activities in Turku
https://app.maptionnaire.com/en/8697/
67%
Search
Turun yliopisto
UTU Webmail
Turku
Where do you live? Click this gray field and the point marker will jump to the map. Zoom in and move the marker near your home within 100 meter accuracy.
Google
13.44
30.6.2021

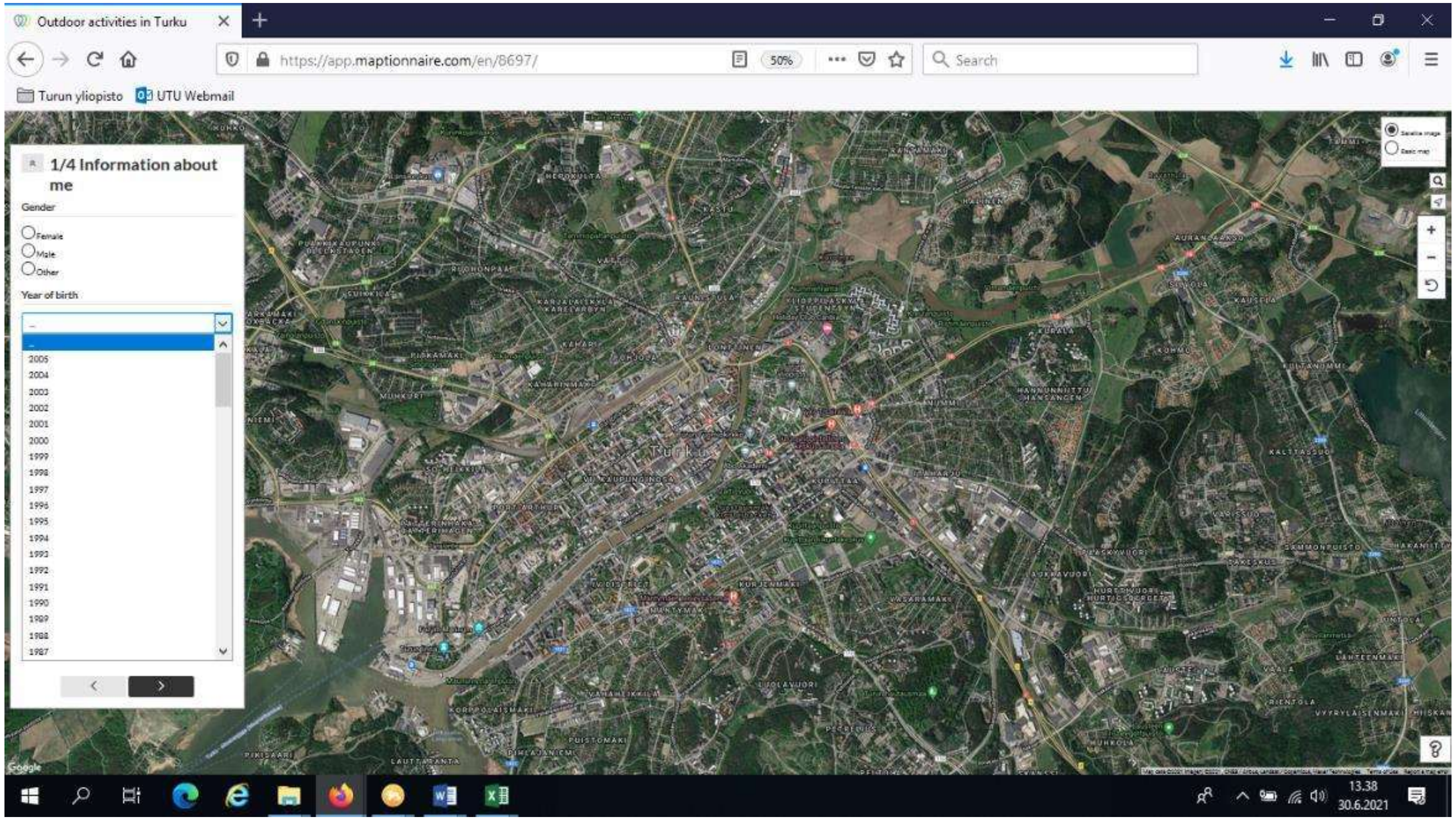
Outdoor activities in Turku
https://app.maptionnaire.com/en/8697/
Turun yliopisto
UTU Webmail
1/4 Information about me
Gender
Female
Male
Other
Year of birth
2005
2004
2003
2002
2001
2000
1999
1998
1997
1996
1995
1994
1993
1992
1991
1990
1989
1988
1987
13.38
30.6.2021

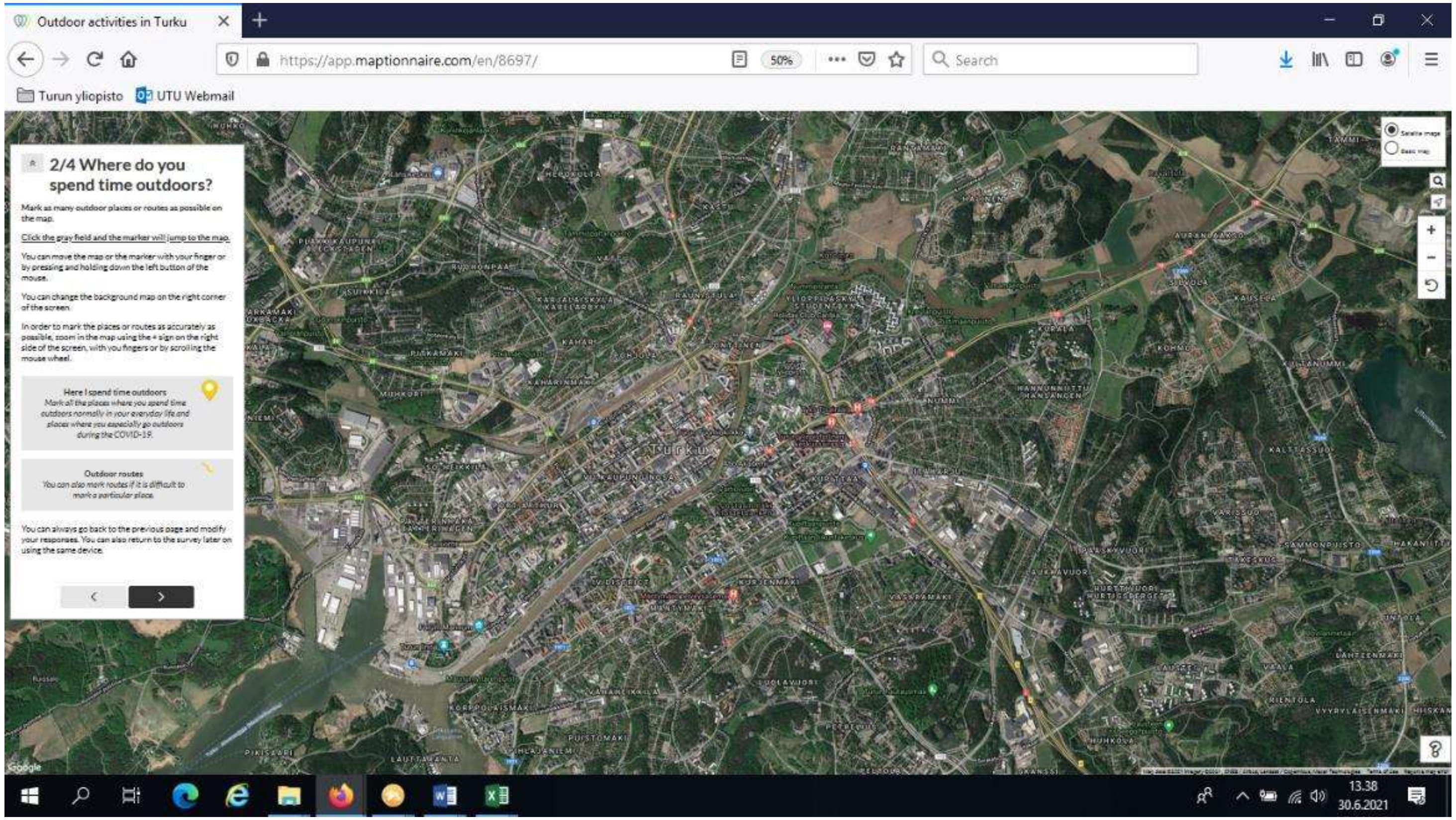
Outdoor activities in Turku
https://app.maptionnaire.com/en/8697/
Search
Turun yliopisto
UTU Webmail
2/4 Where do you spend time outdoors?
Mark as many outdoor places or routes as possible on the map.
Click the gray field and the marker will jump to the map.
You can move the map or the marker with your finger or by pressing and holding down the left button of the mouse.
You can change the background map on the right corner of the screen.
In order to mark the places or routes as accurately as possible, zoom in the map using the + sign on the right side of the screen, with you fingers or by scrolling the mouse wheel.
Here I spend time outdoors
Mark all the places where you spend time outdoors normally in your everyday life and places where you especially go outdoors during the COVID-19.
Outdoor routes
You can also mark routes if it is difficult to mark a particular place.
You can always go back to the previous page and modify your responses. You can also return to the survey later on using the same device.
Turku
13.38
30.6.2021

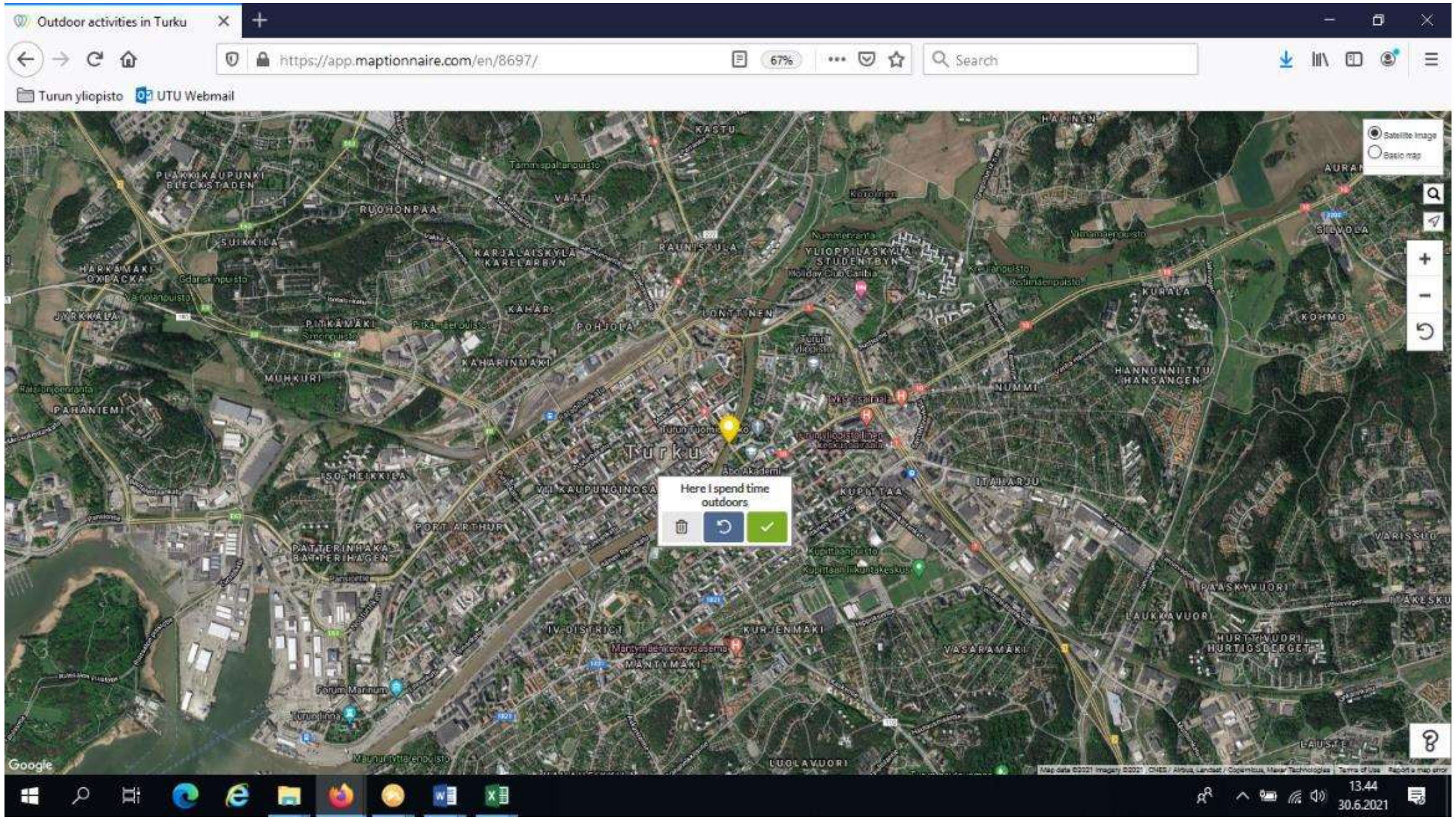
Outdoor activities in Turku
https://app.maptionnaire.com/en/8697/
Turun yliopisto
UTU Webmail
Satellite image
Basic map
Here I spend time outdoors
Turku
Google
13.44
30.6.2021

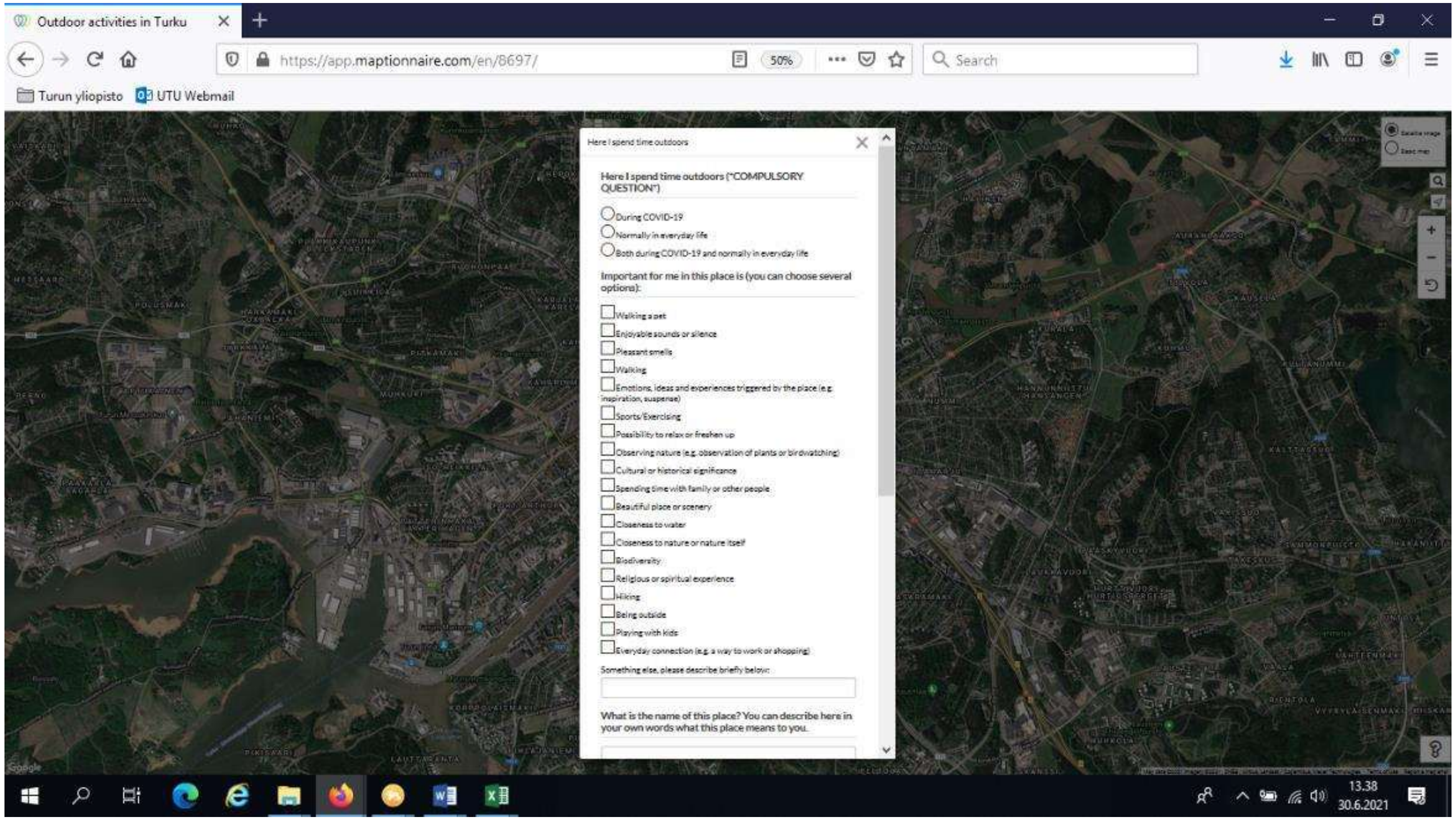
Outdoor activities in Turku
https://app.maptionnaire.com/en/8697/
Search
Turun yliopisto
UTU Webmail
Here I spend time outdoors
Here I spend time outdoors (*COMPULSORY QUESTION*)
During COVID-19
Normally in everyday life
Both during COVID-19 and normally in everyday life
Important for me in this place is (you can choose several options):
Walking a pet
Enjoyable sounds or silence
Pleasant smells
Walking
Emotions, ideas and experiences triggered by the place (e.g. inspiration, suspense)
Sports/Exercising
Possibility to relax or freshen up
Observing nature (e.g. observation of plants or birdwatching)
Cultural or historical significance
Spending time with family or other people
Beautiful place or scenery
Closeness to water
Closeness to nature or nature itself
Biodiversity
Religious or spiritual experience
Hiking
Being outside
Playing with kids
Everyday connection (e.g. a way to work or shopping)
Something else, please describe briefly below:
What is the name of this place? You can describe here in your own words what this place means to you.
13.38
30.6.2021

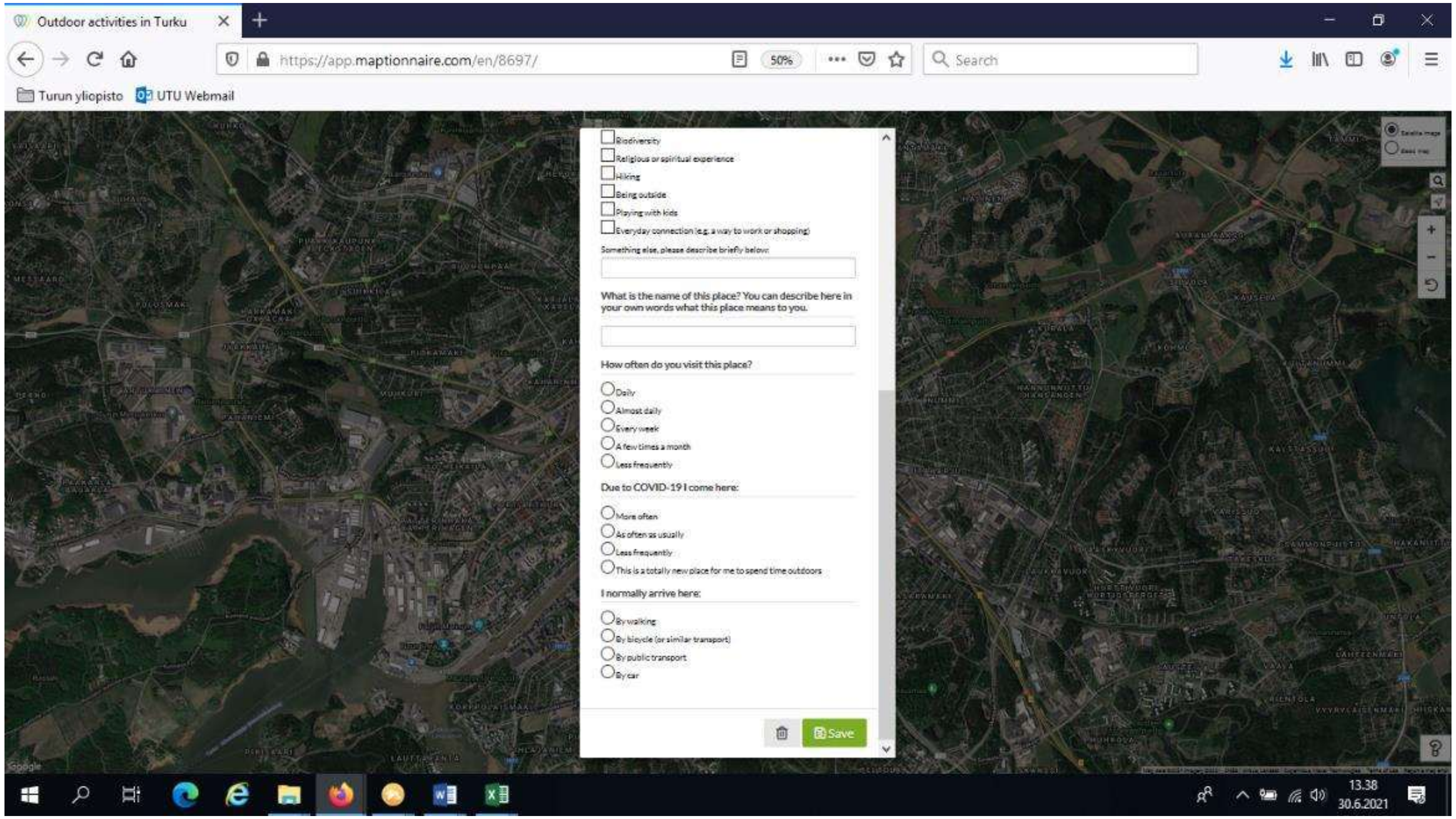

Outdoor activities in Turku
https://app.maptionnaire.com/en/8697/
50%
Search
Turun yliopisto
UTU Webmail
Biodiversity
Religious or spiritual experience
Hiking
Being outside
Playing with kids
Everyday connection (e.g. a way to work or shopping)
Something else, please describe briefly below:
What is the name of this place? You can describe here in your own words what this place means to you.
How often do you visit this place?
Daily
Almost daily
Every week
A few times a month
Less frequently
Due to COVID-19 I come here:
More often
As often as usually
Less frequently
This is a totally new place for me to spend time outdoors
I normally arrive here:
By walking
By bicycle (or similar transport)
By public transport
By car
Save
13.38
30.6.2021

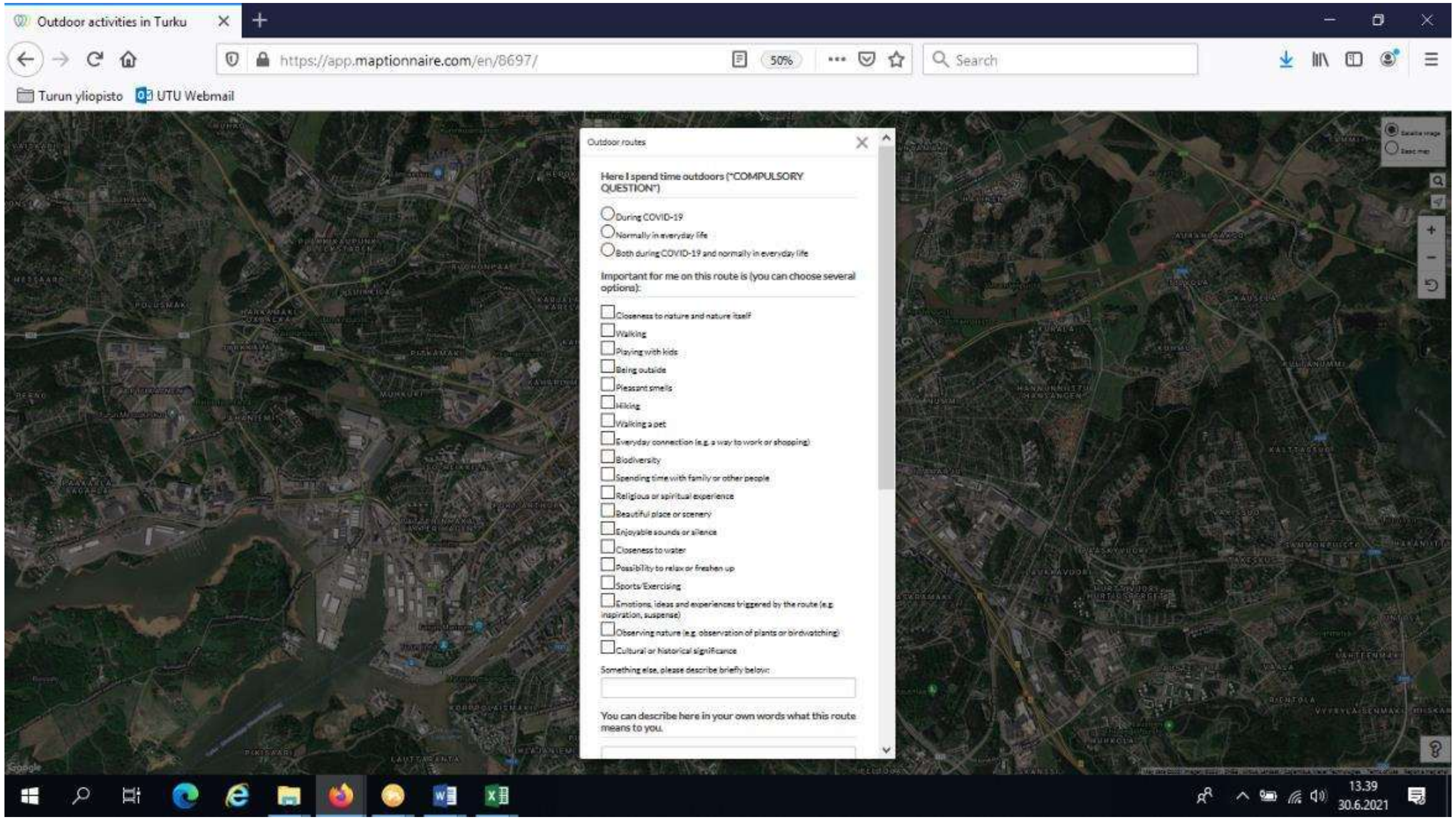
Outdoor activities in Turku
https://app.maptionnaire.com/en/8697/
50%
Search
Turun yliopisto
UTU Webmail
Outdoor routes
Here I spend time outdoors (*COMPULSORY QUESTION*)
During COVID-19
Normally in everyday life
Both during COVID-19 and normally in everyday life
Important for me on this route is (you can choose several options):
Closeness to nature and nature itself
Walking
Playing with kids
Being outside
Pleasant smells
Hiking
Walking a pet
Everyday connection (e.g. a way to work or shopping)
Biodiversity
Spending time with family or other people
Religious or spiritual experience
Beautiful place or scenery
Enjoyable sounds or silence
Closeness to water
Possibility to relax or freshen up
Sports/Exercising
Emotions, ideas and experiences triggered by the route (e.g. inspiration, suspense)
Observing nature (e.g. observation of plants or birdwatching)
Cultural or historical significance
Something else, please describe briefly below:
You can describe here in your own words what this route means to you.
13.39
30.6.2021

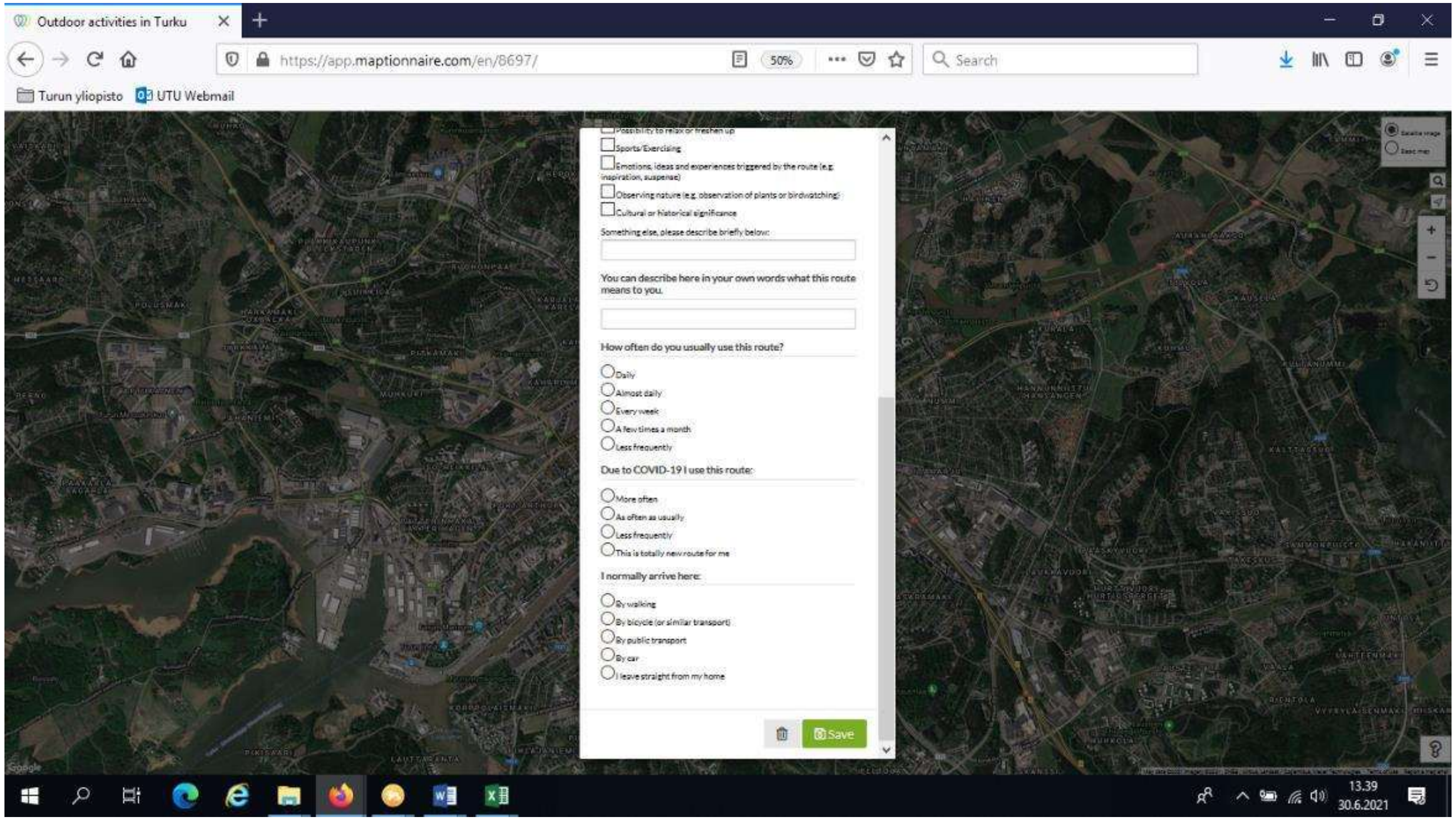

Outdoor activities in Turku
https://app.maptionnaire.com/en/8697/
50%
Search
Turun yliopisto
UTU Webmail
Possibility to relax or freshen up
Sports/Exercising
Emotions, ideas and experiences triggered by the route (e.g. inspiration, suspense)
Observing nature (e.g. observation of plants or birdwatching)
Cultural or historical significance
Something else, please describe briefly below:
You can describe here in your own words what this route means to you.
How often do you usually use this route?
Daily
Almost daily
Every week
A few times a month
Less frequently
Due to COVID-19 I use this route:
More often
As often as usually
Less frequently
This is totally new route for me
I normally arrive here:
By walking
By bicycle (or similar transport)
By public transport
By car
I leave straight from my home
Save
13.39
30.6.2021

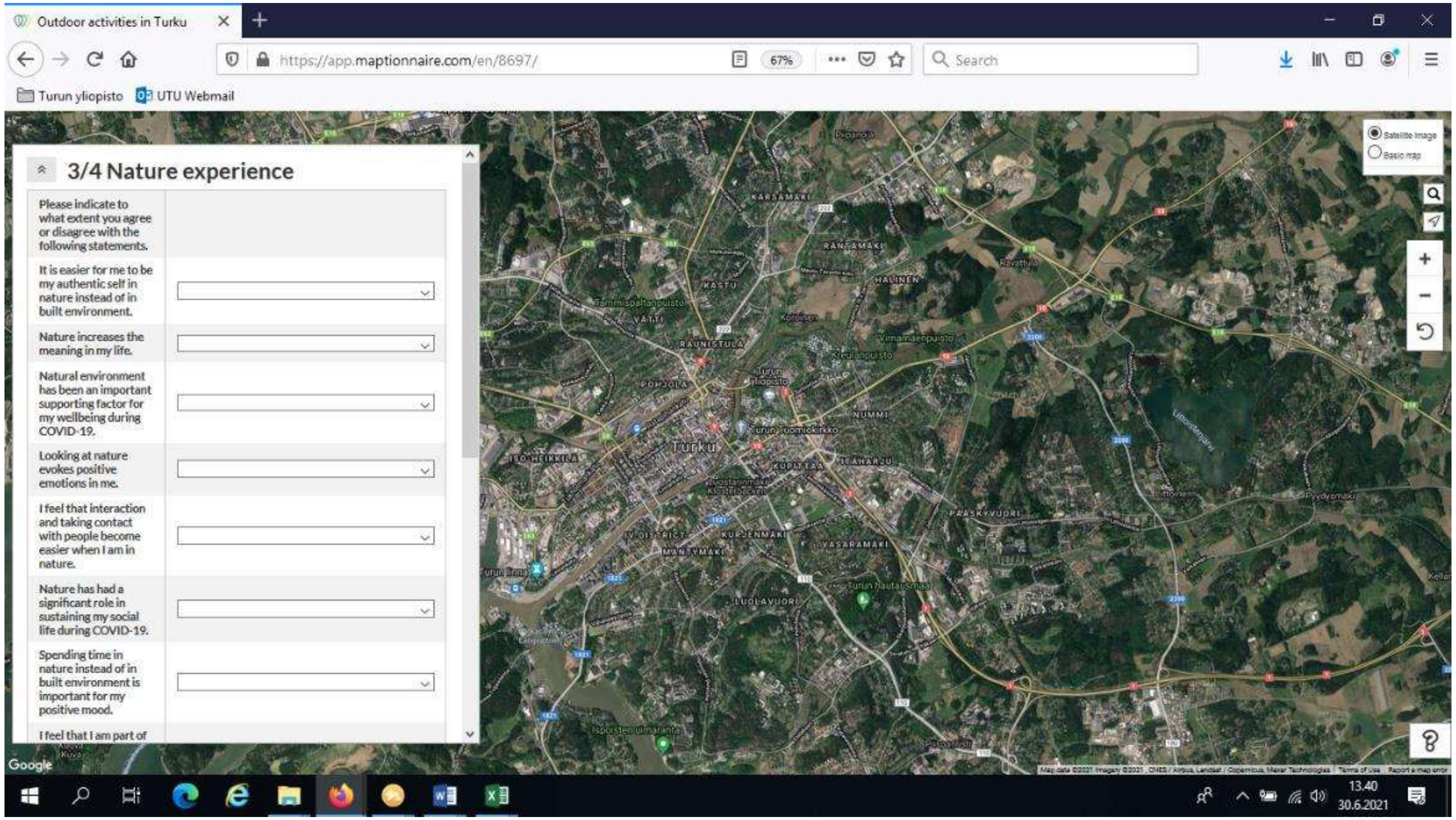
Outdoor activities in Turku
https://app.maptionnaire.com/en/8697/
Search
Turun yliopisto
UTU Webmail
3/4 Nature experience
Please indicate to what extent you agree or disagree with the following statements.
It is easier for me to be my authentic self in nature instead of in built environment.
Nature increases the meaning in my life.
Natural environment has been an important supporting factor for my wellbeing during COVID-19.
Looking at nature evokes positive emotions in me.
I feel that interaction and taking contact with people become easier when I am in nature.
Nature has had a significant role in sustaining my social life during COVID-19.
Spending time in nature instead of in built environment is important for my positive mood.
I feel that I am part of
Satellite image
Basic map
Turku
Google
13.40
30.6.2021

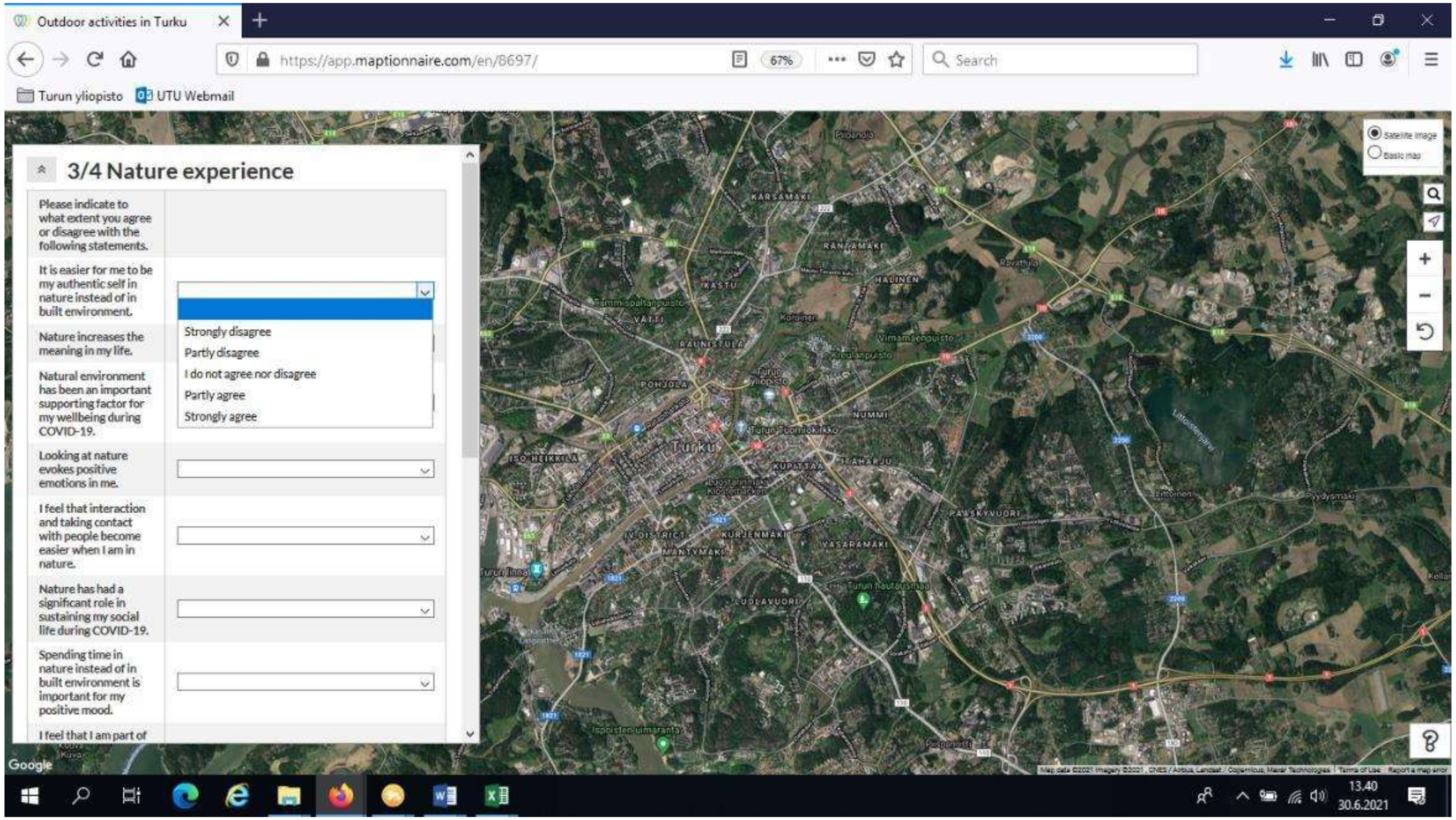
Outdoor activities in Turku
https://app.maptionnaire.com/en/8697/
Turun yliopisto
UTU Webmail
3/4 Nature experience
Please indicate to what extent you agree or disagree with the following statements.
It is easier for me to be my authentic self in nature instead of in built environment.
Strongly disagree
Partly disagree
I do not agree nor disagree
Partly agree
Strongly agree
Nature increases the meaning in my life.
Natural environment has been an important supporting factor for my wellbeing during COVID-19.
Looking at nature evokes positive emotions in me.
I feel that interaction and taking contact with people become easier when I am in nature.
Nature has had a significant role in sustaining my social life during COVID-19.
Spending time in nature instead of in built environment is important for my positive mood.
I feel that I am part of
Satellite image
Basic map
Turku
13.40
30.6.2021

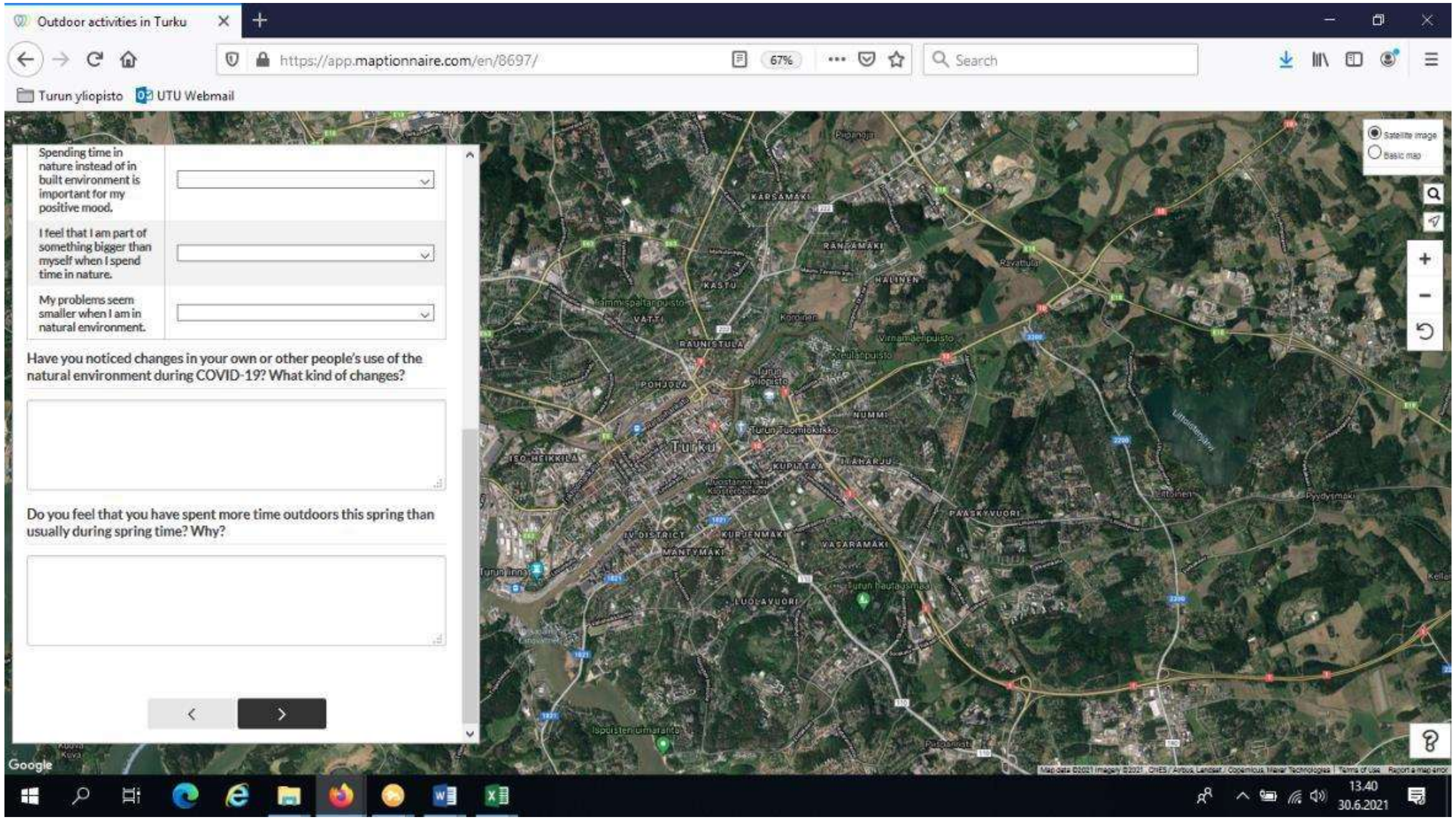
Outdoor activities in Turku
https://app.maptionnaire.com/en/8697/
Search
Turun yliopisto
UTU Webmail
Spending time in nature instead of in built environment is important for my positive mood.
I feel that I am part of something bigger than myself when I spend time in nature.
My problems seem smaller when I am in natural environment.
Have you noticed changes in your own or other people's use of the natural environment during COVID-19? What kind of changes?
Do you feel that you have spent more time outdoors this spring than usually during spring time? Why?
Satellite image
Basic map
Turku
Google
13.40
30.6.2021

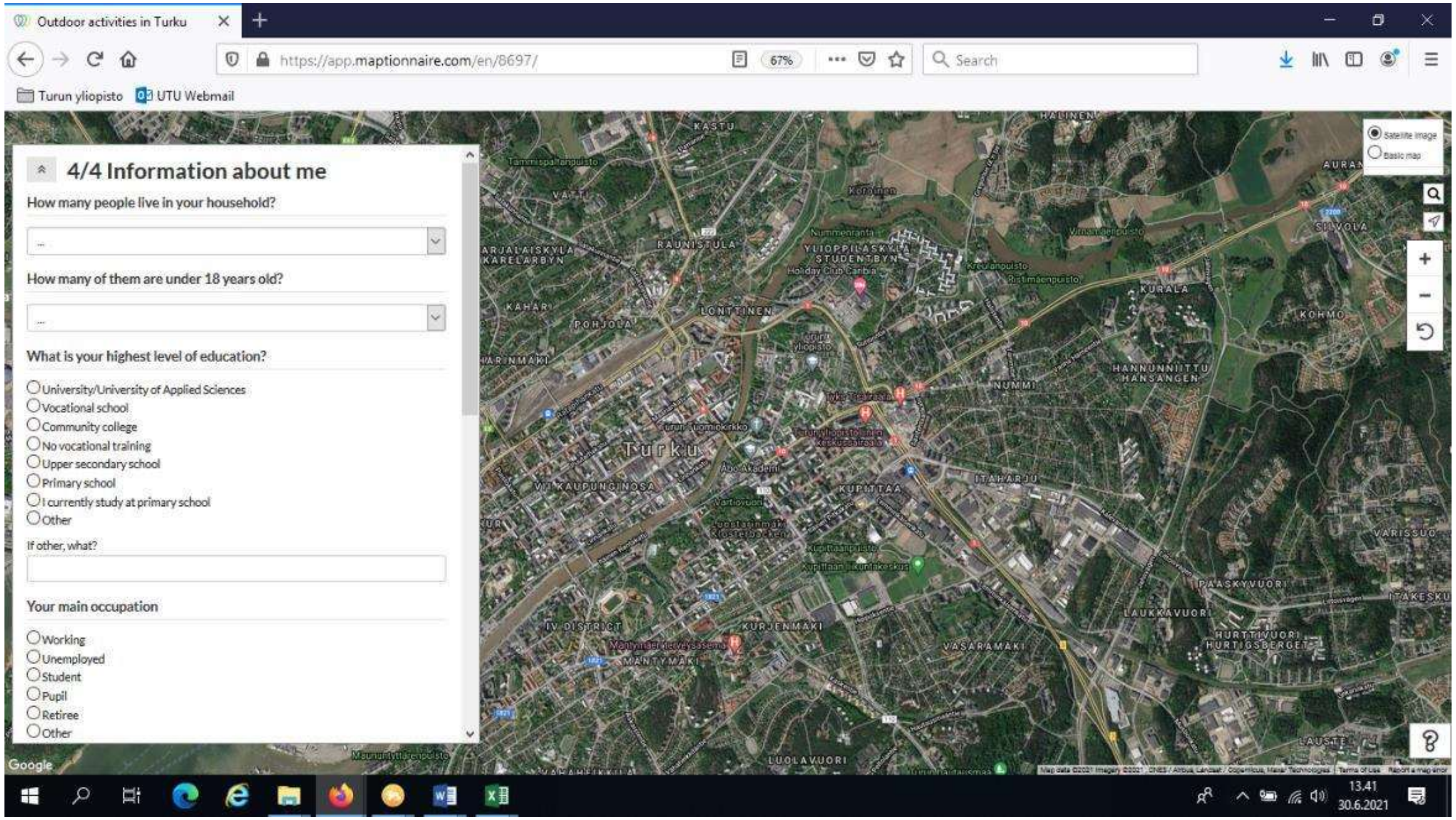
Outdoor activities in Turku
https://app.maptionnaire.com/en/8697/
67%
Search
Turun yliopisto
UTU Webmail
4/4 Information about me
How many people live in your household?
How many of them are under 18 years old?
What is your highest level of education?
University/University of Applied Sciences
Vocational school
Community college
No vocational training
Upper secondary school
Primary school
I currently study at primary school
Other
If other, what?
Your main occupation
Working
Unemployed
Student
Pupil
Retiree
Other
Satellite image
Basic map
Turku
Google
13.41
30.6.2021

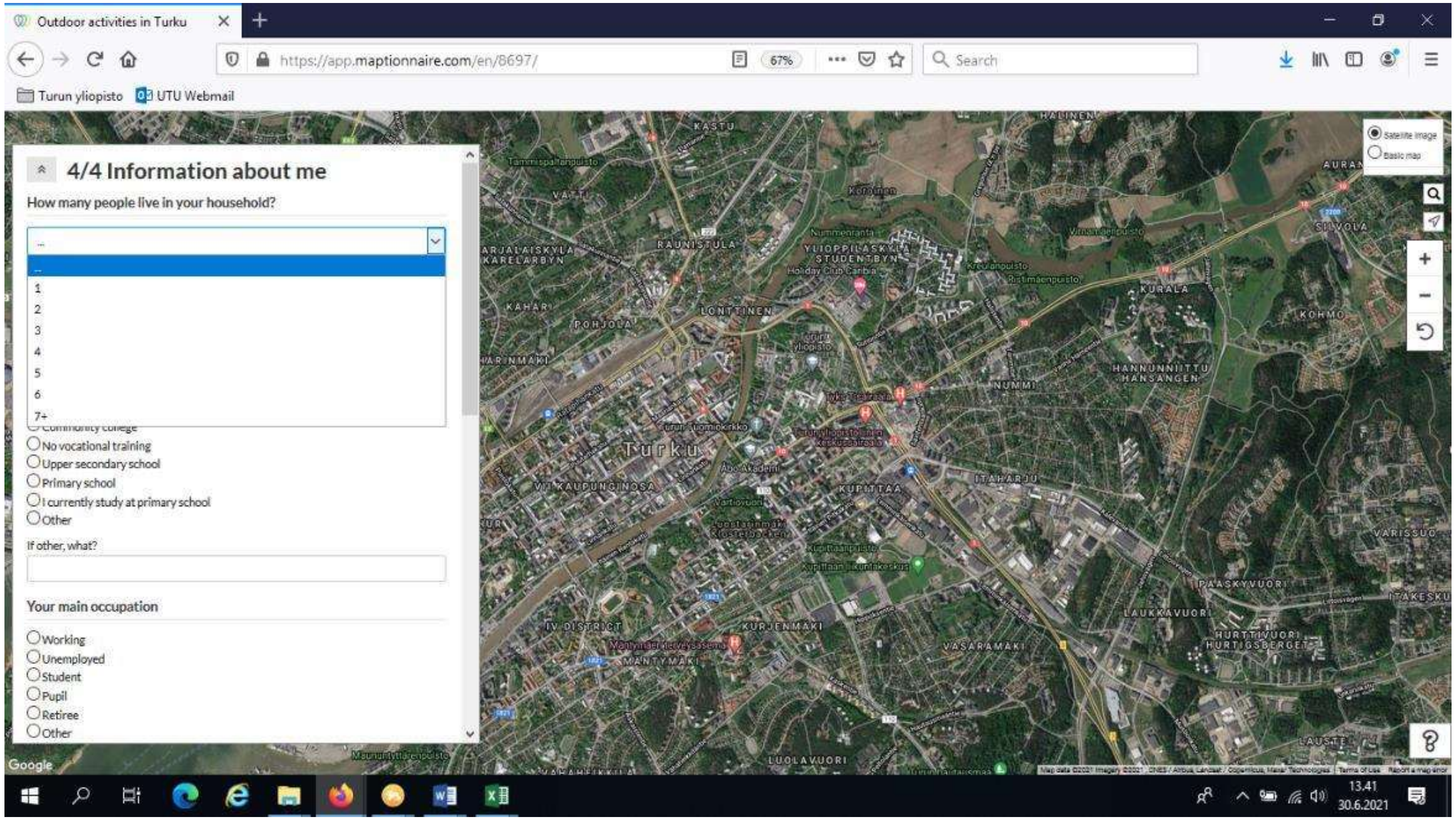
Outdoor activities in Turku
https://app.maptionnaire.com/en/8697/
Turun yliopisto
UTU Webmail
4/4 Information about me
How many people live in your household?
1
2
3
4
5
6
7+
No vocational training
Upper secondary school
Primary school
I currently study at primary school
Other
If other, what?
Your main occupation
Working
Unemployed
Student
Pupil
Retiree
Other
Turku
13.41
30.6.2021

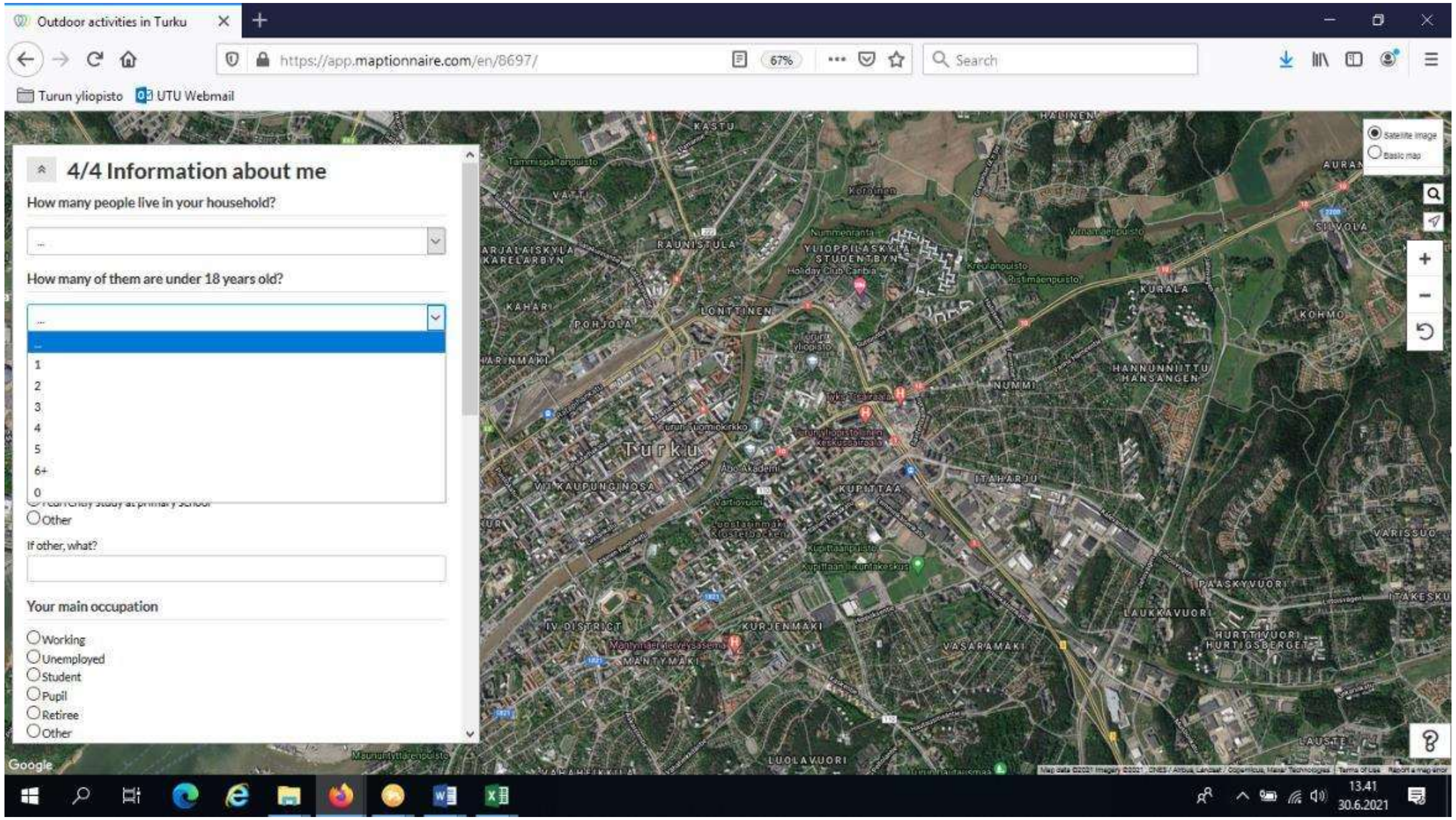
Outdoor activities in Turku
https://app.maptionnaire.com/en/8697/
Turun yliopisto
UTU Webmail
4/4 Information about me
How many people live in your household?
How many of them are under 18 years old?
1
2
3
4
5
6+
0
Other
If other, what?
Your main occupation
Working
Unemployed
Student
Pupil
Retiree
Other
Turku
13.41
30.6.2021

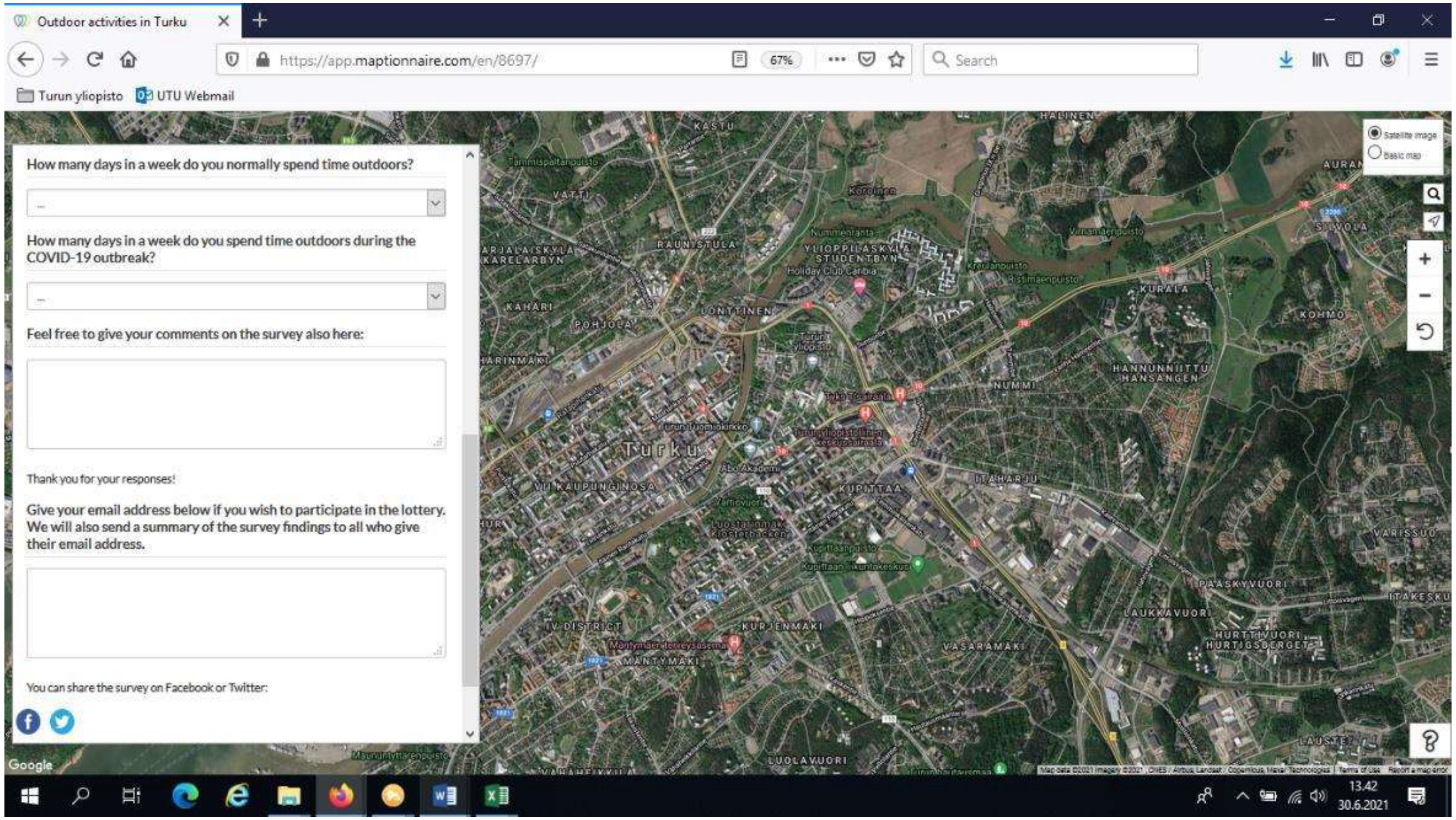
Outdoor activities in Turku
https://app.maptionnaire.com/en/8697/
Turun yliopisto
UTU Webmail
How many days in a week do you normally spend time outdoors?
How many days in a week do you spend time outdoors during the COVID-19 outbreak?
Feel free to give your comments on the survey also here:
Thank you for your responses!
Give your email address below if you wish to participate in the lottery. We will also send a summary of the survey findings to all who give their email address.
You can share the survey on Facebook or Twitter:
Turku
Satellite image
Basic map
13.42
30.6.2021

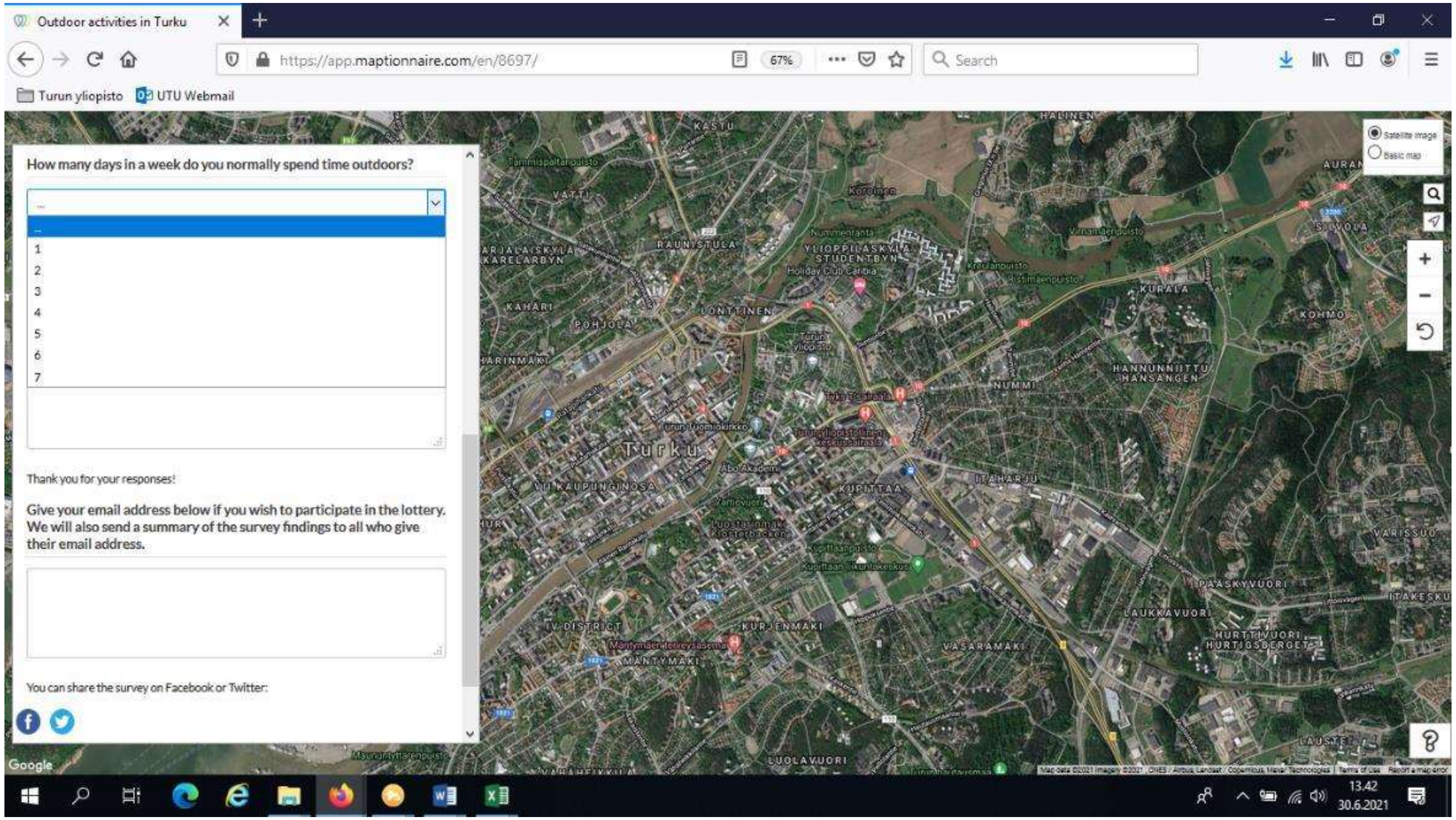
Outdoor activities in Turku
https://app.maptionnaire.com/en/8697/
Search
Turun yliopisto
UTU Webmail
How many days in a week do you normally spend time outdoors?
1
2
3
4
5
6
7
Thank you for your responses!
Give your email address below if you wish to participate in the lottery. We will also send a summary of the survey findings to all who give their email address.
You can share the survey on Facebook or Twitter:
Google
13.42
30.6.2021

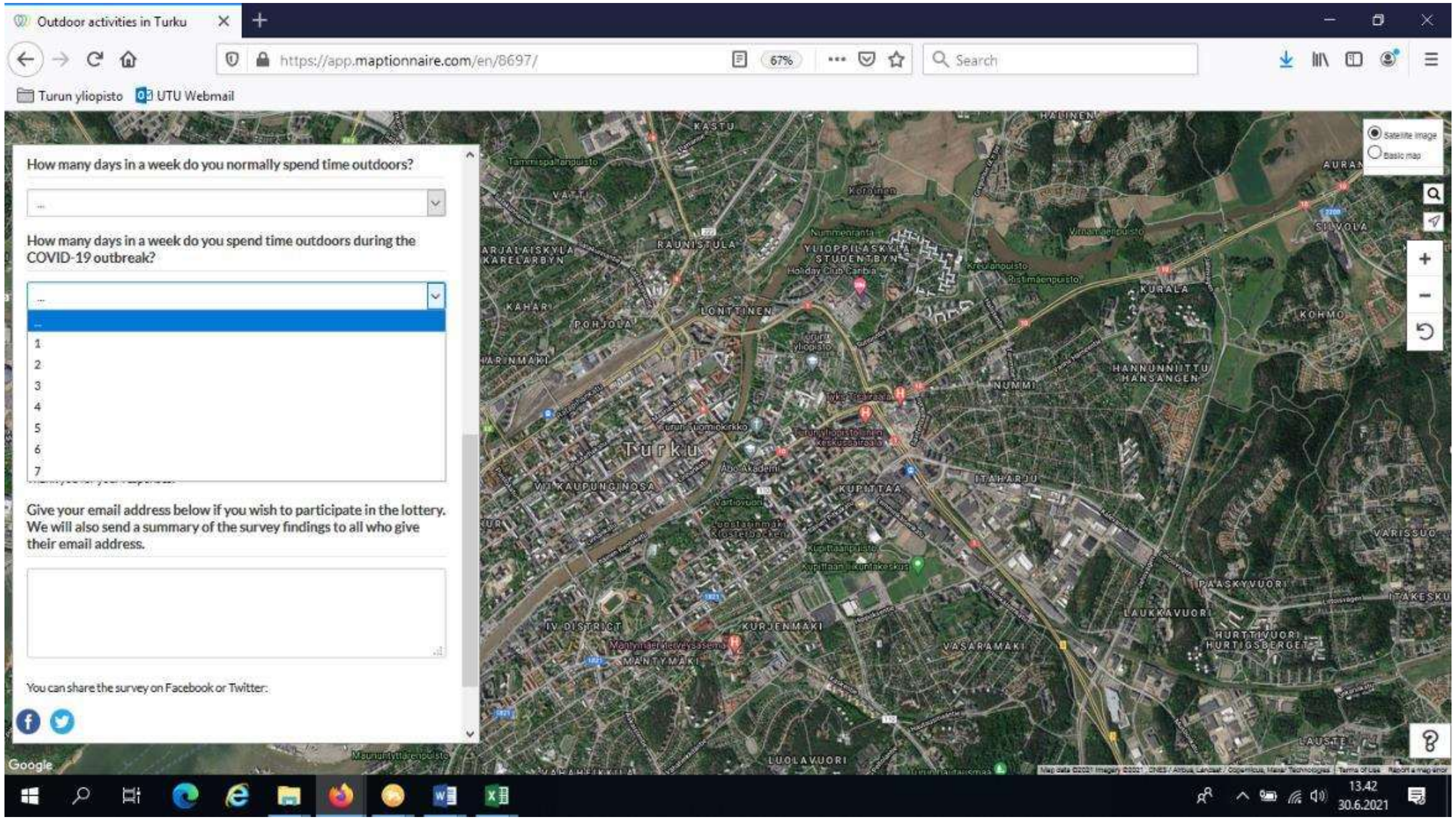

Udendørs aktiviteter i København under Coronavirus

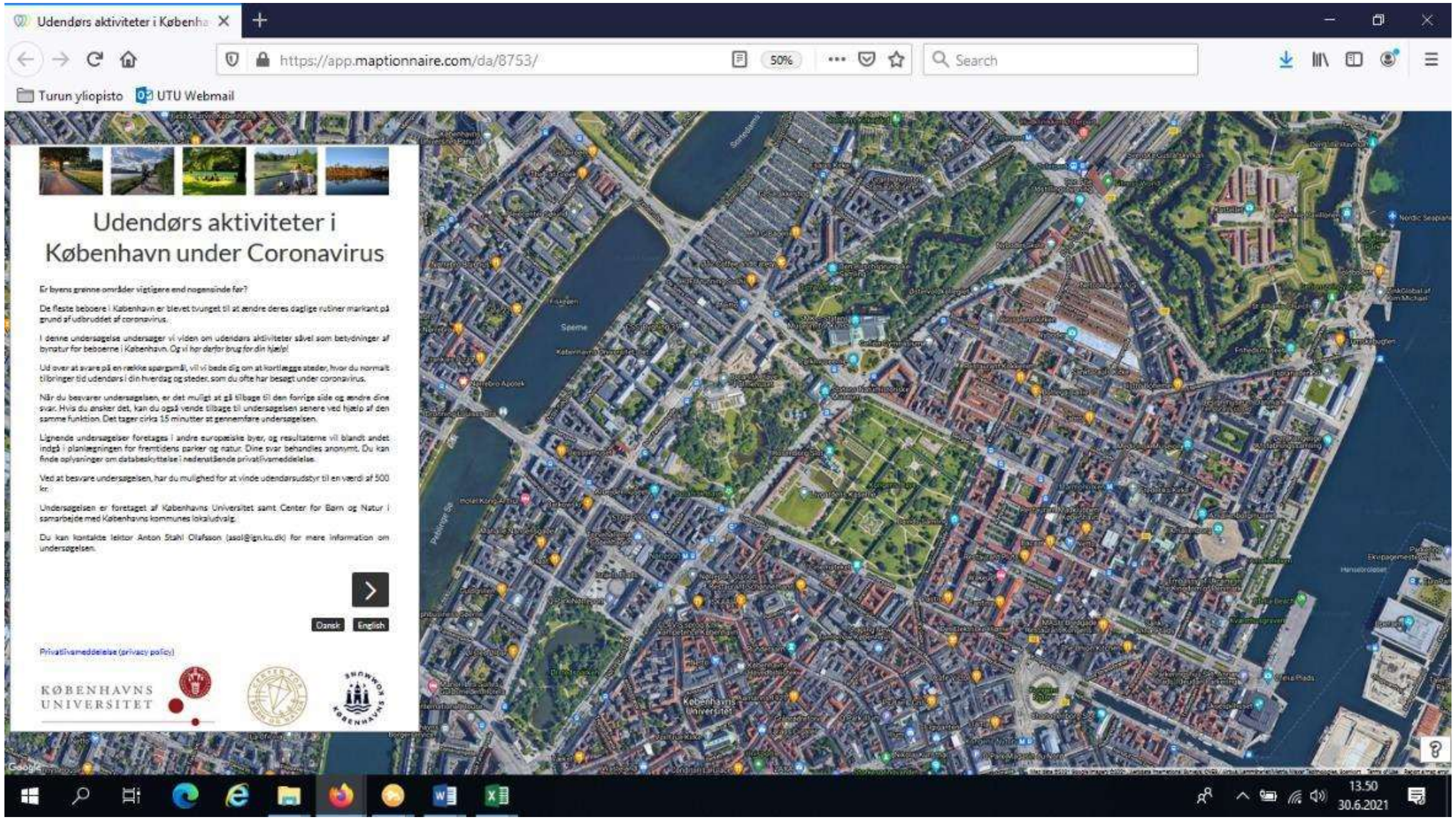
Udendørs aktiviteter i København
https://app.maptionnaire.com/da/8753/
Search
Turun yliopisto
UTU Webmail
Udendørs aktiviteter i København under Coronavirus
Er byens grønne områder vigtigere end nogensinde før?
De fleste beboere i København er blevet tvunget til at ændre deres daglige rutiner markant på grund af udbruddet af coronavirus.
I denne undersøgelse undersøger vi viden om udendørs aktiviteter såvel som betydninger af bynatur for beboerne i København. Og vi har derfor brug for din hjælp!
Ud over at svare på en række spørgsmål, vil vi bede dig om at kortlægge steder, hvor du normalt tilbringer tid udendørs i din hverdag og steder, som du ofte har besøgt under coronavirus.
Når du besvarer undersøgelsen, er det muligt at gå tilbage til den forrige side og ændre dine svar. Hvis du ønsker det, kan du også vende tilbage til undersøgelsen senere ved hjælp af den samme funktion. Det tager cirka 15 minutter at gennemføre undersøgelsen.
Lignende undersøgelser foretages i andre europæiske byer, og resultaterne vil blandt andet indgå i planlægningen for fremtidens parker og natur. Dine svar behandles anonymt. Du kan finde oplysninger om databeskyttelse i nedenstående privatlivsmeddelelse.
Ved at besvare undersøgelsen, har du mulighed for at vinde udendørsudstyr til en værdi af 500 kr.
Undersøgelsen er foretaget af Københavns Universitet samt Center for Børn og Natur i samarbejde med Københavns kommunes lokaludvalg.
Du kan kontakte lektor Anton Stahl Olafsson (asol@ign.ku.dk) for mere information om undersøgelsen.
Dansk
English
Privatlivsmeddelelse (privacy policy)
KØBENHAVNS UNIVERSITET
13.50
30.6.2021

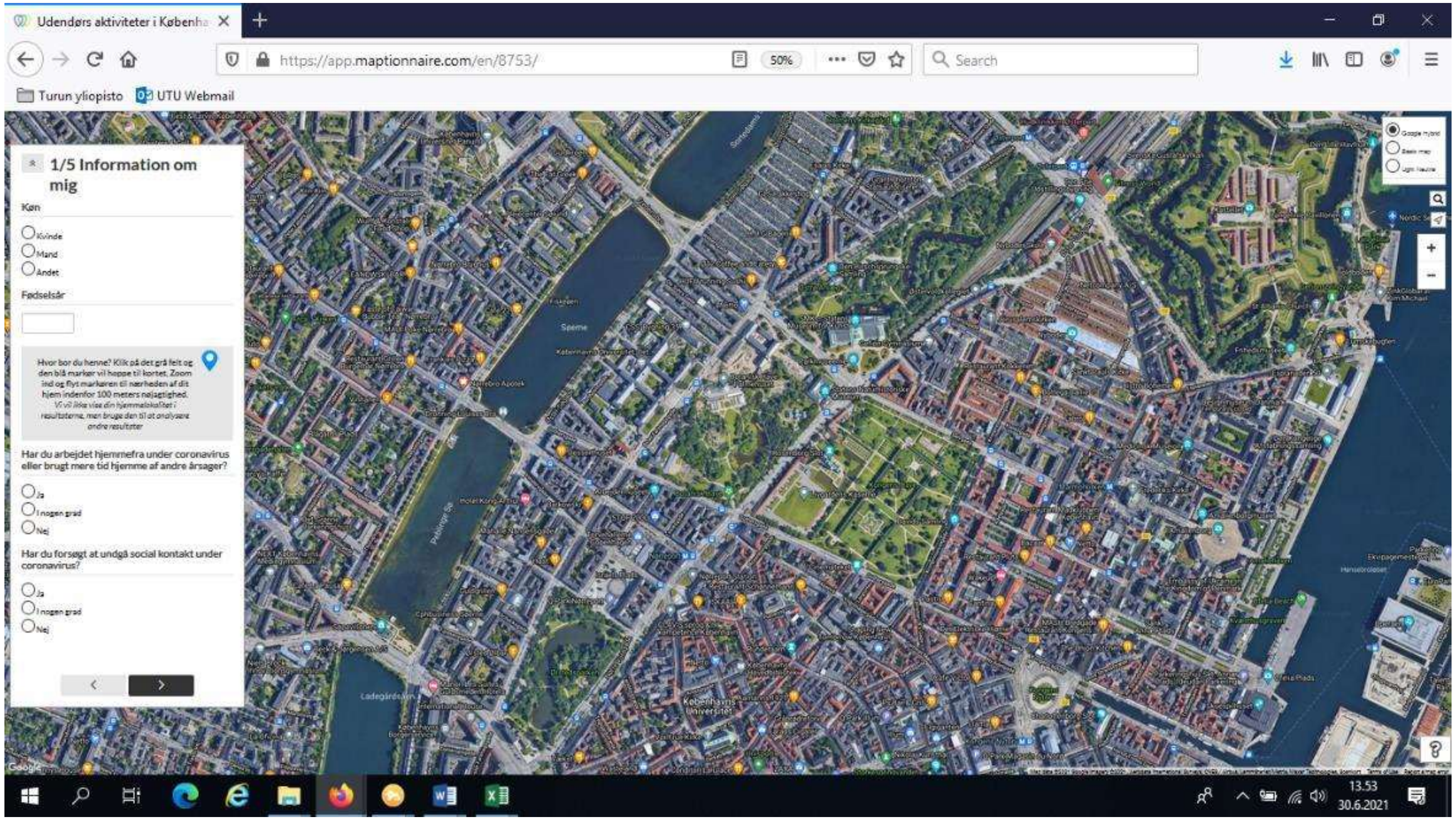

Udendørs aktiviteter i Københa
https://app.maptionnaire.com/en/8753/
50%
Search
Turun yliopisto
UTU Webmail
1/5 Information om mig
Køn
Kvinde
Mand
Andet
Fødselsår
Hvor bor du henne? Klik på det grå felt og den blå marker vil hoppe til kortet. Zoom ind og flyt markøren til nærheden af dit hjem indenfor 100 meters nøjagtighed.
Vi vil ikke vise din hjemmelokalitet i resultaterne, men bruge den til at analysere andre resultater
Har du arbejdet hjemmefra under coronavirus eller brugt mere tid hjemme af andre årsager?
Ja
I nogen grad
Nej
Har du forsøgt at undgå social kontakt under coronavirus?
Ja
I nogen grad
Nej
13.53
30.6.2021

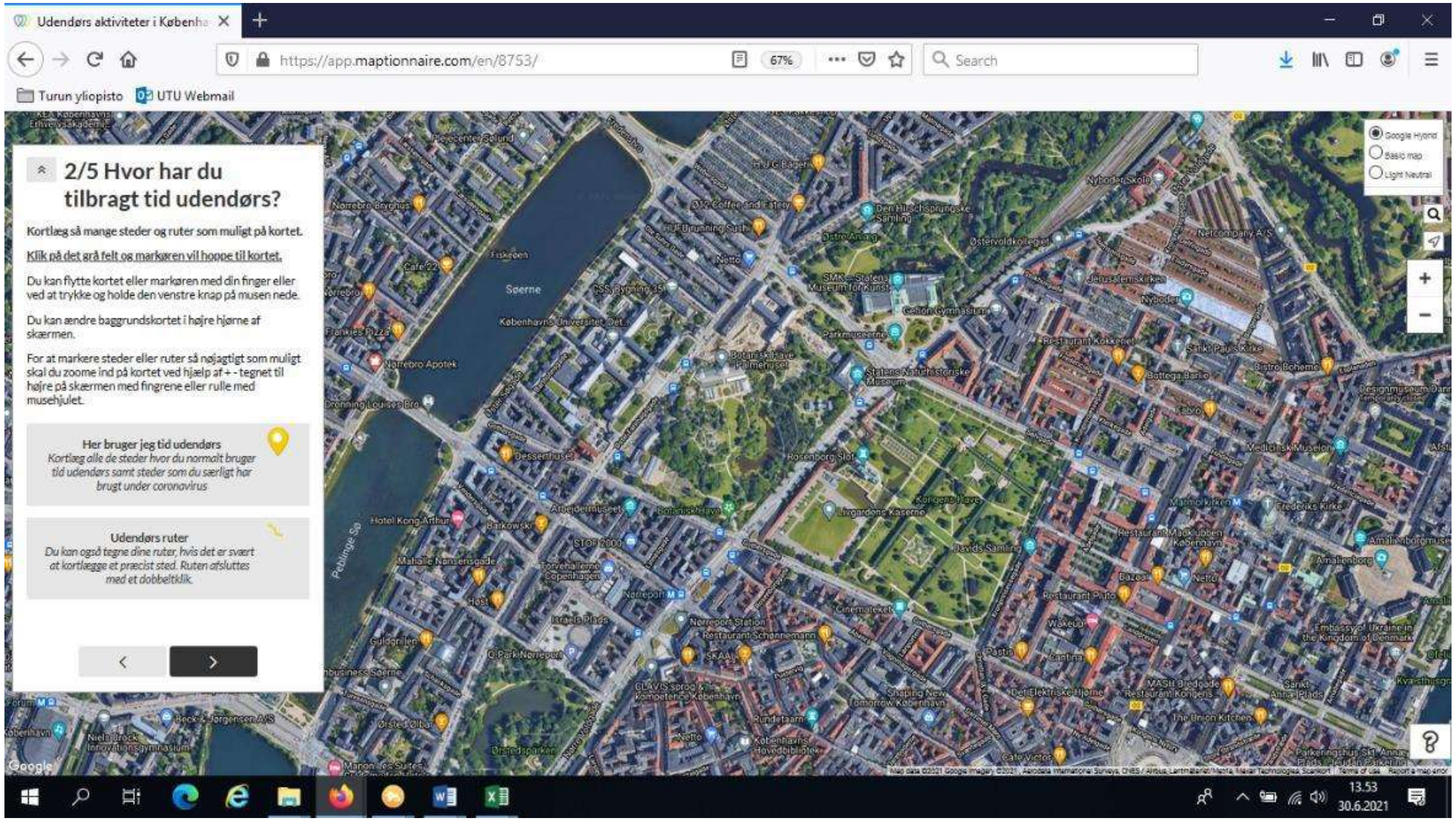
Udendørs aktiviteter i Københa
https://app.maptionnaire.com/en/8753/
Search
Turun yliopisto
UTU Webmail
2/5 Hvor har du tilbragt tid udendørs?
Kortlæg så mange steder og ruter som muligt på kortet.
Klik på det grå felt og markøren vil hoppe til kortet.
Du kan flytte kortet eller markøren med din finger eller ved at trykke og holde den venstre knap på musen nede.
Du kan ændre baggrundskortet i højre hjørne af skærmen.
For at markere steder eller ruter så nøjagtigt som muligt skal du zoome ind på kortet ved hjælp af + - tegnet til højre på skærmen med fingrene eller rulle med musehjulet.
Her bruger jeg tid udendørs
Kortlæg alle de steder hvor du normalt bruger tid udendørs samt steder som du særligt har brugt under coronavirus
Udendørs ruter
Du kan også tegne dine ruter, hvis det er svært at kortlægge et præcist sted. Ruten afsluttes med et dobbeltklik.
Søerne
Peblinge Sø
13.53
30.6.2021

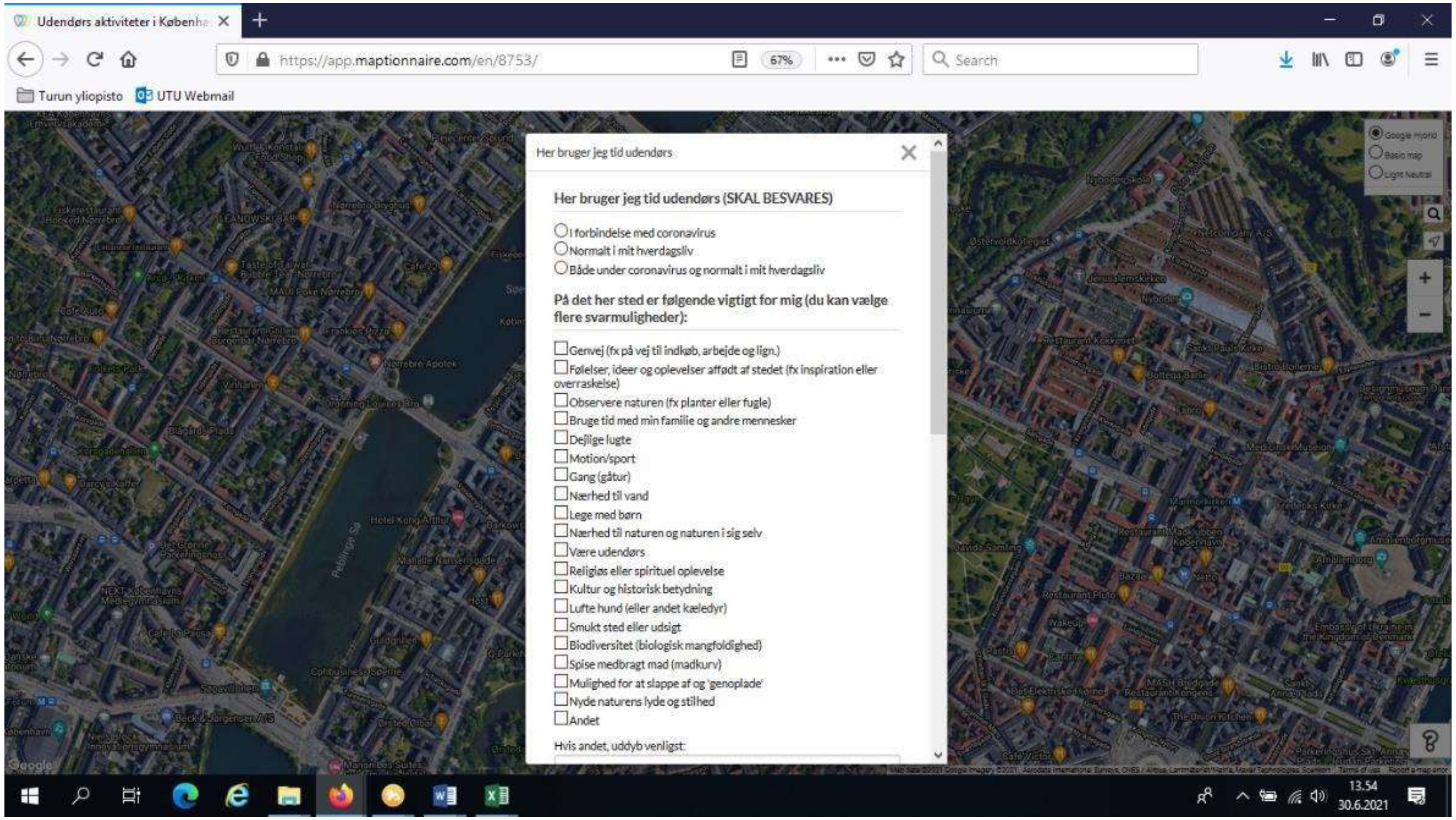
Udendørs aktiviteter i Københa
https://app.maptionnaire.com/en/8753/
Turun yliopisto
UTU Webmail
Her bruger jeg tid udendørs
Her bruger jeg tid udendørs (SKAL BESVARES)
I forbindelse med coronavirus
Normalt i mit hverdagsliv
Både under coronavirus og normalt i mit hverdagsliv
På det her sted er følgende vigtigt for mig (du kan vælge flere svarmuligheder):
Genvej (fx på vej til indkøb, arbejde og lign.)
Følelser, ideer og oplevelser affødt af stedet (fx inspiration eller overraskelse)
Observere naturen (fx planter eller fugle)
Bruge tid med min familie og andre mennesker
Dejlige lugte
Motion/sport
Gang (gåtur)
Nærhed til vand
Lege med børn
Nærhed til naturen og naturen i sig selv
Være udendørs
Religiøs eller spirituel oplevelse
Kultur og historisk betydning
Lufte hund (eller andet kæledyr)
Smukt sted eller udsigt
Biodiversitet (biologisk mangfoldighed)
Spise medbragt mad (madkurv)
Mulighed for at slappe af og 'genoplade'
Nyde naturens lyde og stilhed
Andet
Hvis andet, uddyb venligst:
13.54
30.6.2021

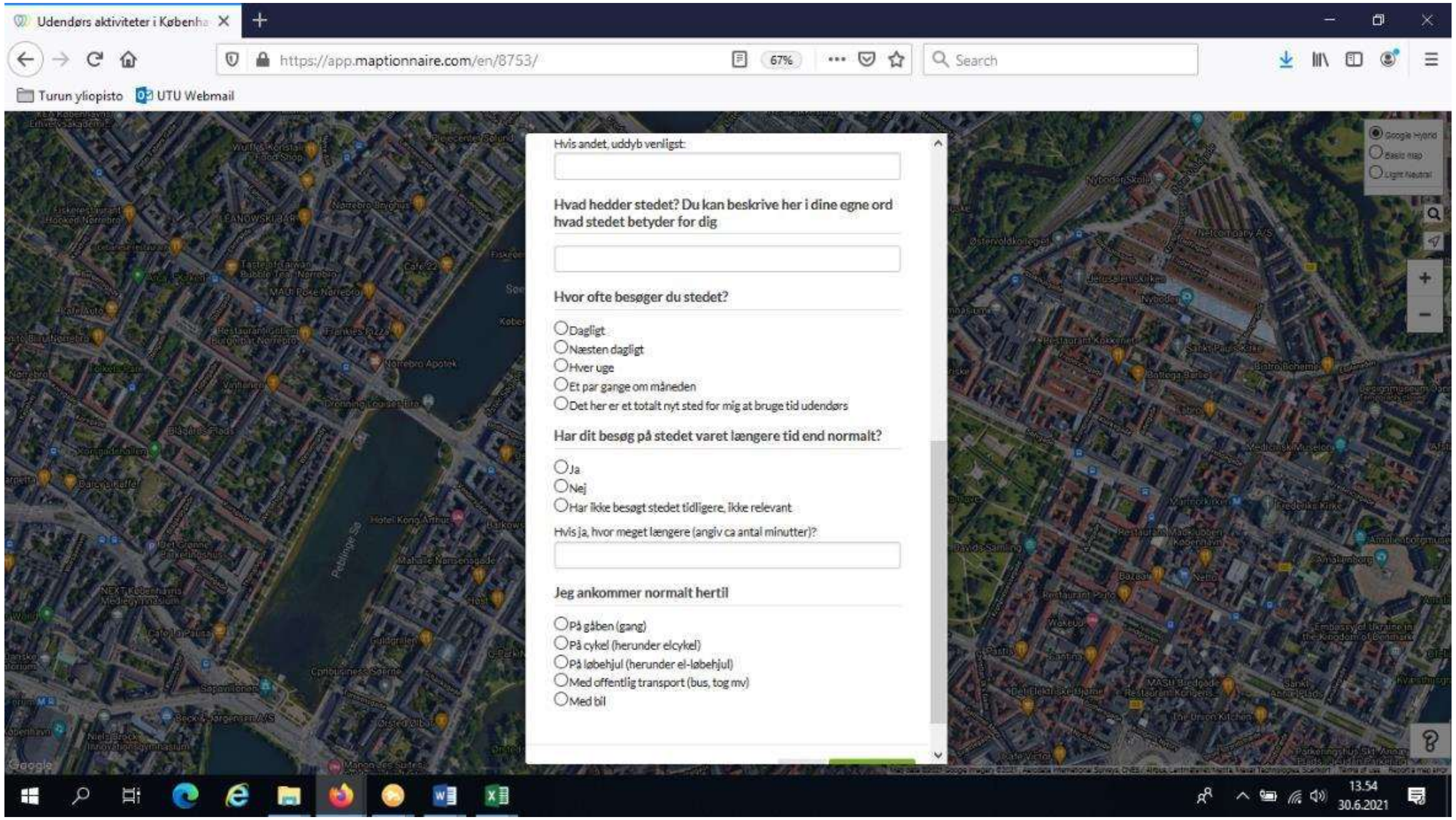
Udendørs aktiviteter i Københa
https://app.maptionnaire.com/en/8753/
Search
Turun yliopisto
UTU Webmail
Hvis andet, uddyb venligst:
Hvad hedder stedet? Du kan beskrive her i dine egne ord hvad stedet betyder for dig
Hvor ofte besøger du stedet?
Dagligt
Næsten dagligt
Hver uge
Et par gange om måneden
Det her er et totalt nyt sted for mig at bruge tid udendørs
Har dit besøg på stedet varet længere tid end normalt?
Ja
Nej
Har ikke besøgt stedet tidligere, ikke relevant
Hvis ja, hvor meget længere (angiv ca antal minutter)?
Jeg ankommer normalt hertil
På gåben (gang)
På cykel (herunder elcykel)
På løbehjul (herunder el-løbehjul)
Med offentlig transport (bus, tog mv)
Med bil
13.54
30.6.2021

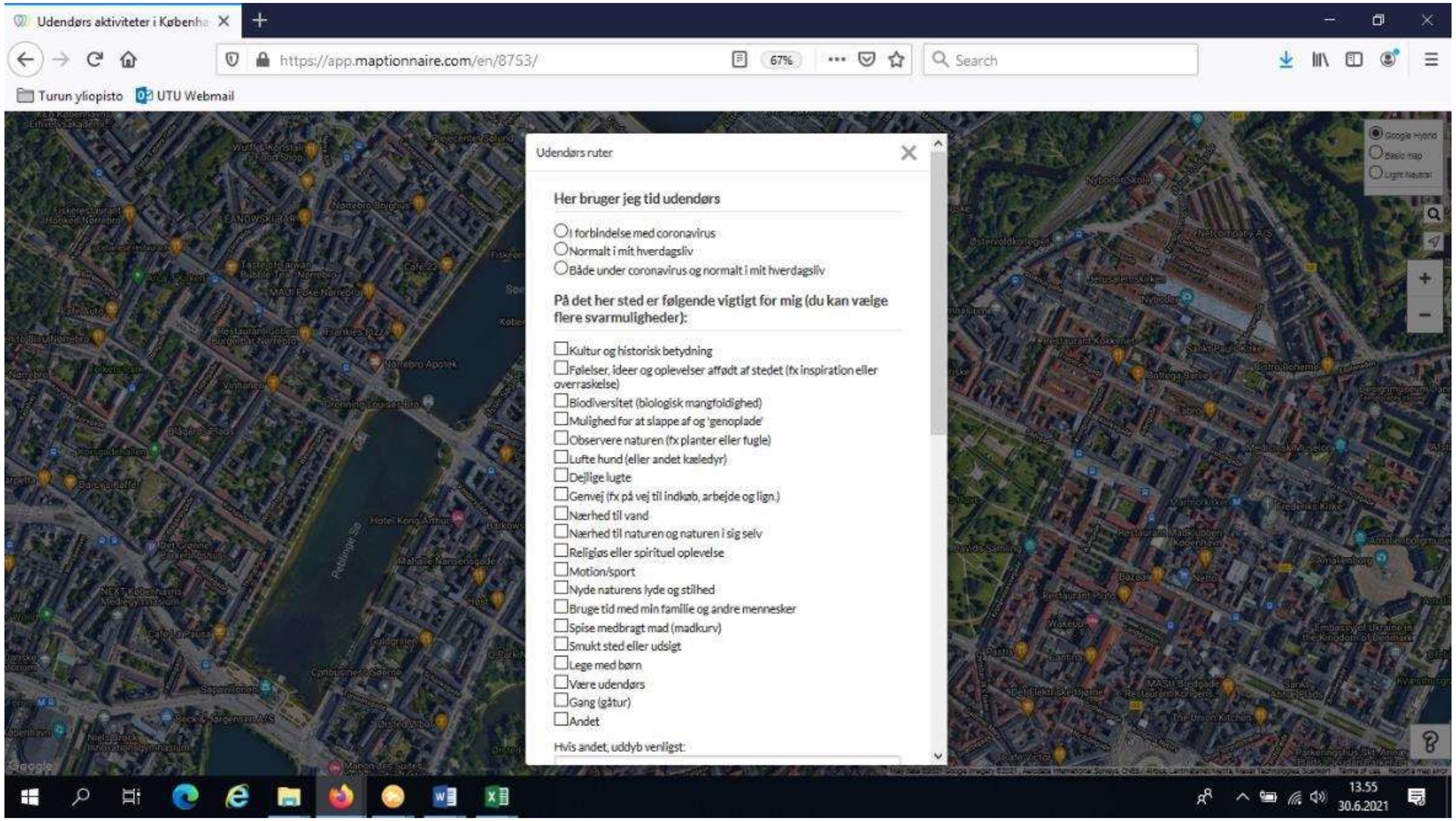

Udendørs aktiviteter i Københa
https://app.maptionnaire.com/en/8753/
67%
Search
Turun yliopisto
UTU Webmail
Udendørs ruter
Her bruger jeg tid udendørs
I forbindelse med coronavirus
Normalt i mit hverdagsliv
Både under coronavirus og normalt i mit hverdagsliv
På det her sted er følgende vigtigt for mig (du kan vælge flere svarmuligheder):
Kultur og historisk betydning
Følelser, ideer og oplevelser affødt af stedet (fx inspiration eller overraskelse)
Biodiversitet (biologisk mangfoldighed)
Mulighed for at slappe af og 'genoplade'
Observere naturen (fx planter eller fugle)
Lufte hund (eller andet kæledyr)
Dejlige lugte
Genvej (fx på vej til indkøb, arbejde og lign.)
Nærhed til vand
Nærhed til naturen og naturen i sig selv
Religiøs eller spirituel oplevelse
Motion/sport
Nyde naturens lyde og stilhed
Bruge tid med min familie og andre mennesker
Spise medbragt mad (madkurv)
Smukt sted eller udsigt
Lege med børn
Være udendørs
Gang (gåtur)
Andet
Hvis andet, uddyb venligst:
13.55
30.6.2021

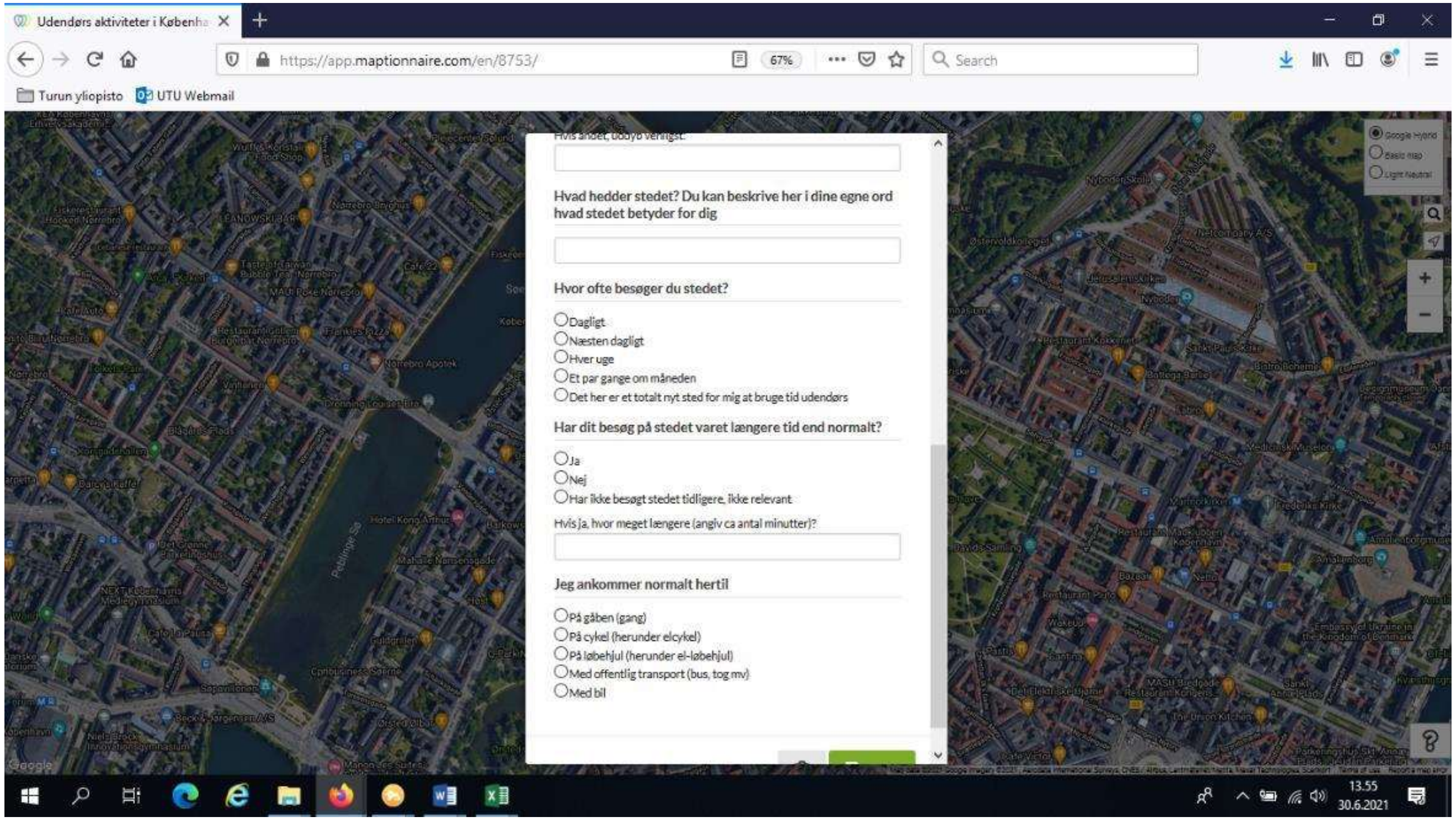

Udendørs aktiviteter i Københa
https://app.maptionnaire.com/en/8753/
Turun yliopisto
UTU Webmail
Hvad hedder stedet? Du kan beskrive her i dine egne ord hvad stedet betyder for dig
Hvor ofte besøger du stedet?
Dagligt
Næsten dagligt
Hver uge
Et par gange om måneden
Det her er et totalt nyt sted for mig at bruge tid udendørs
Har dit besøg på stedet varet længere tid end normalt?
Ja
Nej
Har ikke besøgt stedet tidligere, ikke relevant
Hvis ja, hvor meget længere (angiv ca antal minutter)?
Jeg ankommer normalt hertil
På gåben (gang)
På cykel (herunder elcykel)
På løbehjul (herunder el-løbehjul)
Med offentlig transport (bus, tog mv)
Med bil
13.55
30.6.2021

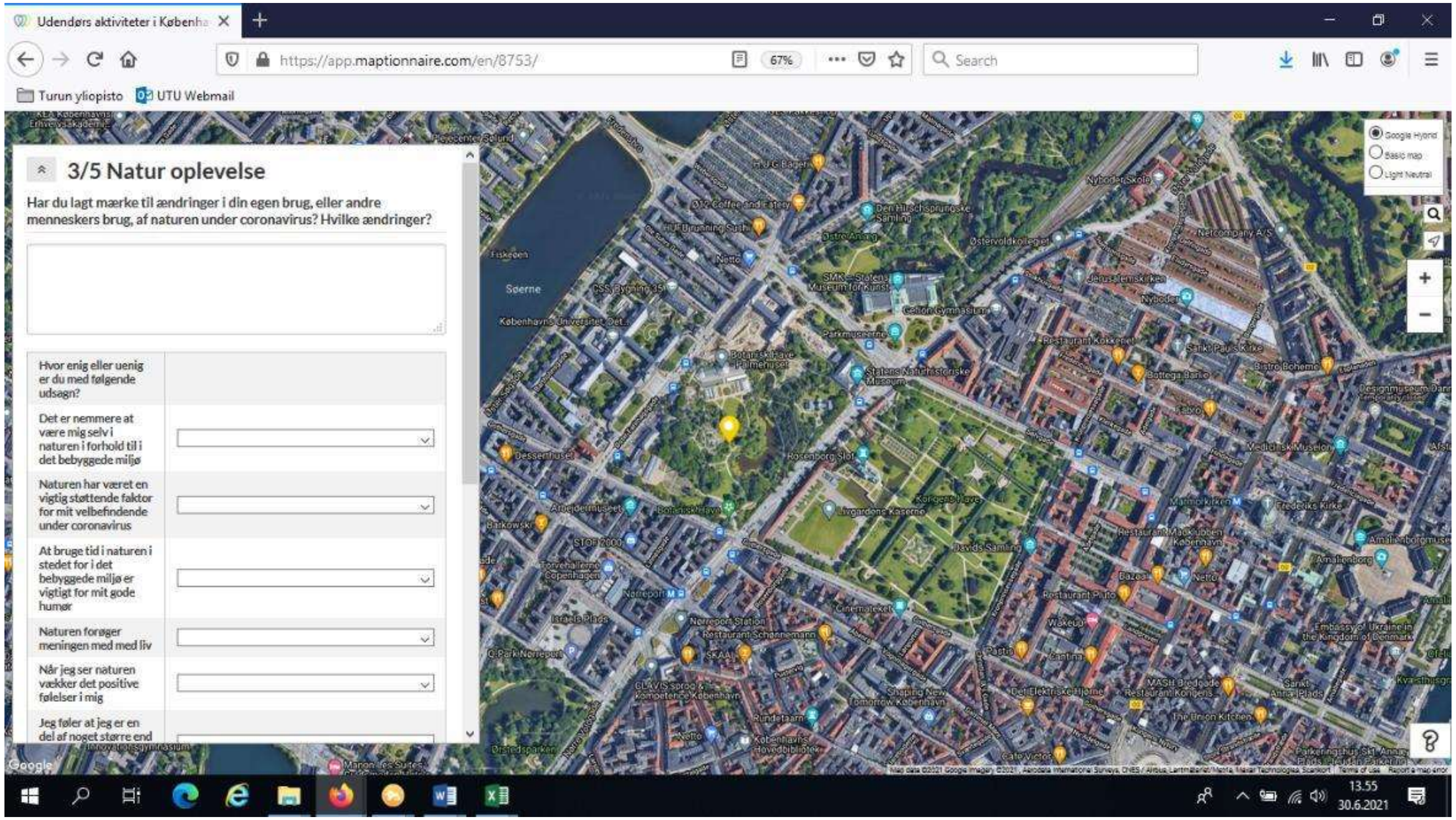
Udendørs aktiviteter i Københa
https://app.maptionnaire.com/en/8753/
Turun yliopisto
UTU Webmail
3/5 Natur oplevelse
Har du lagt mærke til ændringer i din egen brug, eller andre menneskers brug, af naturen under coronavirus? Hvilke ændringer?
Hvor enig eller uenig er du med følgende udsagn?
Det er nemmere at være mig selv i naturen i forhold til i det bebyggede miljø
Naturen har været en vigtig støttende faktor for mit velbefindende under coronavirus
At bruge tid i naturen i stedet for i det bebyggede miljø er vigtigt for mit gode humør
Naturen forøger meningen med med liv
Når jeg ser naturen vækker det positive følelser i mig
Jeg føler at jeg er en del af noget større end
Google Hybrid
Basic map
Light Neutral
13.55
30.6.2021

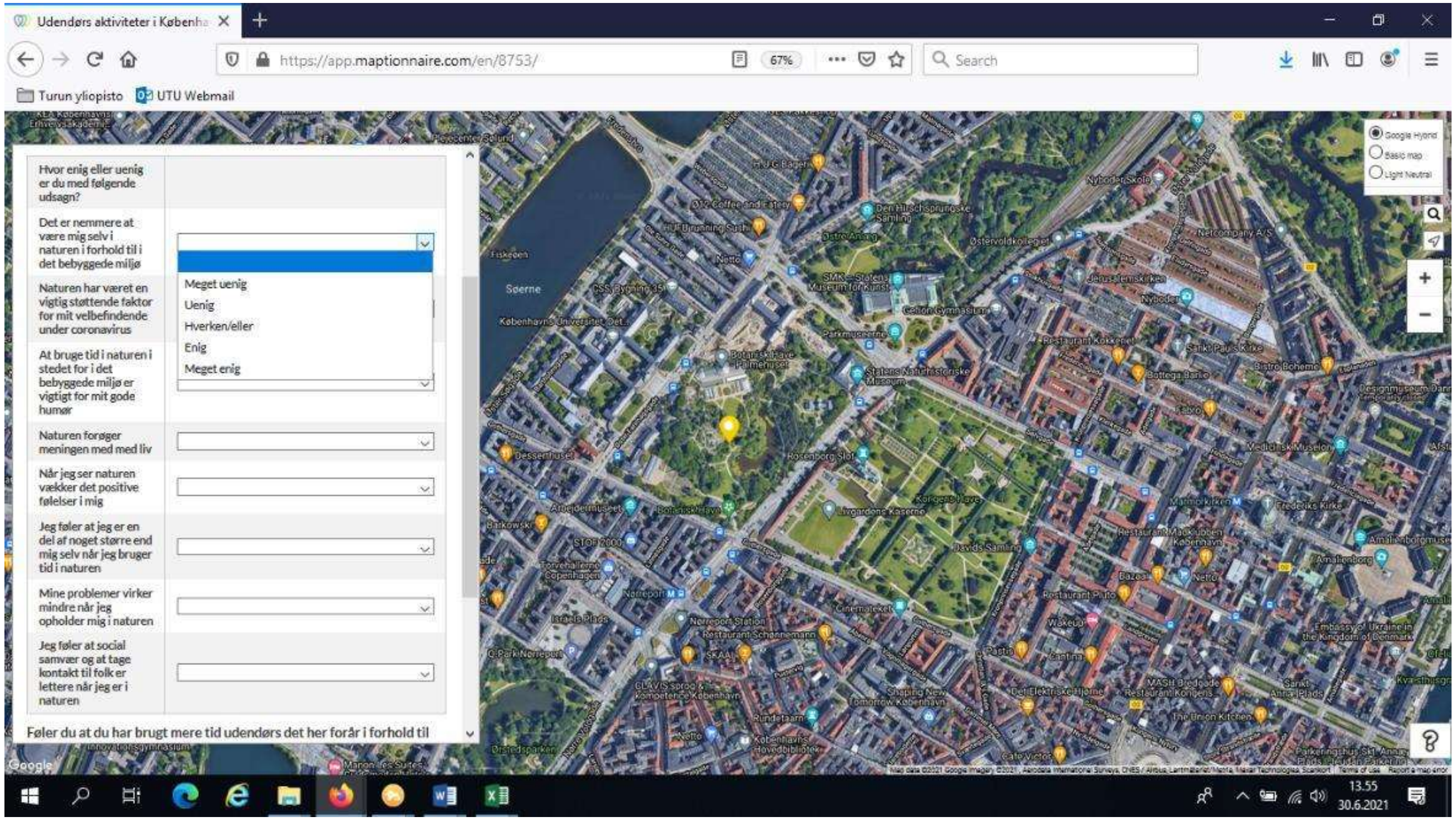
Udendørs aktiviteter i Københa
https://app.maptionnaire.com/en/8753/
Search
Turun yliopisto
UTU Webmail
Hvor enig eller uenig er du med følgende udsagn?
Det er nemmere at være mig selv i naturen i forhold til i det bebyggede miljø
Meget uenig
Uenig
Hverken/eller
Enig
Meget enig
Naturen har været en vigtig støttende faktor for mit velbefindende under coronavirus
At bruge tid i naturen i stedet for i det bebyggede miljø er vigtigt for mit gode humør
Naturen forøger meningen med med liv
Når jeg ser naturen vækker det positive følelser i mig
Jeg føler at jeg er en del af noget større end mig selv når jeg bruger tid i naturen
Mine problemer virker mindre når jeg opholder mig i naturen
Jeg føler at social samvær og at tage kontakt til folk er lettere når jeg er i naturen
Føler du at du har brugt mere tid udendørs det her forår i forhold til
Google Hybrid
Basic map
Light Neutral
13.55
30.6.2021

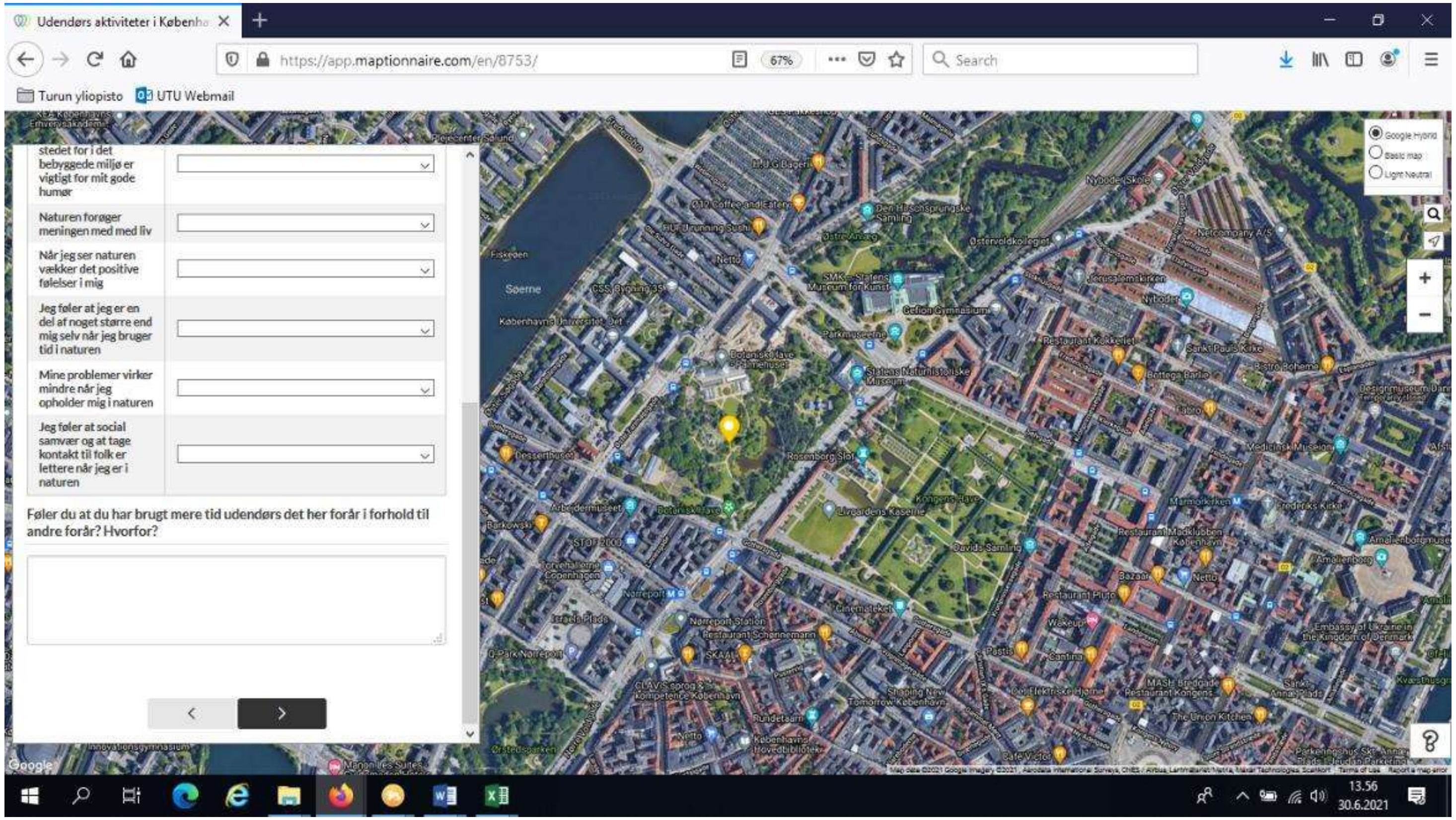

Udendørs aktiviteter i Københa
https://app.maptionnaire.com/en/8753/
Search
Turun yliopisto
UTU Webmail
stedet for i det bebyggede miljø er vigtigt for mit gode humør
Naturen forøger meningen med med liv
Når jeg ser naturen vækker det positive følelser i mig
Jeg føler at jeg er en del af noget større end mig selv når jeg bruger tid i naturen
Mine problemer virker mindre når jeg opholder mig i naturen
Jeg føler at social samvær og at tage kontakt til folk er lettere når jeg er i naturen
Føler du at du har brugt mere tid udendørs det her forår i forhold til andre forår? Hvorfor?
Søerne
Rosenborg Slot
13.56
30.6.2021

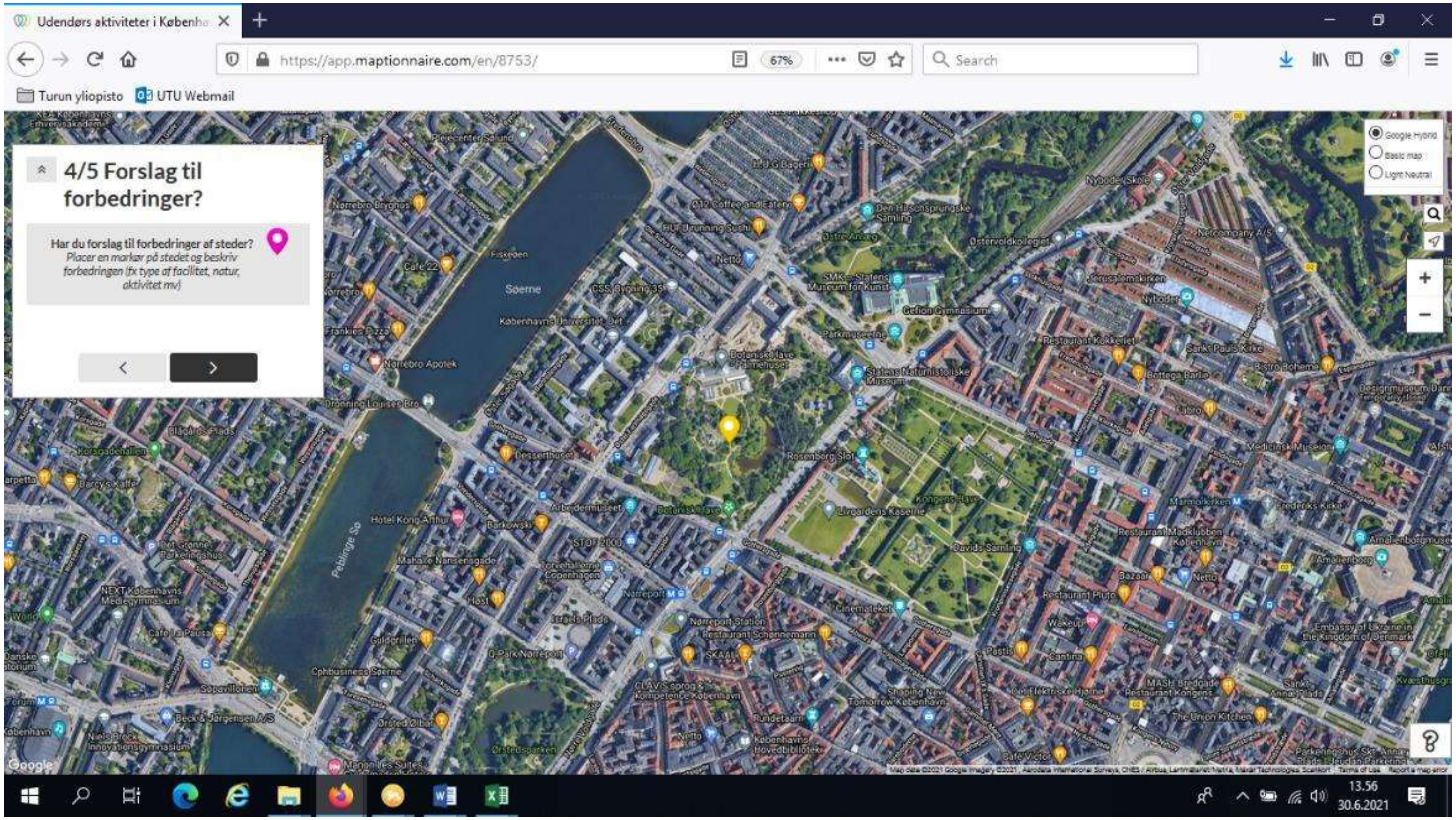

Udendørs aktiviteter i Københ
https://app.maptionnaire.com/en/8753/
Search
Turun yliopisto
UTU Webmail
4/5 Forslag til forbedringer?
Har du forslag til forbedringer af steder?
Placer en markør på stedet og beskriv forbedringen (fx type af facilitet, natur, aktivitet mv)
Google Hybrid
Basic map
Light Neutral
Søerne
Peblinge Sø
Rosenborg Slot
Nørreport Station
13.56
30.6.2021

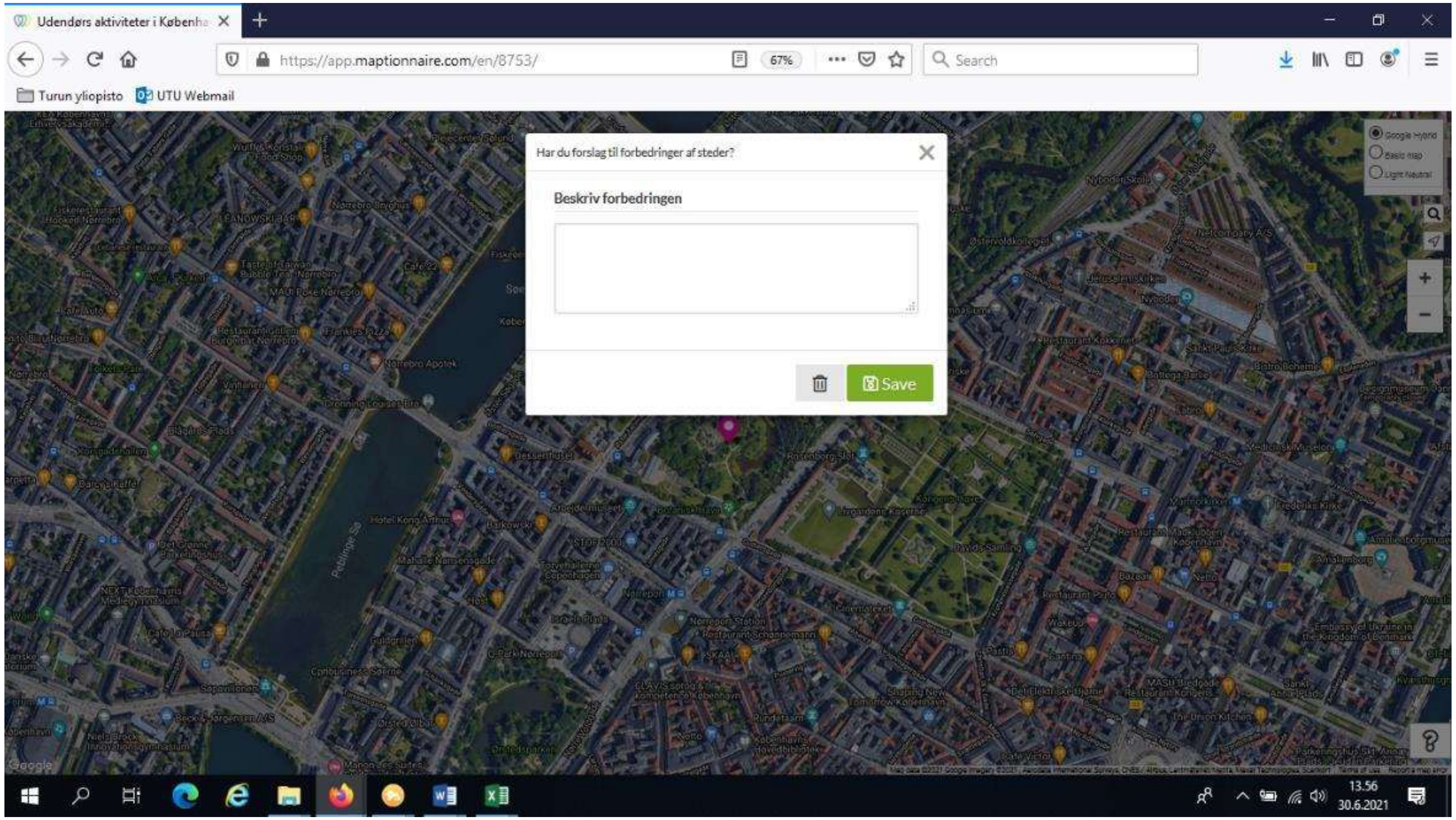
Udendørs aktiviteter i Københa
https://app.maptionnaire.com/en/8753/
67%
Search
Turun yliopisto
UTU Webmail
Har du forslag til forbedringer af steder?
Beskriv forbedringen
Save
13.56
30.6.2021

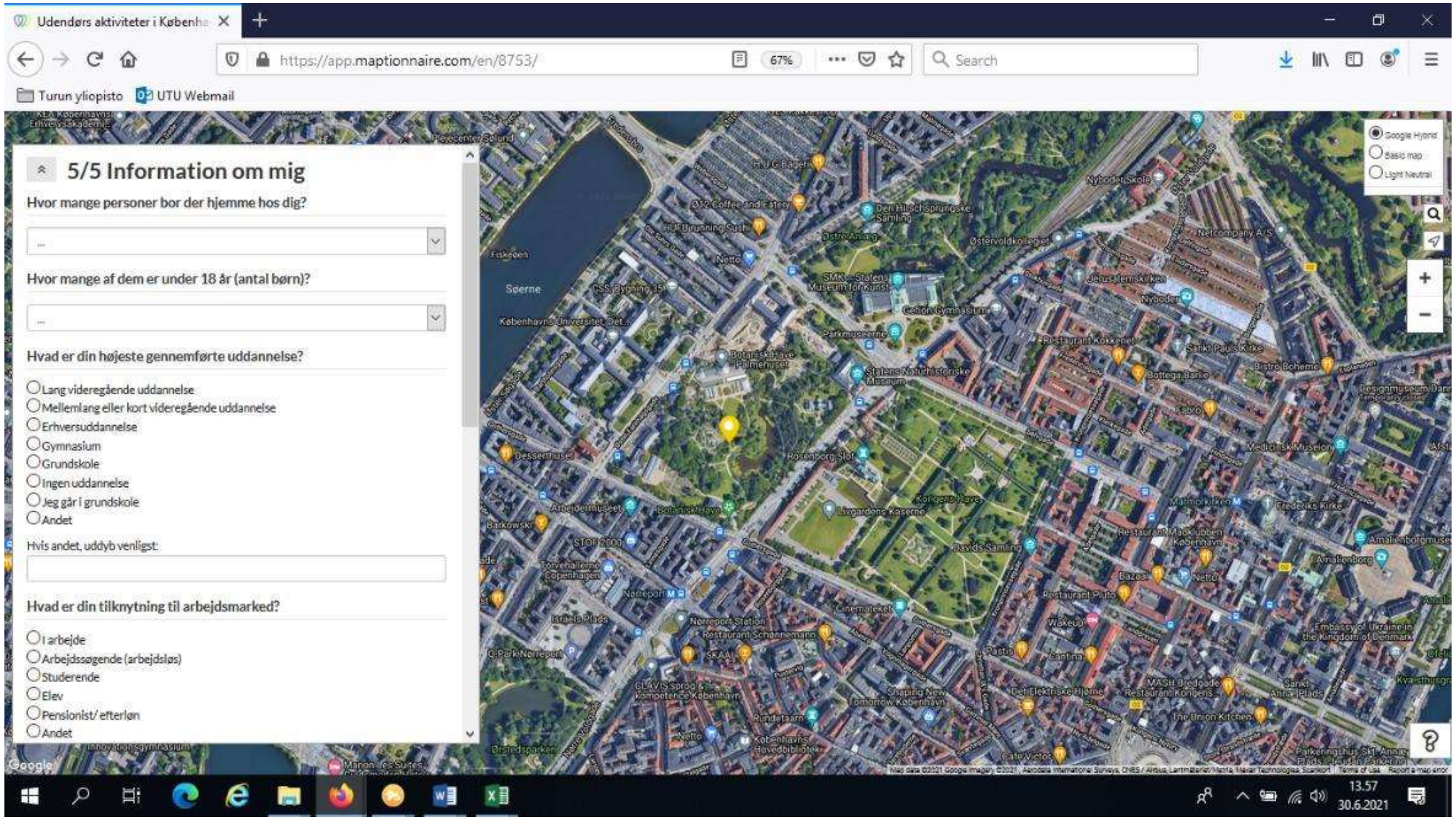
Udendørs aktiviteter i Københa
https://app.maptionnaire.com/en/8753/
Search
Turun yliopisto
UTU Webmail
5/5 Information om mig
Hvor mange personer bor der hjemme hos dig?
Hvor mange af dem er under 18 år (antal børn)?
Hvad er din højeste gennemførte uddannelse?
Lang videregående uddannelse
Mellemlang eller kort videregående uddannelse
Erhversuddannelse
Gymnasium
Grundskole
Ingen uddannelse
Jeg går i grundskole
Andet
Hvis andet, uddyb venligst:
Hvad er din tilknytning til arbejdsmarked?
I arbejde
Arbejdssøgende (arbejdsløs)
Studerende
Elev
Pensionist/ efterløn
Andet
13.57
30.6.2021

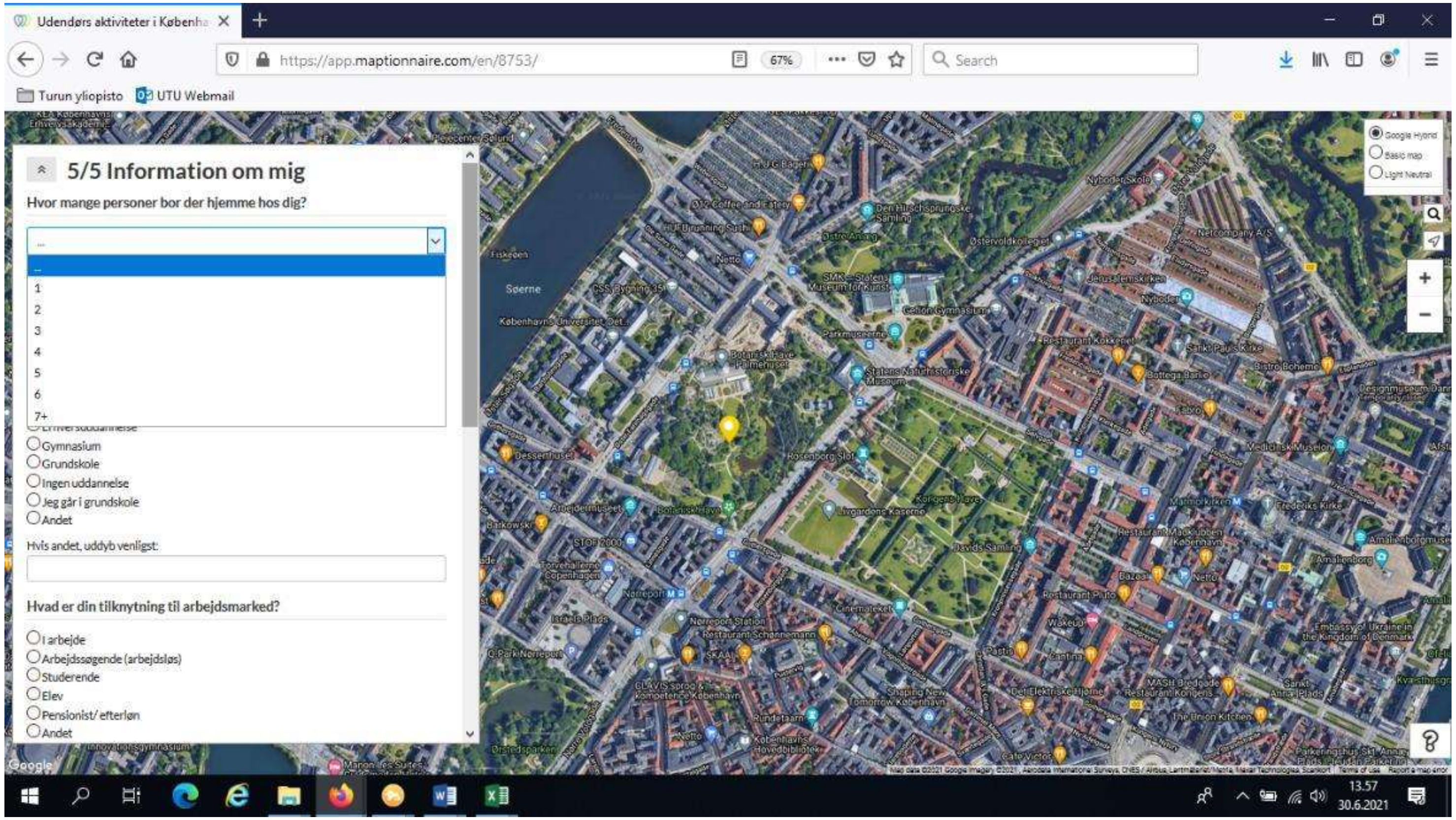
Udendørs aktiviteter i Københ
https://app.maptionnaire.com/en/8753/
Turun yliopisto
UTU Webmail
5/5 Information om mig
Hvor mange personer bor der hjemme hos dig?
1
2
3
4
5
6
7+
Gymnasium
Grundskole
Ingen uddannelse
Jeg går i grundskole
Andet
Hvis andet, uddyb venligst:
Hvad er din tilknytning til arbejdsmarked?
I arbejde
Arbejdssøgende (arbejdsløs)
Studerende
Elev
Pensionist/ efterløn
Andet
Google Hybrid
Basic map
Light Neutral
13.57
30.6.2021

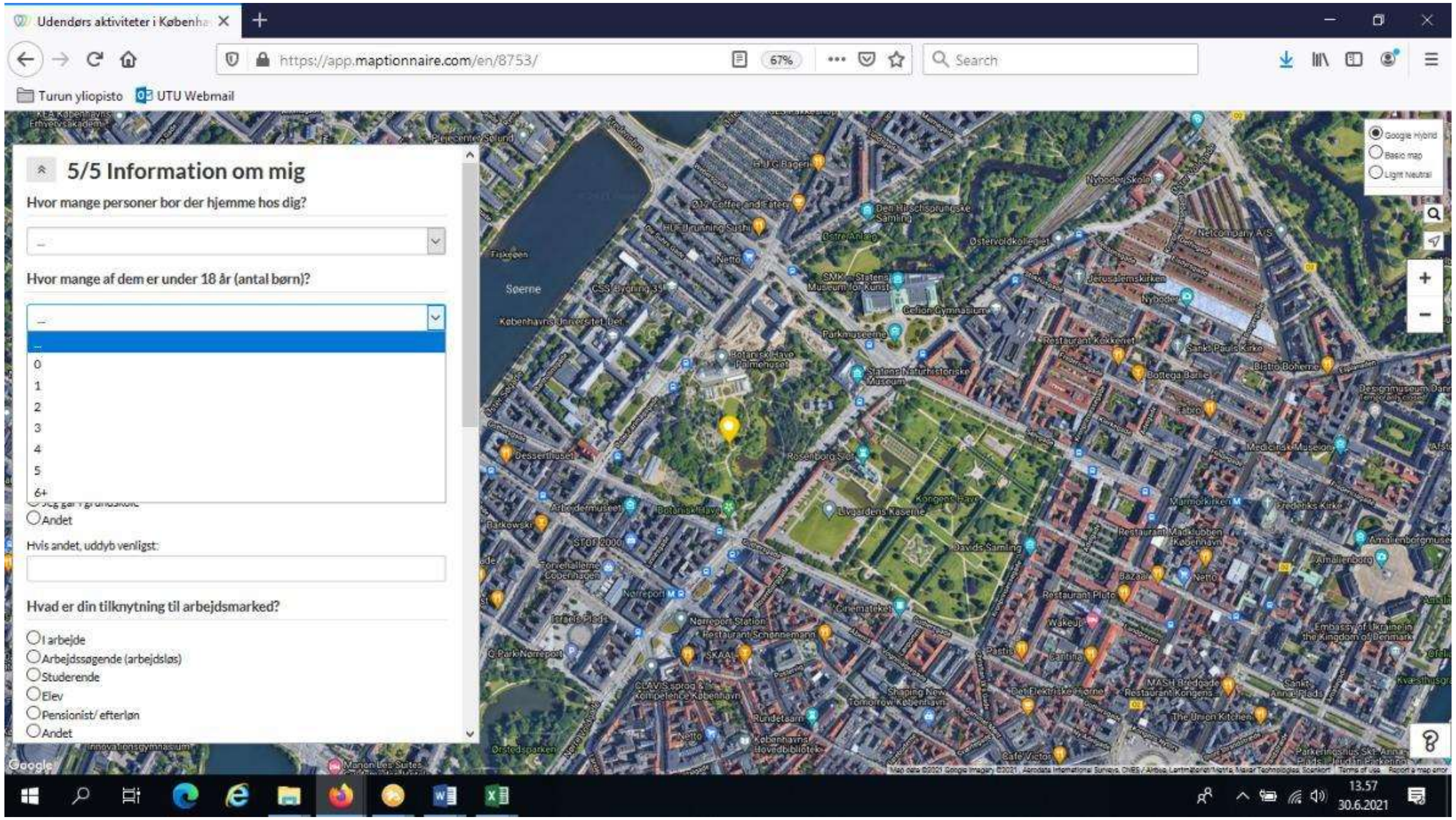
Udendørs aktiviteter i Københa
https://app.maptionnaire.com/en/8753/
Turun yliopisto
UTU Webmail
5/5 Information om mig
Hvor mange personer bor der hjemme hos dig?
Hvor mange af dem er under 18 år (antal børn)?
0
1
2
3
4
5
6+
Andet
Hvis andet, uddyb venligst:
Hvad er din tilknytning til arbejdsmarked?
I arbejde
Arbejdssøgende (arbejdsløs)
Studerende
Elev
Pensionist/ efterløn
Andet
Google Hybrid
Basic map
Light Neutral
13.57
30.6.2021

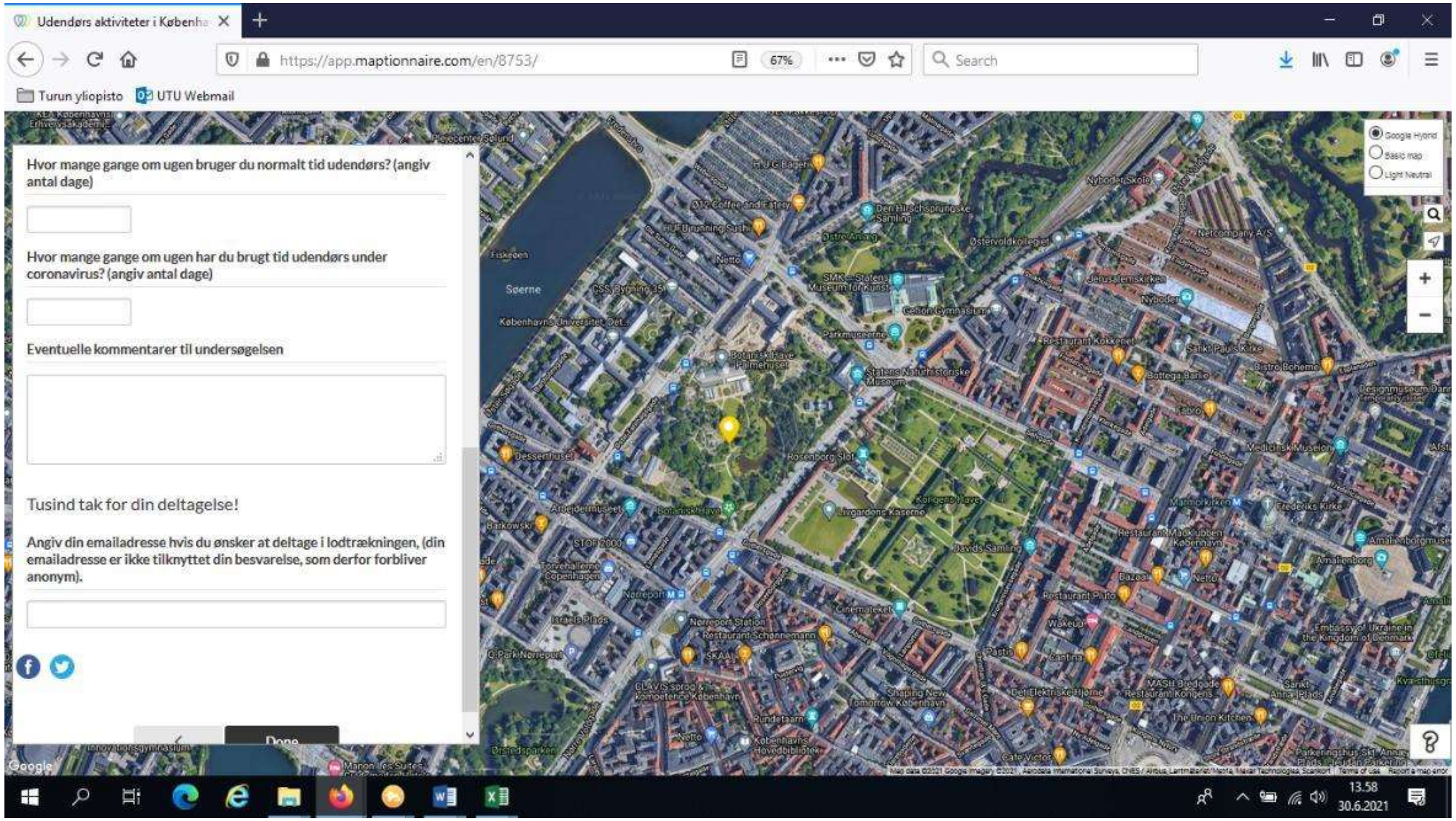

Vår Stad – Sverige

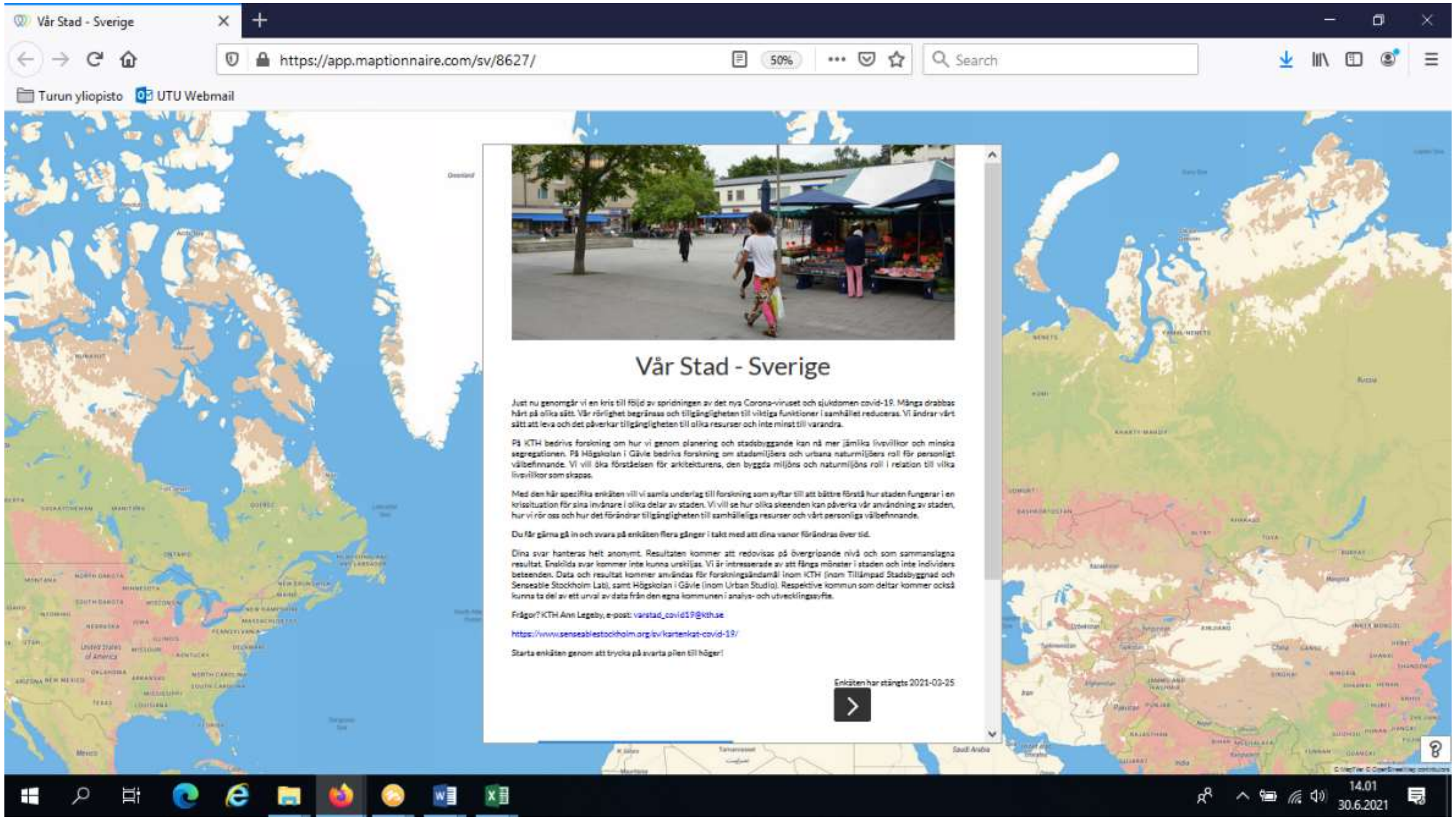
Vår Stad - Sverige
https://app.maptionnaire.com/sv/8627/
Search
Turun yliopisto
UTU Webmail
Vår Stad - Sverige
Just nu genomgår vi en kris till följd av spridningen av det nya Corona-viruset och sjukdomen covid-19. Många drabbas hårt på olika sätt. Vår rörlighet begränsas och tillgängligheten till viktiga funktioner i samhället reduceras. Vi ändrar vårt sätt att leva och det påverkar tillgängligheten till olika resurser och inte minst till varandra.
På KTH bedrivs forskning om hur vi genom planering och stadsbyggande kan nå mer jämlika livsvillkor och minska segregationen. På Högskolan i Gävle bedrivs forskning om stadsmiljöers och urbana naturmiljöers roll för personligt välbefinnande. Vi vill öka förståelsen för arkitekturens, den byggda miljöns och naturmiljöns roll i relation till vilka livsvillkor som skapas.
Med den här specifika enkäten vill vi samla underlag till forskning som syftar till att bättre förstå hur staden fungerar i en krissituation för sina invånare i olika delar av staden. Vi vill se hur olika skeenden kan påverka vår användning av staden, hur vi rör oss och hur det förändrar tillgängligheten till samhälleliga resurser och vårt personliga välbefinnande.
Du får gärna gå in och svara på enkäten flera gånger i takt med att dina vanor förändras över tid.
Dina svar hanteras helt anonymt. Resultaten kommer att redovisas på övergripande nivå och som sammanslagna resultat. Enskilda svar kommer inte kunna urskiljas. Vi är intresserade av att fånga mönster i staden och inte individers beteenden. Data och resultat kommer användas för forskningsändamål inom KTH (inom Tillämpad Stadsbyggnad och Senseable Stockholm Lab), samt Högskolan i Gävle (inom Urban Studio). Respektive kommun som deltar kommer också kunna ta del av ett urval av data från den egna kommunen i analys- och utvecklingssyfte.
Frågor? KTH Ann Legeby, e-post: varstad_covid19@kth.se
https://www.senseablestockholm.org/sv/kartenkat-covid-19/
Starta enkäten genom att trycka på svarta pilen till höger!
Enkäten har stängts 2021-02-25
14.01
30.6.2021

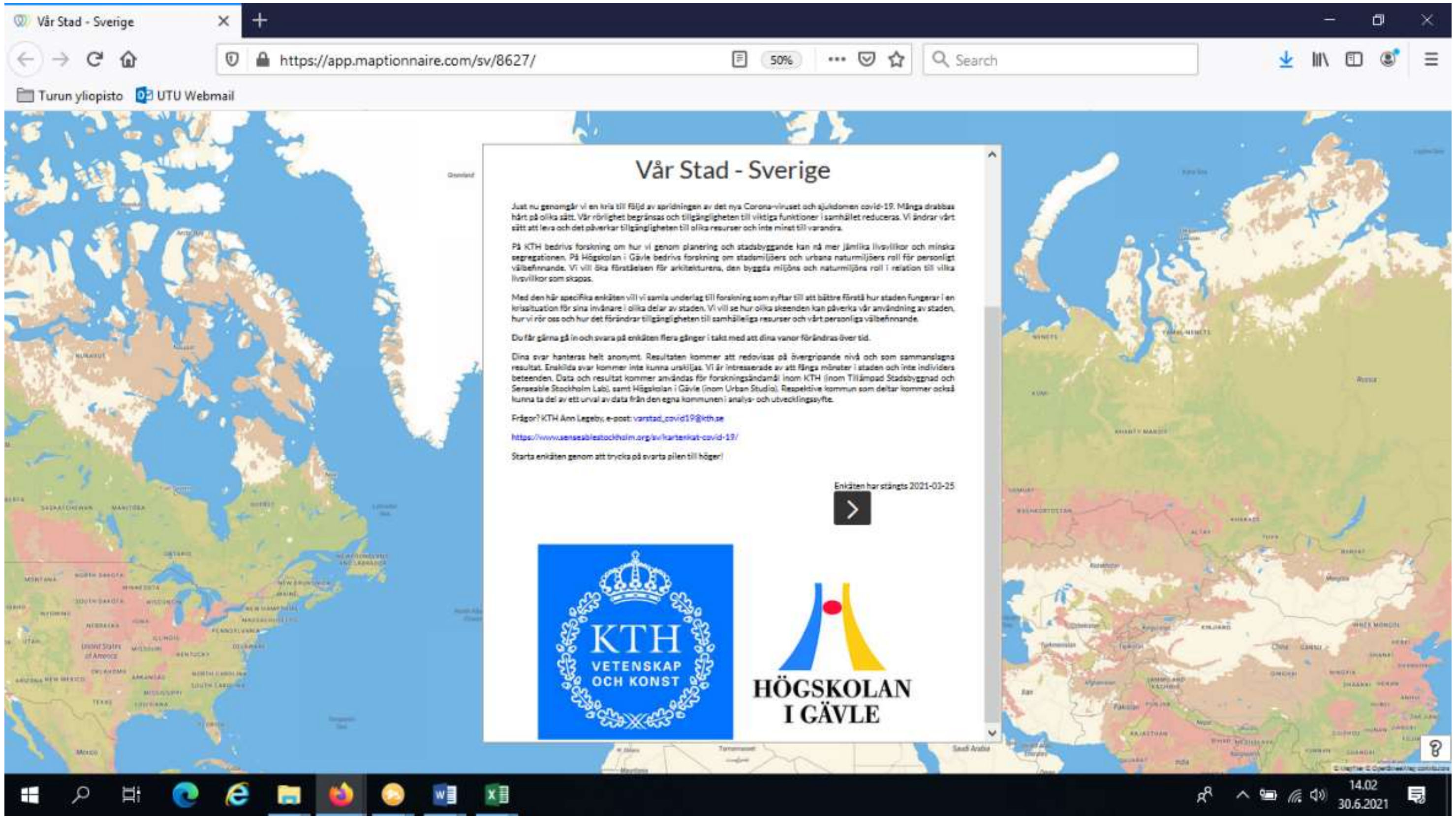
Vår Stad - Sverige
https://app.maptionnaire.com/sv/8627/
Turun yliopisto
UTU Webmail
Vår Stad - Sverige
Just nu genomgår vi en kris till följd av spridningen av det nya Corona-viruset och sjukdomen covid-19. Många drabbas hårt på olika sätt. Vår rörlighet begränsas och tillgängligheten till viktiga funktioner i samhället reduceras. Vi ändrar vårt sätt att leva och det påverkar tillgängligheten till olika resurser och inte minst till varandra.
På KTH bedrivs forskning om hur vi genom planering och stadsbyggande kan nå mer jämlika livsvillkor och minska segregationen. På Högskolan i Gävle bedrivs forskning om stadsmiljöers och urbana naturmiljöers roll för personligt välbefinnande. Vi vill öka förståelsen för arkitekturens, den byggda miljöns och naturmiljöns roll i relation till vilka livsvillkor som skapas.
Med den här specifika enkäten vill vi samla underlag till forskning som syftar till att bättre förstå hur staden fungerar i en krissituation för sina invånare i olika delar av staden. Vi vill se hur olika skeenden kan påverka vår användning av staden, hur vi rör oss och hur det förändrar tillgängligheten till samhälleliga resurser och vårt personliga välbefinnande.
Du får gärna gå in och svara på enkäten flera gånger i takt med att dina vanor förändras över tid.
Dina svar hanteras helt anonymt. Resultaten kommer att redovisas på övergripande nivå och som sammanslagna resultat. Enskilda svar kommer inte kunna urskiljas. Vi är intresserade av att fånga mönster i staden och inte individers beteenden. Data och resultat kommer användas för forskningsändamål inom KTH (inom Tillämpad Stadsbyggnad och Senseable Stockholm Lab), samt Högskolan i Gävle (inom Urban Studio). Respektive kommun som deltar kommer också kunna ta del av ett urval av data från den egna kommunen i analys- och utvecklingssyfte.
Frågor? KTH Ann Legeby, e-post: varstad_covid19@kth.se
https://www.senseablestockholm.org/sv/kartenkat-covid-19/
Starta enkäten genom att trycka på svarta pilen till höger!
Enkäten har stängts 2021-03-25
KTH
VETENSKAP OCH KONST
HÖGSKOLAN I GÄVLE
14.02
30.6.2021

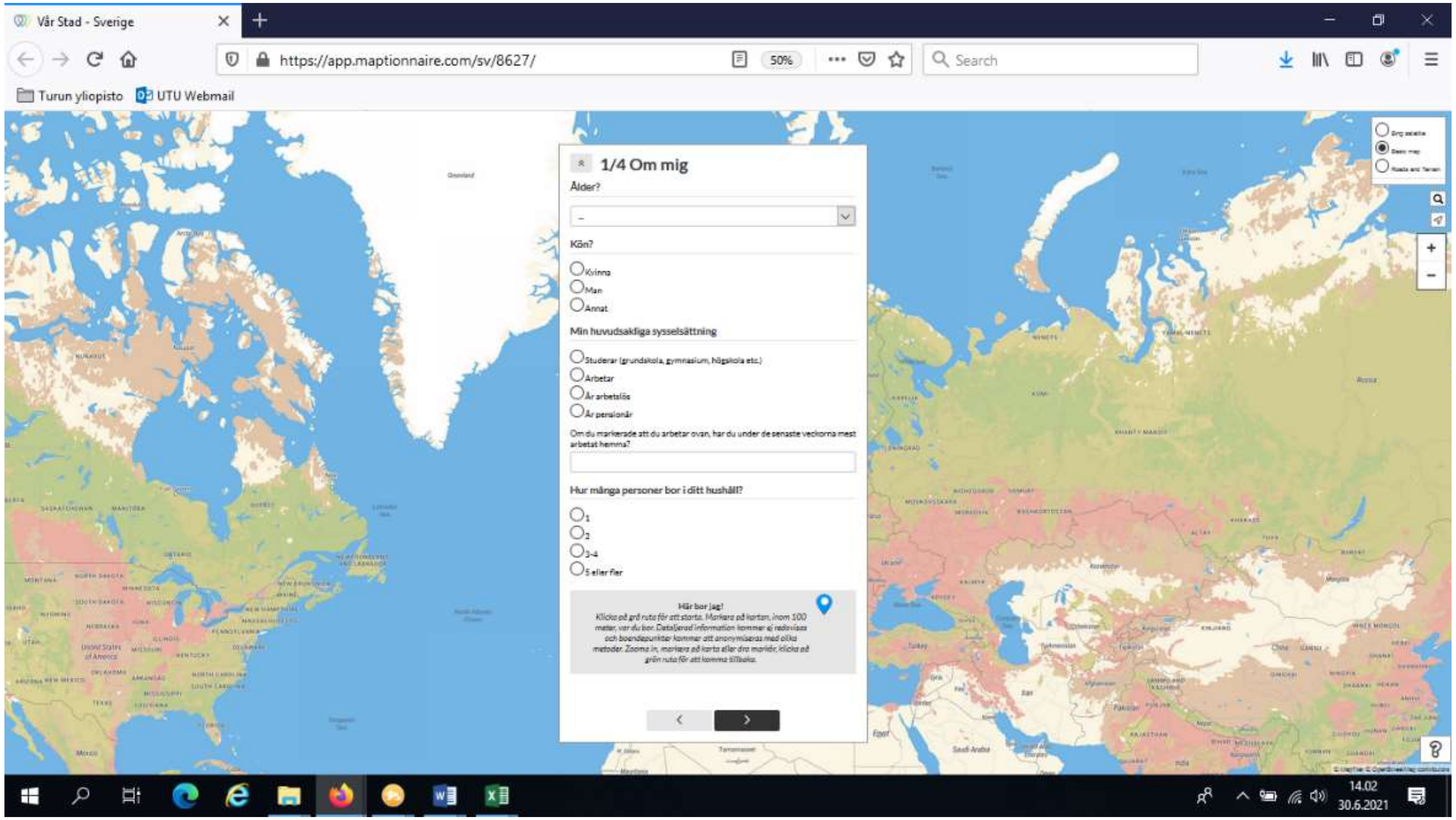
Vår Stad - Sverige
https://app.maptionnaire.com/sv/8627/
Search
Turun yliopisto
UTU Webmail
1/4 Om mig
Ålder?
Kön?
Kvinna
Man
Annat
Min huvudsakliga sysselsättning
Studerar (grundskola, gymnasium, högskola etc.)
Arbetar
Är arbetslös
Är pensionär
Om du markerade att du arbetar ovan, har du under de senaste veckorna mest arbetat hemma?
Hur många personer bor i ditt hushåll?
1
2
3-4
5 eller fler
Här bor jag!
Klicka på grå ruta för att starta. Markera på kartan, inom 100 meter, var du bor. Detaljerad information kommer ej redovisas och boendepunkter kommer att anonymiseras med olika metoder. Zooma in, markera på karta eller dra markör, klicka på grön ruta för att komma tillbaka.
14.02
30.6.2021

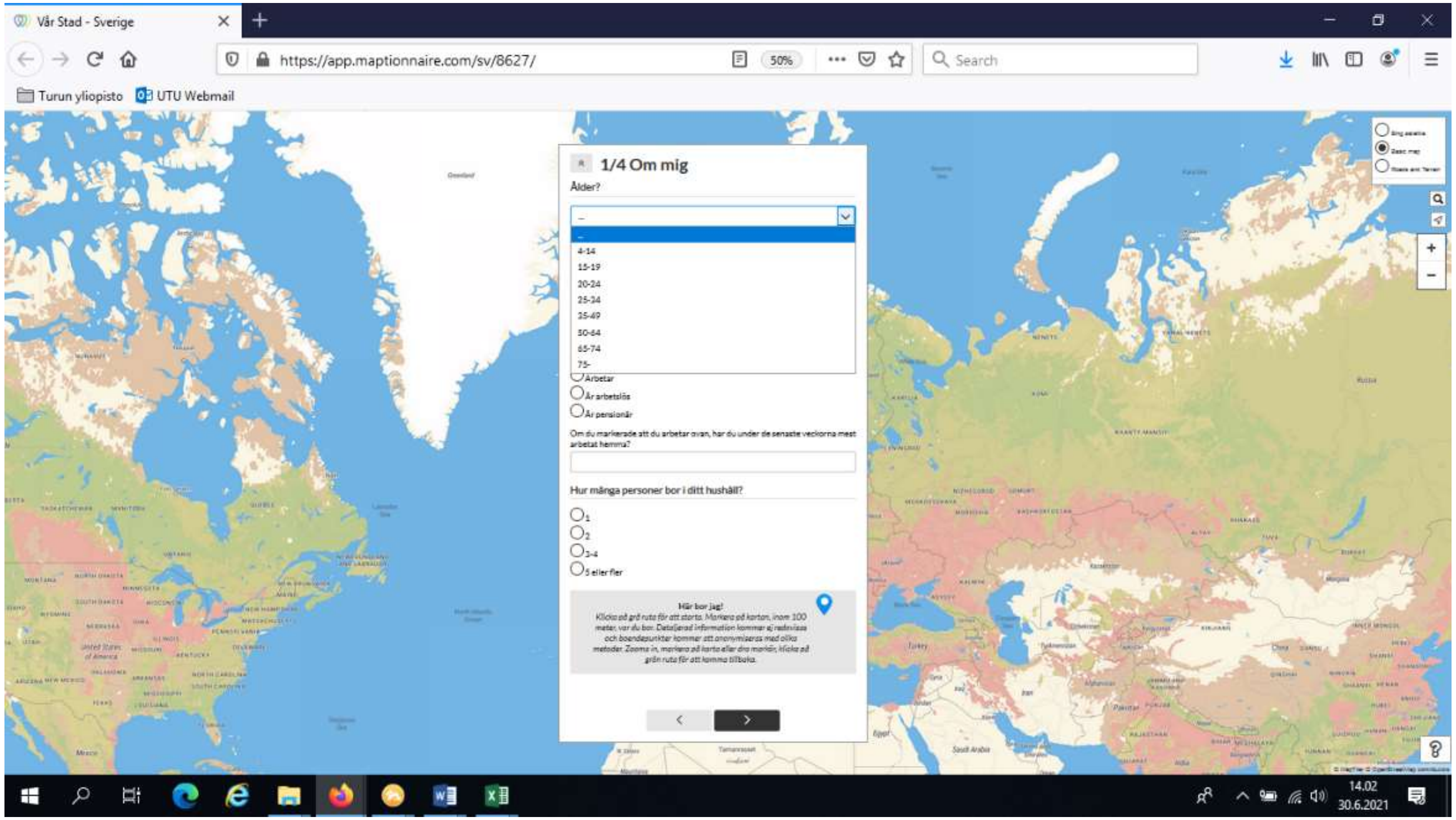
Vår Stad - Sverige
https://app.maptionnaire.com/sv/8627/
50%
Search
Turun yliopisto
UTU Webmail
1/4 Om mig
Ålder?
–
4-14
15-19
20-24
25-34
35-49
50-64
65-74
75-
Arbetar
Är arbetslös
Är pensionär
Om du markerade att du arbetar ovan, har du under de senaste veckorna mest arbetat hemma?
Hur många personer bor i ditt hushåll?
1
2
3-4
5 eller fler
Här bor jag!
Klicka på grå ruta för att starta. Markera på kartan, inom 100 meter, var du bor. Detaljerad information kommer ej redovisas och boendepunkter kommer att anonymiseras med olika metoder. Zooma in, markera på karta eller dra markör, klicka på grön ruta för att komma tillbaka.
14.02
30.6.2021

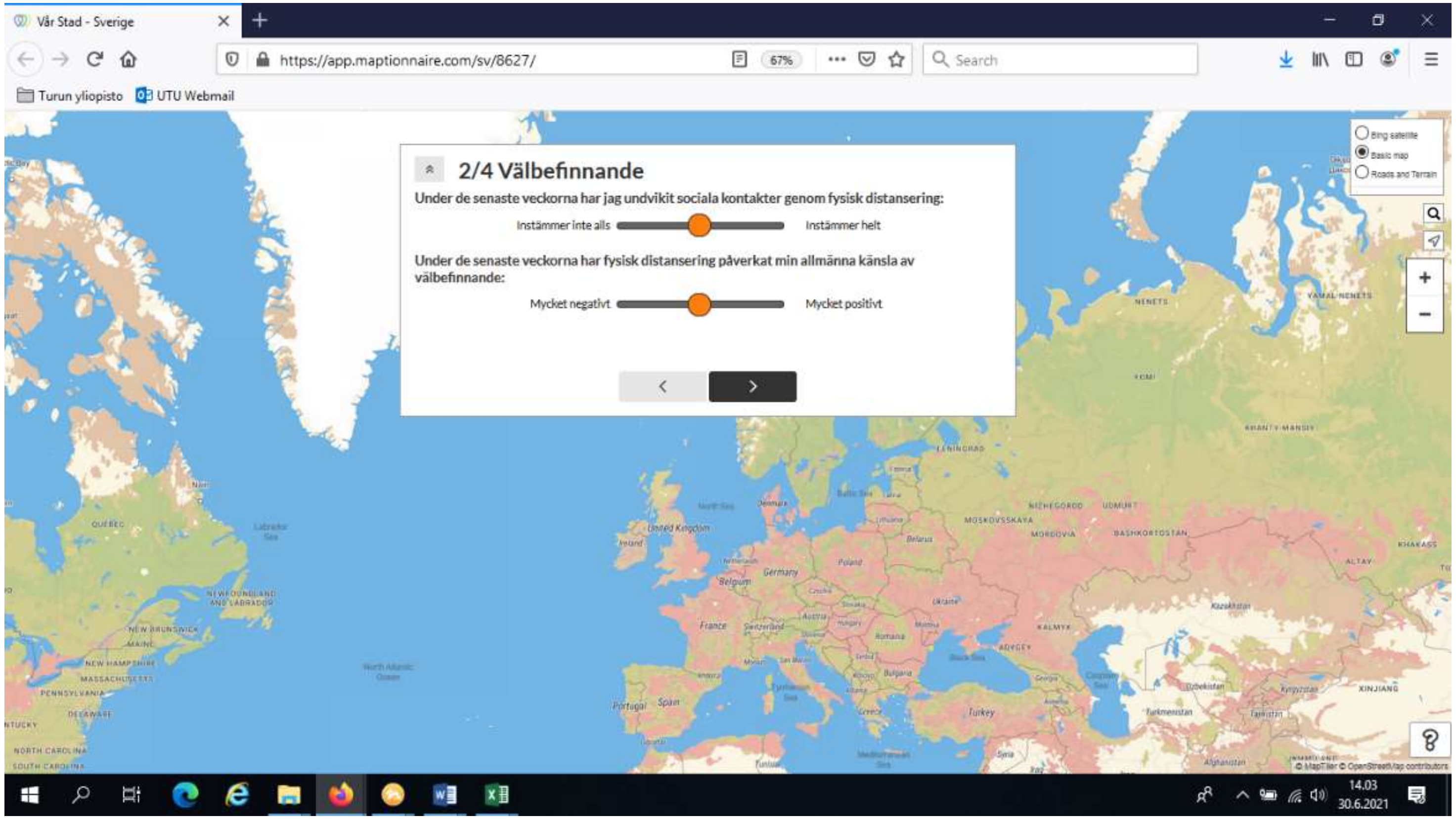

Vår Stad - Sverige
https://app.maptionnaire.com/sv/8627/
Turun yliopisto
UTU Webmail
2/4 Välbefinnande
Under de senaste veckorna har jag undvikit sociala kontakter genom fysisk distansering:
Instämmer inte alls
Instämmer helt
Under de senaste veckorna har fysisk distansering påverkat min allmänna känsla av välbefinnande:
Mycket negativt
Mycket positivt
Bing satellite
Basic map
Roads and Terrain
14.03
30.6.2021

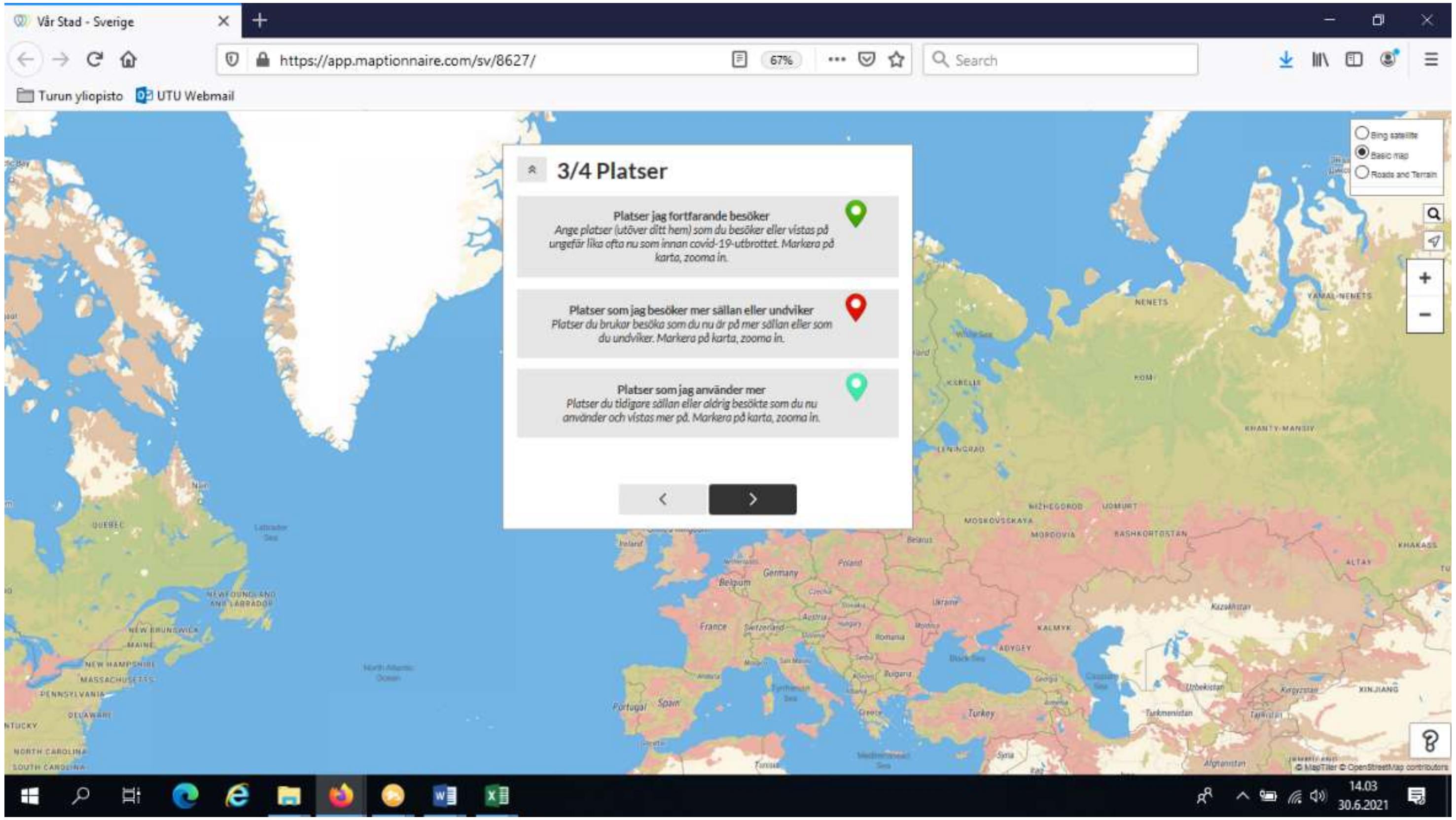
Vår Stad - Sverige
https://app.maptionnaire.com/sv/8627/
Turun yliopisto
UTU Webmail
3/4 Platser
Platser jag fortfarande besöker
Ange platser (utöver ditt hem) som du besöker eller vistas på ungefär lika ofta nu som innan covid-19-utbrottet. Markera på karta, zooma in.
Platser som jag besöker mer sällan eller undviker
Platser du brukar besöka som du nu är på mer sällan eller som du undviker. Markera på karta, zooma in.
Platser som jag använder mer
Platser du tidigare sällan eller aldrig besökte som du nu använder och vistas mer på. Markera på karta, zooma in.
14.03
30.6.2021

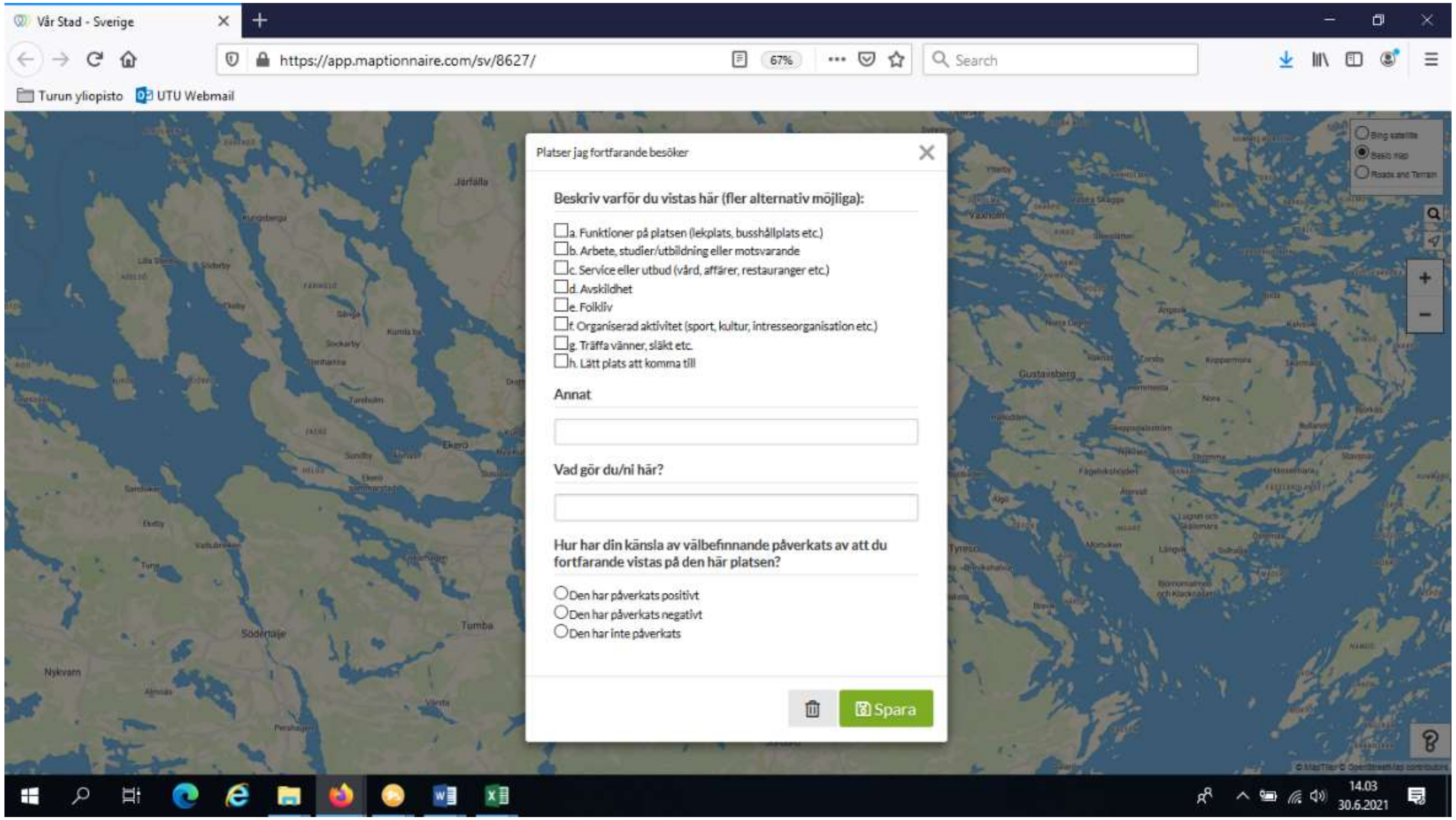
Vår Stad - Sverige
https://app.maptionnaire.com/sv/8627/
Platser jag fortfarande besöker
Beskriv varför du vistas här (fler alternativ möjliga):
a. Funktioner på platsen (lekplats, busshållplats etc.)
b. Arbete, studier/utbildning eller motsvarande
c. Service eller utbud (vård, affärer, restauranger etc.)
d. Avskildhet
e. Folkliv
f. Organiserad aktivitet (sport, kultur, intresseorganisation etc.)
g. Träffa vänner, släkt etc.
h. Lätt plats att komma till
Annat
Vad gör du/ni här?
Hur har din känsla av välbefinnande påverkats av att du fortfarande vistas på den här platsen?
Den har påverkats positivt
Den har påverkats negativt
Den har inte påverkats
Spara

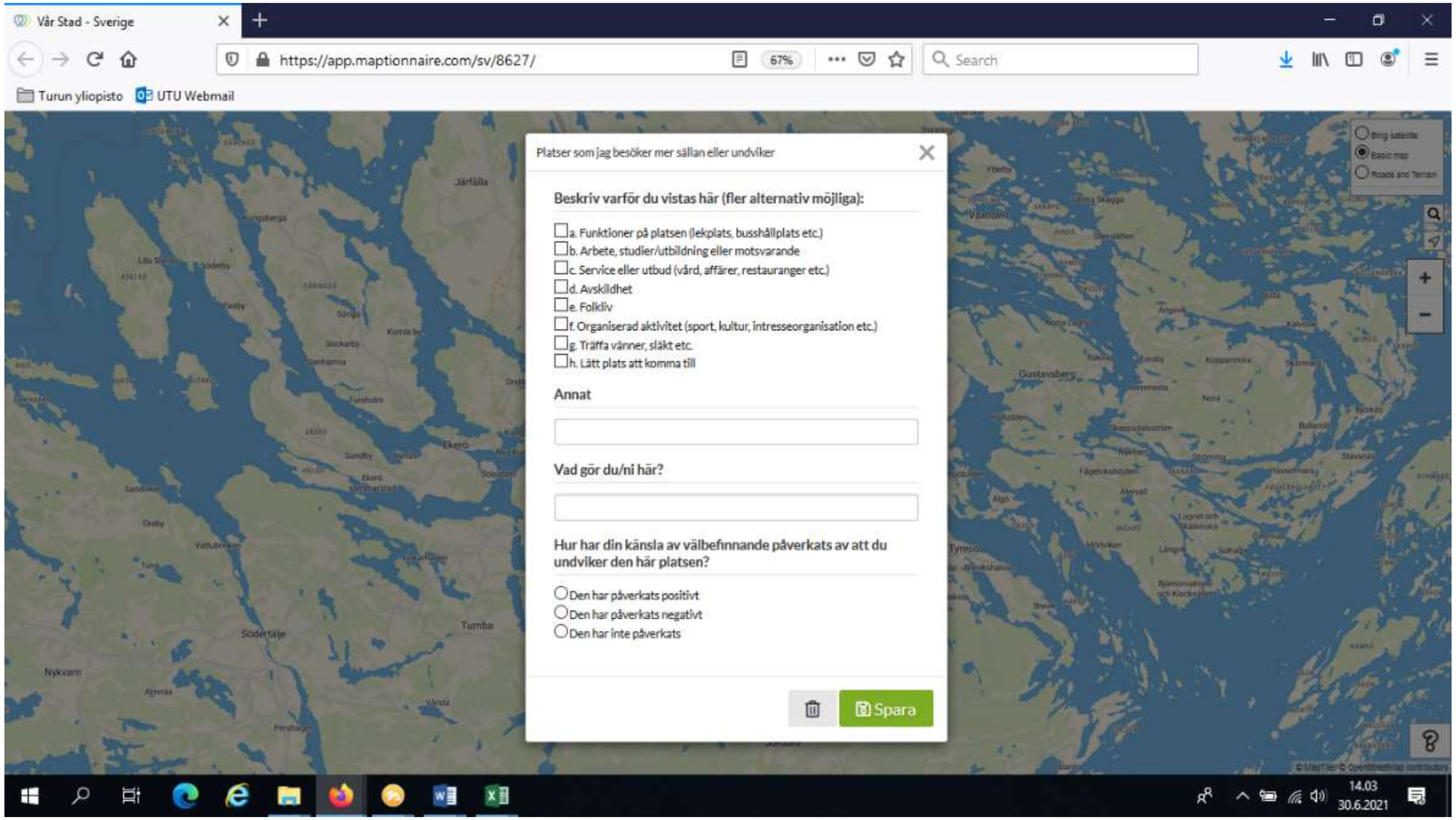
Vår Stad - Sverige
https://app.maptionnaire.com/sv/8627/
Search
Turun yliopisto
UTU Webmail
Platser som jag besöker mer sällan eller undviker
Beskriv varför du vistas här (fler alternativ möjliga):
a. Funktioner på platsen (lekplats, busshållplats etc.)
b. Arbete, studier/utbildning eller motsvarande
c. Service eller utbud (vård, affärer, restauranger etc.)
d. Avskildhet
e. Folkliv
f. Organiserad aktivitet (sport, kultur, intresseorganisation etc.)
g. Träffa vänner, släkt etc.
h. Lätt plats att komma till
Annat
Vad gör du/ni här?
Hur har din känsla av välbefinnande påverkats av att du undviker den här platsen?
Den har påverkats positivt
Den har påverkats negativt
Den har inte påverkats
Spara
14.03
30.6.2021

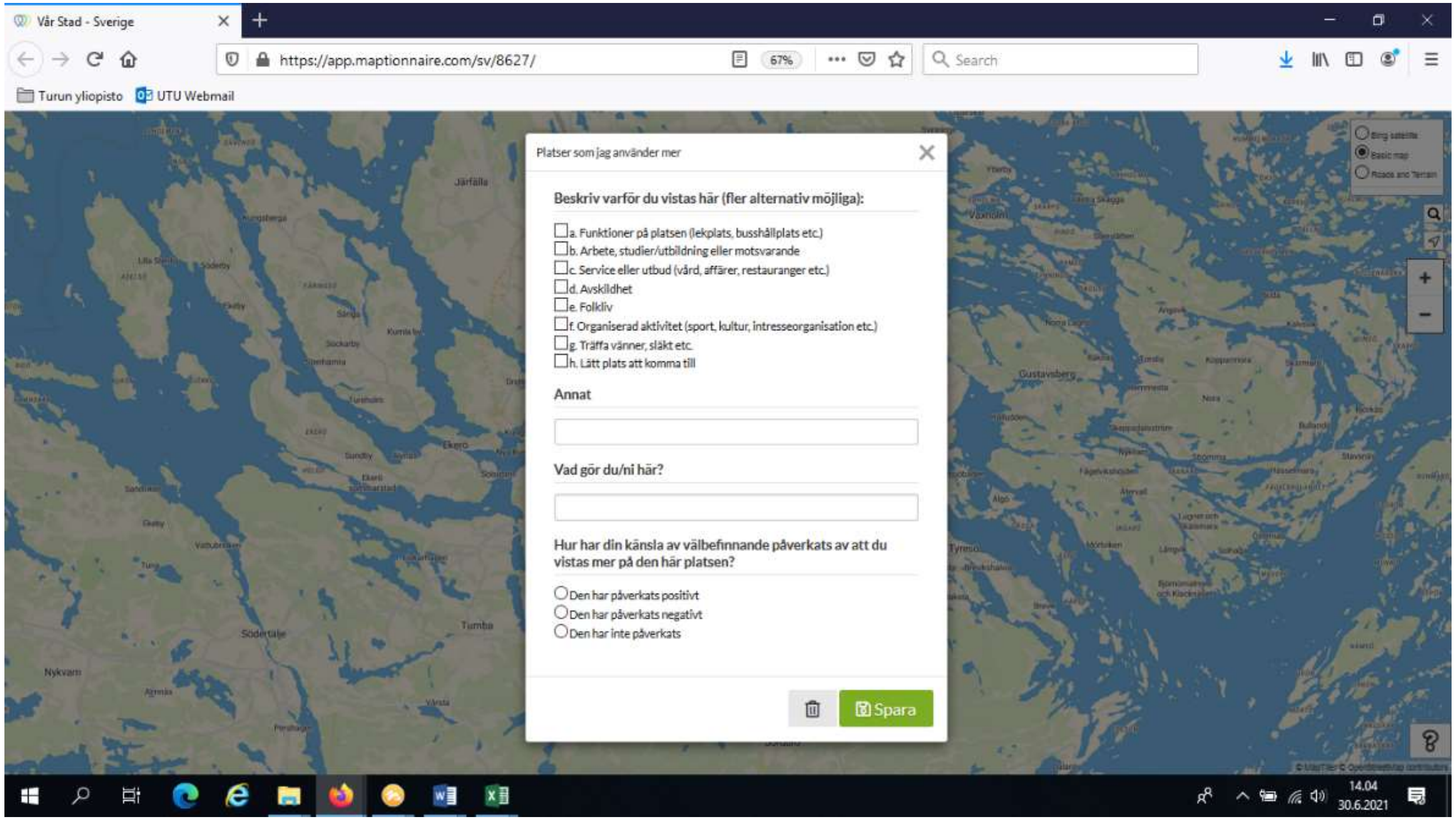
Vår Stad - Sverige
https://app.maptionnaire.com/sv/8627/
Turun yliopisto
UTU Webmail
Platser som jag använder mer
Beskriv varför du vistas här (fler alternativ möjliga):
a. Funktioner på platsen (lekplats, busshållplats etc.)
b. Arbete, studier/utbildning eller motsvarande
c. Service eller utbud (vård, affärer, restauranger etc.)
d. Avskildhet
e. Folkliv
f. Organiserad aktivitet (sport, kultur, intresseorganisation etc.)
g. Träffa vänner, släkt etc.
h. Lätt plats att komma till
Annat
Vad gör du/ni här?
Hur har din känsla av välbefinnande påverkats av att du vistas mer på den här platsen?
Den har påverkats positivt
Den har påverkats negativt
Den har inte påverkats
Spara
14.04
30.6.2021

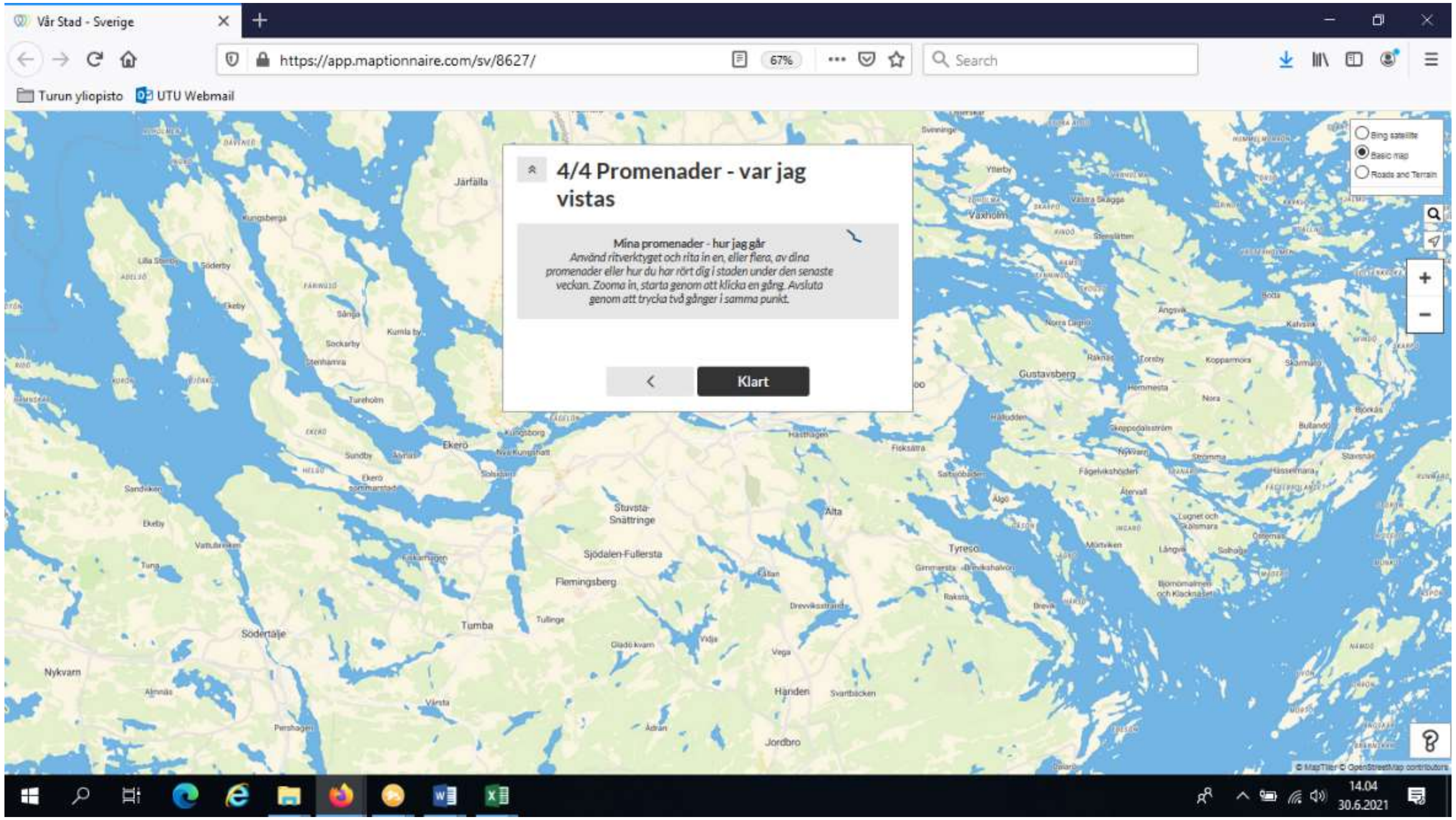
Vår Stad - Sverige
https://app.maptionnaire.com/sv/8627/
Search
Turun yliopisto
UTU Webmail
4/4 Promenader - var jag vistas
Mina promenader - hur jag går
Använd ritverktyget och rita in en, eller flera, av dina promenader eller hur du har rört dig i staden under den senaste veckan. Zooma in, starta genom att klicka en gång. Avsluta genom att trycka två gånger i samma punkt.
Klart
Järfälla
Gustavsberg
Stuvsta-Snättringe
Sjödalen-Fullersta
Flemingsberg
Tumba
Södertälje
Nykvarn
Tyresö
Handen
Jordbro
Vaxholm
Ekerö
14.04
30.6.2021

Supplementary Notes

Supplementary Table 2. Stockholm: Statistics on sample and place of residence. Group differences were assessed with Mann Whitney U test or Kruskal-Wallis test. Significant group differences (p<0.05) are in bold.

**Variable**	**Sample**		**Place of residence**				
	% of respondents in whole sample	% of respondents who mapped home	Mean TCD (%) within 300 m	Mean TCD (%) within 1000 m	Mean ID (%) within 300 m	Mean ID (%) within 1000 m	Median distance (km) to water
**Age (n or p-value)**	*NA*	593	**0.005****	**0.040***	**0.001****	**0.009****	0.798
Young (15–29)		29.0	**13.2**	**15.3**	**42.8**	**36.2**	2.21
Middle-aged (30–64)		62.2	**14.2**	**16.1**	**37.7**	**33.1**	2.19
Elderly (65+)		8.8	**10.9**	**13.4**	**44.7**	**37.4**	2.25
**Gender (n or p-value)**		593	0.357	0.178	0.179	0.455	0.853
Male		33.6	13.0	14.8	41	35.1	2.24
Female		66.4	13.9	16.0	39.2	34.0	2.19
**Occupation (n or p-value)**		593	0.437	0.572	0.351	0.702	0.276
Employed/student		88.2	13.7	15.7	39.9	34.5	2.18
Unemployed		3.0	13.8	16.0	33.3	30.8	2.81
Retired		8.8	12.3	14.5	40.7	34.8	2.24

Supplementary Table 3. Stockholm: Statistics on outdoor recreation sites. Group differences were assessed with Mann Whitney U test or Kruskal-Wallis test. Significant group differences (p<0.05) are in bold.

**Variable**	**All sites**					**Sites with increased visits**				
	% of Sites	Median distance (km) from home	Mean TCD (%) within 300 m	Mean ID (%) within 300 m	Median distance (km) to water	% of Sites	Median distance (km) from home	Mean TCD (%) within 300 m	Mean ID (%) within 300 m	Median distance (km) to water
**Age (n or p-value)**	1381	**0.029***	0.517	0.517	**0.001*****	423	0.079	0.858	0.965	**0.018***
Young (15–29)	31.2	**2.0**	17.1	38.1	**1.4**	40.0	2.3	23.1	25.3	**0.9**
Middle-aged (30–64)	58.9	**2.1**	16.5	39.8	**1.6**	53.5	2.5	23.5	25.8	**1.2**
Elderly (65+)	9.9	**3.0**	17.4	38.3	**1.9**	6.6	3.4	22.2	26.3	**1.9**
**Gender (n or p-value)**	1381	0.059	**0.001*****	**0.001*****	0.483	423	0.102	0.515	0.783	0.885
Male	34.3	2.5	**15.3**	**46.2**	1.5	112	2.8	23.3	26.2	0.9
Female	65.7	2.0	**17.5**	**37.3**	1.5	311	2.3	23.3	25.5	1.2
**Occupation (n or p-value)**	1381	**0.004****	0.67	0.393	**0.009****	423	**0.005****	0.087	0.119	**0.006****
Employed/ student	87.8	**2.1**	16.6	39.4	**1.5**	384	**2.3**	23.1	25.8	**1.1**
Unemployed	2.7	**2.3**	17.0	39.6	**1.0**	9	**5.4**	36	12	**0.1**
Retired	9.5	**3.3**	18.1	36.2	**1.9**	30	**3.6**	21.5	27.9	**1.9**

Supplementary Table 4. Copenhagen: Statistics on sample and place of residence. Group differences were assessed with Mann Whitney U test or Kruskal-Wallis test. Significant group differences (p<0.05) are in bold.

Variable	Sample		Place of residence				
	% of respondents in whole sample	% of respondents who mapped home	Mean TCD (%) within 300 m	Mean TCD (%) within 1000 m	Mean ID (%) within 300 m	Mean ID (%) within 1000 m	Median distance (km) to water
**Age (n or p-value)**	5171	4299	**0.000*** **	**0.000*** **	**0.000*** **	**0.000*** **	**0.000*** **
Young (15–29)	11.3	11.5%	**3.8**	**5.6**	**75.6**	**67.9**	**1.6**
Middle-aged (30–64)	71.9	73.5%	**4.9**	**6.2**	**68.4**	**62.9**	**1.5**
Elderly (65+)	16.8	14.9%	**6.0**	**7.3**	**60.9**	**56.8**	**1.0**
**Gender (n or p-value)**	5213	4302	0.288	0.437	0.175	**0.004** **	0.543
Male	40.5	39.2%	4.8	6.2	67.8	**61.9**	1.4
Female	59.5	60.8%	5.0	6.3	68.3	**62.9**	1.5
**Occupation (n or p-value)**	3090	2686	**0.000*** **	**0.000*** **	**0.000*** **	**0.000*** **	**0.000*** **
Employed/student	79.6	80.4%	**4.8**	**6.2**	**68.7**	**62.8**	**1.5**
Unemployed	5.4	5.5%	**4.6**	**6.3**	**70.7**	**63.9**	**1.6**
Retired	15.0	14.1%	**5.9**	**7.5**	**60.8**	**56.5**	**1.0**
**Working remotely (n or p-value)**	5084	4299	**0.004** **	**0.003** **	**0.000*** **	**0.000*** **	0.101
Yes	81.6	83.6%	**4.9**	**6.2**	**68.6**	**63.0**	1.5
No	18.4	16.4%	**5.3**	**6.7**	**65.4**	**60.1**	1.4
**Children in household (n or p-value)**	3081	2687	0.902	0.915	0.052	**0.038***	0.816
Yes	30.7	31.3%	5.1	6.4	66.9	**61.3**	1.4
No	69.3	68.7%	4.9	6.4	68.3	**62.4**	1.5

Supplementary Table 5. Copenhagen: Statistics on outdoor recreation sites. Group differences were assessed with Mann Whitney U test or Kruskal-Wallis test. Significant group differences (p<0.05) are in bold.

Variable	All sites					Sites with increased visits			
	% of Sites	Median distance (km) from home	Mean TCD (%) within 300 m	Mean ID (%) within 300 m	Median distance (km) to water	% of Sites	Median distance (km) from home	Mean TCD (%) within 300 m	Mean ID (%) within 300 m
**Age (n or p-value)**	7225	**0.000*** **	0.054	**0.000*** **	**0.000*** **	2254	**0.042***	0.417	**0.016***
Young (15–29)	14.0	**1.3**	13.1	**47.9**	**1.3**	15.90%	**1.3**	15.0	**44.4**
Middle-aged (30–64)	75.3	**1.1**	12.7	**45.3**	**1.1**	75.50%	**1.2**	14.0	**43.5**
Elderly (65+)	10.6	**1.0**	12.7	**42.2**	**0.9**	8.60%	**1.2**	14.0	**38.2**
**Gender (n or p-value)**	7217	0.082	0.800	**0.000*** **	**0.017***	2244	0.65	0.768	0.48
Male	33.8	1.1	12.9	**43.6**	**1.1**	30.30%	1.1	14.0	42.4
Female	66.2	1.2	12.8	**46.0**	**1.2**	69.70%	1.2	14.0	43.3
**Occupation (n or p-value)**	5714	**0.000*** **	0.054	**0.001*** **	**0.000*** **	1945	0.936	0.097	**0.004** **
Employed/student	83.9	**1.2**	13.1	**44.5**	**1.1**	84.40%	1.2	14.0	**42.8**
Unemployed	6.1	**1.2**	12.4	**47.6**	**1.3**	7.40%	1.2	12.9	**47.9**
Retired	10.0	**1.1**	13.1	**41.1**	**0.8**	8.20%	1.2	14.9	**37.8**
**Working remotely (n or p-value)**	7265	**0.000*** **	0.506	0.685	**0.000*** **	2261	**0.046***	0.742	0.388
Yes	88.5	**1.2**	12.9	45.4	**1.2**	92.30%	**1.2**	13.9	43.1
No	11.5	**1.0**	12.1	45.1	**1.0**	7.70%	**1.1**	13.4	45.1
**Children in household (n or p-value)**	5808	**0.000*** **	**0.000*** **	**0.000*** **	0.058	1958	**0.009** **	**0.001*** **	**0.002** **
Yes	36.0	**1.1**	**11.7**	**46.6**	1.1	36.80%	**1.1**	**12.4**	**45.5**
No	64.0	**1.3**	**13.7**	**43.4**	1.1	63.20%	**1.3**	**15.0**	**41.6**

Supplementary Table 6. Helsinki: Statistics on sample and place of residence. Group differences were assessed with Mann Whitney U test or Kruskal-Wallis test. Significant group differences (p<0.05) are in bold.

**Variable**	**Sample**		**Place of residence**				
	% of respondents in whole sample	% of respondents who mapped home	Mean TCD (%) within 300 m	Mean TCD (%) within 1000 m	Mean ID (%) within 300 m	Mean ID (%) within 1000 m	Median distance (km) to water
**Age (n or p-value)**	NA	420	0.440	0.136	0.238	**0.011***	0.511
Young (15–29)		13.1	14.5	15.6	47.5	**42.2**	1.82
Middle-aged (30–64)		76.2	16.3	18.3	42.7	**35.7**	2.24
Elderly (65+)		10.7	16.6	18.9	44.2	**36.9**	2.06
**Gender (n or p-value)**		417	0.390	0.108	0.146	0.061	0.790
Male		43.2	17.0	17.5	45.0	35.1	2.24
Female		56.8	15.4	18.8	41.4	37.7	2.12
**Occupation (n or p-value)**		345	0.474	0.056	0.284	**0.050***	0.971
Employed/student		74.8	16.5	18.0	42.3	**36.5**	2.19
Unemployed		11.6	14.1	16.1	45.4	**38.0**	2.16
Retired		13.6	17.0	20.8	43.3	**33.0**	2.15
**Working remotely (n or p-value)**		271	0.507	0.087	0.495	0.33	0.070
Yes		64.6	15.9	17.1	44.1	37.7	1.97
No		35.4	16.5	19.0	40.9	35.3	2.51
**Children in household (n or p-value)**		410	**0.001****	**0.000*****	**0.000*****	**0.000*****	0.281
Yes		32.4	**18.7**	**20.2**	**37.9**	**32.6**	2.41
No		67.6	**14.7**	**16.8**	**46.5**	**38.9**	2.05

Supplementary Table 7. Helsinki: Statistics on outdoor recreation sites. Group differences were assessed with Mann Whitney U test or Kruskal-Wallis test. Significant group differences ($p<0.05$) are in bold.

**Variable**	**All sites**				
	% of Sites	Median distance (km) from home	Mean TCD (%) within 300 m	Mean ID (%) within 300 m	Median distance (km) to water
**Age (n or p-value)**	1697.0	0.638	0.143	0.116	**0.000*****
Young (15–29)	16,91	3.0	29.2	29.2	**2.1**
Middle-aged (30–64)	71,24	2.9	29.2	29.0	**1.9**
Elderly (65+)	11,84	3.1	33.7	33.7	**1.4**
**Gender (n or p-value)**	1692	0.319	0.263	0.251	0.812
Male	40.8	2.8	28.4	28.2	1.89
Female	59.2	3.0	30.7	30.5	1.82
**Occupation (n or p-value)**	1536	0.993	0.428	0.334	0.406
Employed/student	72.3	3.0	29.0	28.9	1.9
Unemployed	14.3	2.8	31.1	31.2	1.9
Retired	13.5	2.8	31.7	31.7	1.7
**Working remotely (n or p-value)**	1222	**0.002****	0.236	0.212	0.105
Yes	63.9	**2.7**	29.1	28.9	1.8
No	36.1	**3.4**	29.7	29.4	2.1
**Children in household (n or p-value)**	1658	0.396	**0.000*****	**0.000*****	**0.017***
Yes	32.6	3.0	**26.5**	**26.2**	**2.1**
No	67.4	3.0	**31.7**	**31.5**	**1.7**

Supplementary Table 8. Turku: Statistics on sample and place of residence. Group differences were assessed with Mann Whitney U test or Kruskal-Wallis test. Significant group differences (p<0.05) are in bold.

Variable	Sample		Place of residence				
	% of respondents in whole sample	% of respondents who mapped home	Mean TCD (%) within 300 m	Mean TCD (%) within 1000 m	Mean ID (%) within 300 m	Mean ID (%) within 1000 m	Median distance (km) to water
**Age (no of cases/Sig.)**	717	662	**0.000*** **	**0.000*** **	**0.000*** **	**0.000*** **	**0.001*** **
Young (15–29)	19.9	20.8	**12.7**	**14.3**	**45.9**	**41.0**	**1.1**
Middle-aged (30–64)	70.0	70.2	**17.6**	**18.8**	**37.6**	**34.3**	**1.8**
Elderly (65+)	10.0	8.9	**17.6**	**17.9**	**40.7**	**37.9**	**1.8**
**Gender (no of cases/Sig.)**	713	663	0.321	0.270	0.178	0.080	0.117
Male	27.1	25.3	17.0	18.2	38.1	34.2	1.7
Female	72.9	74.7	16.4	17.6	40.3	36.8	1.5
**Occupation (no of cases/Sig.)**	568	523	0.827	0.922	0.971	0.768	0.513
Employed/student	80.6	81.8	16.5	17.7	39.2	35.6	1.6
Unemployed	5.5	5.5	17.2	18.6	40.5	35.4	1.4
Retired	13.9	12.6	17.4	18.1	39.7	37.7	1.1
**Working remotely (no of cases/Sig.)**	728	428	**0.008** **	**0.002** **	**0.013***	**0.005***	**0.000*** **
Dist. work (Yes)	73.5	79.7	**15.8**	**17.0**	**40.4**	**36.7**	**1.3**
Dist. work (No)	26.5	20.3	**19.4**	**20.6**	**34.8**	**31.3**	**2.2**
**Children in household (no of cases/Sig.)**	361	329	**0.000*** **	**0.000*** **	**0.000*** **	**0.000*** **	**0.002** **
Yes	54.0	51.7	**20.0**	**20.4**	**32.9**	**30.8**	**1.9**
No	46.0	48.3	**13.8**	**16.3**	**43.1**	**39.0**	**1.3**

Supplementary Table 9. Turku: Statistics on outdoor recreation sites. Group differences were assessed with Mann Whitney U test or Kruskal-Wallis test. Significant group differences (p<0.05) are in bold.

**Variable**	**All sites**					**Sites with increased visits**			
	% of Sites	Median distance (km) from home	Mean TCD (%) within 300 m	Mean ID (%) within 300 m	Median distance (km) to water	% of Sites	Median distance (km) from home	Mean TCD (%) within 300 m	Mean ID (%) within 300 m
**Age (n or p-value)**	2244	0.063	**0.002****	**0.000*****	0.757	761	**0.000*****	0.211	**0.003*****
Young (15–29)	19.7	3.6	**23.6**	**22.3**	1.3	22.5	**3.7**	25.9	**19.7**
Middle-aged (30–64)	72.2	3.5	**26.4**	**18.0**	1.2	68.1	**2.6**	26.8	**18.0**
Elderly (65+)	8.1	2.8	**24.6**	**22.8**	1.1	9.5	**2.7**	24.0	**25.9**
**Gender (n or p-value)**	2232	**0.006****	**0.034***	0.105	**0.011***	757	0.419	0.075	0.847
Male	25.6	**3.8**	**27.0**	17.6	**1.8**	76.9	2.8	28.3	18.8
Female	74.5	**3.2**	**25.2**	20.0	**1.1**	23.1	2.9	25.7	19.2
**Occupation (n or p-value)**	1906	**0.016***	**0.002****	**0.000*****	0.149	671	**0.004*****	0.083	**0.007*****
Employed/student	82.4	**3.6**	**25.6**	**18.9**	1.2	80.8	**2.8**	26.1	**18.9**
Unemployed	5.9	**4.8**	**29.1**	**14.8**	2.2	7.3	**5.9**	31.5	**13.3**
Retired	11.8	**3.0**	**23.6**	**22.8**	0.9	11.9	**2.8**	24.1	**23.5**
**Working remotely (n or p-value)**	1570	**0.024***	**0.000*****	0.174	**0.002****	767	**0.036***	**0.003*****	0.680
Yes	80.1	**3.3**	**24.8**	19.8	**1.1**	79.4	**2.7**	**25.4**	19.5
No	19.9	**3.9**	**29.1**	17.8	**1.5**	20.6	**3.4**	**29.7**	17.5
**Children in household (n or p-value)**	1252	**0.045***	**0.000*****	**0.000*****	**0.017***	449	0.588	**0.002*****	**0.001*****
Yes	43.5	**2.9**	**27.8**	**16.6**	**1.3**	56.6	2.5	**28.4**	**16.7**
No	56.5	**3.5**	**23.2**	**23.1**	**1.1**	43.4	2.6	**24.0**	**23.6**

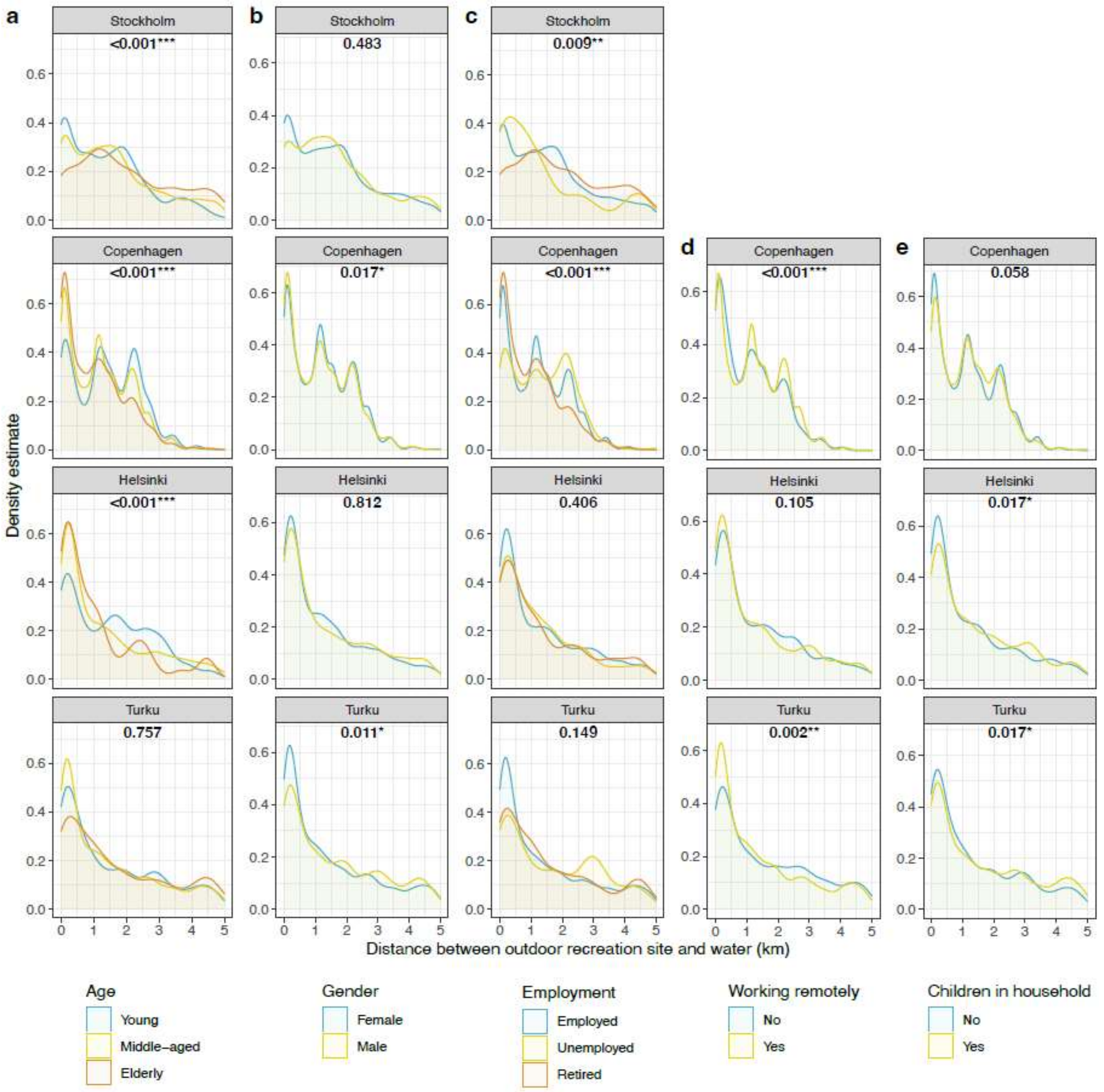

Supplementary Figure 2. Distance between outdoor recreation sites and water elements. Walking distance in km through the street network is given on x axis and kernel density estimates are given on y axis. Panels show data broken down by city and (a) age, (b) gender, (c) employment, (d) remote working, and (e) children in household. The numbers in each plot correspond to the p-value for the Mann Whitney U-test or Kruskal-Wallis test for differences between groups shown in the plot. See also Supplementary Tables 3, 5, 7 and 9 on statistical results as tables.

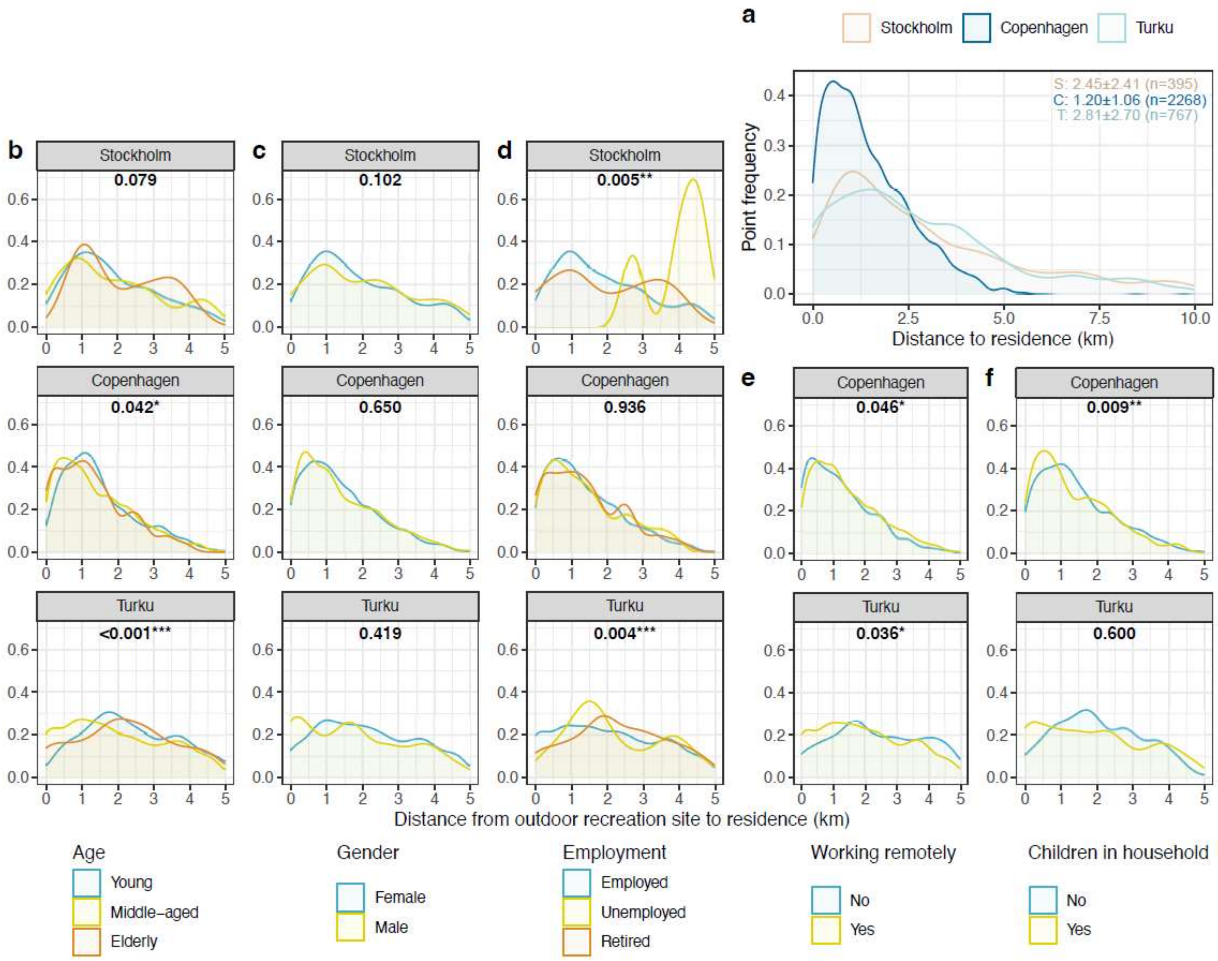

Supplementary Figure 3. Distance between respondent place of residence and outdoor recreation sites *visited more frequently* during COVID-19. Walking distance in km through the street network is given on x axis and data point density estimates are given on y axis. (a) Data grouped by city, with median±median absolute deviation (MAD) and number of outdoor recreation sites for each city in the upper right corner. Remaining panels show data broken down by city and (b) age, (c) gender, (d) employment, (e) remote working, and (f) children in household. For panels b–f, the numbers in each plot correspond to the p-value for the Mann Whitney U-test or Kruskal-Wallis test for differences between groups shown in the plot. See also Supplementary Tables 3, 5, 7 and 9 on statistical results as tables.

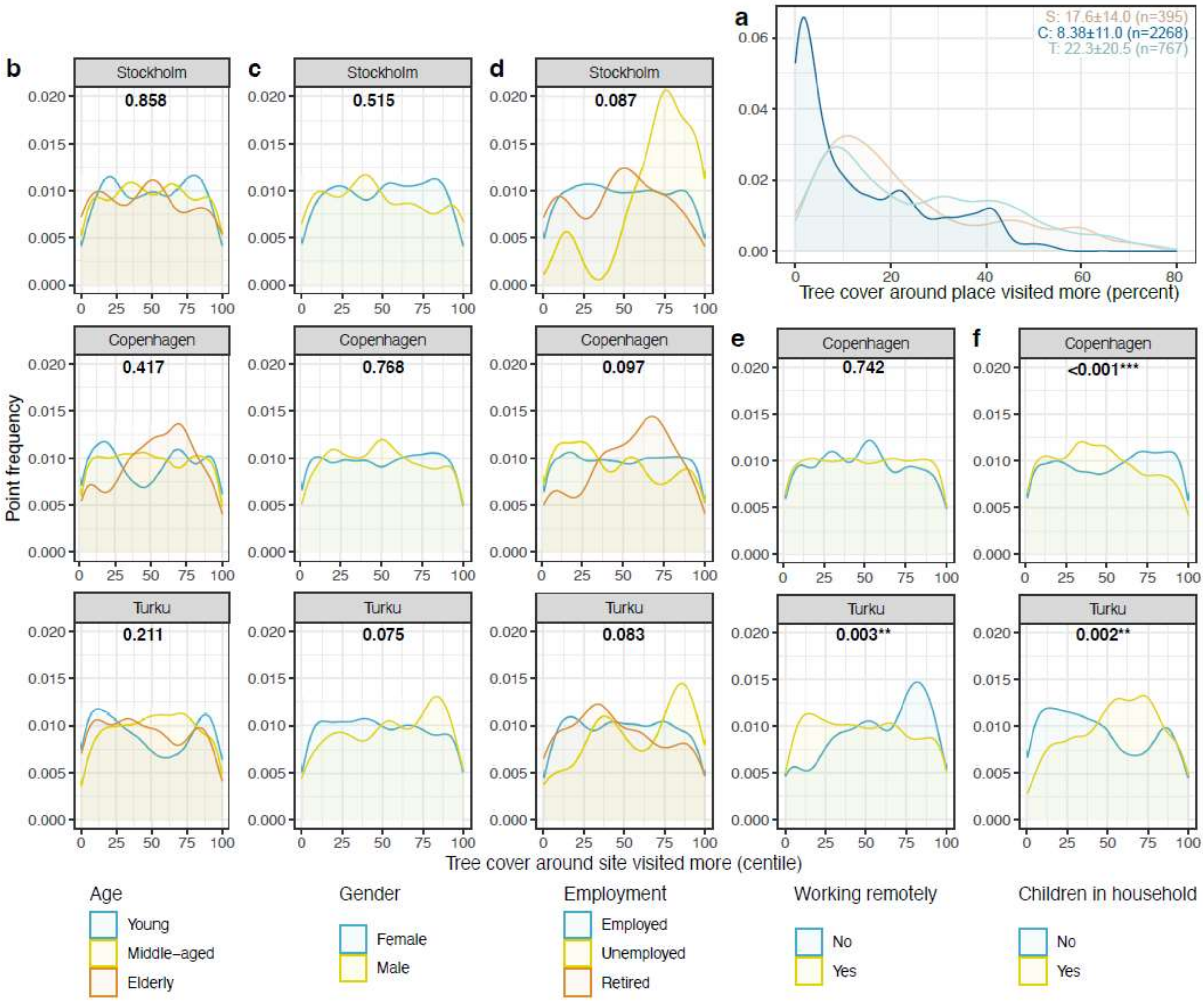

Supplementary Figure 4. Tree cover density (TCD) at 300 m distance around the outdoor recreation sites *visited more frequently* during COVID-19. (a) Percent tree cover is given on the x axis and data point density estimates are given on the y axis. The data are grouped by city, with median±MAD and number of outdoor recreation sites for each city in the upper right corner. Remaining panels show data broken down by city and (b) age, (c) gender, (d) employment, (e) remote working, and (f) children in household. For panels b–f, the numbers in each plot correspond to the p-value for the Mann Whitney U-test or Kruskal-Wallis test for differences between groups shown in the plot. X axes show TCD in centlies of rank order, to illustrate group differences as estimated by the tests used. See also Supplementary Tables 3, 5, 7 and 9 on statistical results as tables.

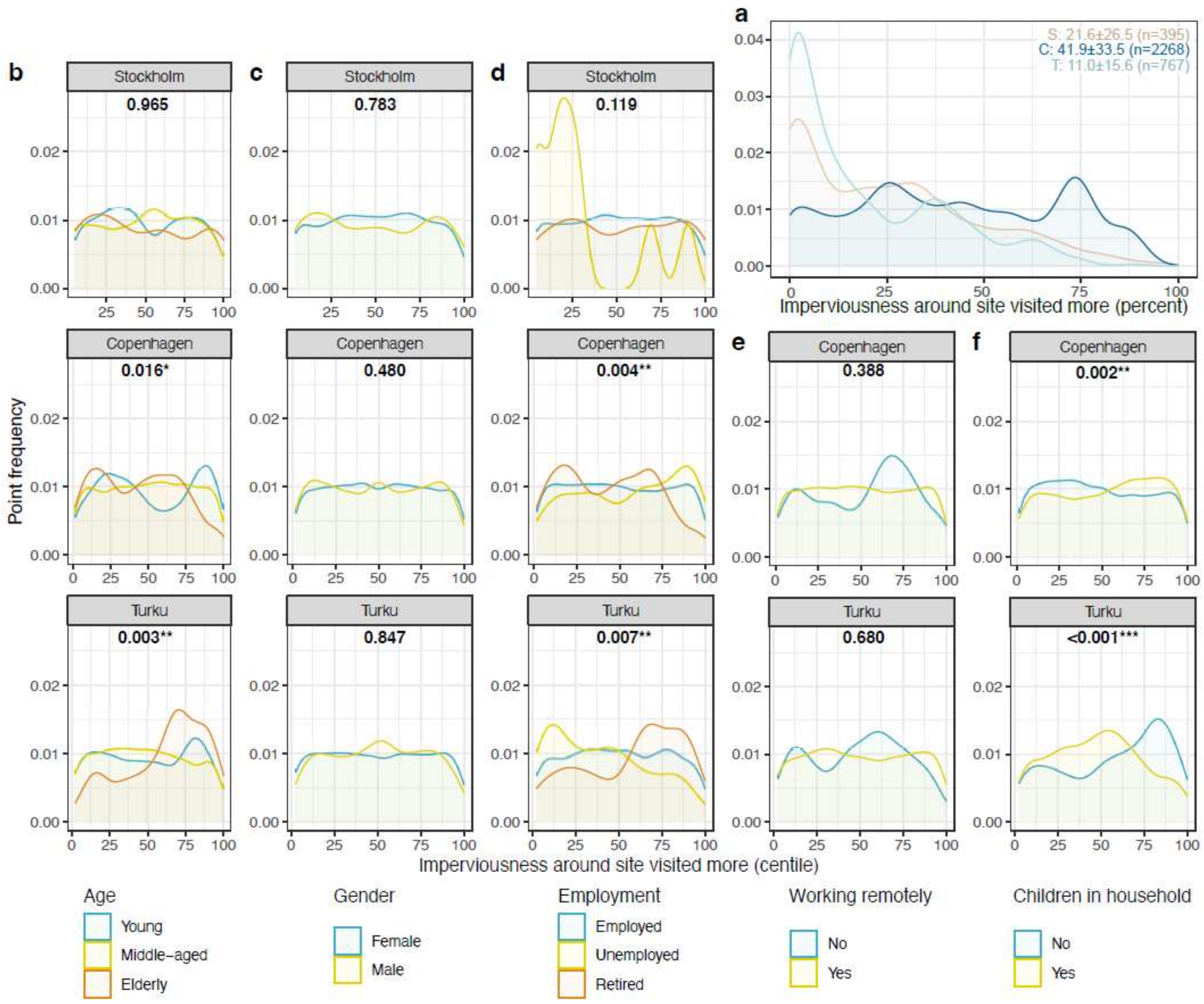

Supplementary Figure 5. Imperviousness density (ID) at 300 m distance around the outdoor recreation sites *visited more frequently* during COVID-19. (a) Percent imperviousness is given on the x axis and data point density estimates are given on the y axis. The data are grouped by city, with median±MAD and number of outdoor recreation sites for each city in the upper right corner. Remaining panels show data broken down by city and (b) age, (c) gender, (d) employment, (e) remote working, and (f) children in household. For panels b–f, the numbers in each plot correspond to the p-value for the Mann Whitney U-test or Kruskal-Wallis test for differences between groups shown in the plot. X axes show ID in centlies of rank order, to illustrate group differences as estimated by the tests used. See also Supplementary Tables 3, 5, 7 and 9 on statistical results as tables.

Tree cover density and imperviousness density (at residence)

In all cities, at 300 m distance from respondents' residence, the tree cover density was lower than at the 1000 m distance, and the imperviousness density, vice versa, was higher closer to residence (Fig. 8 vs. Supplementary Fig. 6).

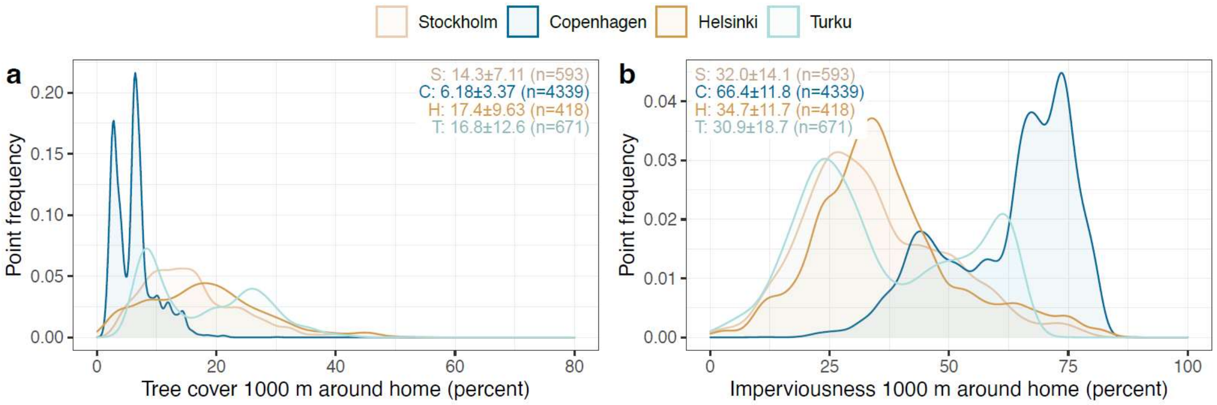

Supplementary Figure 6. Tree cover density (TCD) and imperviousness density (ID) within 1000 m around the respondent's place of residence. X axes show (a) percent tree cover and (b) percent imperviousness surface around the respondent's place of residence at 300 m distance, while y axis show data point density estimates. The data are grouped by city, with median±MAD and number of outdoor recreation sites for each city in the upper corners.

In terms of tree cover and imperviousness density, most commonly the differences across cities in the socio-demographic variables are found for respondent age (all cities indicate stat. sig., except Helsinki where only ID at 1000 m stat. sig.) (Supplementary Figs. 7–10). Young people differed from other groups and live in the surroundings with the lowest tree cover and the highest imperviousness density, observed both at 300 m and 1000 m distance in Copenhagen (at 300 m mean TCD 3.8 %, 1.1–2.2 % lower, mean ID 75.6 %, 7.2–14.7 % higher, TCD and ID *p*<0.001***), Helsinki (at 300 m mean TCD 14.5 %, 1.8–2.1 % lower, mean ID 47.5 %, 4.8–3.3 % higher, TCD and ID not sig.), and Turku (at 300 m mean TCD 12.7 %, 4.9 % lower, mean ID 45.9 %, 8.3–5.2 % higher, TCD and ID *p*<0.001***) (Supplementary Figs. 7–10). Then again, in Stockholm, residence of elderly people was related to the lowest tree cover and highest imperviousness density (at 300 m mean TCD 10.9 %, 2.3–3.3 % lower, mean ID 44.7 %, 1.9–7.0 % higher). In contrast to other cities, middle-aged people in Stockholm lived in the surroundings with the highest tree cover and lowest imperviousness density (TCD at 300 m 14.2 %, *p*=0.005**, 1000 m 16.1 %, *p*=0.040*; ID at 300 m 37.7 %, *p*<0.001***, 1000 m 33.1 %, *p*=0.009**).

No statistically significant differences are observed between the genders in any city, except for imperviousness density in Copenhagen, which was for females 62.9 %, i.e. 1.0 % higher at 1000 m distance around the residence compared to men (*p*=0.004**) (Supplementary Fig. 10). In terms of employment, statistically significant differences are observed only in Copenhagen, where retired people lived in surroundings with higher tree cover (TCD at 300 m 5.9 %, 1.1–1.3% higher) and lower imperviousness density (ID at 300 m 60.8 %, 3.1–9.9 %) compared to employed or

unemployed (TCD and ID at 300 and 1000 m all $p<0.001$***) (Supplementary Figs. 7C–10C). Helsinki shows similar direction of results but only one statistically significant difference between the groups (for the retired mean ID at 1000 m 33.0 %, 3.5–5.0 % lower, $p=0.050$*) (Supplementary Fig. 10C).

Those who shifted to remote working due to COVID-19 lived in surroundings with lower tree cover and higher imperviousness density, and the difference between groups indicates statistical significance in Copenhagen (at 300 m mean TCD 4.9 %, 0.4 % lower, mean ID 68.6 %, 3.4 % higher; TCD at 300 m $p=0.004$**, 1000 m $p=0.003$**; ID at 300 and 1000 m $p<0.001$***) and Turku (at 300 m mean TCD 15.8 %, 3.6 % lower, mean ID 40.4 %, 5.6 % higher; TCD at 300 m $p=0.008$** and 1000 m $p=0.002$**; ID at 300 m $p=0.013$* and 1000 m $p=0.005$**) but not in Helsinki (Supplementary Figs. 7D–10D).

Households with children lived in the surroundings with higher tree cover and lower impervious density, and the results point to strong significant differences compared to those respondents not having children in the Finnish cities (at 300 m in Turku mean TCD 20.0 %, 6.2 % higher, mean ID 32.9 %, 10.2 % lower; in Helsinki mean TCD 18.7 %, 4.0 % higher, mean ID 37.9 %, 8.6 % lower, TCD and ID all $p<0.001$) but not in Copenhagen (Supplementary Figs. 7E–8E).

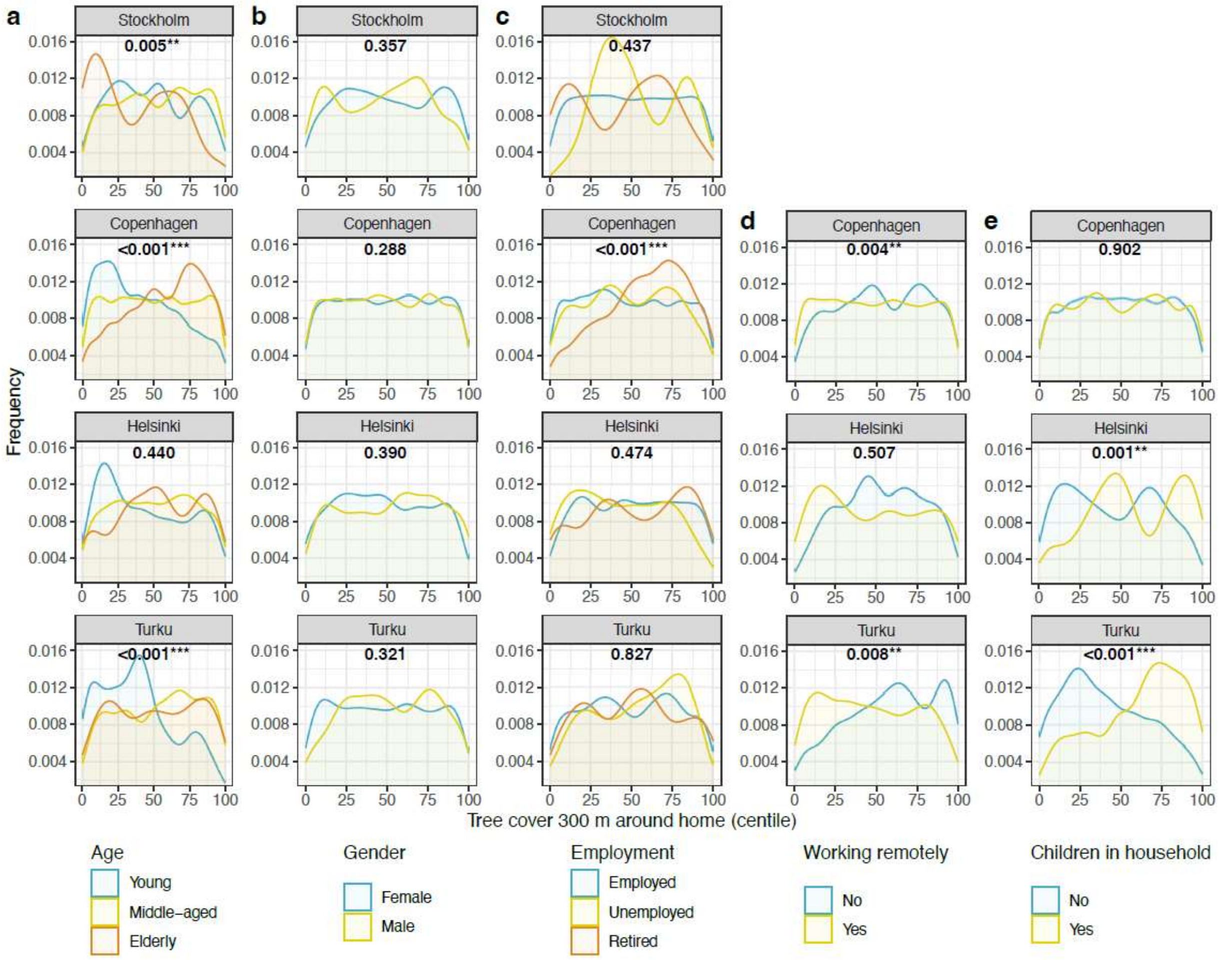

Supplementary Figure 7. Tree cover density (TCD) within 300 m buffer distance around the place of residence. Data are broken down in each city for age (a), gender (b), employment (c), remote working (d), and children in household (e). The numbers in each plot correspond to the p-value for the Mann Whitney U-test or Kruskal-Wallis test for differences between groups shown in the plot. X axes show TCD in centlies of rank order, to illustrate group differences as estimated by the tests used (see Fig. 4 for absolute values in each city). See also Supplementary Tables 2, 4, 6 and 8 on statistical results as tables.

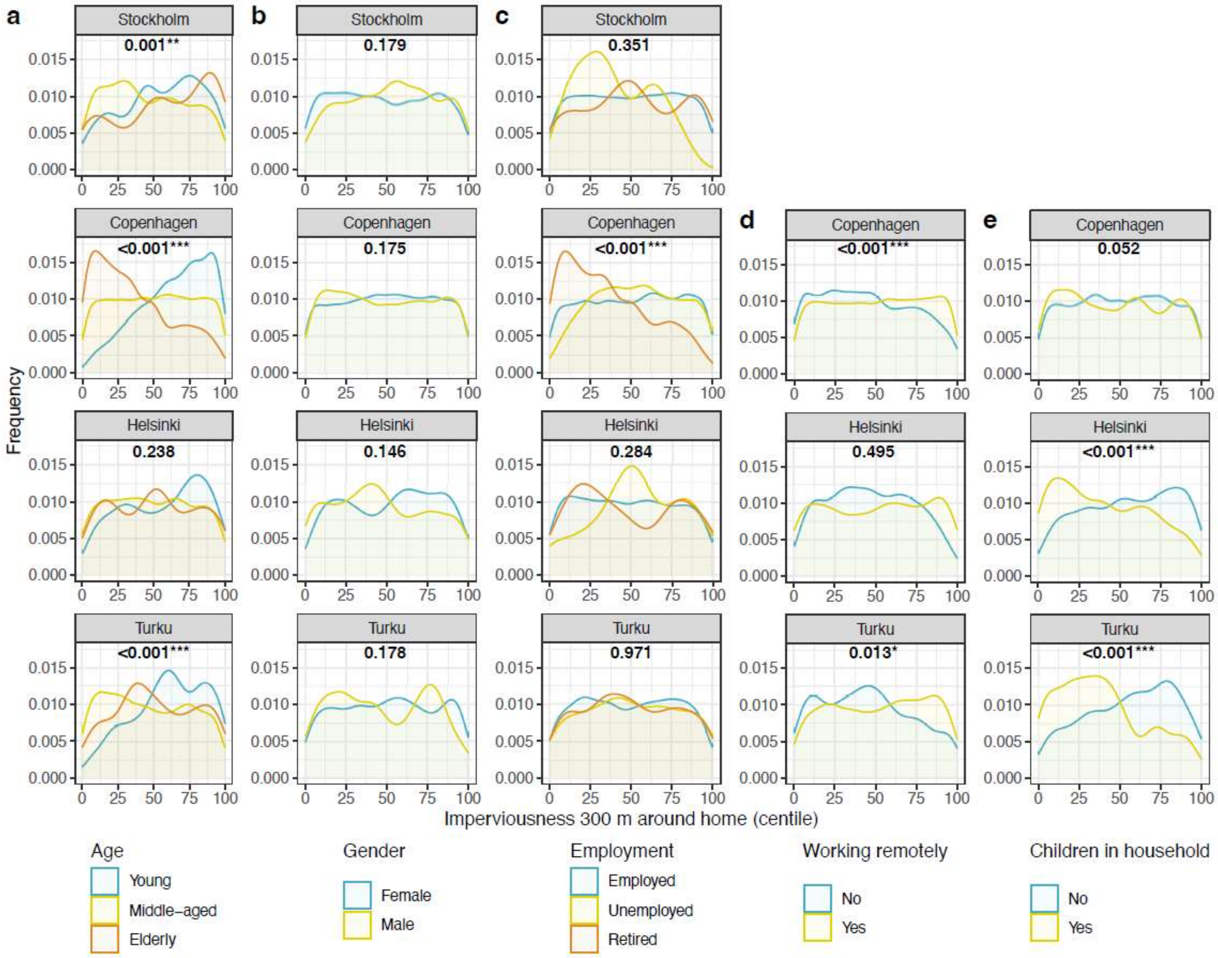

Supplementary Figure 8. Imperviousness density (ID) within 300 m buffer distance around the place of residence. Data are broken down in each city for age (a), gender (b), employment (c), remote working (d), and children in household (e). The numbers in each plot correspond to the p-value for the Mann Whitney U-test or Kruskal-Wallis test for differences between groups shown in the plot. X axes show TCD in centlies of rank order, to illustrate group differences as estimated by the tests used (see Fig. 4 for absolute values in each city). See also Supplementary Tables 2, 4, 6 and 8 on statistical results as tables.

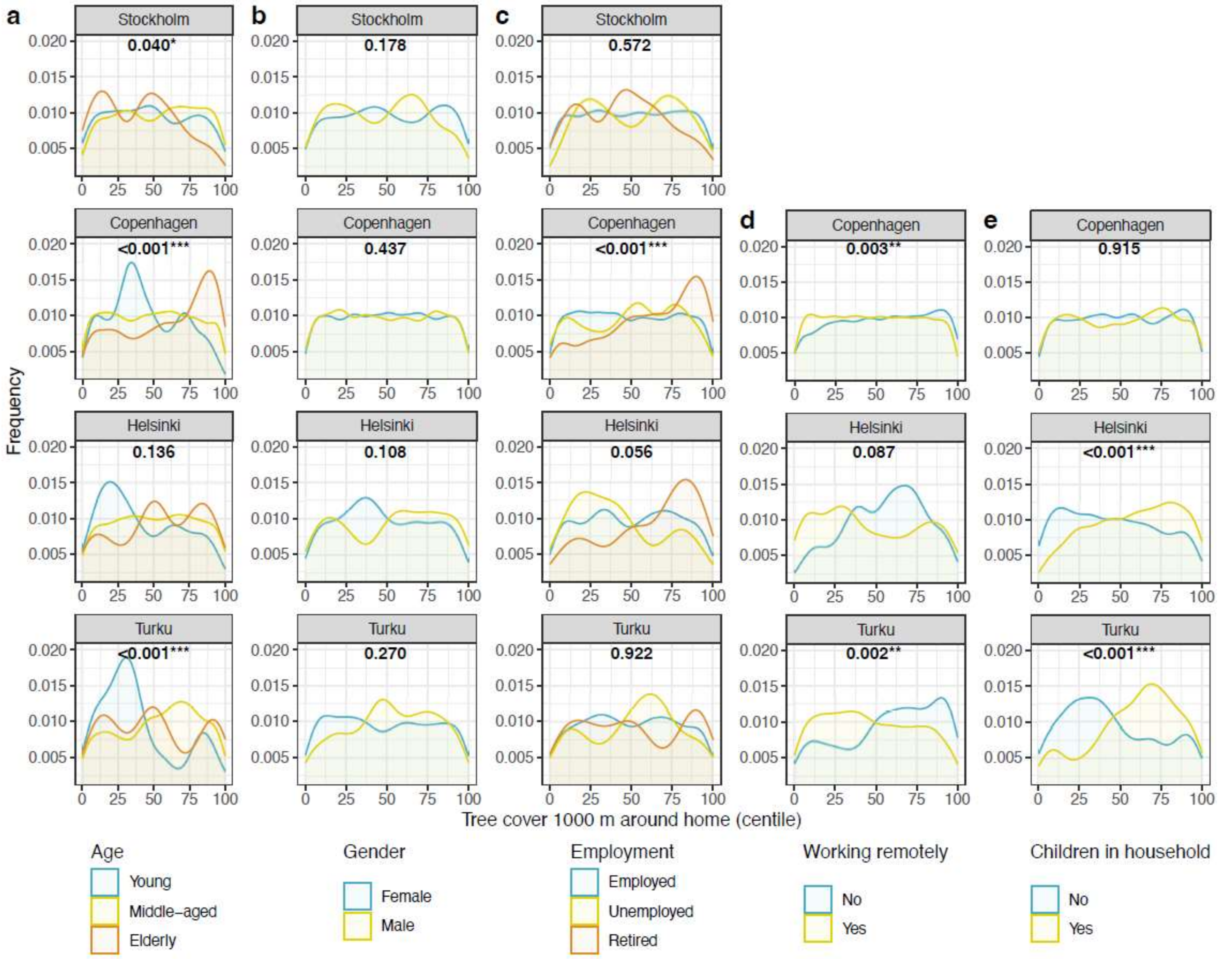

Supplementary Figure 9. Tree cover density (TCD) within 1000 m buffer distance around the place of residence. Data are broken down in each city for age (a), gender (b), employment (c), remote working (d), and children in household (e). The numbers in each plot correspond to the p-value for the Mann Whitney U-test or Kruskal-Wallis test for differences between groups shown in the plot. X axes show TCD in centlies of rank order, to illustrate group differences as estimated by the tests used (see Supplementary Figure 6 for absolute values in each city). See also Supplementary Tables 2, 4, 6 and 8 on statistical results as tables.

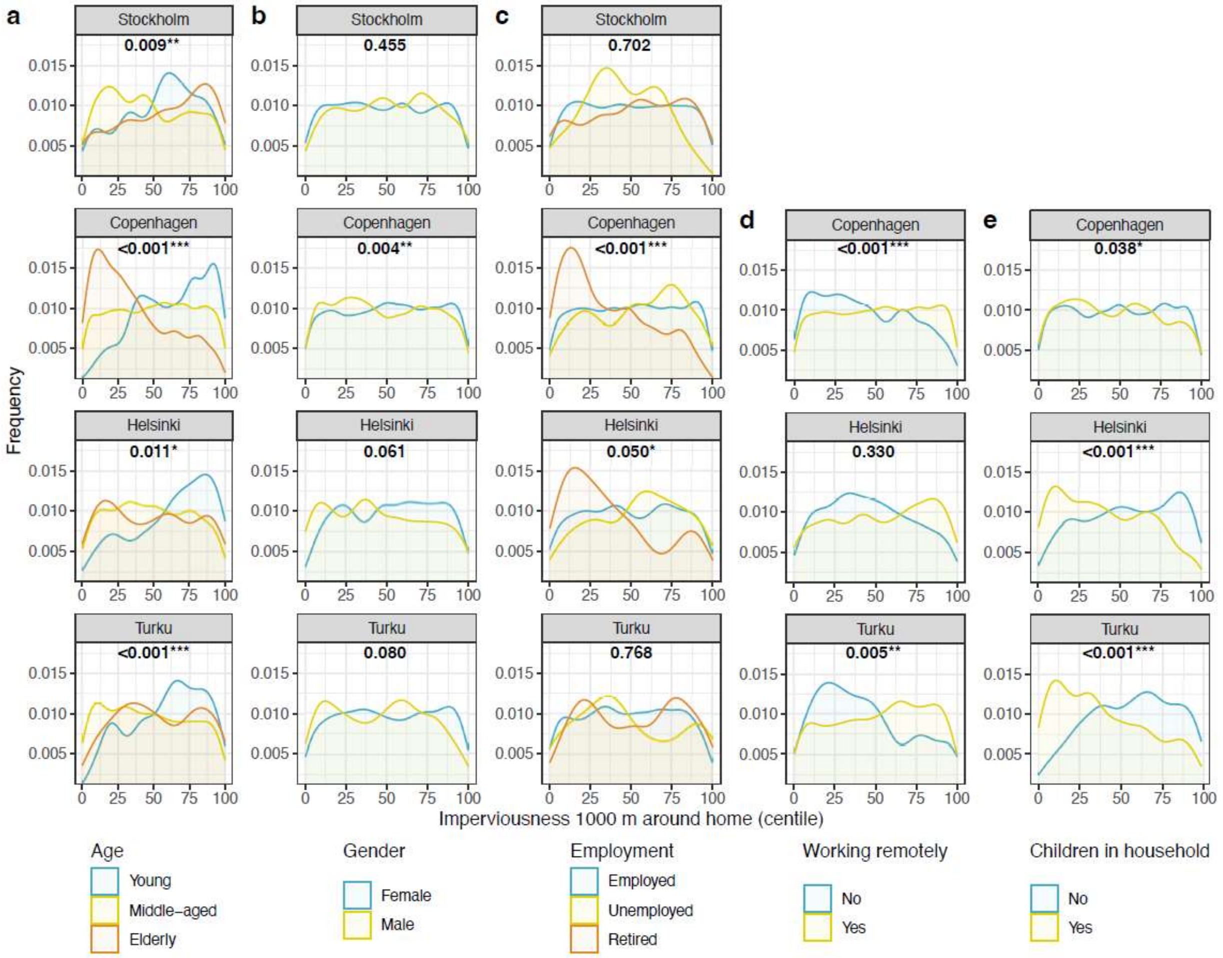

Supplementary Figure 10. Imperviousness density (ID) within 1000 m buffer distance around the place of residence. Data are broken down in each city for age (a), gender (b), employment (c), remote working (d), and children in household (e). The numbers in each plot correspond to the p-value for the Mann Whitney U-test or Kruskal-Wallis test for differences between groups shown in the plot. X axes show TCD in centlies of rank order, to illustrate group differences as estimated by the tests used (see Supplementary Figure 6 for absolute values in each city). See also Supplementary Tables 2, 4, 6 and 8 on statistical results as tables.

Distance between residence and water elements

In terms of age, closest to water lived the elderly people in Copenhagen (median 1.0 km, 0.4–0.5 km lower compared to other groups, $p<0.001$***) and the young people in Turku (median 1.1 km, 0.6 km lower compared to other groups, $p<0.001$***) (Supplementary Fig. 11B). One statistically significant difference is observed in terms of employment, as the retired lived at the shortest distance to water elements in Copenhagen (median 1.0 km, 0.4–0.5 km lower compared to other groups, $p<0.001$***) (Supplementary Fig. 11D). Remote working due to COVID-19 denoted statistically significant shorter distance to water in Turku (median 1.3 km, 0.9 km lower compared to those who did not shift to distance working, $p<0.001$***) (Supplementary Fig. 11E). Also,

households without children show similar results in Turku (median 1.9 km, 0.6 km lower compared to households with children, *p*=0.002**) (Supplementary Fig. 11F).

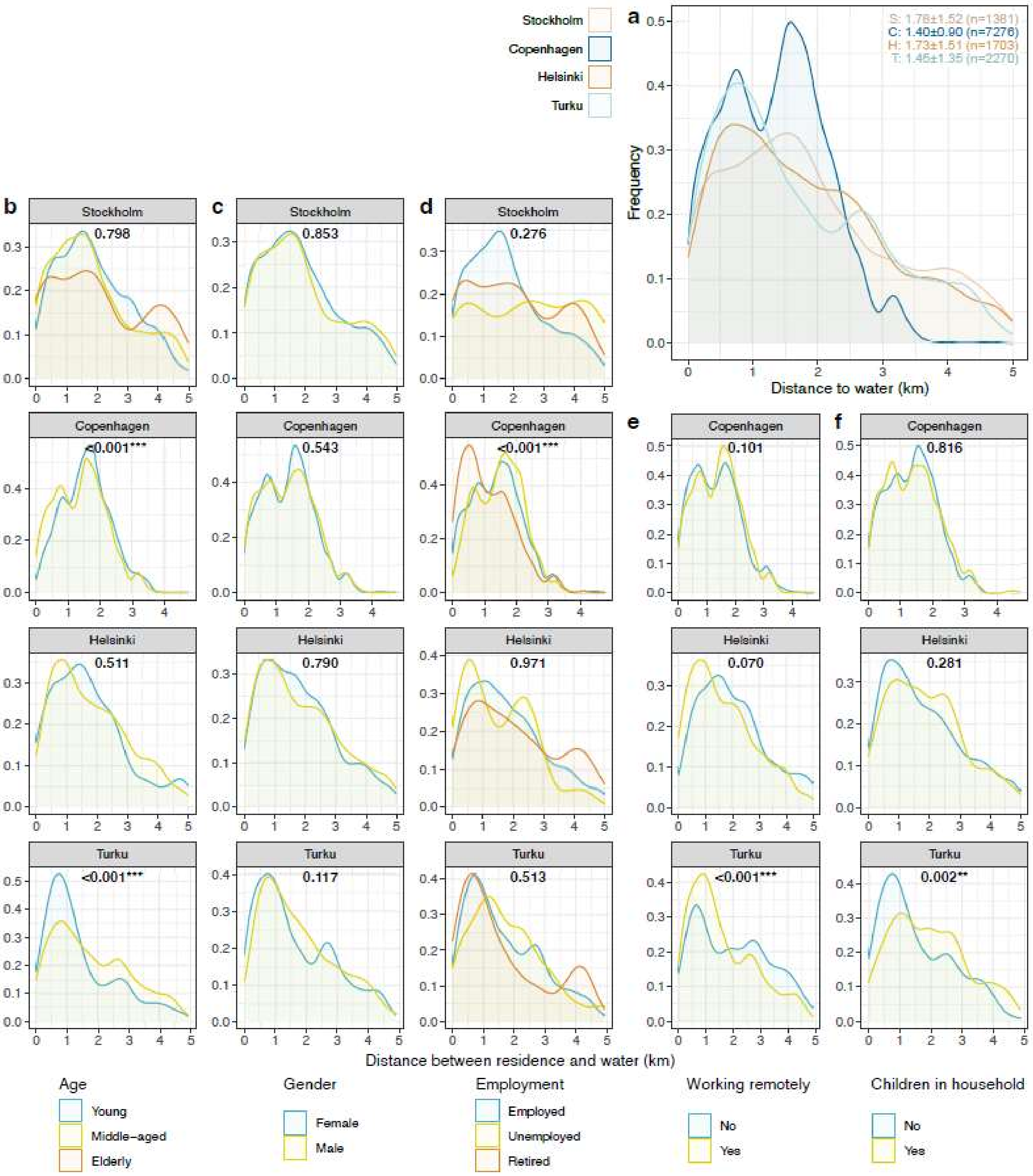

Supplementary Figure 11. Distance between respondent's place of residence and water elements. Walking distance in km through the street network is given on x axis and data point density estimates are given on y axis. (a) Data grouped by city, with median±MAD and number of outdoor recreation sites for each city in the upper right corner. Remaining panels show data broken down by city and (b) age, (c)

gender, (d) employment, (e) remote working, and (f) children in household. For panels B–F, the numbers in each plot correspond to the p-value for the Mann Whitney U-test or Kruskal-Wallis test for differences between groups shown in the plot. See also Supplementary Tables 2, 4, 6 and 8 on statistical results as tables.

Supplementary References

1. Nordh, H. & Olafsson, A. S. Plans for urban green infrastructure in Scandinavia. *J. Environ. Plan. Manag.* **64**, 883–904 (2020).
2. O'Brien, L. *et al.* Social and Environmental Justice: Diversity in Access to and Benefits from Urban Green Infrastructure – Examples from Europe. in *The Urban Forest. Future City* (eds. Pearlmutter *et al.*) 153–190 (Springer, Cham 2017).
3. Borges, L. A. *et al. White Paper on Nordic Sustainable Cities*. (Nordregio, 2017).
4. Caspersen, O. H., Konijnendijk, C. C. & Olafsson, A. S. Green space planning and land use: An assessment of urban regional and green structure planning in Greater Copenhagen. in *Geogr. Tidssk.* **106**, 7–20 (2006).
5. Zinko, U. *et al. Grön Infrastruktur i Urbana Miljöer*. (Nordic Council of Ministers, 2018).
6. Nielsen, A. B., Hedblom, M., Olafsson, A. S. & Wiström, B. Spatial configurations of urban forest in different landscape and socio-political contexts: identifying patterns for green infrastructure planning. *Urban Ecosyst.* **20**, 379–392 (2017).
7. ESPON. *Policy Brief: Green infrastructure in urban areas*. (2020).
8. EC (European Commision). *Communication from the Commission to the European Parliament, the Council, the European Economic and Social Committee and the Committee of the Regions COM/2020/3 - EU Biodiversity Strategy for 2030. Bringing nature back into our lives*. (2020).
9. Hale, T. *et al.* A global panel database of pandemic policies (Oxford COVID-19 Government Response Tracker). *Nat. Hum. Behav.* **5**, 529–538 (2021).
10. Petridou, E. Politics and administration in times of crisis: Explaining the Swedish response to the COVID-19 crisis. *Eur. Policy Anal.* **6**, 147–158 (2020).
11. Finnish government. Restrictions during the coronavirus epidemic.Available at: https://valtioneuvosto.fi/en/information-on-coronavirus/current-restrictions (Accessed: August 31, 2022) (2020)
12. The Danish Government. Corona: Covid-19. Available at: https://en.coronasmitte.dk/ (Accessed: August 31, 2022) (2020).
13. Folkhälsomyndigheten. Mars Nyheter - Folkhälsomyndigheten. Available at: https://www.folkhalsomyndigheten.se/nyheter-och-press/nyhetsarkiv/2020/mars/ (Accessed: August 31, 2022) (2020)
